# Clinical and prognostic significance of parathyroid hormone-related protein in breast cancer: a systematic review and meta-analyses of observational studies in women

**DOI:** 10.1530/ERC-25-0324

**Published:** 2026-03-05

**Authors:** Alisson Clemenceau, Juliette Bherer, Alyssa Grimshaw, Francine Durocher, Caroline Diorio, John J Wysolmerski

**Affiliations:** ^1^ Section of Endocrinology and Metabolism, Department of Internal Medicine, Yale School of Medicine, New Haven, Connecticut, USA; ^2^ CHU de Québec Research Center-Laval University, Quebec City, Quebec, Canada; ^3^ Department of Molecular Medicine, Cancer Research Center, Faculty of Medicine, Laval University, Quebec City, Quebec, Canada; ^4^ Harvey Cushing/John Hay Whitney Medical Library, Yale University, New Haven, Connecticut, USA; ^5^ Department of Social and Preventive Medicine, Cancer Research Center, Faculty of Medicine, Laval University, Quebec City, Quebec, Canada; ^6^ Center for Breast Diseases, Saint-Sacrement Hospital, Quebec City, Quebec, Canada

**Keywords:** PTHrP, breast, cancer, prognosis, hypercalcemia

## Abstract

Parathyroid hormone-related protein (PTHrP) is produced by normal breast epithelial cells and is a cause of hypercalcemia in breast and other cancers. However, there are conflicting data regarding its role(s) in breast cancer biology. This study aimed to clarify PTHrP’s potential function(s) in breast cancer by systematically examining its association with tumor characteristics, patient outcomes and hypercalcemia in women. We designed a predetermined strategy based on PRISMA 2020 guidelines. Seven databases were interrogated to identify studies of women with breast cancer reporting associations between tumor or circulating PTHrP levels and prognostic factors, calcium levels, relapse and survival. The ROBINS-E tool was used to examine the risk of bias. Quantitative syntheses of outcomes were performed by drawing forest plots of individual studies and pooling estimates. Forty-five studies met our eligibility criteria. We found that PTHrP is likely associated with positive progesterone receptor status, the presence of microcalcifications and lymph node invasion, but not with tumor type or grade. Most studies interrogating the association between PTHrP expression and breast cancer prognosis or hypercalcemia were at a very high risk of bias. Despite this, our results suggest associations between PTHrP and patient survival, the presence of bone metastases and the diagnosis of hypercalcemia of malignancy. These meta-analyses underscore the need for robust multivariate analyses in women to rigorously re-evaluate the role of PTHrP in breast cancer. This is important since the gene coding for PTHrP (*PTHLH*) has been consistently identified as a breast cancer susceptibility locus.

## Introduction

Parathyroid hormone-related protein (PTHrP) was discovered as the cause of a common paraneoplastic syndrome, known as humoral hypercalcemia of malignancy, in which tumor cells secrete PTHrP to cause elevations in serum calcium independent of bone metastases (1, 2, 3, 4, 5, 6, 7). The PTHrP gene (*PTHLH*) shares high sequence homology with the parathyroid hormone (PTH) gene, and both peptides activate the same receptor, the type 1 PTH/PTHrP receptor (PTH1R) (5, 7). Under physiological conditions, PTHrP is produced by many tissues and functions predominantly as a local paracrine or autocrine factor, with minimal presence in the systemic circulation. In contrast, in malignancy, excessive tumor-derived PTHrP gains access to the circulation and acts as an endocrine hormone, interacting with the PTH1R in bone and kidney to drive hypercalcemia (8, 9, 10, 11, 12, 13).

PTHrP has important functions during the development of several organs, including the breast, where it is required for outgrowth of the embryonic mammary bud (14, 15, 16). It also has important functions in lactation physiology. PTHrP is secreted into the circulation, where it acts systemically on the PTH1R in bone to mobilize calcium stores from the maternal skeleton to be used for milk production (17, 18). Beyond these normal roles, investigators have examined whether PTHrP contributes to breast carcinogenesis (19, 20, 21, 22) and whether its expression correlates with breast cancer (BC) prognosis (23, 24, 25, 26, 27, 28, 29, 30, 31, 32, 33, 34, 35, 36, 37, 38, 39, 40, 41, 42). However, studies have reported conflicting results and the potential contributions of PTHrP to BC diagnosis, prognosis and/or treatment remain uncertain. Nevertheless, a series of genome-wide association studies (GWASs) have shown that a single nucleotide polymorphism near *PTHLH* (rs10771399) is associated with the risk of BC (43, 44, 45, 46, 47, 48, 49), underscoring the need for a deeper understanding of how PTHrP affects BC behavior.

To start addressing this gap, we reviewed 35 years of human studies focusing on the association between PTHrP and prognosis in women diagnosed with BC and used meta-analyses to examine relationships between PTHrP levels and prognostic characteristics of breast tumors and BC outcomes. Our aim was to refine existing hypotheses and propose new directions for investigating the molecular functions of PTHrP in BC development and progression.

## Materials and methods

This systematic review was conducted following the general methods for Cochrane reviews (50) and reported according to a predetermined strategy based on the Preferred Items for Systematic Reviews and Meta-Analyses (PRISMA) 2020 guidelines (Supplementary Table 1 (see section on Supplementary materials given at the end of the article)) (51, 52). The protocol was registered in PROSPERO (CRD42024501601).

### Search methods for identification of studies

Seven electronic databases (Cochrane Library, Google Scholar, Ovid Embase, Ovid MEDLINE, PubMed, Scopus and Web of Science Core Collection) were used to identify manuscripts from inception to January 21, 2025. Search strategies were developed by an experienced clinical librarian and then validated by a second clinical librarian (53). Text keywords and control terms referring to ‘PTHrP’ or ‘*PTHLH*’ and ‘BC’ were used (Supplementary Table 2). Forward and backward citation chasing was performed using Citationchaser (54, 55) to identify additional relevant studies not retrieved by the database search, and these results were manually screened by one reviewer.

### Eligibility criteria

#### Type of studies

Any observational study evaluating the association between PTHrP and/or *PTHLH* and BC outcomes in humans was eligible for inclusion. Only peer-reviewed and fully published studies, available as full texts in English, were included. Conference abstracts were excluded as they lack sufficient information regarding eligibility criteria and data extraction items and would be at an overall high risk of bias due to poor reporting (56). No restriction was applied regarding type of publication.

#### Type of participants

All women diagnosed with an invasive BC and/or a carcinoma *in situ* were eligible. When there were no details about the sex assigned at birth of the participants, we assumed they were females, since only 0.9% of BCs newly diagnosed in 2025 will be in males (57). Thus, the proportion of male BCs in the populations of the included studies should be marginal.

#### PTHrP measurement

Studies that measured the expression of PTHrP and/or *PTHLH* mRNA in blood and/or breast tissue, regardless of the method, were eligible.

#### BC outcomes

All studies reporting BC outcomes, including overall survival (OS), BC-specific survival, BC-free survival, distant metastasis-free survival, relapse-free survival, preferential sites of metastases and BC-associated hypercalcemia, were eligible.

Studies assessing the association between PTHrP and/or *PTHLH* mRNA and well-established BC prognostic factors, such as age, stage, tumor size, lymph node involvement, grade, histologic type, molecular subtypes, estrogen receptor (ER) and progesterone receptor (PR) status, human epidermal growth factor 2 receptor (HER2) status and treatment regimen, were also eligible for inclusion.

### Exclusion criteria

We excluded studies according to the following criteria: i) abstract or full text unavailable, ii) not in English, iii) not peer-reviewed or not fully published, iv) not an analysis of the association between PTHrP/*PTHLH* and BC outcomes as defined in eligibility criteria, v) not in humans and vi) not in women.

### Data collection and analysis

#### Selection of studies

Deduplication was performed using conditional formatting and manual scanning. All references identified by the search strategy were reviewed in a two-step process. First, titles and abstracts were screened by two reviewers to exclude obviously non-eligible studies. Disagreements between the two reviewers were resolved by consensus, and if required, a third reviewer was consulted. Second, full-text articles were examined by two reviewers and subjected to evaluation based on predefined selection criteria. Disagreements between the two reviewers were resolved by consensus, and if required, a third reviewer was consulted. The Covidence software was used for recording decisions.

#### Data extraction

Data extraction was performed by two different reviewers using exhaustive forms designed for this review to ensure reliability. We extracted information about the study population, tumor characteristics, methods for PTHrP and/or *PTHLH* measurements, statistical analyses, variables used for adjustment and study results. The study’s definition of each variable included in the models was recorded as well as their treatment (categorical or continuous). If multiple publications were based on the same set of patients, the publication reporting the outcome of interest or the one with the longest follow-up was considered as reference. In addition, any required information unavailable in the reference publication that was available in the secondary publications has also been extracted. Extracted data were compared, with any discrepancies being resolved through a discussion.

#### Assessment of risk of bias in included studies

The ‘Reporting recommendations for tumor MARKer prognostic studies (REMARK)’ (58) and the domain-based tools, i.e., the ‘Grades of Recommendation, Assessment, Development, and Evaluation’ (GRADE) approach (59) and the ‘Risk Of Bias In Non-Randomized follow-up Studies – of Exposure’ (ROBINS-E) (60), were used to identify and rate the risk of bias in the included studies. The following domains were evaluated: selection of participants, PTHrP and/or *PTHLH* measurements, measurements of BC prognostic variables, missing data and selection of reported results. Evaluations were performed collegially by two reviewers, and a third reviewer was consulted when required. We derived an overall summary risk of bias judgment for each study based on the highest risk level in any domains that were assessed. The robvis visualization tool was used to create risk-of-bias plots (https://www.riskofbias.info/welcome/robvis-visualization-tool, accessed on January 10, 2025).

#### Data analysis

Odds ratios (ORs) and corresponding 95% confidence intervals (95% CIs) were calculated to assess the association studies between PTHrP/*PTHLH* expression and BC prognostic factors. Crude ORs from methodologically similar studies were pooled using the Mantel–Haenszel method under a random-effects model to account for within- and between-study variability. Forest plots were generated using the *forest* function of the *meta* package (61) to visualize the results. Publication bias risk was assessed using a contour-enhanced funnel plot generated with the *funnel* function of the *meta* package (61) when at least 10 studies were pooled. All analyses were performed in R, version 4.3.2 (R Foundation for Statistical Computing, Austria), within RStudio, version 2023.12.1 + 402.

#### Assessment of between-study heterogeneity

To quantify the effect of variability due to between-study heterogeneity on BC outcomes, the *I^2^* statistic was calculated using the forest plots of individual study results (62). To do so, the *metabin* function of the *meta* R package was used (61) in RStudio, version 2023.12.1 + 402, and R, version 4.3.2. The *I^2^* statistic was interpreted as follows: 0–40%, ‘might not be important’; 30–60%, ‘may represent moderate heterogeneity’; 50–90%, ‘may represent substantial heterogeneity’; and 75–100%, ‘considerable heterogeneity’. Then, the major differences between studies were explored to identify potential sources of heterogeneity.

#### Data synthesis

A systematic qualitative synthesis of the study characteristics, including the study population, tumor characteristics, PTHrP and/or *PTHLH* measurements and BC outcomes measurements, is available for each outcome in Supplementary Tables. A quantitative synthesis of outcomes was done by drawing forest plots of individual studies and pooling estimates.

#### Analysis of subgroups

Because we identified a high level of heterogeneity in the methodology used for PTHrP assessment, we have performed subgroup analyses according to the methods of exposure measurement, as well as the targeted PTHrP isoforms/portions.

## Results

**Figure 1:**
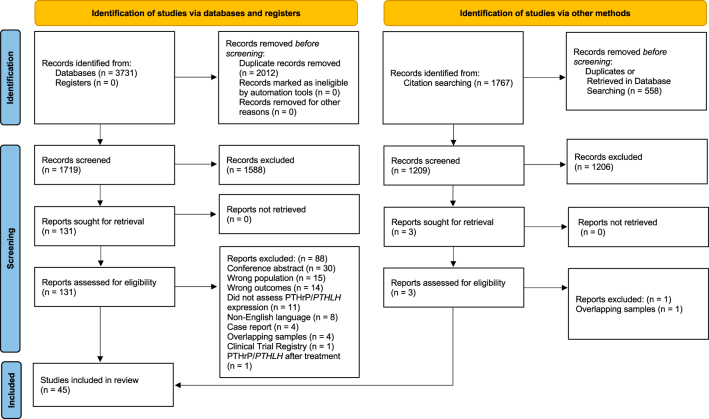
PRISMA flow diagram. Adapted from Page *et al.* (51) (for more information, visit: https://www.prisma-statement.org/). A full color version of this figure is available at https://doi.org/10.1530/ERC-25-0324.

Database searches identified 3,731 citations, among which 45 met eligibility criteria (Fig. 1).

Supplementary Table 3 provides a comprehensive overview of the rationale for article exclusion following full-text screening.

### Association between PTHrP and BC prognostic factors

**Figure 2:**
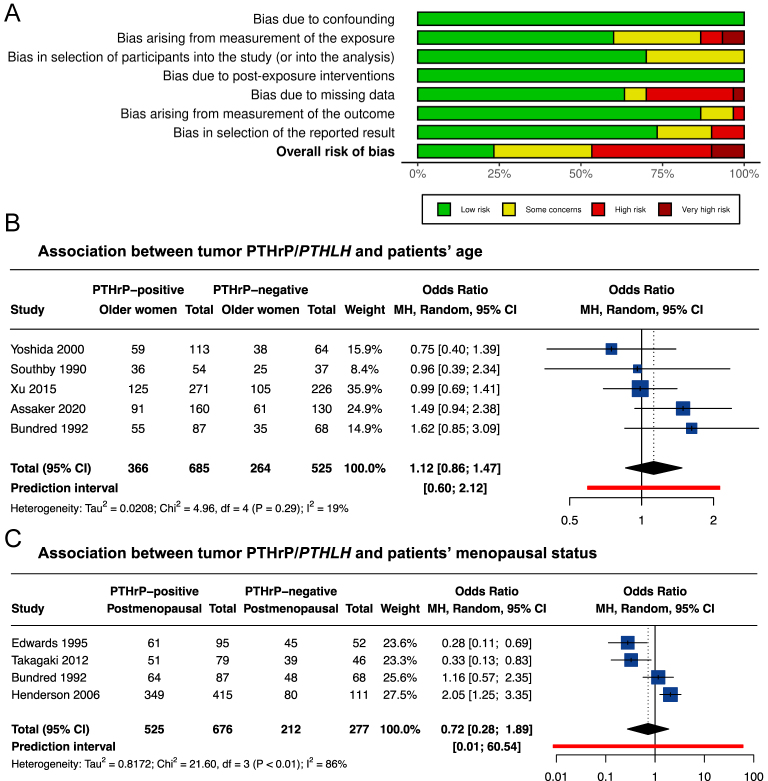
Association between PTHrP/*PTHLH* and patients’ characteristics. (A) Summary plot of risk-of-bias assessment in studies having examined the association between PTHrP and breast cancer prognostic factors. The ROBINS-E tool has been used. (B) Forest plot for univariate comparison of PTHrP-positive *versus* PTHrP-negative tumors with patients’ age. The age variable has been categorized in ‘older’ *versus* ‘younger’ women according to the age categories presented by the authors. Fifty years was the threshold in 4/5 studies, while 55 year was the threshold in 1/5 studies (Xu *et al.* (39)). Odds ratio <1 means that PTHrP-positive tumors were less frequent in older women compared to younger women, while odds ratio >1 means that PTHrP-positive tumors were more frequent in older women compared to younger women. Random-effects model *P*-value = 0.3988. (C) Forest plot for univariate comparison of PTHrP-positive *versus* PTHrP-negative tumors with postmenopausal status. Odds ratio <1 means that PTHrP-positive tumors were less frequent in postmenopausal women compared to premenopausal women, while odds ratio >1 means that PTHrP-positive tumors were more frequent in postmenopausal women compared to premenopausal women. Random-effects model *P*-value = 0.5082. Abbreviations: MH, Mantel–Haenszel; 95% CI, 95% confidence interval; and df, degree of freedom. A full color version of this figure is available at https://doi.org/10.1530/ERC-25-0324.

To identify confounding variables that might influence associations between PTHrP and BC outcomes, we initially studied the relationship between PTHrP expression and standard prognostic factors in BC. For these analyses, we identified 29 studies conducted by 20 research groups between 1990 and 2025 (21, 23, 25, 26, 27, 28, 29, 30, 31, 35, 36, 38, 39, 40, 41, 42, 63, 64, 65, 66, 67, 68, 69, 70, 71, 72, 73, 74, 75). PTHrP/*PTHLH* expression was assessed in 8,983 tumors and 423 blood samples. The extraction sheets are available in Supplementary Tables 4 and 5, and the main results are summarized in Table 1. Most of the studies were at a high risk of bias arising from the measurement of the exposure and/or bias due to missing data (Fig. 2A, Supplementary Fig. 1).

**Table 1:** Main results of studies reporting associations of PTHrP/*PTHLH* expression and breast cancer prognostic factors (*n* = 28).

Prognostic factors	Total number of studies (*n*)	Number of studies reporting an association between prognostic factor and		No association (*n*)
		High PTHrP expression (*n*)	Low PTHrP expression (*n*)	
Breast tumor tissue (*n* = 25)				
Age (older)	12 (2,238)	0	3 (299)	9 (1,939)
Menopausal status (postmenopausal)	10 (1,370)	2 (649)	5 (495)	4 (300)
Tumor stage (advanced)	5 (1,196)	1 (497)	1 (526)	3 (173)
Tumor size (bigger tumor)	14 (2,219)	1 (497)	1 (526)	12 (1,196)
Lymph node status (positivity)	18 (2,685)	4^A^ (796)	1 (81)	13 (1,808)
Distant metastases (M1)	3 (425)	2 (248)	0	1 (177)
Grade (high)	16 (7,955)	1 (177)	2^B^ (5,559)	14^C^ (2,293)
Ki-67 score (high)	1 (107)	0	0	1 (107)
Histological type	10 (1,664)	1^D^ (35)	0	9^E^ (1,629)
Molecular subtypes	2 (148)	2^F^ (148)	0	0
ER status (positive)	18 (7,833)	4^G^ (6,162)	1^H^ (74)	14^I^ (1,671)
PR status (positive)	13 (1,514)	5 (812)	0	8^J^ (702)
HER2 status (positive)	2 (734)	1^K^ (45)	0	2^L^ (689)
Prognostic index score (high risk)	3 (308)	0	1^M^ (102)	2 (206)
Breast tissue calcifications (presence)	6 (523)	4 (383)	0	2 (140)
Lymphovascular invasion (positive)	4 (1,088)	1 (123)	0	3 (965)
Skin involvement (positive)	1 (497)	0	0	1 (497)
Monocyte infiltration (positive)	1 (237)	1^K^ (45)	0	1^L^ (192)
Peripheral blood (*n* = 6)				
Age (older)	1 (54)	0	0	1 (54)
Tumor stage (advanced)	1 (54)	1 (54)	0	0
Tumor size (bigger tumor)	2 (88)	0	0	2 (88)
Lymph node status (positivity)	3 (136)	0	0	3 (136)
Distant metastases (M1)	1 (54)	0	0	1 (54)
Grade (high)	2 (102)	0	0	2 (102)
Ki-67 score (high)	1 (54)	0	0	1 (54)
Histological type	3 (113)	0	0	3 (113)
ER status (positive)	2 (102)	0	0	2 (102)
PR status (positive)	2 (102)	0	0	2 (102)
HER2 status (positive)	1 (54)	0	0	1 (54)
Lymphovascular invasion (positive)	1 (54)	0	0	1 (54)

Note: in some studies, several isoforms of PTHrP have been interrogated. In a same cohort of patients, one isoform could have been associated with a prognostic factor, while the other ones have not. This explains that the sum of study/patients shown in the three columns from the right might exceed the total presented on the left.

^A^ Two out of four studies have reported an association in premenopausal women only.

^B^ One study included four cohorts. In the other one, PTHrP(139) expression was associated with grade II specifically.

^C^ One study has reported an association in pre- and postmenopausal women separately.

^D^ PTHrP/*PTHLH* was associated with papillotubular adenocarcinoma.

^E^ One out of eight studies have reported an absence of association in pre- and postmenopausal women separately.

^F^ The number of PTHrP/*PTHLH*-positive cells was higher in TNBC compared to ER-positive and HER2-positive tumors.

^G^ One out of three studies have reported an association between PTHrP/*PTHLH* and ER status in pre- and postmenopausal women separately, and 1/3 studies has performed the analyses in four cohorts.

^H^ P2-initiated PTHrP specifically.

^I^ One out of thirteen studies has reported an absence of association in all patients, in pre- and in postmenopausal women.

^J^ One out of eight studies has reported an absence of association in pre- and postmenopausal women separately.

^K^ In ductal carcinoma *in situ* only.

^L^ In invasive breast cancer only.

^M^ St. Vincent’s Hospital prognostic index score (>27 *versus* ≤27).

### PTHrP measurements in tumors

The clinicopathological characteristics of the study populations are similar to those presented in the American Cancer Society’s update on female BC statistics in the USA 2022 (76). Indeed, 67% of evaluable patients were ≥50 years old, 75% were postmenopausal, 97% of the patients suffered from locoregional tumors, and 47% had intermediate-grade tumors. However, the cohorts had a lower rate of ER-positive tumors (range 19–79%) compared to the American population of BC patients (80%). This might be explained by the high rate of missing data (62%) and by the heterogeneity of the methods and thresholds used to define ER-positivity (Supplementary Tables 4 and 5).

### Patient characteristics

#### Age and menopausal status

Overall, 12 studies examined the association between tumor PTHrP expression and patients’ age (Table 1) (23, 25, 26, 28, 31, 35, 39, 41, 42, 66, 67, 68). Authors assessed PTHrP levels by immunohistochemistry, using semi-quantitative (23, 31, 42, 68, 77) or qualitative methods (25, 35, 39, 41), and compared either the frequencies of PTHrP-positive/negative tumors in different age categories (23, 25, 31, 39, 41) or the average age in PTHrP-positive *versus* PTHrP-negative group (35, 68). A meta-analysis of the five studies that reported the frequencies of PTHrP-positive/negative tumors in different age categories does not support any association between tumor PTHrP and patients’ age (Fig. 2B). In addition, three studies used quantitative approaches to measure PTHrP/*PTHLH* (26, 28, 66) and performed correlation analyses between PTHrP/*PTHLH* levels and age as continuous variables. All three reported an association between low PTHrP/*PTHLH* and older age. However, only two of these studies reported the coefficient of correlations (−0.4 and −0.2). Therefore, a meta-analysis was not performed.

Ten studies examined the relationship between PTHrP and menopausal status, defining postmenopausal as the absence of menstrual periods for 12 consecutive months (Table 1) (25, 26, 28, 30, 35, 38, 66, 72, 75, 77). In 4/10 studies, authors compared the PTHrP-positive/negative tumor frequencies according to menopausal status (Fig. 2C) (25, 35, 38, 66). The available data are variable due to between-study heterogeneity, potentially stemming from differences in study populations. For example, ER-positive samples comprised 67% of the Henderson *et al.* (35) cohort but only 39–49% of other cohorts (25, 38, 66) (Supplementary Tables 4 and 5). In the six other reports, authors compared quantitative or semi-quantitative PTHrP measurement in premenopausal *versus* postmenopausal women (26, 28, 30, 66, 72, 75). The heterogeneity of the reported estimates units did not allow us to perform a meta-analysis. However, PTHrP(1–86) (28, 66), *PTHLH* (exons 3–4) (72) and *PTHLH*(141) (30) levels were associated with premenopausal status, while *PTHLH* (exon 4), *PTHLH*(139) and *PTHLH*(173) levels were not (26, 30). In the most recent study, authors reported an association between a higher tumor PTHrP expression, measured by the histochemical score (H-score), a semi-quantitative method that combines staining intensity and the percentage of positive cells, and postmenopausal status (75). However, the study population was enriched in patients with positive lymph nodes (78%) compared to other studies (range 32–69%).

Overall, there is an absence of solid evidence supporting any association between tumor PTHrP and patient’s age and menopausal status. Further studies using robust methodologies are required to confirm or refute this possibility.

### Tumor characteristics

#### Estrogen receptor (ER) status

**Figure 3:**
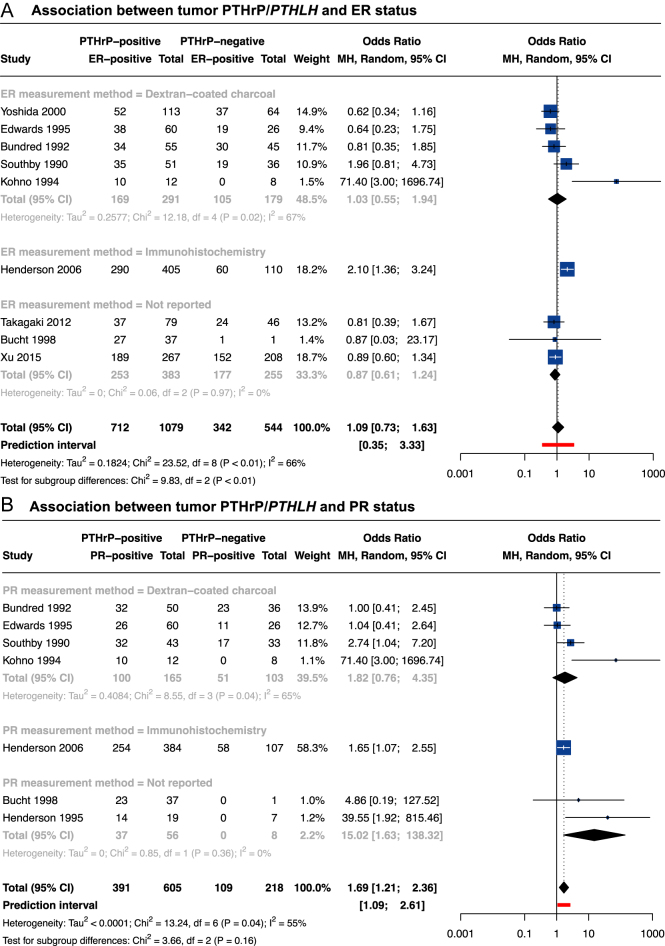
Association between PTHrP/*PTHLH* and tumor molecular subtype. (A) Forest plot for univariate comparison of PTHrP-positive *versus* PTHrP-negative tumors with ER status. Odds ratio <1 means that PTHrP-positive tumors were less frequent in ER-positive tumors compared to ER-negative tumors, while odds ratio >1 means that PTHrP-positive tumors were more frequent in ER-positive tumors compared to ER-negative tumors. Random-effects model *P*-value = 0.6829. (B) Forest plot for univariate comparison of PTHrP-positive *versus* PTHrP-negative tumors with PR status. Odds ratio <1 means that PTHrP-positive tumors were less frequent in PR-positive tumors compared to PR-negative tumors, while odds ratio >1 means that PTHrP-positive tumors were more frequent in PR-positive tumors compared to PR-negative tumors. Random-effects model *P*-value = 0.0020. Of note, data from Downey *et al.* (68) were excluded as the number of patients with ER/PR-positive or ER/PR-negative tumors presented in the manuscript appeared inaccurate. Abbreviations: MH, Mantel–Haenszel; 95% CI, 95% confidence interval; and df, degree of freedom. A full color version of this figure is available at https://doi.org/10.1530/ERC-25-0324.

Patients from the 18 identified studies were diagnosed between 1962 and 2022 (23, 25, 26, 30, 31, 35, 38, 39, 40, 42, 64, 66, 68, 70, 71, 72, 75, 77). Standard medical practices evolved during the recruitment period, so the methods and thresholds used to segregate ER-positive tumors from ER-negative tumors varied (Supplementary Tables 4 and 5). Thirteen studies examined PTHrP/*PTHLH* and ER expression qualitatively (23, 25, 31, 35, 38, 39, 42, 64, 66, 68, 70, 71, 77). The frequencies were reported in nine studies, allowing a meta-analysis (Fig. 3A) (23, 25, 31, 35, 38, 39, 42, 70, 78). The combined data do not support any association between tumor PTHrP/*PTHLH* expression and ER status and are of substantial variability due to between-study heterogeneity likely due to differing methods used for defining ER-positivity. In four studies, tumor PTHrP/*PTHLH* levels were assessed by quantitative or semi-quantitative measurement by PCR (26, 72), immunohistochemistry (75), immunofluorescence-based immunohistochemistry (40) or microarray (40). In PCR analyses (*n* = 38 and 35), *PTHLH* mRNA levels were not associated with ER status (26, 72). In contrast, Tran *et al.* reported that PTHrP/*PTHLH* expression was associated with ER-positive tumors in four cohorts representing a total of 5,485 patients (40). Similarly, Shalaby *et al.* reported that a higher PTHrP H-score was associated with ER-positivity (75). Inconsistent methods and units prevent data pooling, hindering conclusions about tumor PTHrP and ER status association. Further studies using current St. Gallen consortium criteria (79) are required to answer this question.

#### Progesterone receptor (PR) status

Methods and thresholds used to segregate PR-positive from PR-negative tumors in the 13 included studies were also highly heterogeneous (Supplementary Tables 4 and 5) (23, 25, 26, 30, 35, 42, 64, 66, 68, 70, 71, 75, 77). In 11 studies, both PTHrP/*PTHLH* and PR were studied as qualitative variables (23, 25, 30, 35, 42, 64, 66, 68, 70, 71, 77). The frequencies were available for estimate pooling in 7/11 studies (23, 25, 35, 42, 66, 70, 77). The meta-analysis showed that PTHrP-positive tumors were more frequently PR-positive than PTHrP-negative tumors (Fig. 3B). The moderate between-study variability may notably be explained by the different methodologies used for PR assessment. Shalady *et al.* compared the average PTHrP H-scores in PR-positive *versus* PR-negative tumors (75). A higher PTHrP H-score was associated with tumor PR-positivity. Finally, in three studies, PTHrP/*PTHLH* measurements were assessed quantitatively either by PCR (26, 30) or immunoradiometric assay (66). All three studies reported an absence of association between PTHrP/*PTHLH* expression and PR status. Overall, there is uncertainty regarding relations between PTHrP and tumor PR status in women. Detection of PTHrP using qualitative and semi-quantitative methods suggests that tumor PTHrP expression is associated with tumor PR-positive status, but available quantitative analyses do not support this hypothesis. Therefore, further studies are needed to reach a firm consensus.

#### HER2 and molecular subtype

Three studies investigated the association between tumor (39, 73) or blood (74) PTHrP/*PTHLH* levels and HER2 status (Table 1). These data do not support any association between PTHrP/*PTHLH* expression and HER2 status in BC patients (Supplementary Fig. 2). Two studies examined the association between tumor PTHrP and BC molecular subtypes (21, 75). In one study (*n* = 25), the percentage of *PTHLH*-positive tumor cells was higher in triple-negative BC (TNBC, ∼9%) compared to ER-positive and HER2-positive tumors (∼2%) (21). In the second study (*n* = 123), a higher PTHrP H-score was associated with luminal B subtype (75). These results obtained using distinct methodologies in small study populations do not allow any consensus. However, determining whether PTHrP expression is associated with BC molecular subtypes is an important issue for future studies.

#### Tumor stage

In 3 of 5 included studies, PTHrP was assessed by immunohistochemistry and was treated as a qualitative variable (Supplementary Tables 4 and 5) (23, 35, 39). Thus, we performed a meta-analysis comparing the frequency of advanced tumors (stages III–IV) in PTHrP-positive *versus* PTHrP-negative tumors. The estimate pooling does not support any statistical association between PTHrP and tumor stage (Supplementary Fig. 3A). However, the meta-analysis shows considerable variability due to between-study heterogeneity and neither the methodologies used for outcome/exposure measurements, nor the study population characteristics can clearly explain such discrepancies. One study compared mean *PTHLH* expression between tumor stages and found similar levels of expression among patients with stage 1, 2 or 3 tumors (26). In the last study, authors did not detail the methods but mentioned that PTHrP was not associated with tumor stage in their study population (77). Altogether, these data suggest an absence of evidence rather than evidence of absence of association between tumor PTHrP/*PTHLH* and BC stage.

We also examined the relationship between PTHrP/*PTHLH* expression and each component of the modern American Joint Committee on Cancer (AJCC) staging system, which classifies cancers by tumor size, lymph node involvement and distant metastasis, to assign an overall stage from 0 (*in situ*) to IV (advanced) (80). After reviewing 14 studies, we found no evidence regarding any association between tumor PTHrP expression and tumor size (Supplementary Fig. 3B) (23, 25, 26, 31, 35, 38, 39, 41, 42, 68, 69, 70, 72, 77). The average PTHrP expression level was compared between tumor size categories in 2/14 studies (26, 72), while the average and/or median tumor size was compared between different categories of PTHrP expression in 5/14 studies (23, 25, 35, 68, 70). The heterogeneity in the reported estimates and in PTHrP categorization prevented meta-analysis. In six studies, authors reported the frequencies of PTHrP-positive/negative tumors according to tumor size categories (31, 35, 38, 39, 41, 69), and in four of them, authors have compared tumors smaller/larger than 2 cm (38, 39, 41, 77). The meta-analysis shows, however, a considerable variability due to between-study heterogeneity (Supplementary Fig. 3B), and the available data did not allow for identification of its cause. The two remaining studies did not detail how tumor size was characterized (42, 77). Thus, the available literature cannot rigorously determine any association between PTHrP/*PTHLH* and tumor size.

**Figure 4:**
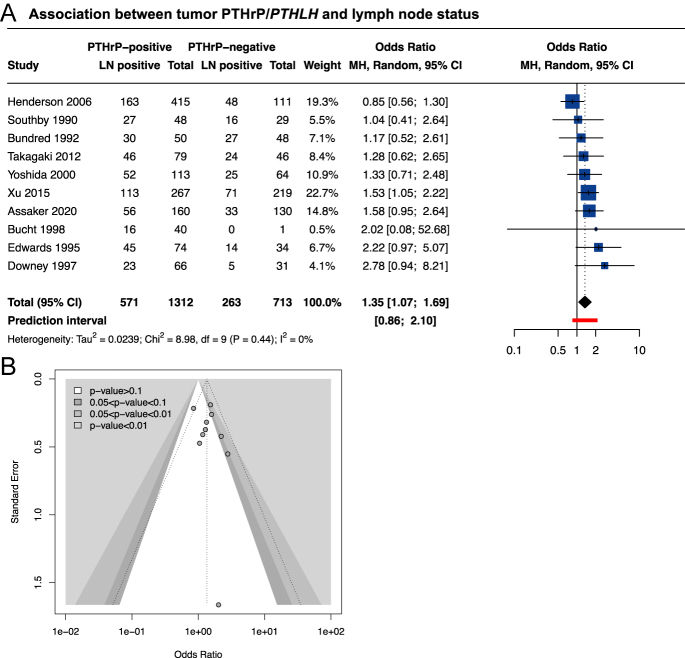
Association between PTHrP/*PTHLH* and tumor stage. (A) Forest plot for univariate comparison of PTHrP-positive *versus* PTHrP-negative tumors with lymph node status. Odds ratio <1 means that lymph node-positive tumors were less frequent in PTHrP-positive tumors compared to PTHrP-negative tumors, while odds ratio >1 means that lymph node-positive tumors were more frequent in PTHrP-positive tumors compared to PTHrP-negative tumors. Random-effects model *P*-value = 0.0109. (B) Contour enhanced funnel plot for association studies between PTHrP expression and patients’ lymph node status. Abbreviations: LN, lymph node; MH, Mantel–Haenszel; 95% CI, 95% confidence interval; and df, degree of freedom. A full color version of this figure is available at https://doi.org/10.1530/ERC-25-0324.

Eighteen studies examined the association between tumor PTHrP/*PTHLH* expression and patients’ lymph node status (Table 1) (23, 25, 26, 28, 31, 35, 36, 38, 39, 41, 42, 64, 66, 68, 69, 70, 72, 77). In 10/18, the authors compared the frequencies of PTHrP-positive/negative tumors according to lymph node involvement (23, 25, 31, 35, 38, 39, 41, 66, 68, 70). Tumor PTHrP/*PTHLH* expression was consistently associated with a higher frequency of lymph node invasion (Fig. 4A). Furthermore, the risk of publication bias can reasonably be excluded (Fig. 4B). In addition, 4/18 reports included data on the average/median tumor PTHrP expression according to categorical lymph node status (26, 28, 36, 72). However, the heterogeneity of the estimates and the multiple categorizations of lymph node status did not allow us to perform a meta-analysis. Nevertheless, two of these studies (*n* = 159) reported an absence of association between the variables (36, 72), while the other two (*n* = 152) reported an association between PTHrP and the presence of lymph node invasion in all (26) or in premenopausal patients (28). In two other studies, the statistical strategy or the frequencies of PTHrP-positive/negative tumors according to the patients’ nodal status were not available (69, 77). Finally, one study reported that PTHrP-positivity was associated with less than three positive lymph nodes (64), while another study showed that the number of positive lymph nodes was not associated with qualitative tumor PTHrP (42).

Only two studies examined the distant metastasis status at the time of diagnosis according to qualitative PTHrP status (Supplementary Fig. 3C) (31, 38). The meta-analysis shows a higher frequency of stage 4 lesions in patients with PTHrP-positive tumors. One study compared the average percent PTHrP expression as well as the average PTHrP H-score in patients with or without distant metastases and reported an association between PTHrP-positivity and the presence of distant metastases in both cases (75). Overall, PTHrP seems to be associated with lymph node and distant metastases, suggesting that its role in BC cell invasion warrants investigation.

#### Pathological grade

In 10/16 included studies, the frequency of the PTHrP-positive and PTHrP-negative status was assessed in high-grade (III) *versus* low-grade (I and II) tumors (23, 25, 31, 35, 39, 41, 64, 66, 68, 70). The meta-analysis of these studies does not support any association between PTHrP expression and pathological grade (Supplementary Fig. 4A). The data were of moderate variability because of between-study heterogeneity, partly due to differing PTHrP measurement methods. The funnel plot is symmetrical; thus, we can reasonably exclude the existence of a publication bias (Supplementary Fig. 4B). Tumor PTHrP was assessed semi-quantitatively by immunohistochemistry in two studies; however, the frequency of PTHrP-positive/negative tumors according to pathological grade was not reported (42, 71). Four studies quantified tumor PTHrP/*PTHLH* levels by qPCR (26, 30, 36), immunofluorescence-based immunohistochemistry or microarray (40). The heterogeneity in the arbitrary units quantifying PTHrP expression prevented estimate pooling. *PTHLH* exon 4 (26), *PTHLH*(141) (30), *PTHLH*(173) (30) and *PTHLH* (36) levels were not associated with tumor grade, while *PTHLH*(139) expression was associated with grade II tumors (30). Tran *et al.* reported in four cohorts representing 5,485 patients that PTHrP and *PTHLH* mRNA expression were associated with low-grade tumors (40). Nevertheless, the overall data do not support any association between PTHrP/*PTHLH* and tumor grade.

#### Other prognostic factors

The list of studied prognostic factors is available in Table 1 and is detailed in Supplementary Tables 4 and 5. Available data do not support any association between qualitative tumor PTHrP/*PTHLH* and tumor type (Supplementary Fig. 5A) (23, 25, 35, 39, 64, 66, 70). Quantitative PTHrP assessments did not differ according to tumor histologic subtypes either (26, 72).

Tumor PTHrP levels may correlate with the presence of histological microcalcifications (Supplementary Fig. 5B) (23, 27, 28, 64). The frequencies of PTHrP-positive/negative tumors according to the presence of microcalcifications were only available for 4/6 studies (23, 26, 27, 28, 64, 65). Moreover, substantial between-study variability may partly result from differences in patients’ characteristics (Supplementary Tables 4 and 5). Thus, the association between PTHrP/*PTHLH* and the presence of histological microcalcifications warrants further study.

Qualitative assessment of tumor PTHrP was not associated with lymphovascular infiltration (Supplementary Fig. 5C) (35, 38, 41). However, patients with lymphovascular invasion had a higher percent PTHrP-positive cells compared to patients without lymphovascular invasion (75). Thus, it remains unclear whether tumor PTHrP/*PTHLH* is associated with lymphovascular invasion.

In summary, tumor PTHrP/*PTHLH* expression may be associated with PR-positive tumors, lymph node infiltration and the presence of histologic microcalcifications. Whether calcifications affect BC prognosis remains unclear (81, 82). Thus, association studies between PTHrP expression and BC prognosis should minimally adjust for PR status and lymph node involvement.

### PTHrP measurement in blood (plasma) samples

Six studies investigated the association between circulating PTHrP levels and BC prognostic factors (Supplementary Tables 4 and 5) (29, 63, 66, 69, 70, 74). However, in 2/6 studies, authors did not detect circulating PTHrP (29, 66). In addition, most of these studies were at a high risk of misclassification of the exposure due to the absence of protease inhibitors used during blood collection and/or imprecise data about sample handling and storage. In the absence of a protease inhibitor, more than 50% of PTHrP may be degraded in plasma at room temperature (83). The four other studies reported an absence of association between circulating PTHrP and prognostic factors, with the exception of one association between PTHrP levels and advanced tumor stage (Table 1) (74). The existing literature provides no conclusive link between BC prognostic factors and circulating PTHrP levels.

### Association between PTHrP and prognosis

The patients’ clinicopathological characteristics were similar to those observed in the general population of women diagnosed with BC in the USA (76). However, ER-positive tumors (range 36–88%) were less common than what is observed (80%) using current standards for ER-positivity (79). Thus, the number of ER-positive tumors might have been underestimated in these studies.

### Overall survival

Ten studies led by 7 research groups between 1992 and 2012 interrogated the association between tumor PTHrP/*PTHLH* expression and patient survival (Supplementary Tables 6 and 7) (25, 27, 28, 31, 33, 34, 35, 38, 40, 41). This represents 11 cohorts and 6,346 tumors, among which 72% were examined in one study (40).

Univariate analyses were performed in 8/10 studies (*n* = 6,044) and showed inconsistent results (25, 27, 28, 33, 34, 35, 40, 41). These studies all have a very high risk of bias due to confounding, thereby limiting their robustness. Six studies reported the results of multivariate survival analyses according to tumor PTHrP/*PTHLH* expression (Table 2, Supplementary Fig. 6) (31, 33, 35, 38, 40, 41). PTHrP/*PTHLH* expression displayed variable association with OS in different studies. Linforth *et al.* found that patients with tumors that were positive for both PTH1R and PTHrP had a worse prognosis than patients with PTH1R-positive but PTHrP-negative tumors (33). PTH1R was detected in 50–96% of the examined PTHrP-positive BCs (Supplementary Table 8) (68, 71). Thus, the association between PTHrP and survival might depend on PTH1R expression. In addition, 2/6 studies reported that tumor PTHrP expression was associated with a worse survival in patients without distant metastases at the time of diagnosis (31, 38), suggesting that higher PTHrP expression might predict a reduced survival in early BC. A meta-analysis was not conducted due to substantial heterogeneity among the included studies. Sources of heterogeneity included variation in study populations (all BC patients (33, 35, 40) *versus* TNBC only (41), *versus* non-metastatic primary BC (31, 38)), length of follow-up (ranging from 0.1 (41) to 243 months (38)), PTHrP assessment (binary (33, 35, 38, 40, 41) *versus* ternary (31) categorization) and statistical methodologies/reported estimates (including absence of reporting (33, 40) *versus* HRs (35, 41) and RRs (31, 38)).

**Table 2:** Main results of multivariate analyses comparing PTHrP/*PTHLH* and breast cancer prognosis (*n* = 7).

Study (year, country)	*n*	Follow-up	Adjustment variables (categorization)	Estimates	Overall risk of bias	Conclusion
**Overall survival**						
*All patients*						
Linforth *et al.* (2002, UK)	177	5 years	- Age (<50, 50–70, >70) - Tumor size (<20 mm, >20 mm) - Tumor grade (I, II, III) - Nodal status (+, −) - ER status (+, −) - PR status (+, −) - PTHrP receptor expression (+, −)	NR	High	No association
Henderson *et al.* (2006, Australia)	526	Minimum of 8 years, median = 10 years	- Lymph node status (0, 1–3, 4–9 and >9) - PR status - Log tumor size	HR = 0.47 95% CI (0.32–0.69)	Some concerns	Association between higher PTHrP expression and better survival outcome
Tran *et al.* (2018, USA)	737	Censored at 10 years	- Tumor size (>2.0 cm) - Age (>50) - Tumor grade (III) - Nodal status (−) - ER status (+)	NR	High	Association between higher PTHrP expression and better survival outcome
*In TNBC only*						
Assaker *et al.* (2020, Canada)	314	Median = 3.6 years, range 0.1–9.8 years	- Age at diagnosis (≤50 years, >50 years) - Tumor size (T2, T3 *versus* T1) - Lymph node status (N1, N2 *versus* N0) - Type of surgery (modified radical mastectomy, breast-conserving surgery) - Adjuvant chemotherapy (yes, no)	HR = 1.590 95% CI (0.925–2.736)	Some concerns	No association
*In patients without distant metastases at the time of diagnosis*						
Yoshida *et al.* (2000, Japan)	171	Mean = 6.1 years	- Age (≤50, >50) - Tumor size (≤2.5 cm, >2.5 cm) - Nodal status (−, +) - Grade (I, II, III) - ER status (−, +)	RR^A^ = 1.979 95% CI (1.090–3.590)	Low	Association between higher PTHrP expression and worse survival outcome
Takagaki *et al.* (2012, Japan)	116	Median = 97 months, range 5–243 months	- Menopausal status (pre-, postmenopausal) - Tumor size (T1–T3 *versus* T4) - Nodal status (−, +) - ER status (−, +) - Lymphatic infiltration (−, +) - Vascular invasion (−, +)	RR = 3.644 95% CI (1.182–15.880)	High	Association between higher PTHrP expression and worse survival outcome
**Recurrence-free survival**						
*In patients without distant metastases at the time of diagnosis*						
Xu *et al.* (2015, China)	497	Median = 48 months, range 2–85 months	- Tumor size (≤2 cm, >2 cm) - Skin involvement (no, yes) - Lymph node metastasis (no, yes) - Histologic grade (≤II, >II) - ER status (−, +) - TGF-β (+, −)	HR = 1.022 95% CI (0.684–1.527)	High	No association
**Bone metastasis development**						
*All patients*						
Yoshida *et al.* (2000, Japan)	177	Mean = 6.1 years	- Age (≤50, >50) - Tumor size (≤2.5 cm, >2.5 cm) - Nodal status (−, +) - Grade (I, II, III) - ER status (−, +)	OR^B^ = 3.131 95% CI (NR)	Low	Association between higher PTHrP and higher risk of bone metastasis development
Takagaki *et al.* (2012, Japan)	125	Median = 97 months, range 5–243 months	- Menopausal status (pre-, postmenopausal) - Tumor size (T1–T3 *versus* T4) - Nodal status (−, +) - ER status (−, +) - Lymphatic infiltration (−, +) - Vascular invasion (−, +)	OR = 7.104 95% CI (1.782–48.110)	High	Association between higher PTHrP and higher risk of bone metastasis development

Abbreviations: NR, not reported; HR, hazard ratio; 95% CI, 95% confidence interval; RR, relative risk; OR, odds ratio.

^A^ PTHrP was assessed semi-quantitatively and divided into three categories (−, + and ++).

^B^ PTHrP was divided into two categories (−, +/++). No association was found when PTHrP was divided in three categories (−, + and ++).

Overall, the current data are inconclusive regarding the association between breast tumor PTHrP/*PTHLH* expression and OS.

### Recurrence-free survival

Eleven studies, led by nine research groups, examined the association between tumor PTHrP/*PTHLH* expression and recurrence-free survival (Supplementary Tables 6 and 7) (23, 25, 27, 30, 31, 33, 34, 36, 39, 40, 41). This represents a total of 12 cohorts and 2,546 samples.

Six studies compared the frequency of relapse according to qualitative (23, 25, 27, 30, 39) or quantitative (30, 36) PTHrP/*PTHLH* expression in primary breast tumors from patients without distant metastases at the time of diagnosis. The conclusions of these reports were inconsistent (Supplementary Tables 6 and 7). Furthermore, patient’s follow-up length varied widely (2 months to 14 years), and no information about follow-up length by PTHrP/*PTHLH* status was available. Therefore, no conclusion can be made about the influence of tumor PTHrP on the risk of BC relapse.

Five studies (6 cohorts, *n* = 1,737) examined the univariate relationship between PTHrP expression and recurrence-free survival time, none of which reported any association (27, 33, 34, 40, 41). Meta-analysis was not performed since most estimates were not reported, and these crude analyses were at a very high risk of bias due to confounding. Tran *et al.* reported specifically that higher nuclear PTHrP staining was associated with an increased recurrence-free survival time in two independent cohorts (40). Thus, the association between tumor PTHrP and clinical outcome may depend on the intracellular localization of PTHrP. Finally, one study (*n* = 497) examined PTHrP/*PTHLH* expression with respect to recurrence-free survival after adjusting for potential confounding variables and found no association (Table 2, Supplementary Fig. 6) (39). Thus, limited evidence supports an association between PTHrP expression and BC recurrence.

### Development of bone metastases

Fifteen studies, led by 11 research groups, investigated possible links between PTHrP and bone metastases (Supplementary Tables 6 and 7) (24, 25, 26, 28, 29, 30, 31, 32, 34, 35, 37, 38, 39, 42, 75). Thirteen studies (14 cohorts, *n* = 2,252) measured PTHrP/*PTHLH* expression in breast tumors (24, 25, 26, 28, 30, 31, 34, 35, 37, 38, 39, 42, 75), while four studies (*n* = 498) measured PTHrP in blood (24, 28, 29, 32).

Six studies (*n* = 869) compared the development of bone metastases according to tumor PTHrP expression in patients with early-stage disease at the time of diagnosis (24, 25, 28, 30, 39, 42). In all six reports, the frequency of bone metastases was higher in patients with PTHrP-positive tumors compared to PTHrP-negative tumors. In addition, Bouizar *et al.* reported that higher levels of *PTHLH* mRNA were associated with bone metastases (26, 30). However, the follow-up length in these studies varied and, in most reports, it was unclear whether the follow-up length according to patients’ PTHrP status was similar. Therefore, the association between tumor PTHrP/*PTHLH* and bone metastasis risk remains to be fully addressed.

In 2/13 studies, the authors examined associations between tumor PTHrP/*PTHLH* and bone metastasis-free survival time (34, 35). Surowiak *et al.* observed an absence of association (34), while Henderson *et al.* reported that a higher PTHrP expression was associated with a longer bone metastasis-free survival (35). The heterogeneity in the reported estimates type prevented estimate pooling.

In 1/13 studies (*n* = 80), authors measured tumor PTHrP in patients with bone metastases at the time of diagnosis, compared to patients with early primary BC (37). PTHrP expression was similar in the two populations. Finally, 1/13 studies (*n* = 123) examined the percentage of PTHrP-positive cells in patients with or without bone metastases (75) and found that a higher percentage of PTHrP-positive cells was associated with the presence of bone metastases.

Two studies reported the results of multivariate logistic regression analyses (Table 2, Supplementary Fig. 6) (31, 38). Both reported that higher primary tumor PTHrP/*PTHLH* expression was associated with a higher likelihood of bone metastasis events.

In four studies, authors compared circulating PTHrP levels in patients with bone metastases *versus* early BC (24, 28, 29, 32). Estimate pooling was possible for 3/4 studies and does not support any association between circulating PTHrP levels and the diagnosis of bone metastases (Supplementary Fig. 7). Nevertheless, the results are of considerable variability due to between-study heterogeneity. None of the included studies detailed their blood collection and sample treatment methodologies. Thus, there is a high risk of information bias. Therefore, tumor PTHrP, but not circulating PTHrP, may be linked to bone metastasis development.

### Preferential sites of metastases

Sixteen studies led by 7 research groups examined the association between PTHrP/*PTHLH* expression and the preferential sites of BC metastases (Supplementary Table 9) (23, 26, 27, 30, 31, 35, 39, 41, 42, 65, 77, 84, 85, 86, 87). Of these, we excluded three studies because the same group of patients had been analyzed several times (85, 86, 88). PTHrP/*PTHLH* expression was assessed in tumors or metastases in 12/13 studies (*n* = 461) (23, 27, 30, 31, 35, 39, 41, 42, 65, 77, 84) and in blood in 1/13 studies (3 cohorts, *n* = 111) (87). Overall, some concerns were identified regarding potential bias arising from exposure measurement and in selection of participants (Supplementary Fig. 8).

**Figure 5:**
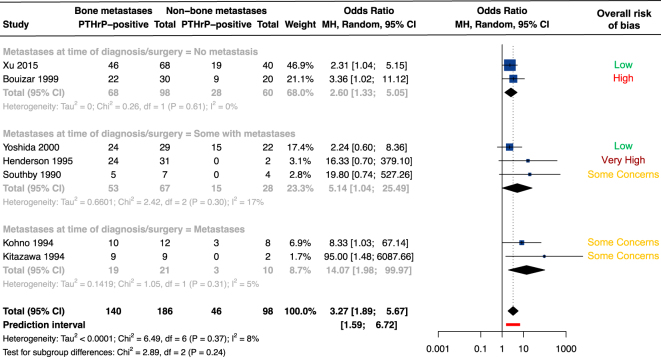
Association between PTHrP/*PTHLH* and breast cancer preferential site of metastases. Forest plot for crude comparison of PTHrP-positive *versus* PTHrP-negative tumors with the preferential site of metastases. Odd ratio <1 means that tumor PTHrP expression is more frequent in patients with non-osseous metastases compared to bone metastases, while odds ratio >1 means that tumor PTHrP expression is more frequent in patients with bone metastases compared to non-osseous metastases. Random-effects model *P*-value <0.0001. Of note, Bouizar *et al.* (30) examined the expression of *PTHLH*(139), *PTHLH*(141) and P2-initiated and P3-initiated transcripts. The frequencies of *PTHLH* detection according to the site of metastasis were reported for P2-initiated transcript only. Thus, the results of P2-initiated transcript only are presented in this figure. In addition, Kissin *et al.* (27) and Assaker *et al.* (41) were excluded from the meta-analysis as the patients had multiple sites of metastases and the presentation of the results did not allow us to identify those without bone metastases and their PTHrP status. Abbreviations: MH, Mantel–Haenszel; 95% CI, 95% confidence interval; and df, degree of freedom. A full color version of this figure is available at https://doi.org/10.1530/ERC-25-0324.

All reported results for PTHrP/*PTHLH* measurements in tumor tissue were assessed by univariate analyses. We did not identify any confounding variables that could be associated with both the preferential site of metastases and circulating PTHrP detection in BC patients. In 7/12 studies (*n* = 284), authors reported the frequencies of PTHrP/*PTHLH*-positive/negative primary tumors from patients having developed bone metastases compared to patients having developed non-osseous metastases (23, 30, 31, 39, 42, 65, 77, 84). The frequency of PTHrP-positive primary tumors was consistently higher in patients who subsequently developed bone metastases compared to non-bone metastases (Fig. 5). In addition, continuous *PTHLH*(139) and *PTHLH* exon 4 expression was higher in primary tumors from patients who subsequently developed bone metastases compared to patients who developed soft tissue metastases (26, 30). Conversely, *PTHLH*(141) and P3-initiated transcripts were not associated with a preferential site of metastases (30). These results support an association between tumor PTHrP/*PTHLH* expression and a higher rate of BC metastases to bone compared to non-osseous metastases in patients with stage 4 disease.

One study (3 cohorts, *n* = 111) examined the association between circulating PTHrP levels and preferential sites of metastases. Authors used mass spectrometry protein profiling and identified PTHrP(12–48) as a potential biomarker to segregate patients with bone metastases from patients with non-bone metastases (87). Subsequently, this finding was validated in two independent cohorts. The authors did not specify whether protease inhibitors were used at the time of blood collection.

### Association between PTHrP and hypercalcemia

Hypercalcemia of malignancy is a common metabolic complication of BC (89, 90). Considering that hypercalcemia is associated with worse cancer outcomes compared to normocalcemic patients (89), we examined this issue in our data set.

Thirteen studies, led by 9 research groups, met our eligibility criteria (Supplementary Tables 10 and 11) (23, 24, 25, 27, 28, 29, 91, 92, 93, 94, 95, 96, 97). This represents 219 tumors and 501 blood samples from patients recruited between 1984 and 1993. Twelve of these studies were at a very high risk of bias due to either confounding (no control for hyperparathyroidism, renal diseases and bone metastases) or because there was no detail about the methods used for blood collection (23, 24, 25, 27, 28, 29, 92, 93, 94, 95, 96, 97).

Four studies (*n* = 283) interrogated the frequency of hypercalcemia according to tumor PTHrP/*PTHLH* expression (23, 24, 25, 27). In 3/4 reports, hypercalcemia was more frequent in PTHrP-positive tumors than in PTHrP-negative tumors (23, 24, 25). However, the length of follow-up was not reported in 2/3 studies (23, 24), nor was it clear whether the duration of follow-up was similar in all patients (25). Thus, we cannot estimate the risk of hypercalcemia according to tumor PTHrP status from these data.

**Figure 6:**
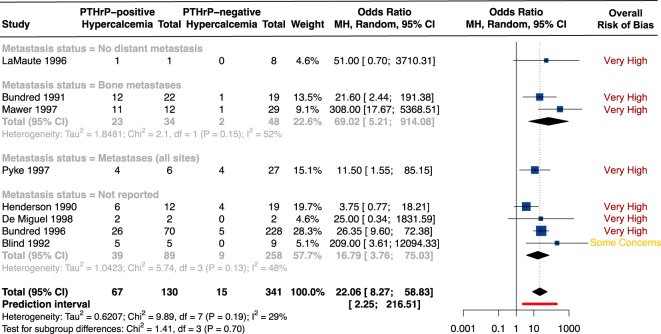
PTHrP/*PTHLH* and hypercalcemia of malignancy. Forest plot for univariate comparison of circulating PTHrP status with the diagnosis of hypercalcemia. Odds ratio <1 means that patients with hypercalcemia display more frequently PTHrP-negative tumors than normocalcemic patients, while odds ratio >1 means that patients with hypercalcemia display more frequently PTHrP-positive tumors than normocalcemic patients. Random-effects model *P*-value <0.0001. Of note, if the hypercalcemic patients displayed bone metastases, we have, when possible, restrained the normocalcemic control group to the patients with bone metastases as well. Abbreviations: MH, Mantel–Haenszel; 95% CI, 95% confidence interval; and df, degree of freedom. A full color version of this figure is available at https://doi.org/10.1530/ERC-25-0324.

In addition, 8/13 studies (*n* = 501) examined the potential of circulating PTHrP to be used as a diagnostic tool to identify patients with hypercalcemia of malignancy in BC patients (24, 28, 29, 91, 92, 93, 94, 95). Circulating PTHrP was found more frequently in BC patients with hypercalcemia of malignancy than in normocalcemic patients (Fig. 6). The meta-analysis shows moderate variability due to between-study heterogeneity. However, due to the cross-sectional design of these studies, no conclusion about the predictive value or the causal role of tumor PTHrP secretion can be drawn from these data.

Finally, only 2/13 studies examined the relationship between PTHrP and humoral *versus* osteolytic hypercalcemia (96, 97). Methodologies and study populations were not comparable, preventing any solid conclusion. Overall, the data support that circulating PTHrP is associated with the frequency and diagnosis of hypercalcemia of malignancy in BC patients.

## Discussion

This systematic review found no clear association between tumor PTHrP expression and patients’ menopausal or ER status but suggests a possible association with PR status. Experimental work on the relationship between sex hormone receptors and PTHrP expression has also been inconsistent. In some studies, PTHrP expression was decreased by estradiol-induced activation of endogenous or exogenously introduced ERα in KPL-3C and MDA-MB-231 cells, respectively (98, 99). Conversely, other investigations using ERα-positive BC cell lines and cell line-derived bone metastases reported that a single dose of estradiol increased PTHrP expression and tumor-associated osteolysis, whereas estrone had no effect (100, 101). Although these findings appear discordant, they may reflect context-dependent differences in ER signaling rather than a uniform regulatory mechanism. In clinical practice, ER status is determined by nuclear ERα immunostaining, which does not account for variations in ligand exposure, signaling dynamics or downstream pathway activation that may influence PTHrP expression. As a result, the available evidence remains insufficient to draw definitive conclusions, and further clinically relevant studies are required to clarify the relationship between PTHrP expression and hormone receptor status in BC.

We also found that PTHrP expression correlates with lymph node invasion and the development of bone metastases. *Pthlh* disruption in mammary epithelial cells of MMTV-PyMT mice delays tumor development and progression (19), while PTHrP overexpression accelerates tumor development and increases tumor burden (21). In addition, PTHrP overexpression in MDA-MB-231 cells increased osteolytic bone metastases in nude mice (102). These results support the functional role of PTHrP in both primary tumor progression and bone metastasis burden. Furthermore, in the luminal A, MCF-7 cell line, one study reported that full-length PTHrP overexpression did not alter tumorigenesis (22), while others showed that intracrine functions of PTHrP promotes mitogenesis and invasion (103, 104, 105). Conversely, Tran *et al.* showed that nuclear PTHrP was associated with a better outcome in all BC patients (40). This analysis was, however, at a very high risk of bias due to confounding. The study populations included in this systematic review were composed of all BC molecular subtypes, and no subgroup analyses according to ER/PR status were feasible. Further prospective studies and experimental research must be performed to examine the functions of PTHrP in BC progression according to molecular subtypes.

Finally, the results of the meta-analyses suggest an association between tumor PTHrP detection and the frequency of hypercalcemia events in BC patients. Similarly, circulating PTHrP was more often detectable in patients with hypercalcemia compared to normocalcemic patients. These are consistent with experimental evidence demonstrating that PTHrP production by mammary tumors can cause hypercalcemia (106, 107). However, whether PTHrP expression in tumor tissue at the time of diagnosis predicts future risk of hypercalcemia is not clear.

Although the employed methods were highly heterogeneous, the collective results summarized here suggest an association between tumor PTHrP expression and PR-positivity, the presence of lymph node invasion and bone metastases. This underscores the importance of investigating the molecular mechanisms of PTHrP, particularly its role in facilitating tumor cell migration, within the context of modern molecular subtyping. Nevertheless, accurate PTHrP measurement in tumors and blood remains challenging, hindering research. Indeed, PTHrP expression is heterogeneous, with our group finding only 1.5–9% of tumor cells *PTHLH*-positive by single-cell RNA sequencing (21), highlighting the impact of biopsy location and method on quantitation. In addition, immunohistochemistry is limited by antibody specificity, while mRNA-based techniques may not reflect protein abundance. Furthermore, circulating PTHrP is subject to rapid clearance and short half-life (83). Therefore, single blood measurements may not accurately capture systemic exposure and tumor secretion. Future advances in assay development, such as mass spectrometry-based methods (87), and the development of highly specific antibodies are needed to improve assay sensitivity and reproducibility for robustly evaluating PTHrP as a prognostic marker. This is clinically important because the *PTHLH* gene has been repeatedly identified as a potential BC susceptibility gene in GWASs (43, 44, 45, 46, 47, 48, 49). Hopefully, the results of this systematic review and meta-analysis can guide the design of future studies to better understand how PTHrP influences BC development and behavior in patients.

Despite the limitations inherent in literature reviews, such as potential misinterpretation or omission of studies, this analysis is the first systematic evaluation of 35 years of observational research on PTHrP’s prognostic value in BC patients.

## Supplementary materials







































## Declaration of interest

The authors declare that there is no conflict of interest that could be perceived as prejudicing the impartiality of the work reported.

## Funding

A Clemenceau holds a Leslie H Warner postdoctoral fellowship for cancer research from the Yale Cancer Center. J Bherer holds a doctoral research award from the Cancer Research Society and a doctoral training scholarship from the Fonds de Recherche du Québec – Health.

## Author contribution statement

A Clemenceau conceived the study; curated the data; performed formal analysis, investigation and visualization; designed the methodology; and wrote, reviewed and edited the original draft of the manuscript. J Bherer curated the data; performed investigation, validation and visualization; designed the methodology; and wrote, reviewed and edited the original draft of the manuscript. A Grimshaw conceived the study, curated the data, designed the methodology, acquired resources and software and wrote, reviewed and edited the original draft of the manuscript. F Durocher acquired resources and wrote, reviewed and edited the original draft of the manuscript. C Diorio conceived the study, curated the data, designed the methodology, supervised the study, performed validation and wrote, reviewed and edited the original draft of the manuscript. JJ Wysolmerski conceived the study, acquired funding, supervised the study, perfumed validation and wrote, reviewed and edited the original draft of the manuscript.

## Data sharing/availability statement

The data generated in this study are available within the article and its supplementary data files.
