# Supplementary material for: Clinical and prognostic significance of parathyroid hormone-related protein in breast cancer: a systematic review and meta-analyses of observational studies in women
Source: Endocr Relat Cancer. 2026 Mar 5;33(3):e250324. doi: 10.1530/ERC-25-0324 (PMC12978662; doi:10.1530/ERC-25-0324)
Supplement: Supplementary file 1 [file supplementary_figure_1.pdf]

Risk of bias domains

Study	D1	D2	D3	D4	D5	D6	D7	Overall
Southby 1990	+	+	+	+	+	+	-	-
Bundred 1992	+	+	+	+	+	+	+	+
Bouizar 1993	+	+	-	+	+	+	+	-
Fraser 1993	+	+	+	+	+	+	+	+
Kissin 1993	+	+	-	+	+	+	-	-
Liapis 1993	+	-	-	+	X	+	+	X
Kitazawa 1994	+	-	-	+	+	+	-	-
Kohno 1994	+	+	-	+	+	+	+	-
Edwards 1995	+	-	-	+	!	+	-	!
Bundred 1996	+	-	-	+	X	-	X	X
Downey 1997	+	X	+	+	+	+	+	X
Mawer 1997	+	!	-	+	X	-	+	!
Wulf 1997 (tumor)	+	+	+	+	X	+	+	X
Wulf 1997 (blood)	+	+	+	+	X	+	+	X
Bucht 1998 (tumor)	+	+	+	+	X	+	+	X
Bucht 1998 (blood)	+	-	+	+	X	+	+	X
Iezzoni 1998	+	+	+	+	-	X	+	X
Bouizar 1999	+	+	+	+	X	+	X	X
Sugimoto 1999	+	+	+	+	+	+	+	+
Yoshida 2000	+	+	+	+	+	+	+	+
Henderson 2006	+	-	+	+	+	+	+	-
Zia 2007	+	+	+	+	+	+	+	+
Fleming 2009	+	-	+	+	+	+	+	-
Takagaki 2012	+	X	+	+	+	+	+	X
Skondra 2014	+	!	-	+	+	-	-	!
Xu 2015	+	+	+	+	+	+	+	+
Tran 2018	+	+	+	+	-	+	+	-
Assaker 2020	+	-	+	+	+	+	+	-
Grinman 2022	+	+	+	+	+	+	+	+
Shalaby 2025	+	+	+	+	+	+	X	X

Domains:
D1: Bias due to confounding.
D2: Bias arising from measurement of the exposure.
D3: Bias in selection of participants into the study (or into the analysis).
D4: Bias due to post-exposure interventions.
D5: Bias due to missing data.
D6: Bias arising from measurement of the outcome.
D7: Bias in selection of the reported result.

Judgement:
+ Low risk
- Some concerns
X High risk
! Very high risk
