# Supplementary figures and images for: Clinical and prognostic significance of parathyroid hormone-related protein in breast cancer: a systematic review and meta-analyses of observational studies in women
Source: Endocr Relat Cancer. 2026 Mar 5;33(3):e250324. doi: 10.1530/ERC-25-0324 (PMC12978662; doi:10.1530/ERC-25-0324)

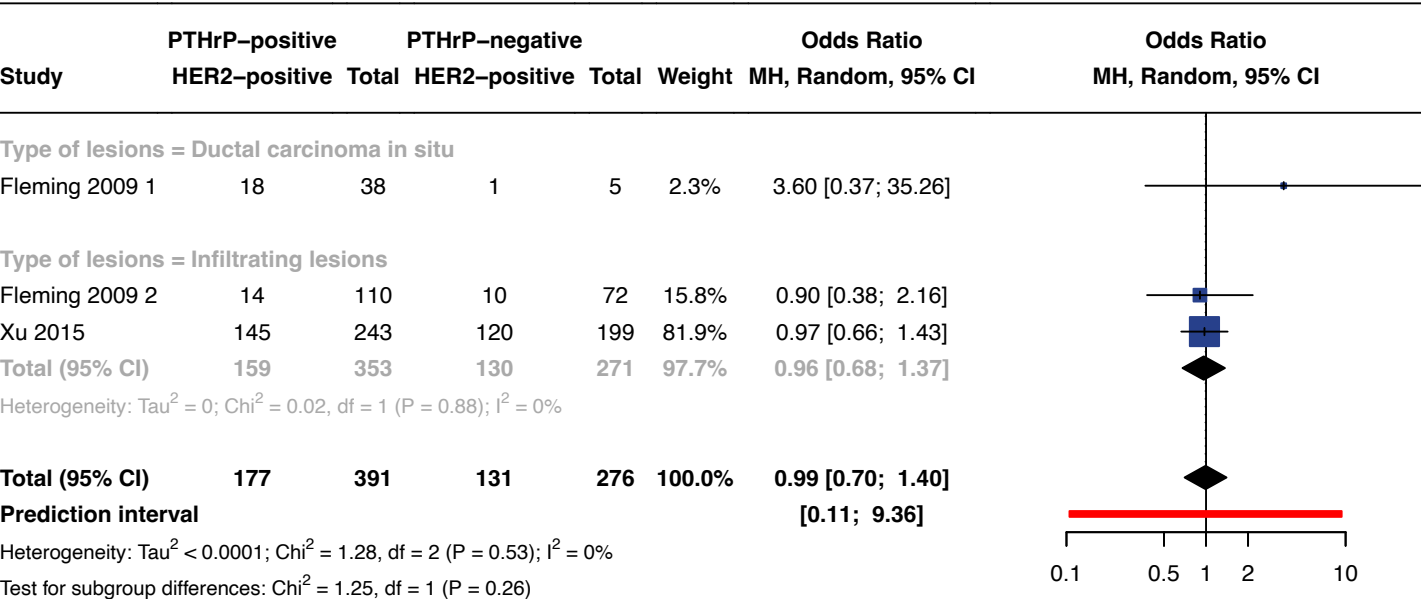

Supplement: Supplementary file 2 [file supplementary_figure_2.pdf]

**A** Association between tumor PTHrP/*PTHLH* and tumor grade

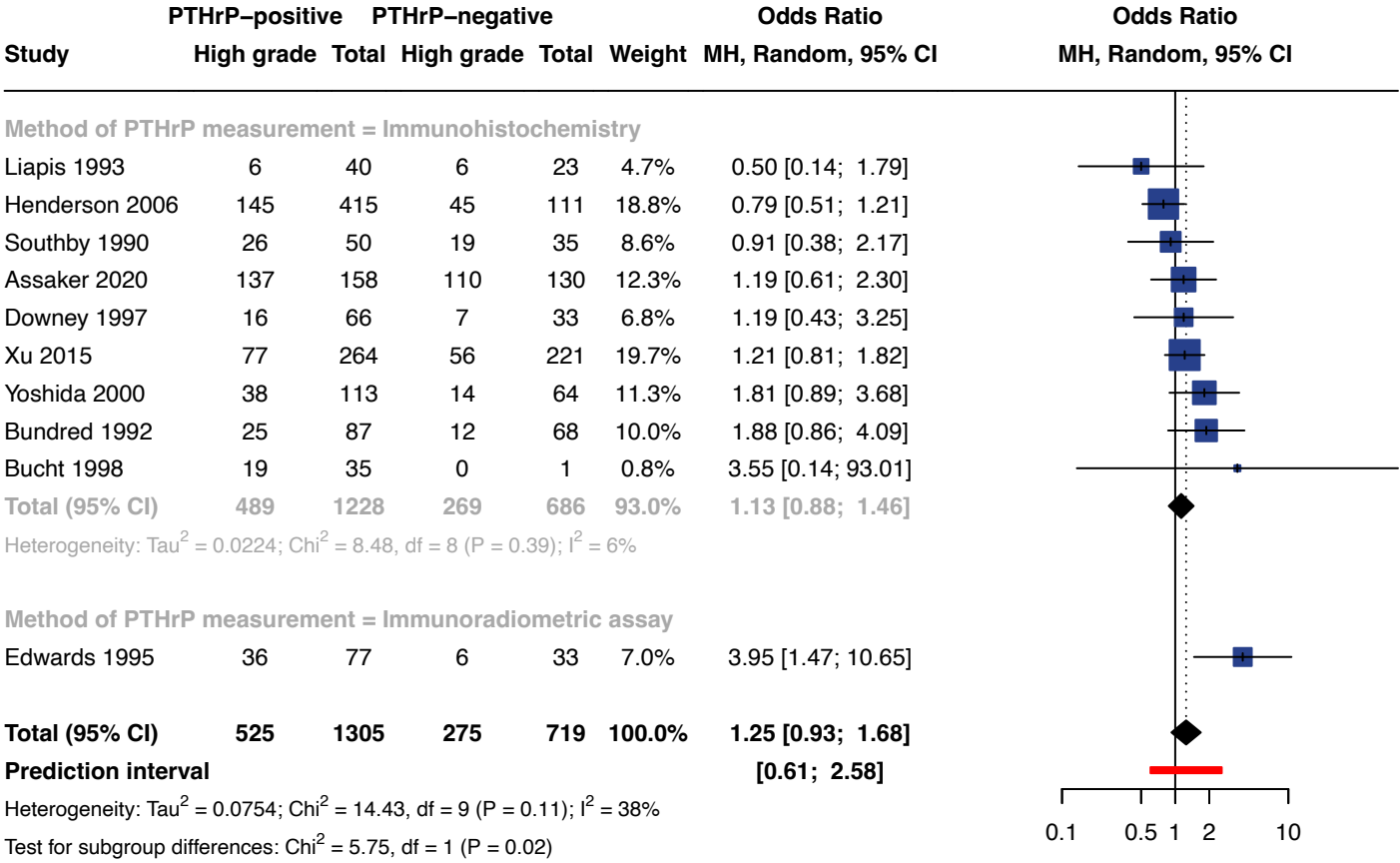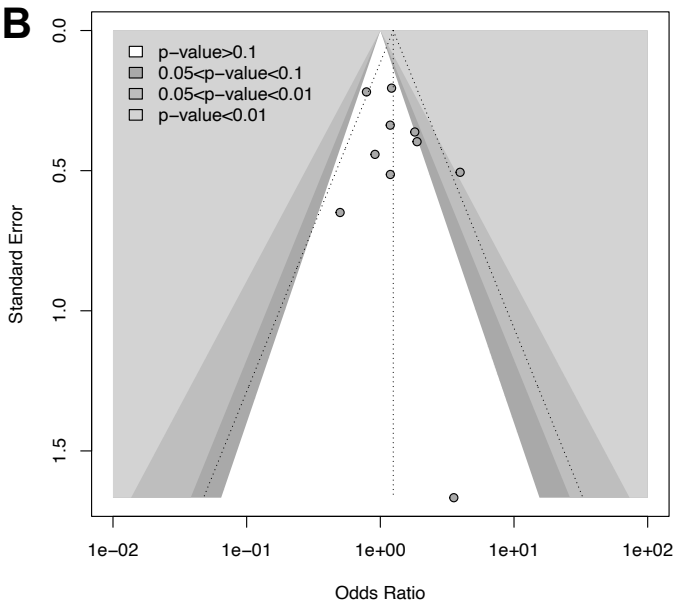

Supplement: Supplementary file 4 [file supplementary_figure_4.pdf]

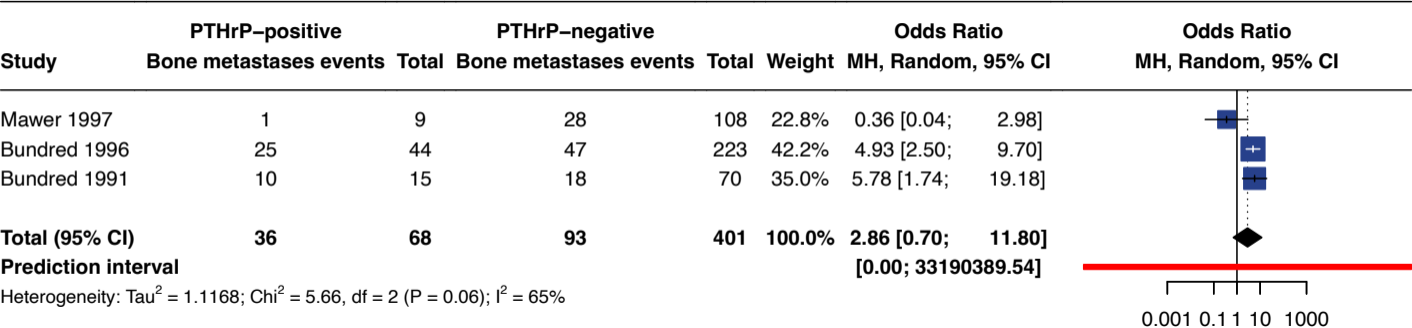

Supplement: Supplementary file 7 [file supplementary_figure_7.pdf]
