# Supplementary material for: Clinical and prognostic significance of parathyroid hormone-related protein in breast cancer: a systematic review and meta-analyses of observational studies in women
Source: Endocr Relat Cancer. 2026 Mar 5;33(3):e250324. doi: 10.1530/ERC-25-0324 (PMC12978662; doi:10.1530/ERC-25-0324)

A Association between tumor PTHrP/*PTHLH* and tumor stage

Study	PTHrP−positive Stage 3−4	PTHrP−positive Total	PTHrP−negative Stage 3−4	PTHrP−negative Total	Weight	Odds Ratio MH, Random, 95% CI
Henderson 2006	80	415	31	111	37.3%	0.62 [0.38; 1.00]
Southby 1990	14	54	12	37	25.1%	0.73 [0.29; 1.83]
Xu 2015	60	271	32	226	37.6%	1.72 [1.08; 2.76]
**Total (95% CI)**	**154**	**740**	**75**	**374**	**100.0%**	**0.95 [0.48; 1.88]**
**Prediction interval**						**[0.00; 2698.91]**

Heterogeneity: $Tau^2 = 0.2692$; $Chi^2 = 9.47$, df = 2 ($P < 0.01$); $I^2 = 79\%$

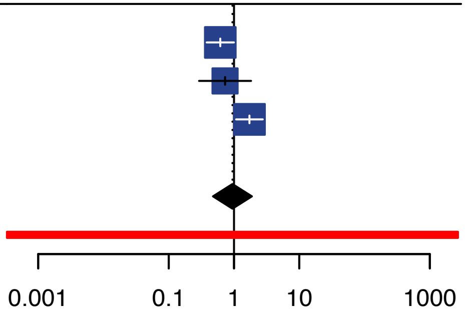

B Association between tumor PTHrP/*PTHLH* and tumor size

Study	PTHrP−positive Tumor > 2cm	PTHrP−positive Total	PTHrP−negative Tumor > 2cm	PTHrP−negative Total	Weight	Odds Ratio MH, Random, 95% CI
Henderson 2006	200	415	68	111	26.7%	0.59 [0.38; 0.90]
Assaker 2020	80	160	51	130	25.9%	1.55 [0.97; 2.48]
Xu 2015	138	271	81	226	27.8%	1.86 [1.29; 2.67]
Takagaki 2012	62	79	29	46	19.6%	2.14 [0.96; 4.78]
**Total (95% CI)**	**480**	**925**	**229**	**513**	**100.0%**	**1.34 [0.75; 2.40]**
**Prediction interval**						**[0.10; 18.64]**

Heterogeneity: $Tau^2 = 0.2854$; $Chi^2 = 18.96$, df = 3 ($P < 0.01$); $I^2 = 84\%$

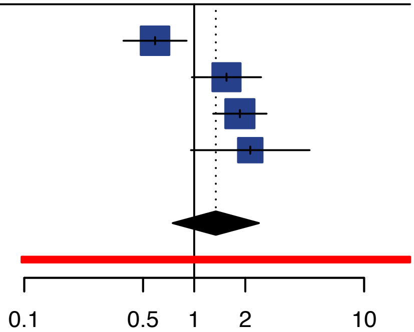

C Association between tumor PTHrP/*PTHLH* and metastasis status

Study	PTHrP−positive Distant metastasis	PTHrP−positive Total	PTHrP−negative Distant metastasis	PTHrP−negative Total	Weight	Odds Ratio MH, Random, 95% CI
Yoshida 2000	5	113	1	64	63.6%	2.92 [0.33; 25.53]
Takagaki 2012	9	79	0	46	36.4%	12.53 [0.71; 220.54]
**Total (95% CI)**	**14**	**192**	**1**	**110**	**100.0%**	**4.96 [0.88; 27.97]**

Heterogeneity: $Tau^2 = 0$; $Chi^2 = 0.63$, df = 1 ($P = 0.43$); $I^2 = 0\%$

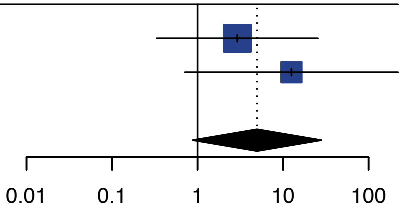

Supplement: Supplementary file 3 [file supplementary_figure_3.pdf]
