# Supplementary material for: Clinical and prognostic significance of parathyroid hormone-related protein in breast cancer: a systematic review and meta-analyses of observational studies in women
Source: Endocr Relat Cancer. 2026 Mar 5;33(3):e250324. doi: 10.1530/ERC-25-0324 (PMC12978662; doi:10.1530/ERC-25-0324)

A Association between tumor PTHrP/*PTHLH* and tumor histologic subtype

Study	PTHrP−positive Ductal carcinoma	Total	PTHrP−negative Ductal carcinoma	Total	Weight	Odds Ratio MH, Random, 95% CI
Liapis 1993	53	56	25	25	1.1%	0.30 [0.01; 6.02]
Henderson 2006	361	414	97	110	24.1%	0.91 [0.48; 1.74]
Xu 2015	231	271	192	226	41.1%	1.02 [0.62; 1.68]
Southby 1990	44	54	30	37	8.8%	1.03 [0.35; 3.00]
Edwards 1995	71	82	30	36	8.6%	1.29 [0.44; 3.81]
Bundred 1992	73	87	53	68	15.4%	1.48 [0.66; 3.32]
Bucht 1998	30	40	0	1	0.9%	8.71 [0.33; 230.79]
**Total (95% CI)**	**863**	**1004**	**427**	**503**	**100.0%**	**1.08 [0.79; 1.49]**
**Prediction interval**						**[0.71; 1.64]**

Heterogeneity: $Tau^2 < 0.0001$; $Chi^2 = 3.25$, df = 6 (P = 0.78); $I^2 = 0\%$

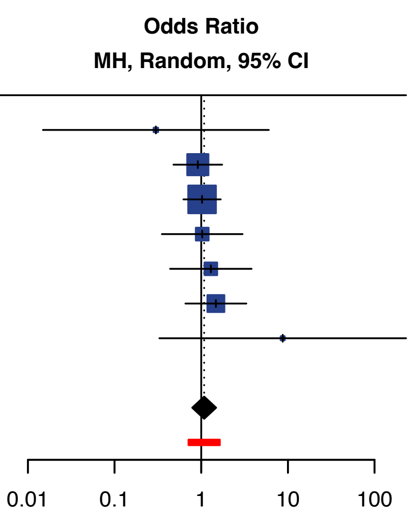

B Association between tumor PTHrP/*PTHLH* and the presence of histologic microcalcifications

Study	PTHrP−positive Microcalcifications	Total	PTHrP−negative Microcalcifications	Total	Weight	Odds Ratio MH, Random, 95% CI
Southby 1990	31	54	16	37	28.5%	1.77 [0.76; 4.12]
Bundred 1996	25	110	8	75	28.3%	2.46 [1.04; 5.81]
Liapis 1993	24	55	3	25	21.2%	5.68 [1.52; 21.23]
Kissin 1993	24	30	6	29	22.0%	15.33 [4.32; 54.49]
**Total (95% CI)**	**104**	**249**	**33**	**166**	**100.0%**	**4.00 [1.59; 10.07]**
**Prediction interval**						**[0.08; 194.34]**

Heterogeneity: $Tau^2 = 0.5928$; $Chi^2 = 8.83$, df = 3 (P = 0.03); $I^2 = 66\%$

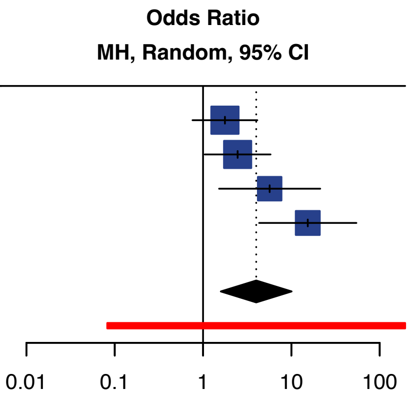

C Association between tumor PTHrP/*PTHLH* and lympho-vascular invasion

Study	PTHrP−positive Invaded	Total	PTHrP−negative Invaded	Total	Weight	Odds Ratio MH, Random, 95% CI
Outcome = Lympho−vascular infiltration						
Assaker 2020	57	160	43	130	35.7%	1.12 [0.69; 1.82]
Henderson 2006	275	411	70	109	43.5%	1.13 [0.72; 1.75]
**Total (95% CI)**	**332**	**571**	**113**	**239**	**79.2%**	**1.12 [0.81; 1.56]**
Heterogeneity: $Tau^2 = 0$; $Chi^2 = 0$, df = 1 (P = 0.99); $I^2 = 0\%$						
Outcome = Lymphatic infiltration						
Takagaki 2012 1	42	77	19	43	15.1%	1.52 [0.72; 3.21]
Outcome = Vascular infiltration						
Takagaki 2012 2	10	77	4	43	5.7%	1.46 [0.43; 4.95]
**Total (95% CI)**	**384**	**725**	**136**	**325**	**100.0%**	**1.19 [0.89; 1.60]**
**Prediction interval**						**[0.63; 2.26]**

Heterogeneity: $Tau^2 = 0$; $Chi^2 = 0.62$, df = 3 (P = 0.89); $I^2 = 0\%$

Test for subgroup differences: $Chi^2 = 0.62$, df = 2 (P = 0.73)

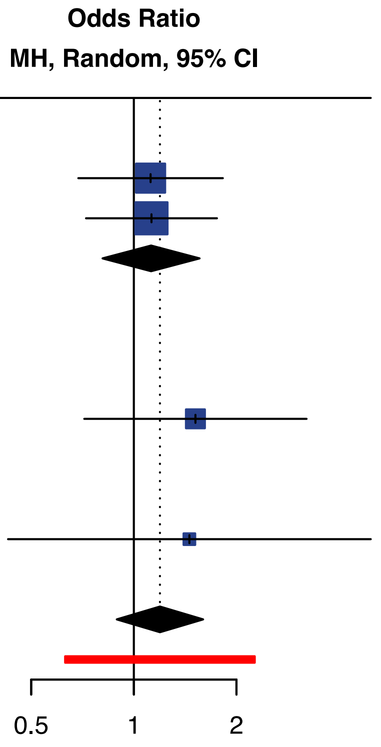

Supplement: Supplementary file 5 [file supplementary_figure_5.pdf]
