# Supplementary material for: Clinical and prognostic significance of parathyroid hormone-related protein in breast cancer: a systematic review and meta-analyses of observational studies in women
Source: Endocr Relat Cancer. 2026 Mar 5;33(3):e250324. doi: 10.1530/ERC-25-0324 (PMC12978662; doi:10.1530/ERC-25-0324)
Supplement: Supplementary file 6 [file supplementary_figure_6.pdf]

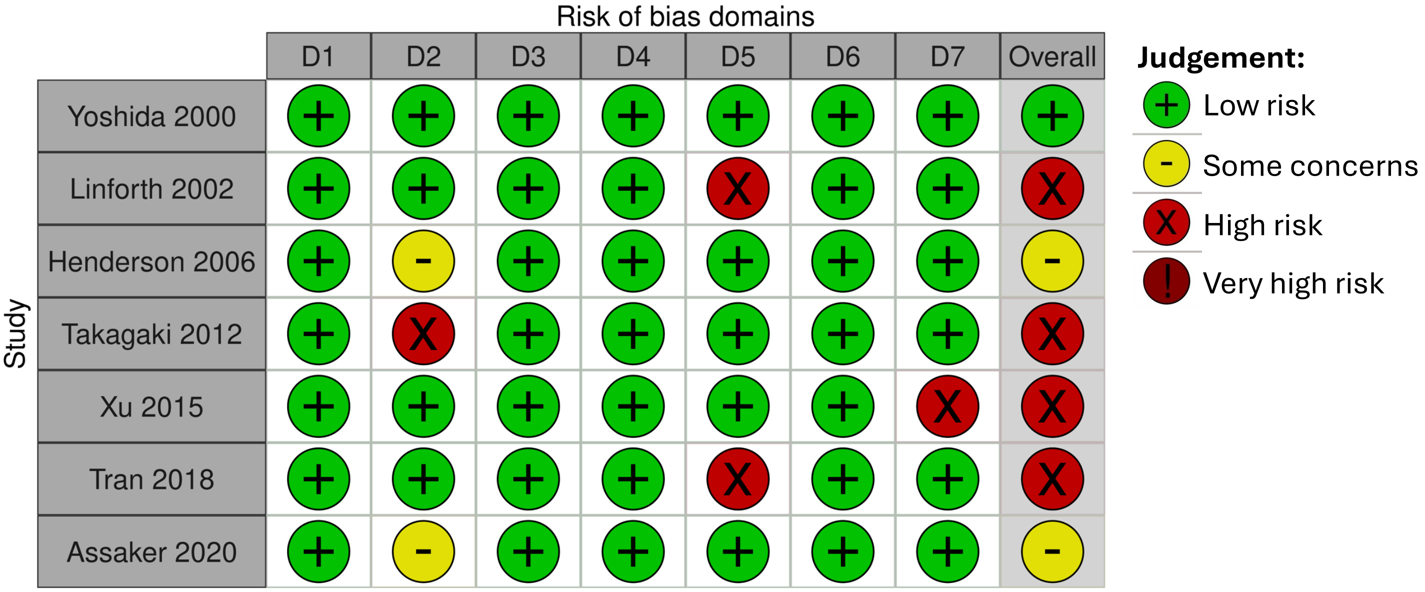


**Domains:**

**D1:** Bias due to confounding.

**D2:** Bias arising from measurement of the exposure.

**D3:** Bias in selection of participants into the study (or into the analysis).

**D4:** Bias due to post-exposure interventions.

**D5:** Bias due to missing data.

**D6:** Bias arising from measurement of the outcome.

**D7:** Bias in selection of the reported result.
