# Supplementary material for: Clinical and prognostic significance of parathyroid hormone-related protein in breast cancer: a systematic review and meta-analyses of observational studies in women
Source: Endocr Relat Cancer. 2026 Mar 5;33(3):e250324. doi: 10.1530/ERC-25-0324 (PMC12978662; doi:10.1530/ERC-25-0324)
Supplement: Supplementary file 8 [file supplementary_figure_8.pdf]

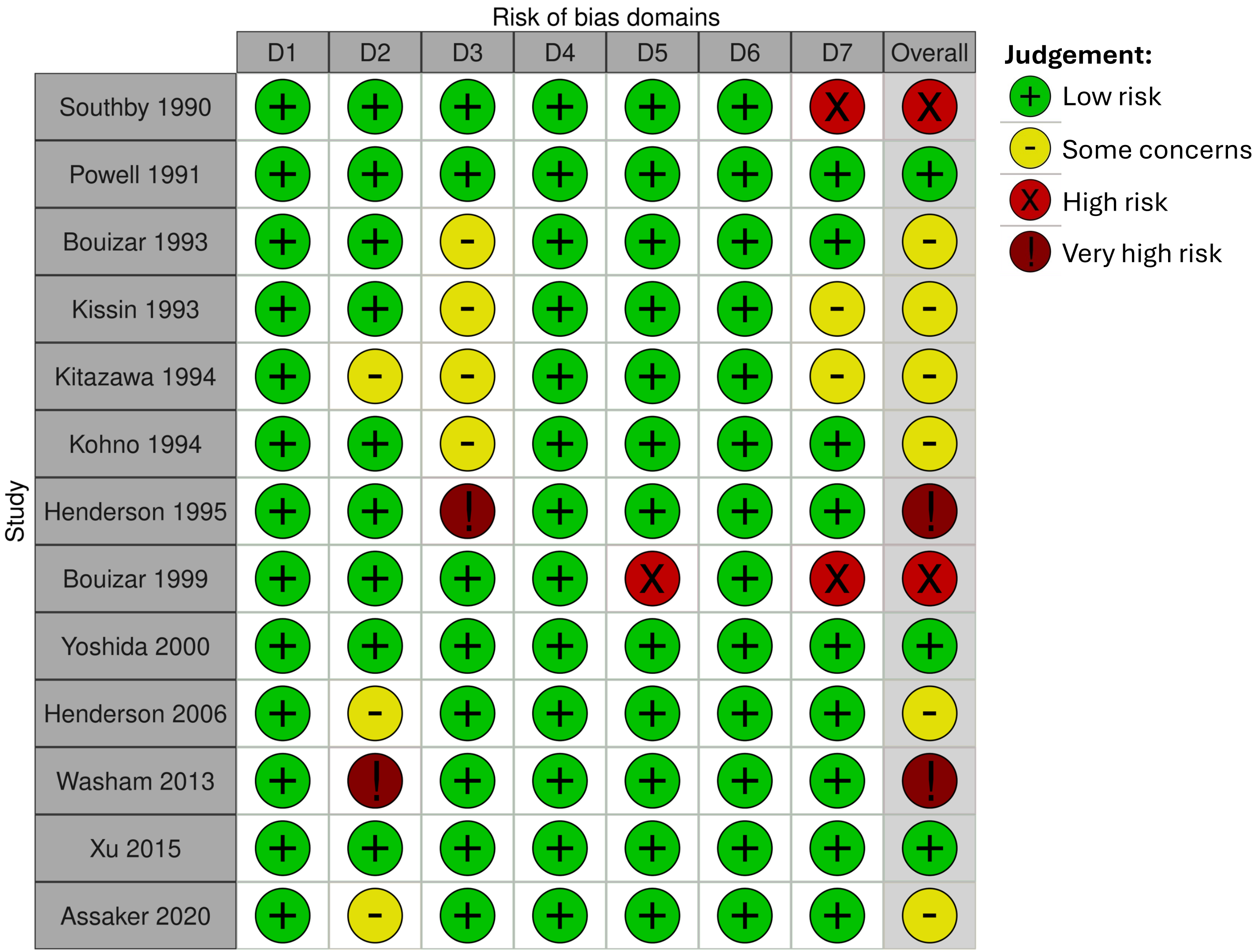

Domains:

D1: Bias due to confounding.

D2: Bias arising from measurement of the exposure.

D3: Bias in selection of participants into the study (or into the analysis)

D4: Bias due to post-exposure interventions.

D5: Bias due to missing data.

D6: Bias arising from measurement of the outcome.

D7: Bias in selection of the reported result.
