# Supplementary material for: Clinical and prognostic significance of parathyroid hormone-related protein in breast cancer: a systematic review and meta-analyses of observational studies in women
Source: Endocr Relat Cancer. 2026 Mar 5;33(3):e250324. doi: 10.1530/ERC-25-0324 (PMC12978662; doi:10.1530/ERC-25-0324)
Supplement: Supplementary file 10 [file supplementary_table_2.pdf]

Supplementary Table 2: Search Strategies

Ovid Embase

1 exp breast cancer/
2 ((breast* or mamma* or invasive ductal or invasive lobular) adj3 (neoplasm* or tumor* or tumour* or cyst* or cancer* or carcinogenes* or carcinoma* or adenocarcinoma* or malignan* or neoplasia* or lesion* or sarcoma* or angiosarcoma*)).tw,kf.
3 (DCIS or ductal-carcinoma-in-situ).tw,kf.
4 or/1-3
5 parathyroid hormone related protein/
6 ((parathyroid-hormone* or PTH) adj3 (peptide* or protein* or factor*)).tw,kf.
7 (parathyroid gland hormone* or parathyroid hormone like hormone* or tumor hypercalcemic factor*).tw,kf.
8 (PTHrP or PTH-rP or PTHLH or HHM or PLP or BDE2 or PTHR).ti,ab.
9 5 or 6 or 7 or 8
10 4 and 9 880

Ovid MEDLINE(R) ALL

1 exp Breast Neoplasms/
2 ((breast* or mamma* or invasive ductal or invasive lobular) adj3 (neoplasm* or tumor* or tumour* or cyst* or cancer* or carcinogenes* or carcinoma* or adenocarcinoma* or malignan* or neoplasia* or lesion* or sarcoma* or angiosarcoma*)).tw,kf.
3 (DCIS or ductal-carcinoma-in-situ).tw,kf.
4 or/1-3
5 Parathyroid Hormone-Related Protein/
6 ((parathyroid-hormone* or PTH) adj3 (peptide* or protein* or factor*)).tw,kf.
7 (parathyroid gland hormone* or parathyroid hormone like hormone* or tumor hypercalcemic factor*).tw,kf.
8 (PTHrP or PTH-rP or PTHLH or HHM or PLP or BDE2 or PTHR).ti,ab. 10383
9 5 or 6 or 7 or 8 13223
10 4 and 9

Scopus

(TITLE-ABS-KEY ((breast* OR mamma* OR "invasive ductal" OR "invasive lobular") W/3 (neoplasm* OR tumor* OR tumour* OR cyst* OR cancer* OR carcinogenes* OR carcinoma* OR adenocarcinoma* OR malignan* OR neoplasia* OR lesion* OR sarcoma* OR angiosarcoma*)) OR TITLE-ABS-KEY (dcis OR ductal-carcinoma-in-situ)) AND (TITLE-ABS-KEY ((parathyroid-hormone* OR pth) W/3 (peptide* OR protein* OR factor*)) OR TITLE-ABS-KEY ("parathyroid gland hormone*" OR "parathyroid hormone like hormone*" OR "tumor hypercalcemic factor*") OR TITLE-ABS-KEY (pthrp OR pth-rp OR pthlh OR hhm OR plp OR bde2 OR pthr))

Web of Science Core Collection

#1 TS=((breast* or mamma* or "invasive ductal" or "invasive lobular") near/3 (neoplasm* or tumor* or tumour* or cyst* or cancer* or carcinogenes* or carcinoma* or adenocarcinoma* or malignan* or neoplasia* or lesion* or sarcoma* or angiosarcoma*)) or TS=(DCIS or ductal-carcinoma-in-situ)

#2 TS=((parathyroid-hormone* or PTH) near/3 (peptide* or protein* or factor*)) or TS=("parathyroid gland hormone*" or "parathyroid hormone like hormone*" or "tumor hypercalcemic factor*") or TS=(PTHrP or PTH-rP or PTHLH or HHM or PLP or BDE2 or PTHR)
#3 #1 AND #2

The Core Collection included in this review is:
1. Science Citation Index Expanded (1900 - Data Searched)
2. Social Sciences Citation Index (1900 - Date Searched)
3. Art & Humanities - (1975 - Date Searched)
4. Conference Proceedings Citation Index - Science (1991 - Date Searched)
5. Conference Proceedings Citation Index - Social Sciences and Humanities (1991 - Date Searched)
6. Book Citation Index - Science (2005 - Date Searched)
7. Book Citation Index - Social Sciences and Humanities (2005 - Date Searched)
8. Emerging Source Citation Index - (2018 - Date Searched)
9. Current Chemical Reactions (1985 - Date Searched)
10. Index Chemicus (1993 - Date Searched)

PubMed

(breast cancer*[Title/Abstract] OR breast neoplasm*[Title/Abstract] OR breast tumor*[Title/Abstract] OR breast tumour*[Title/Abstract] OR breast cyst*[Title/Abstract] OR breast carcinogenes*[Title/Abstract] OR breast carcinoma*[Title/Abstract] OR breast adenocarcinoma*[Title/Abstract] OR breast maligenan*[Title/Abstract] OR breast neoplasia*[Title/Abstract] OR breast lesion*[Title/Abstract] OR breast sarcoma*[Title/Abstract] OR breast angiosarcoma*[Title/Abstract] OR mamma* cancer*[Title/Abstract] OR mamma* neoplasm*[Title/Abstract] OR mamma* tumor*[Title/Abstract] OR mamma* tumour*[Title/Abstract] OR mamma* cyst*[Title/Abstract] OR mamma* carcinogenes*[Title/Abstract] OR mamma* carcinoma*[Title/Abstract] OR mamma* adenocarcinoma*[Title/Abstract] OR mamma* maligenan*[Title/Abstract] OR mamma* neoplasia*[Title/Abstract] OR mamma* lesion*[Title/Abstract] OR mamma* sarcoma*[Title/Abstract] OR mamma* angiosarcoma*[Title/Abstract] OR invasive ductal cancer*[Title/Abstract] OR invasive ductal neoplasm*[Title/Abstract] OR invasive ductal tumor*[Title/Abstract] OR invasive ductal tumour*[Title/Abstract] OR invasive ductal cyst*[Title/Abstract] OR invasive ductal carcinogenes*[Title/Abstract] OR invasive ductal carcinoma*[Title/Abstract] OR invasive ductal adenocarcinoma*[Title/Abstract] OR invasive ductal maligenan*[Title/Abstract] OR invasive ductal neoplasia*[Title/Abstract] OR invasive ductal lesion*[Title/Abstract] OR invasive ductal sarcoma*[Title/Abstract] OR invasive ductal angiosarcoma[Title/Abstract] OR invasive lobular cancer*[Title/Abstract] OR invasive lobular neoplasm*[Title/Abstract] OR mamma* tumor*[Title/Abstract] OR invasive lobular tumour*[Title/Abstract] OR invasive lobular cyst*[Title/Abstract] OR invasive lobular carcinogenes*[Title/Abstract] OR invasive lobular carcinoma*[Title/Abstract] OR invasive lobular adenocarcinoma*[Title/Abstract] OR invasive lobular maligenan*[Title/Abstract] OR invasive lobular neoplasia*[Title/Abstract] OR invasive lobular lesion*[Title/Abstract] OR invasive lobular sarcoma*[Title/Abstract] OR invasive lobular angiosarcoma*[Title/Abstract]) AND ("parathyroid gland hormone*"[Title/Abstract] OR "parathyroid hormone like hormone*"[Title/Abstract] OR "tumor hypercalcemic factor*"[Title/Abstract] OR pthrp[Title/Abstract] OR pth-rp[Title/Abstract] OR pthlh[Title/Abstract] OR hhm[Title/Abstract] OR plp[Title/Abstract] OR bde2[Title/Abstract] OR pthr[Title/Abstract] OR parathyroid hormone*[Title/Abstract])

Cochrane Library

#1 ((breast* or mamma* or "invasive ductal" or "invasive lobular") near/3 (neoplasm* or tumor* or tumour* or cyst* or cancer* or carcinogenes* or carcinoma* or adenocarcinoma* or malignan* or neoplasia* or lesion* or sarcoma* or angiosarcoma*)):ti,ab or (DCIS or ductal-carcinoma-in-situ):ti,ab

#2 ((parathyroid-hormone* or PTH) near/3 (peptide* or protein* or factor*)):ti,ab or (parathyroid gland hormone* or parathyroid hormone like hormone* or tumor hypercalcemic factor*):ti,ab or (PTHrP or PTH-rP or PTHLH or HHM or PLP or BDE2 or PTHR):ti,ab
#3 #1 and #2

The Cochrane Library database includes:
1. Cochrane Database of Systematic Reviews
2. Cochrane Central Register of Controlled Trials
3. Cochrane Clinical Answers

Google Scholar

Breast cancer parathyroid hormone related protein
