# Supplementary material for: Clinical and prognostic significance of parathyroid hormone-related protein in breast cancer: a systematic review and meta-analyses of observational studies in women
Source: Endocr Relat Cancer. 2026 Mar 5;33(3):e250324. doi: 10.1530/ERC-25-0324 (PMC12978662; doi:10.1530/ERC-25-0324)
Supplement: Supplementary file 11 [file supplementary_table_3.pdf]

Supplementary Table 3: Excluded Studies Table

Studies Excluded from the Search Results				
**First Authors Last Name**	**Year**	**Title**	**Journal**	**Reason for Exclusion**
Antoniou	2012	Common variants at 12p11, 12q24, 9p21, 9q31.2 and in ZNF365 are associated with breast cancer risk for BRCA1 and/or BRCA2 mutation carriers	Breast cancer research: BCR	Did not assess PTHrP/PTHLH expression
Antoniou	2012	Common variants at 12p11, 12q24, 9p21, 9q31.2 and in ZNF365 are associated with breast cancer risk for BRCA1 and/or BRCA2 mutation carriers	Breast cancer research: BCR	Did not assess PTHrP/PTHLH expression
Antoniou	2012	Common variants at 12p11, 12q24, 9p21, 9q31.2 and in <i>ZNF365</i> are associated with breast cancer risk for <i>BRCA1</i> and/or <i>BRCA2</i> mutation carriers	Breast cancer research: BCR	Did not assess PTHrP/PTHLH expression
Assaker	2018	In silico gene expression analysis of PTHrP and its association with molecular subtypes and organ-specific metastasis in human triple-negative breast cancer	Cancer Research.	Conference paper
Audran	1995	CONTRIBUTION OF PARATHYROID HORMONE-RELATED PEPTIDE TO THE EVALUATION OF HYPERCALCEMIA	Rev. Rhum.	Wrong population
Ayguz	2018	Investigation of polymorphisms involved in turkish breast cancer patients	Human Heredity	Conference paper
Basuyau	1998	Value of PTH-rP as diagnostic aid and prognostic factor in hypercalcemia during the course of cancers	Immuno-Anal. Biol. Spec.	Not in English
Berruti	1996	Serum and urinary profile of bone turn-over markers in a patient with tamoxifen-induced hypercalcaemia submitted to pamidronate therapy	Breast	Case report
Body	2000	Decreased efficacy of bisphosphonates for recurrences of tumor-induced hypercalcemia	Support Care Cancer	Wrong outcomes
Bouizar	1995	PARATHYROID-HORMONE RELATED PROTEIN (PTH-RP) DIFFERENT SPLICING FORMS IN BREAST-CANCER AND THE OCCURRENCE OF METASTASIS TO BONE	J. Bone Miner. Res.	Conference paper
Budayr	1994	Effects of treatment of malignancyâ€• associated hypercalcemia on serum parathyroid hormoneâ€• related protein	J. Bone Miner. Res.	Wrong outcomes
Budayr	1989	Increased serum levels of a parathyroid hormone-like protein in malignancy-associated hypercalcemia	Ann Intern Med	Wrong population
Bundred	1993	Parathyroid hormone-related protein: cytosol extraction in breast cancer and normal breast tissue	â€¦ Breast	Conference paper

Burtis	1990	Immunochemical characterization of circulating parathyroid hormone-related protein in patients with humoral hypercalcemia of cancer	N Engl J Med	Wrong population
Chen	2022	Genome-wide and transcriptome-wide association studies of mammographic density phenotypes reveal novel loci	Breast Cancer Res.	Wrong population
Clinical Trial Registry	2003	An Investigational Drug (CAL) Versus Zoledronic Acid (ZometaÂ®) in Patients With Breast Cancer	https://clinicaltrials.gov/show/NCT00051779	Not fully published
Cros	2002	Constitutive production of parathyroid hormone-related protein (PTHrP) by fibroblasts derived from normal and pathological human breast tissue	Oncol Res	Wrong outcomes
Danks	1989	Parathyroid hormone-related protein: immunohistochemical localization in cancers and in normal skin	J Bone Miner Res	Wrong outcomes
Dawoud	2022	Expression Profile of Myoepithelial Cells in DCIS: Do They Change From Protective Angels to Wicked Witches?	Appl Immunohistochem Molecul Morphol	Wrong outcomes
Dodwell	1991	Parathyroid hormone-related protein(50-69) and response to pamidronate therapy for tumour-induced hypercalcaemia	Eur J Cancer	Wrong outcomes
Downey	1996	Differential expression of PTHrP and its receptor in breast cancer patients	Br. J. Cancer	Conference paper
Downey	1995	PARATHYROID HORMONE-RELATED PROTEIN - DETECTION AND PROGNOSIS IN BREAST-CANCER	Br. J. Surg.	Conference paper
Edwards	2023	Intracrine actions of the PTHrP nuclear localization sequence and C-terminus regulate cyclin dependent kinase inhibitor proteins to influence breast tumor growth	J. Bone Miner. Res.	Conference paper
Edwards	2022	Nuclear localizing signal and C-terminal domain influence the intracrine/paracrine actions of PTHrP in primary breast tumor growth and bone invasion	J. Bone Miner. Res.	Conference paper
Eriksson	2012	Genetic variants associated with breast size also influence breast cancer risk	BMC Med Genet	Did not assess PTHrP/PTHLH expression
Fraser	1993	CLINICAL AND LABORATORY STUDIES OF A NEW IMMUNORADIOMETRIC ASSAY OF PARATHYROID HORMONE-RELATED PROTEIN	Clin. Chem.	Wrong outcomes
Freeman	2015	The iCOGS breast cancer GWAS reveals 4 unique signals in the PTHLH region in patients of European origin	Cancer Research.	Conference paper

Freeman	2015	Analysis of the iCOGS breast cancer GWAS reveals 4 unique signals in the PTHLH region in patients of European origin	Cancer Research.	Conference paper
Freeman	2014	The parathyroid hormone-related peptide region likely features seven discrete breast cancer susceptibility loci	Cancer Research.	Conference paper
Gallacher	1990	Breast cancer-associated hypercalcaemia: a reassessment of renal calcium and phosphate handling	Ann Clin Biochem	Did not assess PTHrP/PTHLH expression
Ghoussaini	2012	Genome-wide association analysis identifies three new breast cancer susceptibility loci	Nature Genetics.	Did not assess PTHrP/PTHLH expression
Ghoussaini	2012	Genome-wide association analysis identifies three new breast cancer susceptibility loci	Nat Genet	Did not assess PTHrP/PTHLH expression
Gillespie	2001	PTHrP production by breast cancer cells stimulates osteoclast activity and bone resorption	Bone	Conference paper
Gollan	1997	Immunoreactive detection of parathormone related protein (PTHrP) in primary breast cancers is a good prognostic factor for subsequent bone metastases	Eur. J. Cancer	Conference paper
Grant	2023	The PTHrP nuclear localization sequence inhibits breast cancer bone colonization	J. Bone Miner. Res.	Conference paper
Grill	1991	Parathyroid hormone-related protein: elevated levels in both humoral hypercalcemia of malignancy and hypercalcemia complicating metastatic breast cancer	J Clin Endocrinol Metab	Wrong outcomes
Gurney	1993	Parathyroid hormone-related protein and response to pamidronate in tumour-induced hypercalcaemia	Lancet	Wrong population
Heath	1990	Parathyroid-hormone-related protein in tumours associated with hypercalcaemia	Lancet	Wrong population
Henderson	1995	Immunohistochemical detection of parathyroid hormone related protein in the primary tumours of hypercalcaemic breast cancer patients	The Breast	Wrong outcomes
Henderson	1995	IMMUNOHISTOCHEMICAL DETECTION OF PARATHYROID-HORMONE RELATED PROTEIN IN THE PRIMARY TUMORS OF HYPERCALCAEMNIC BREAST-CANCER PATIENTS	Breast	Overlapping samples
Henderson	1999	Production of PTHrP by primary breast cancers predicts improved patient survival and decreased bone metastases	J. Bone Miner. Res.	Conference paper
Henderson	2003	Increased survival and decreased metastasis formation after ten years in patients with PTHrP-positive breast cancer; a prospective study in 526 patients	J. Bone Miner. Res.	Conference paper
Henderson	2004	Long-term follow-up of a parathyroid hormone-related	J. Bone Miner. Res.	Conference paper

		protein in breast cancer		
Henderson	2001	Parathyroid hormone-related protein production by breast cancers, improved survival, and reduced bone metastases	J Natl Cancer Inst	Overlapping samples
Ikeda	1988	MESSENGER-RNAS ENCODING A PTH-LIKE PEPTIDE IN A VARIETY OF MAMMALIAN TUMORS	Clin. Res.	Conference paper
Jin	2017	Clinical Characteristics, Causes and Survival in 115 Cancer Patients with Parathyroid Hormone Related Protein-mediated Hypercalcemia	J Bone Metab	Wrong population
Kamalakar	2017	PTHrP(12-48) Modulates the Bone Marrow Microenvironment and Suppresses Human Osteoclast Differentiation and Lifespan	J Bone Miner Res	Wrong outcomes
Kanbara	1993	[Immunohistological evaluation of parathyroid hormone-related protein in breast cancer with and without calcification on mammography]	Nihon Geka Gakkai Zasshi	Not in English
Kitazawa	1995	Development of skeletal metastases	Clin Orthop Relat Res	Overlapping samples
Kohno	1994	Parathyroid Hormone-related Protein in Breast Cancer Tissues: Relationship between Primary and Metastatic Sites	Breast Cancer	PTHrP/PTHLH after treatment
Kremer	2023	PTHrP expression in Circulating Tumour Cells from Breast Cancer Patients Without Hypercalcemia and Its Targeting with Anti-PTHrP Monoclonal Antibodies	J. Bone Miner. Res.	Conference paper
Kuchenbaecker	2013	Extended replication of a GWAS for breast cancer in BRCA2 mutation carriers	Medizinische Genetik	Conference paper
Lee	1997	Parathyroid hormone and parathyroid hormone related protein assays in the investigation of hypercalcemic patients in hospital in a Chinese population	J Endocrinol Invest	Wrong population
Liersch	1999	Parathyroid hormone-related protein (PTHrP) in breast cancer:: An auto-/paracrine growth factor?: Studies on cells of the bone marrow microenvironment	Langenbecks Arch. Surg.	Not in English
Liersch	1998	Detection of disseminated carcinoma cells in bone marrow and peripheral blood in primary breast cancer with RT/PCR of parathyroid hormone-related protein (PTHrP). [German]	Langenbecks Archiv fur Chirurgie	Not in English
Linforth	2001	Early breast cancers (EBC) expressing epidermal growth factor receptor (EGFR) have increased parathyroid hormone related protein (PTHrP) expression	Br. J. Surg.	Conference paper

Loibl	2006	[PTHrP and bone sialoprotein as prognostic markers for developing bone metastases in breast cancer patients]	Zentralbl Gynakol	Not in English
Motellon	2000	Parathyroid hormone-related protein, parathyroid hormone, and vitamin D in hypercalcemia of malignancy	Clin Chim Acta	Wrong population
Myoui	1997	TGF-beta activation of c-Src and subsequent stimulation of PTH-rP enhance osteolytic metastases associated with breast cancer	J. Bone Miner. Res.	Conference paper
Pecherstorfer	1994	Parathyroid hormone-related protein and life expectancy in hypercalcemic cancer patients	J Clin Endocrinol Metab	Wrong population
Pillay	1997	Association of hypercalcemia, PTHrP expression and disease progression in a woman with primary squamous cell carcinoma of the breast	Pathology	Case report
Powell	1991	THE PATTERN OF PARATHYROID HORMONE-RELATED PROTEIN DETECTED IMMUNOHISTOCHEMICALLY IN BREAST-CANCER METASTASES - A ROLE IN PATHOGENESIS	Aust. N. Z. J. Surg.	Conference paper
Purrington	2014	Expression quantitative trait locus analysis of triple negative breast cancer	Cancer Research.	Conference paper
Purrington	2014	Genome-wide association study identifies 25 known breast cancer susceptibility loci as risk factors for triple-negative breast cancer	Carcinogenesis	Did not assess PTHrP/PTHLH expression
Ratcliffe	1992	Role of assays for parathyroid-hormone-related protein in investigation of hypercalcaemia	Lancet	Wrong population
Rizzoli	1999	Serum parathyroid hormone-related protein levels and response to bisphosphonate treatment in hypercalcemia of malignancy	J. Clin. Endocrinol. Metab.	Wrong outcomes
Roskams	1993	Parathyroid hormone-related peptide expression in primary and metastatic liver tumours	Histopathology	Wrong patient population
Schweitzer	1994	Malignancy-associated hypercalcaemia: resolution of controversies over vitamin D metabolism by a pathophysiological approach to the syndrome	Clin Endocrinol (Oxf)	Wrong population
Shi	2010	Combined examination of the expression of BSP and PTHrP in detection of bone metastasis of breast cancer. [Chinese]	Chinese Journal of Cancer Prevention and Treatment	Not in English
Shi	2010	Combined examination of the expression of BSP and PTHrP in detection of bone metastasis of breast cancer	Chin. J. Cancer Prev. Treat.	Not in English

Soomro	1990	IMMUNOHISTOLOGY OF PARATHYROID HORMONE-RELATED PROTEIN IN BREAST-CARCINOMA USING A POLYCLONAL ANTIBODY	J. Pathol.	Conference paper
Southby	1995	ALTERNATIVE PROMOTER USAGE AND MESSENGER-RNA SPLICING PATHWAYS FOR PARATHYROID HORMONE-RELATED PROTEIN IN NORMAL-TISSUES AND TUMORS	Br. J. Cancer	Wrong outcomes
Takyar	2016	PTHRP activates STAT5 signaling in mammary epithelium and affects breast cancer initiation and progression	Journal of Bone and Mineral Research. Conference	Conference paper
Teraoka	2011	Single nucleotide polymorphisms associated with risk for contralateral breast cancer in the Women's Environment, Cancer, and Radiation Epidemiology (WECARE) Study	Breast Cancer Res	Did not assess PTHrP/PTHLH expression
Teraoka	2011	Single nucleotide polymorphisms associated with risk for contralateral breast cancer in the Women's Environment, Cancer, and Radiation Epidemiology (WECARE) Study	Breast cancer research : BCR	Did not assess PTHrP/PTHLH expression
Truong	2003	Parathyroid hormone-related peptide and survival of patients with cancer and hypercalcemia	Am J Med	Wrong population
Vargas	1992	Tissue localization of parathyroid hormone-related protein mRNA in breast cancer and metastatic lesions	Bone and Mineral	Conference paper
Vargas	1992	Localization of parathyroid hormone-related protein mRNA expression in breast cancer and metastatic lesions by in situ hybridization	J Bone Miner Res	Overlapping samples
Vashum	2021	Inhibitory Effects of Cathepsin K Inhibitor (ODN-MK-0822) on the Paracrine Pro-Osteoclast Factors of Breast Cancer Cells	Curr Mol Pharmacol	Wrong outcomes
Wada	1990	[A parathyroid hormone related protein (PTHrP) implicated in hypercalcemia associated with malignancy: research of the PTHrP for novel hormonal tumor marker]	Rinsho Byori	Not in English
Walls	1994	Parathyroid hormone and parathyroid hormone-related protein in the investigation of hypercalcaemia in two hospital populations	Clin Endocrinol (Oxf)	Wrong outcomes
Washam	2011	P4-16-01: Proteomic Analysis of Patient Plasma Identifies Parathyroid Hormone Related Protein (PTHrP12-48) as a Potential Biomarker of Breast Cancer Bone	Cancer Research	Conference paper

Washam	2011	Proteomic analysis of patient plasma identifies parathyroid hormone related protein (PTHrP12-48) as a potential biomarker of breast cancer bone metastasis	Cancer Research.	Conference paper
Washam	2011	Identification of plasma biomarkers that predict breast cancer bone metastasis	Bone	Conference paper
Wimalawansa	1994	SIGNIFICANCE OF PLASMA PTH-RP IN PATIENTS WITH HYPERCALCEMIA OF MALIGNANCY TREATED WITH BISPHOSPHONATE	Cancer	Wrong population
Xu	2020	Case report: 16-yr life history and genomic evolution of an ER+ HER2âˆ’ breast cancer	Cold Spring Harb. Mol. Case Stud.	Case report
Xu	2020	Case report: 16-yr life history and genomic evolution of an E+ HER2- breast cancer	Cold Spring Harb. Mol. Case Stud.	Case report
Zeng	2016	Identification of independent association signals and putative functional variants for breast cancer risk through fine-scale mapping of the 12p11 locus	Breast Cancer Res.	Did not assess PTHrP/PTHLH expression
		**Studies Excluded from the Citation Chasing Results**		
**First Authors Last Name**	**Year**	**Title**	**Journal**	**Reason for Exclusion**
Shalaby	2024	Immunohistochemical expression of Parathyroid hormone-related protein and Ezrin in Invasive Breast Carcinoma of No Special Type: A retrospective Analysis	Preprint – Research Square	Duplicate study data
