# Supplementary material for: Clinical and prognostic significance of parathyroid hormone-related protein in breast cancer: a systematic review and meta-analyses of observational studies in women
Source: Endocr Relat Cancer. 2026 Mar 5;33(3):e250324. doi: 10.1530/ERC-25-0324 (PMC12978662; doi:10.1530/ERC-25-0324)
Supplement: Supplementary file 12 [file supplementary_table_4.pdf]

Supplementary Table 4. Extraction sheet: Association between PTHrP/*PTHLH* expression and breast cancer prognostic factors

First author, year, country, study design	N	Participant characteristics	Tumor characteristics	Tissue sample(s) type and preprocessing	PTHrP/*PTHLH* measurement(s)	Studied factor(s) (categorization)	Crude association
Southby *et al.*, 1990, Australia, cohort study	102	- *Period of recruitment:* August 1987 to March 1989<br>- *Age:*<br>30/102 29% <50 years<br>61/102 60% >51 years<br>11/102 11% missing<br>- *Ethnicity:* NR<br>- *Menopausal status:* NR<br>- *Parity status:* NR<br>- *Follow-up:* NR<br>- *Treatment regimen:* Of the 91 patients who did not present with a local recurrence, 78% had a mastectomy, 20% had breast conservation and 2% had biopsy alone. In addition, 85% had an axillary clearance. Patients considered to have a high risk of recurrence were given adjuvant chemotherapy (25%), tamoxifen (29%) and radiotherapy (25%).<br><br>**Calcemia:**<br>- *Status:* 102/102 100% normocalcemic<br>- *Method of diagnostic:* NR	**Breast tumor:**<br>- *Stage[A]:*<br>19/102 19% stage 1<br>46/102 45% stage 2<br>19/102 19% stage 3<br>7/102 7% stage 4<br>11/102 11% missing<br>- *Grade[B]:*<br>8/102 8% grade I<br>32/102 31% grade II<br>45/102 44% grade III<br>17/102 17% missing<br>- *Histological types:*<br>52/102 51% infiltrating ductal<br>22/102 22% infiltrating ductal + ductal carcinoma *in situ*<br>5/102 5% infiltrating lobular<br>3/102 3% mucinous<br>5/102 5% anaplastic<br>4/102 4% tubular/papillary/cribriform<br>11/102 11% missing<br>- *Molecular subtypes:*<br>ER[C]+ 54/102 53% (15/102 15% missing)<br>PR[C]+ 49/102 48% (26/102 25% missing)<br>HER2+ NR<br>Ki67 NR<br><br>**Metastases:**<br>- *N:* 7/102 (7%) stage 4 at time of diagnosis, 17/102 (17%) missing<br>- *Method of diagnostic:* NR	- *Sample type:* tumor<br>- *Tumor cells :* NR<br>- *Sampling method:* surgery or biopsy<br>- *Sample fixation:* fixed in 10% buffered formalin for 12-24 hours and paraffin-embedded<br>- *Samples storage:* NR<br>- *RNA extraction method:* NA<br>- *RNA quality assessment:* NA<br>- *cDNA synthesis method:* NA	- *Measurement method:* IHC<br>- *Antibodies/probes:* New Zealand white rabbits' polyclonal antibody against PTHrP(1-34) (1:25 and 1:50 dilutions)<br>- *Housekeeping gene(s):* NA<br>- *Quantification methods:*<br>• Positive/negative = tumors were called positive if at least 1 tumoral cell was stained<br>• Intensity of staining = no staining of tumor cells (0), weak positive staining (1), moderately strong positive staining (2), strong positive staining (3)<br>• Type of staining = cytoplasmic pattern, vesicular pattern, mixed pattern<br>• Area of staining = estimate percentage of cells in the section which stained positively, 0-5% of positive tumor cells (1), 5-25% of positive tumor cells (2), 26-50% of positive tumor cells (3) and >50% positive cells (4)<br>• Staining index = area times intensity of staining, weak (1 and 2), moderate (3 and 4) and strong (>6)<br>- *Positive controls:* squamous cell carcinoma of the skin<br>- *Negative/specificity controls:* Alternative deletion of the antibody layers, pre-absorption of the primary antibody with 0.5mg/ml PTHrP(1-34), application of 0.5mg/ml PTHrP(1-34) to the section 5 minutes before the addition of the primary antibody, replacement of the primary antibody with non-immune rabbit serum<br>- *Reproducibility assessments:* Each tumor was assessed independently by two observers with ~95% agreement, when the two were not in concordance, the tumor was re-stained and reassessed by a panel of five	- Age (<50, >51 years)<br>- Tumor size (mm)<br>- Histological type (infiltrating ductal, infiltrating ductal + ductal carcinoma *in situ*, infiltrating lobular, mucinous, anaplastic, tubular/papillary/cribriform<br>- Grade[B] (I, II, III)<br>- Nodal status (No nodal involvement, 1-3 positive nodes, >3 positive nodes)<br>- Stage[A] (1, 2, 3, 4)<br>- ER[C] status (positive, negative)<br>- PR[C] status (positive, negative)<br>- Calcifications (absence, low, high)<br>- St Vincent's Hospital Prognostic index score (N+E+P+A), subdivided in high >27 *versus* low ≤27:<br>• N<br>If no positive node N = 0, if 1-3 positive node(s) N = 13, if >3 positive nodes N = 31<br>• E<br>If ER- E = 0, if ER+ E = 15<br>• P<br>If PR- P = 0, if PR+ P = 12.5<br>• A<br>Number of years over 65	**All patients:**<br>*Association (positive):*<br>- PR status<br>Tumors were more frequently PR+ in PTHrP-positive tumors (32/54, 65%) compared to PTHrP-negative tumors (19/37, 51%) Fisher exact test P = 0.039<br><br>*No association:*<br>- Age<br>- Grade<br>- ER status<br>- Histological type<br>- Stage<br>- Nodal status<br>- Tumor size<br>- Calcifications<br><br>**Subgroup analysis:**<br>In group A patients (*n*=84 women with stage 1-3 cancers) with evaluable St Vincent's Hospital Prognostic index score (*n*=57), 21/29 patients at low risk were positive for PTHrP expression while 13/28 patients at high risk were positive for PTHrP expression.<br>Chi-squared = 4.0<br>p = 0.046

					observers, including the two initial observers and three observers blinded from clinicopathological data as well as previous results.<br>- *Statistical analysis:* qualitative<br>• Positive/negative<br>semiquantitative<br>• Intensity of staining (1, 2, 3)<br>• Area of staining (<5%, 6-25%, 26-50%, >51%)<br>• Staining index (weak, moderate, strong)<br>• Type of staining (cytoplasmic, vesicular, mixed)<br>Frequency data were analyzed by the chi-squared test or Fisher's exact test. Means were compared with Student's *t* test.		
Bundred *et al.*, 1992, UK, cohort study	155	- *Period of recruitment:* August 1984 to December 1985<br>- *Age:*<br>65/155 42% ≤ 50 years<br>90/155 58% ≥ 51 years<br>- *Ethnicity:* NR<br>- *Menopausal status:*<br>43/155 28% premenopausal<br>112/155 72% postmenopausal<br>- *Parity status:* NR<br>- *Follow-up:* ≥ 5 years or until death, all patients were seen every 4 months in the first year after surgery and thereafter at 6-month intervals.<br>- *Treatment regimen:* mastectomy with axillary clearance or node sampling (97/155, 63%), or breast conserving operations and radiotherapy (58/155, 37%)<br><br>**Calcemia:**<br>- *Status:* 155/155 100% normocalcemic<br>- *Method of diagnostic:* the upper limit of the reference range for corrected calcium was 2.65mmol/l	**Breast tumor:**<br>- *Stage:* NR<br>- *Grade*[D]*:*<br>30/155 19% grade I<br>88/155 57% grade II<br>37/155 24% grade III<br>- *Histological types:*<br>126/155 81% infiltrating ductal<br>29/155 19% other types<br>- *Molecular subtypes:*<br>ER[E]+ 64/155 41% (55/155 35% missing)<br>PR[E]+ 55/155 35% (69/155 45% missing)<br>HER2+ NR<br>Ki67+ NR<br><br>**Metastases:**<br>- *N:* none of the patients had clinical evidence of bone metastases at presentation<br>- *Method of diagnostic:* bone scan/scintigraphy, conventional radiography, ultrasound and/or surgical biopsy	- *Sample type:* tumor<br>- *Tumor cells :* NR<br>- *Sampling method:* surgery<br>- *Sample fixation:* 4% formaldehyde in saline and paraffin embedding<br>- *Samples storage:* NR<br>- *RNA extraction method:* NA<br>- *RNA quality assessment:* NA<br>- *cDNA synthesis method:* NA	- *Measurement method:* IHC<br>- *Antibodies/probes:* rabbit anti PTHrP(37-67)<br>- *Housekeeping gene(s):* NA<br>- *Quantification methods:* only cytoplasmic staining was considered<br>- *Positive controls:* squamous carcinoma of the lung included in each batch of slides<br>- *Negative/specificity controls:* preincubation of the primary antibody with 0.5mg/ml PTHrP(37-67), omission of the primary antibody, primary antibody replaced by non-immune serum<br>- *Reproducibility assessments:* staining was assessed by two independent observers<br>- *Statistical analysis:* qualitative (positive *versus* negative)<br>All statistical comparisons were done by chi-squared tests, except for tumor size which was compared by Mann-Whitney test.	- Age (≤ 50 years, ≥ 51 years)<br>- Tumor size (mm)<br>- Grade[D] (I, II, III)<br>- Histological type (infiltrating ductal, other types)<br>- Nodal status (positive, negative)<br>- ER[E] status (positive, negative)<br>- PR[E] status (positive, negative)<br>- Menopausal status (premenopausal, postmenopausal)	**<u>All patients:</u>**<br>*No association:*<br>- Age<br>- Tumor size<br>- Grade[D]<br>- Histological type<br>- Nodal status<br>- ER[E] status<br>- PR[E] status<br><br>- Menopausal status 64/87 (74%) patients with PTHrP-positive tumor were postmenopausal compared to 48/68 (71%) patients with PTHrP-negative tumor
Bouizar *et al.*,	38	- *Period of recruitment:*	**Breast tumor:**	- *Sample type:* tumor	- *Measurement method:* RT-PCR	- Age (years)	**<u>All patients:</u>**

1993, France, cohort study		between 1981 and 1989<br>- *Age:* mean = 58 years, range 38-79 years<br>13/38 34% ≤ 50 years<br>25/38 66% > 51 years<br>- *Ethnicity:* NR<br>- *Menopausal status:*<br>14/38 37% premenopausal<br>24/38 63% postmenopausal<br>- *Parity status:* NR<br>- *Follow-up:* at least 5 years<br>- *Treatment regimen:* NR<br><br>**Calcemia:**<br>- *Status:* 38/38, 100% normocalcemic<br>- *Method of diagnostic:* NR	- *Stage[F]:*<br>4/38 11% stage 1<br>21/38 55% stage 2<br>13/38 34% stage 3<br>- *Grade[G]:*<br>0/38 0% grade I<br>19/38 50% grade II<br>11/38 29% grade III<br>8/38 21% missing<br>- *Histological types:*<br>34/38 89% infiltrating ductal carcinoma<br>2/38 5% infiltrating lobular carcinoma<br>1/38 3% adenocarcinoma<br>1/38 3% medullary carcinoma<br>- *Molecular subtypes:*<br>ER[H]+ NR<br>PR[H]+ NR<br>HER2+ NR<br>Ki67 NR<br><br>**Metastases:**<br>- *N:*<br>11/38, 29% without recurrence nor metastases<br>10/38, 26% who developed metastases in soft tissues<br>17/38, 45% who developed bone metastases<br>- *Method of diagnostic:* NR	- *Tumor cells :* selected by a pathologist<br>- *Sampling method:* surgery<br>- *Sample fixation:* fresh frozen in liquid nitrogen<br>- *Samples storage:* liquid nitrogen tumor bank<br>- *RNA extraction method:* guanidinium thiocyanate-phenol-chloroform method<br>- *RNA quality assessment:* NR<br>- *cDNA synthesis method:* Moloney murine leukemia virus reverse transcriptase (BRL; Gibco France, Cergy, Paris, France)	- *Antibodies/probes: F 5'-GCGACGATTCTTCCTTCACC-3' and R 5'-AGAGTCTAACCAGGCAGAGC-3'*<br>- *Housekeeping gene(s):* NA<br>- *Quantification methods:* densitometric scanning<br>- *Positive controls:* NR<br>- *Negative/specificity controls:* absence of Moloney murine leukemia virus reverse transcriptase during cDNA preparation, reverse transcription done after digestion of RNA by RNAse A (*n* = 4)<br>- *Reproducibility assessments:* PCR and densitometric scanning were performed twice for 12 tumors r = 0.92, p < 0.005, densitometric values were the mean of 3 scans<br>- *Statistical analysis:* continuous (absorbance)<br>Data were analyzed using parametric (analysis of variance and regression) and nonparametric (Kruskal Wallis and Mann-Whitney tests, Spearman rank correlation)	- Menopausal status (pre-, post-menopausal)<br>- Tumor size (≤2cm, >2cm)<br>- Stage[F] (1, 2, 3)<br>- Nodal status (ND, 1-3, >3)<br>- Histological type (infiltrating ductal carcinoma, infiltrating lobular carcinoma, adenocarcinoma, medullary carcinoma)<br>- ER[H] status (<10fmol/mg of protein, ≤ 10fmol/mg of protein)<br>- PR[H] status (<10fmol/mg of protein, ≤ 10fmol/mg of protein)<br>- Tumor calcification (absent, present)<br>- Grade[G] (I, II, III)	*Association:*<br>- Nodal status<br>rs = 0.394, p < 0.02<br>- Age<br>r = -0.379, p < 0.02<br><br>*No association:*<br>- Menopausal status<br>- Tumor size<br>- Stage[F]<br>- Histological type<br>- ER[H] status<br>- PR[H] status<br>- Tumor calcification<br>- Grade[I]
Fraser *et al.*, 1993, UK, cross-sectional study	11	- *Period of recruitment:* NR<br>- *Age:* NR<br>- *Ethnicity:* NR<br>- *Menopausal status:* NR<br>- *Parity status:* NR<br>- *Follow-up:* NR<br>- *Treatment regimen:* NR<br><br>**Calcemia:**<br>- *Status:* 11/11 (100%) hypercalcemic<br>- *Method of diagnostic:* >2.7 mmol/L serum calcium adjusted for albumin	**Breast tumor:**<br>- *Stage*: NR<br>- *Grade:* NR<br>- *Histological types:*<br>4/11 % ductal carcinoma<br>6/11 % lobular carcinoma<br>1/11 % papillary carcinoma<br>1/38 3%<br>- *Molecular subtypes:*<br>ER+ NR<br>PR+ NR<br>HER2+ NR<br>Ki67+ NR<br><br>**Metastases:**<br>- *N:* NR<br>- *Method of diagnostic:* radionuclide bone scans, not all patients had a bone scan	- *Sample type:* blood (plasma)<br>- *Tumor cells :* NR<br>- *Sampling method:* venipuncture done between 10AM and 12PM. All samples for PTHrP measurement were collected into evacuated collection tubes supplied by the Nichols Institute and containing aprotinin (2500 kallikrein units/tube), leupeptin (25 μg/tube), pepstatin (25 μg/tube) and EDTA (4.5 mg/tube). Tubes were kept on ice before centrifugation. All plasma samples were obtained within 30	- *Measurement method:* IRMA<br>- *Antibodies/probes:* Capture anti-body against PTHrP(60-72) and radiolabeled antibody against PTHrP(1-40)<br>- *Housekeeping gene(s):* NR<br>- *Quantification methods:* quantitative, PTHrP was considered high if it exceeds the 95% reference interval obtained in normal subjects (>2.6 pmol/L)<br>- *Positive controls:* standard<br>- *Negative/specificity controls:* zero standard<br>- *Reproducibility assessments:* NR<br>- *Statistical analysis:* NR	- Histological types (ductal carcinoma, lobular carcinoma, papillary carcinoma)	**<u>All patients:</u>**<br>3/4 (75%) patients with ductal carcinoma had increased circulating PTHrP level<br><br>5/6 (83%) patients with lobular carcinoma had increased circulating PTHrP level<br><br>0/1 (0%) patients with papillary carcinoma had increased circulating PTHrP level

				minutes after blood collection and frozen immediately after separation.<br>- *Sample fixation:*<br>- *Samples storage:* -70°C and thawed for 60 minutes at room temperature before assay.<br>- *RNA extraction method:* NA<br>- *RNA quality assessment:* NA<br>- *cDNA synthesis method:* NA			
Kissin *et al.*, 1993, Australia, cohort study	82	- *Period of recruitment:* between 1984 and 1987<br>- *Age:* mean =<br>14/82 17% <50 years<br>68/82 83% >50 years<br>- *Ethnicity:* NR<br>- *Menopausal status:* NR<br>- *Parity status:* NR<br>- *Follow-up:* at least 3 years<br>- *Treatment regimen:*<br>9/82, 11% needle biopsy<br>15/82, 18% wide local excision<br>58/82, 71% total mastectomy<br><br>63/82, 77% axillary dissection<br><br>22/82, 27% adjuvant tamoxifen<br>16/82, 20% chemotherapy<br>27/82, 33% radiotherapy<br><br>**Calcemia:**<br>- *Status:* 13/82, 16% patients developed symptomatic hypercalcemia and/or prolonged hypercalcemia during the period of follow up.<br>- *Method of diagnostic:* NR	**Breast tumor:**<br>- *Stage*[F]:<br>8/82 10% stage 1<br>40/82 49% stage 2<br>8/82 10% stage 3<br>26/82 32% stage 4<br><br>- *Grade:* NR<br>- *Histological types:* NR<br>- *Molecular subtypes:*<br>ER[I]+ 53/82 65%<br>PR[I]+ 47/82 57%<br>HER2+ NR<br>Ki67+ NR<br><br>**Metastases:**<br>- *N:* 26/82, 32% patients had metastases at time of initial diagnosis<br>- *Method of diagnostic:* chest X-ray and bone scan	- *Sample type:* tumor<br>- *Tumor cells :* NR<br>- *Sampling method:* surgery<br>- *Sample fixation:* 10% buffered formalin for 12 to 24 hours and embedded in paraffin<br>- *Samples storage:* NR<br>- *RNA extraction method:* NA<br>- *RNA quality assessment:* NA<br>- *cDNA synthesis method:* NA	- *Measurement method:* IHC<br>- *Antibodies/probes:* rabbit polyclonal antibody against PTHrP(1-34)<br>- *Housekeeping gene(s):* NA<br>- *Quantification methods:*<br>A tumor was called positive for PTHrP if any of the tumor cells were specifically stained brown (cytoplasmic and/or vesicular).<br>• Localization = cytoplasmic, vesicular, mixed<br>• Intensity (subjective) = weak (1), moderate (2), strong (3)<br>• Area of staining (percent positive tumor cells) = <5% (1), 6-25% (2), 26-50% (3), >50% (4)<br>• Staining index (intensity x area of staining) = 1 and 2 (weak), 3 and 4 (moderate), >6 (strong)<br>- *Positive controls:* squamous cell carcinoma of the skin<br>- *Negative/specificity controls:* alternate deletions of the primary antiserum, secondary antibody and peroxidase anti-peroxidase complex, pre-absorption of the anti-PTHrP(1-34) antiserum with 0.5 mg/ml PTHrP(1-34) overnight at 4C, sections of breast tumors and squamous cell carcinoma of the skin where the specific antiserum was replaced with non-immune rabbit serum<br>- *Reproducibility assessments:* each tumor was stained twice, and in duplicate, evaluation of staining was done by 1 the principal investigator and subsequently by a panel of 4 observers with no	- St Vincent's prognostic index[J] (group 1, group 2, group 3) including:<br>• Age<br>• Nodal status<br>• ER[I] status<br>• PR[I] status<br><br>- Microcalcifications (presence, absence)	**All patients:**<br>*Association:*<br>- Presence of microcalcifications, PTHrP staining was positive in 24/30 (80%) tumors with microcalcifications compared to 6/29 (21%) tumors without microcalcifications Chi-square p < 0.05<br><br>*No association:*<br>- St Vincent's prognostic index[H]

					knowledge of the patient data. If there was a disagreement between observers, the tumor was restained and reassessed.<br>- *Statistical analysis:* semiquantitative<br>• Intensity of staining (1, 2, 3)<br>• Area of staining (<5%, 6-25%, 26-50%, >51%)<br>• Staining index (weak, moderate, strong)<br>Frequency data were analyzed with the chi-squared test and the Fisher's exact test when numbers were less than 20. Means were compared with Student's *t* test.		
Liapis *et al.*, 1993, USA, cross-sectional study	81	- *Period of recruitment:* NR<br>- *Age:*<br>33/81 41% < 50 years<br>48/81 59% > 50 years<br>- *Ethnicity:* NR<br>- *Menopausal status:* pre- and postmenopausal<br>- *Parity status:* NR<br>- *Follow-up:* NR<br>- *Treatment regimen:* NR<br><br>**Calcemia:**<br>- *Status*: 21/81 have been tested, 21/21, 100% normocalcemic<br>- *Method of diagnostic:* calcium concentration between 8.3 and 9.5mg/dl (normal range 9.2 to 11mg/dl)	- *Stage[A]:*<br>58/81 72% stage 1<br>20/81 25% stage 2<br>3/81 4% stage 3<br>- *Grade[K]:*<br>23/81 28% grade 1<br>28/81 35% grade 2<br>12/81 15% grade 3<br>18/81 22% unknown<br>- *Histological types:*<br>41/81 51% infiltrating ductal carcinoma<br>17/81 21% ductal carcinoma *in situ*<br>20/81 25% combined ductal *in situ* and invasive carcinoma<br>2/81 2% invasive lobular carcinoma<br>1/81 1% invasive mucinous carcinoma<br>- *Molecular subtypes:*<br>ER[L]+ 15/81 19% (55/81 68% missing)<br>PR[L]+ 18/81 22% (55/81 68% missing)<br>HER2+ NR<br>Ki67+ NR<br><br>**Metastases:**<br>- *N:* 0/81 0% at time of surgery<br>- *Method of diagnostic:* NR	- *Sample type:* tumor<br>- *Tumor cells :* NR<br>- *Sampling method:* surgical breast biopsy or mastectomy<br>- *Sample fixation:* 10% formalin fixed, and paraffin embedded<br>- *Samples storage:* NR<br>- *RNA extraction method:* NA<br>- *RNA quality assessment:* NA<br>- *cDNA synthesis method:* NA	- *Measurement method:* IHC<br>- *Antibodies/probes:* rabbit polyclonal anti-PTHrP(1-130), 1:400<br>- *Quantification methods:* Semiquantitative scale of 1 to 4+ intensity, 4+ being the maximum intensity observed for PTHrP-positive squamous carcinoma from patient with humoral hypercalcemia of malignancy<br>○ Strongly positive = >50% of positive cells and ≥3+ intensity<br>○ Moderately positive = 30 to 50% positive cells and ≥2+ intensity<br>○ Equivocal = 5 to 30% positive cells and ≥1+<br>Negative = <5% positive cells<br>- *Positive controls:* parathyroid gland and squamous cell carcinoma<br>- *Negative/specificity controls:* sections incubated with preimmune rabbit IgG and preadsorbed anti-PTHrP(1-130) in PTHrP fusion peptide<br>- *Reproducibility assessments:* NR<br>- *Statistical analysis:* qualitative (presence *versus* absence), semiquantitative (strongly positive, moderately positive, equivocal, negative)<br>Statistical analysis was performed using the chi-squared test to compare the proportions between groups. Power analysis was used when analyzing data on ER/PR status and lymph node status in relation to PTHrP expression	- Grade[K] (I, II, III)<br>- Nodal status (>3 positive nodes, ≤3 positive nodes)<br>- ER[L] status (positive, negative)<br>- PR[L] status (positive, negative)<br>- Calcifications (presence, absence)<br>- Tumor histology (infiltrating ductal carcinoma, ductal carcinoma *in situ,* combined ductal *in situ* and invasive carcinoma, invasive lobular carcinoma, invasive mucinous carcinoma)	**<u>All patients:</u>**<br>*Association:*<br>- ≤3 positive nodes in 81% of PTHrP-positive tumors *versus* 50% of PTHrP-negative tumors Chi-square p < 0.02<br><br>- Presence of calcifications in 24/56 43% of PTHrP positive tumors *versus* 12% of PTHrP negative tumors Chi-square p < 0.007<br><br>*No association:*<br>- Grade[K]<br>- ER[L] status<br>- PR[L] status<br>- Tumor histology
Kitazawa *et*	35[M]	- *Period of recruitment:*	**Breast tumor:**	- *Sample type:* tumor	- *Measurement method:* IHC	- Histologic type	**<u>All patients:</u>**

al., 1994, Japan, cross-sectional study		NR<br>- *Age:* NR<br>- *Ethnicity:* NR<br>- *Menopausal status:* NR<br>- *Parity status:* NR<br>- *Follow-up:* NR<br>- *Treatment regimen:* NR<br><br>**Calcemia:**<br>- *Status:* NR<br>- *Method of diagnostic:* NR	- *Stage:*<br>2/35 6% T2n0<br>1/35 3% T2n1b<br>4/35 11% T2n2<br>1/35 3% T3n1b<br>1/35 3% T3n2<br>2/35 6% T4n3<br>24/35 69% Missing<br>- *Grade:* NR<br>- *Histological types:*<br>9/35 26% papillotubular<br>12/35 34% papillotubular + scirrhous<br>4/35 11% solidtubular + papillotubular<br>7/35 20% solidtubular + scirrhous<br>3/35 9% solidtubular<br>- *Molecular subtypes:* NR<br><br>**Metastases:**<br>- *N:* 11/35, 31% at time of surgery/autopsy<br>- *Method of diagnostic:* NR	- *Tumor cells:* NR<br>- *Sampling method:* autopsy or surgery<br>- *Sample fixation:* formalin-fixed and paraffin-embedded<br>- *Samples storage :* NR<br>- *RNA extraction method:* NA<br>- *RNA quality assessment:* NA<br>- *cDNA synthesis method:* NA	- *Antibody:* monocloncal antibody against h-PTHrP(1-34) designated 4B3<br>- *Housekeeping gene(s):* NA<br>- *Quantification methods:*<br>• Percent positive cells : no staining (-), <10% (+-), 10% to 50% (+) and >50% (++)<br>- *Positive controls:* normal tissue (skin, esophagus, bladder, cervix, mammary gland, acanthotic cell layers of the squamous epithelia in the skin, parathyroid gland, pancreatic islet, adrenal cortex, meningoepithelial cells, renal tubules, placenta)<br>- *Negative/specificity controls:* basal cells of the squamous epithelia in the skin, epithelia of stomach and intestine, hepatocytes, renal glomeruli<br>- *Reproducibility assessments:* NR<br>- *Statistical analysis:* categorical (-, +-, +, ++)	(papillotubular, papillotubular + scirrhous, solidtubular + papillotubular, solidtubular + scirrhous, solidtubular)<br>- Presence of microcalcifications	*Association:*<br>- Papillotubular adenocarcinoma had stronger immunoreactivity than solidtubular adenocarcinoma<br><br>- Positive correlation between PTHrP expression and the presence of calcified lesion in mammary carcinoma
Kohno *et al.*, 1994, Japan, cohort study	28	- *Period of recruitment:* between 1980 and 1985<br>- *Age:*<br>Group A, *n*=12 (skeletal metastases) 51±9 years<br>Group B, *n*=8 (lung metastases) 52±13 years<br>Group C, *n*=8 (no distant metastasis) 51±12 years<br>- *Ethnicity:* NR<br>- *Menopausal status:* NR<br>- *Parity status:* NR<br>- *Follow-up:* 8 years<br>- *Treatment regimen:* 28/28, 100% mastectomy<br><br>**Calcemia:**<br>- *Status:* NR<br>- *Method of diagnostic:* NR	**Breast tumor:**<br>- *Stage:* NR<br>- *Grade*[B]*:*<br>7/28 25% grade I<br>13/28 46% grade II<br>8/28 29% grade III<br>- *Histological types:* 28/28, 100% infiltrating ductal carcinoma<br>- *Molecular subtypes:*<br>ER[N]+ 10/28 36% (8/28, 29% missing)<br>PR[N]+ 10/28 36% (8/28, 29% missing)<br>HER2+ NR<br>Ki67 NR<br><br>**Metastases:**<br>- *N:* 12/28, 43% bone metastases, 8/28, 29% lung metastases<br>- *Method of diagnostic:* bone scanning, plain X-rays films	- *Sample type:* tumor<br>- *Tumor cells:* NR<br>- *Sampling method:* mastectomy<br>- *Sample fixation:* formalin-fixed and paraffin embedding<br>- *Samples storage :* NR<br>- *RNA extraction method:* NA<br>- *RNA quality assessment:* NA<br>- *cDNA synthesis method:* NA	- *Measurement method:* IHC<br>- *Antibody:* monocloncal antibody against PTHrP(1-34) designated 4B3<br>- *Housekeeping gene(s):* NA<br>- *Quantification methods:*<br>• Grade of the intensity of staining: no positive staining (0), weak positive staining (1), moderate staining (2), strongly positive staining (3)<br>• Grade of the area of staining: the percentage of stained cells in each section, no positive tumor cells (0), 1%-5% positive tumor cells (1), 6%-25% positive tumor cells (2), 26%-50% positive tumor cells (3), >50% positive tumor cells (5)<br>• Degree of staining: as sum of staining grades of the intensity and area, 1-2 (-) , 3 (±), 4-5 (+) and 6 (++)<br>Of note: mostly cytoplasmic<br>- *Positive controls:* NR<br>- *Negative/specificity controls:* non-immunized mouse serum<br>- *Reproducibility assessments:* NR<br>- *Statistical analysis:* frequences in degree of staining (-, ±, +, ++)<br>The statistical analysis of frequency data was done by Yates' corrected chi-squared test	- Age (years)<br>- Tumor size (NR)<br>- Grade[B] (I, II, III)<br>- Number of lymph node metastases (continuous)<br>- ER[N] status (positive, negative)<br>- PR[N] status (positive, negative)	**All patients:**<br>*Association:*<br>- ER[N] status<br>- PR[N] status<br>Staining grades ++ and + were more common in ER+ and PR+ tumors than grades ± and – Yates' corrected chi-square test or Fisher's exact test P < 0.01<br><br>*No association:*<br>- Age<br>- Tumor size<br>- Grade[B]<br>- Number of lymph node metastases

					or Fisher's exact test		
Edwards *et al.*, 1995, UK, cross-sectional study	147[O]	- *Period of recruitment:* NR<br>- *Age:* mean = 61 years, range 35 to 90 years<br>- *Ethnicity:* NR<br>- *Menopausal status:*<br>41/147 28% premenopausal<br>106/147 72% postmenopausal<br>- *Parity status:* NR<br>- *Follow-up:* NR<br>- *Treatment regimen:* surgery<br><br>**Calcemia:**<br>- *Status:* all patients were normocalcemic at time of surgery<br>- *Method of diagnostic:* NR	**Breast tumor:**<br>- *Stage:* NR<br>- *Grade[D]:*<br>12/147 8% grade I<br>56/147 38% grade II<br>42/147 29% grade III<br>37/147 25% missing<br>- *Histological types:*<br>101/147 69% ductal<br>17/147 12% lobular<br>29/147 20% missing<br>- *Molecular subtypes:*<br>ER[P]+ 57/147 39% (61/147, 41% missing)<br>PR[P]+ 37/147 25% (61/147, 41% missing)<br>HER2+ NR<br>Ki67 NR<br><br>**Metastases:**<br>- *N:* NR<br>- *Method of diagnostic:* NR	- *Sample type:* tumor and blood (plasma)<br>- *Tumor cells :* NR<br>- *Sampling method:*<br>**Tumor:**<br>Following its excision, 0.2 to 1.0g of tumor was cut into pieces of ~0.5cm<br>**Blood:**<br>Collected pre-operatively in presence of EDTA, separated within 15 minutes<br>- *Sample fixation:*<br>**Tumor:**<br>immediately frozen in liquid nitrogen<br>**Blood:** NA<br>- *Samples storage:*<br>**Tumor:** stored at -70°C until protein extraction<br>**Blood:** stored at -20°C until assay<br>- *RNA extraction method:* NA<br>- *RNA quality assessment:* NA<br>- *cDNA synthesis method:* NA	- *Measurement method:* two-site IRMA<br>- *Antibodies/probes:* anti-PTHrP(1-86)<br>- *Housekeeping gene(s):* NR<br>- *Quantification methods:* continuous, based on standard curves<br>- *Positive controls:* NR<br>- *Negative/specificity controls:* NR<br>- *Reproducibility assessments:*<br>**Tumor:** within-batch assay coefficient of variation (n = 7) ≤ 20%<br>**Blood:** NR<br>- *Statistical analysis:*<br>**Tumor:** continuous and qualitative (presence, absence)<br>The relationship between tumor PTHrP levels or positivity and prognostic factors was assessed by Kruskal Wallis, chi-squared test or product moment correlation<br>**Blood:** NR	**Tumor:**<br>- Age (years)<br>- Menopausal status (premenopausal, postmenopausal)<br>- Grade[D] (I, II, III)<br>- Histological type (lobular, ductal)<br>- Nodal status (positive, negative),<br>- ER[P] status (positive, negative)<br>- PR[P] status (positive, negative)<br><br>**Blood:** NR	**Tumor:**<br>**<u>All patients:</u>**<br>*Association:*<br>- Menopausal status: in premenopausal, median PTHrP(1-86) = 275 fmol/g range 52 to 11,576 fmol/g while in postmenopausal, median = 174 fmol/g range 38 to 22,710 fmol/g<br>p<0.05<br>PTHrP(1-86) was detected in higher proportion of tumors from pre- (34/41, 83%) *versus* postmenopausal (61/106, 58%) patients. Chi-square p<0.05<br>- Age<br>PTHrP was inversely correlated with age. r = -0.22<br>p < 0.05<br><br>**<u>Subgroup:</u>**<br>**Pre-, post-menopausal**<br>*Association:*<br>- Nodal status (n=108): frequency of detectable PTHrP was higher in pre-menopausal patients with positive axillary node (91%) compared to pre-menopausal patients with negative axillary node (42%)<br>p < 0.05<br><br>*No association:*<br>- Grade[D]<br>- Histological type<br>- ER[O] status<br>- PR[O] status<br><br>**Blood:**<br>Plasma PTHrP(1-86) was <0.23pmol/l in 46 unselected patients prior to surgery, 32 of whom had tumors containing immunoreactive PTHrP(1-86).
Henderson *et al.*, 1995, Australia,	33	- *Period of recruitment:* between 1984 and 1987<br>- *Age:* median = 49	**Breast tumor:**<br>- *Stage[F]:*<br>7/33 (21%) stage 1	- *Sample type:* tumor<br>- *Tumor cells :* examination of the	- *Measurement method:* IHC<br>- *Antibodies:* polyclonal antiserum (394.7) raised against the	- Tumor size<br>- Nodal status<br>- Age	**<u>All patients:</u>**<br>*No association:*<br>- Tumor size

cohort study		years, range 28 to 74 years<br>- *Ethnicity:* NR<br>- *Menopausal status:* NR<br>- *Parity status:* NR<br>- *Follow-up:* median = 57 months<br>- *Treatment regimen:* NR<br><br>**Calcemia:**<br>- *Status:* 33/33 (100%) subsequently developed hypercalcemia<br>- *Method of diagnostic:* albumin corrected calcium >2.7 mmol/L for 1 month	15/33 (45%) stage 2<br>5/33 (15%) stage 3<br>6/33 (18%) stage 4<br>- *Grade:* NR<br>- *Histological types:* NR<br>- *Molecular subtypes:*<br>ER[Q]+ 22/33, 67% (7/33, 21% missing)<br>PR[Q]+ 11/33, 33% (12/33, 36% missing)<br>HER2+ NR<br>Ki67+ NR<br><br>**Metastases:**<br>- *N:* 6/33, 18% stage 4 breast cancer at time of breast cancer diagnostic, 33/33, 100% of the patients by the time the study.<br>31/33 (94%) bone metastases<br>16/33 (48%) lung metastases<br>19/33 (58%) liver metastases<br>23/33 (70%) others<br>- *Method of diagnostic :* technetium bone scans, areas of abnormal tracer uptake were X-rayed. Patients with hypercalcemia but minimal or no evidence of bone metastases had a limited radiological survey. Radionuclide liver scans were performed in patients with liver enlargement or altered liver function tests. In a few patients who were considered too ill for investigation, liver metastases were considered to be present if there was gross hepatomegaly and abnormal liver function tests.<br>- *Bone markers:* NR<br>- *Subtypes:* NR	paraffin block sections to ensure the presence of tumor<br>- *Sampling method:* NR<br>- *Samples fixation:* NR<br>- *Samples storage:* NR<br>- *RNA extraction method:* NA<br>- *RNA quality assessment:* NA<br>- *cDNA synthesis method:* NA	synthetic peptide PTHrP(1-34)<br>- *Housekeeping gene(s):* NA<br>- *Quantification methods:* a tumor was called positive for PTHrP if any of the tumor cells were specifically stained brown (cytoplasmic and/or vesicular).<br>• Localization = cytoplasmic, vesicular, mixed<br>• Intensity (subjective) = weak (1), moderate (2), strong (3)<br>• Area of staining (percent positive tumor cells) = <5% (1), 6-25% (2), 26-50% (3), >50% (4)<br>• Staining index (intensity x area of staining) = 1 and 2 (weak), 3 and 4 (moderate), >6 (strong)<br>- *Positive controls:* NR<br>- *Negative controls:* NR<br>- *Reproducibility assessments:* each tumor was stained twice and in duplicate. A panel of 4 observers who had no knowledge of the patient data reviewed all the sections. If there was a disagreement between observers, the tumor was re-stained and reassessed.<br>- *Statistical analysis:* qualitative (positive *versus* negative) and semiquantitative<br>Frequency data were analyzed with the chi-squared test and the Fisher exact test for small numbers. Means were compared with Student's t-test	- Menopausal status<br>- ER status<br>- PR status<br>- Stage	- Nodal status<br>- Age<br>- Menopausal status<br>- ER status<br>- Stage<br><br>*Association:*<br>- PR status<br>14/14 (100%) PR+ tumors were also PTHrP-positive while 5/12 PR- tumors were PTHrP-positive<br>Chi-squared $p < 0.05$
Bundred *et al.*, 1996, UK, cross-sectional study	114	- *Period of recruitment:* NR<br>- *Age:* NR<br>- *Ethnicity:* NR<br>- *Menopausal status:* NR<br>- *Parity status:* NR<br>- *Follow-up:* NR<br>- *Treatment regimen:* NR<br><br>**Calcemia:**<br>- *Status:* NR<br>- *Method of diagnostic:* NR	**Breast tumor:**<br>- *Stage:* early breast cancers<br>- *Grade:* NR<br>- *Histological types:* NR<br>- *Molecular subtypes:*<br>ER+ NR<br>PR+ NR<br>HER2+ NR<br>Ki67+ NR<br><br>**Metastases:**<br>- *N:* NR<br>- *Method of diagnostic:* NR	- *Sample type:* tumor<br>- *Tumor cells :* NR<br>- *Sampling method:* NR<br>- *Sample fixation:* snap frozen in liquid nitrogen<br>- *Samples storage:* NR<br>- *RNA extraction method:* NA<br>- *RNA quality assessment:* NA<br>- *cDNA synthesis method:* NA	- *Measurement method:* IRMA<br>- *Antibodies/probes:* PTHrP(1-86)<br>- *Housekeeping gene(s):* NA<br>- *Quantification methods:* NR<br>- *Positive controls:* NR<br>- *Negative/specificity controls:* NR<br>- *Reproducibility assessments:* NR<br>- *Statistical analysis:* NR	- Age (years)<br>- Menopausal status (premenopausal, postmenopausal)<br>- Lymph node status (positive, negative)	**All patients:**<br>*Association:*<br>- Age:<br>Negative correlation between PTHrP and age<br><br>- Menopausal status:<br>Higher PTHrP in pre-menopausal women<br><br>**Subgroup: Pre-menopausal**<br>*Association:*<br>- Nodal status:

							The proportion of tumors with detectable PTHrP levels was higher in axillary node-positive premenopausal women<br>p ≤ 0.05
	185	- *Period of recruitment:* NR<br>- *Age:* NR<br>- *Ethnicity:* NR<br>- *Menopausal status:* NR<br>- *Parity status:* NR<br>- *Follow-up:* NR<br>- *Treatment regimen:* NR<br><br>**Calcemia:**<br>- *Status:* NR<br>- *Method of diagnostic:* NR	**Breast tumor:**<br>- *Stage:* early breast cancers<br>- *Grade:* NR<br>- *Histological types:* NR<br>- *Molecular subtypes:*<br>ER+ NR<br>PR+ NR<br>HER2+ NR<br>Ki67+ NR<br><br>**Metastases:**<br>- *N:* NR<br>- *Method of diagnostic:* NR	- *Sample type:* tumor<br>- *Tumor cells :* NR<br>- *Sampling method:* NR<br>- *Sample fixation:* paraffin-fixed sections<br>- *Samples storage:* NR<br>- *RNA extraction method:* NA<br>- *RNA quality assessment:* NA<br>- *cDNA synthesis method:* NA	- *Measurement method:* IHC<br>- *Antibodies/probes:* polyclonal antibody against PTHrP(34-67)<br>- *Housekeeping gene(s):* NA<br>- *Quantification methods:* NR<br>- *Positive controls:* NR<br>- *Negative/specificity controls:* NR<br>- *Reproducibility assessments:* NR<br>- *Statistical analysis:* NR<br>Chi-squared tests have been done to compare frequencies	- Mammographic calcifications (positive, negative)<br>- Histological calcifications (positive, negative)	<u>**All patients:**</u><br>- Mammographic microcalcifications<br>50% of PTHrP-positive breast cancers having mammographic microcalcifications compared to 21% of PTHrP-negative tumors<br>Chi-square p≤0.001<br><br>- Histological calcifications<br>24% of PTHrP-positive breast cancers having histological microcalcifications compared to 11% of PTHrP-negative tumors<br>Chi-square p ≤ 0.001
Downey *et al.*, 1997, UK, cross-sectional study	107	- *Period of recruitment:* NR<br>- *Age:* mean = 58.6 years range 30 to 79 years<br>- *Ethnicity:* NR<br>- *Menopausal status:*<br>9/107 8% premenopausal<br>98/107 92% postmenopausal<br>- *Parity status:* NR<br>- *Follow-up:* NR<br>- *Treatment regimen:* local excision followed by radiotherapy or mastectomy. Axillary node resection was carried out in 98/107 (92%) cases<br><br>**Calcemia:**<br>- *Status:* 107/107, 100% normocalcemic at time of surgery<br>- *Method of diagnostic:* NR	**Breast tumor:**<br>- *Stage:*<br>*Lymph node status*<br>28/107 26% positive<br>79/107 74% negative<br>- *Grade[R]:*<br>13/107 12% 1<br>63/107 59% 2<br>23/107 21% 3<br>8/107 7% missing<br>- *Histological types:*<br>91/107 85% ductal carcinoma<br>12/107 11% lobular carcinoma<br>3/107 3% mucoid carcinoma<br>1/107 1% tubular carcinoma<br>- *Molecular subtypes:*<br>ER[S]+ 75/107 70%<br>PR[S]+ 63/107 59%<br>HER2+ NR<br>Ki67[T] +<br>56/107 52% 1<br>35/107 33% 2<br>16/107 15% 3<br><br>**Metastases:**<br>- *N:* 0/107, 0% known to have bone metastases<br>- *Method of diagnostic:* NR	- *Sample type:* tumor<br>- *Tumor cells :* NR<br>- *Sampling method:* surgery<br>- *Sample fixation:* fixed in 10% v/v neutral buffered formalin and embedded in paraffin wax<br>- *Samples storage:* NR<br>- *RNA extraction method:* NA<br>- *RNA quality assessment:* NA<br>- *cDNA synthesis method:* NA	- *Measurement method:* IHC<br>- *Antibodies/probes:* antiserum raised in rabbit against PTHrP(1-34), dilution 1:800<br>- *Housekeeping gene(s):* NA<br>- *Quantification methods:* semiquantitative<br>• <u>Number of positive tumor cells:</u> 0% (0), <20% (1), 20-80% (2), >80% (3)<br>• <u>Density of stain:</u> none (0), weak (1), moderate (2), strong (3)<br>• <u>Total score (number of positive tumor cells x density of stain):</u> between 0 and 9<br>- *Positive controls:* breast cancer and skin<br>- *Negative/specificity controls:* omission of the primary antibody, non-immune rabbit serum, pre-absorption of PTHrP antibody with PTHrP at 4°C overnight<br>- *Reproducibility assessments:* staining was assessed by two independent observers, inter-observer agreement = 94%, slides were re-stained and reassessed if there was failure to achieve consensus<br>- *Statistical analysis:* categorical (positive *versus* negative) relative	- Age (years)<br>- Lymph node status (positive, negative)<br>- Grade[R] (1, 2, 3)<br>- ER[S] status (positive, negative)<br>- PR[S] status (positive, negative)<br>- Ki67 score (1, 2, 3)<br>- Tumor size (mm)	<u>**All patients:**</u><br>*No association :*<br>- Age<br>- Lymph node status<br>- Grade[R]<br>- ER[S] status<br>- PR[S] status<br>- Ki67[T] score<br>- Tumor size

					to hyperplastic breast tissue<br>Mann-Whitney U-test has been used to compare scores and chi-squared test was used to compare groups for prognostic factors		
Mawer *et al.*, 1997, UK, cross-sectional study	129	- *Period of recruitment:* NR<br>- *Age:* NR<br>- *Ethnicity:* NR<br>- *Menopausal status:* NR<br>- *Parity status:* NR<br>- *Follow-up:* NR<br>- *Treatment regimen:* No treatment or adjuvant tamoxifen. Patients with tamoxifen adjuvant therapy were changed to megesterol if disease progressed<br><br>**Calcemia:**<br>- *Status:* 12/129, 9% had bone metastases with hypercalcemia<br>- *Method of diagnostic:* hypercalcemia was defined as a serum calcium >2.6mmol/L when adjusted for serum albumin	**Breast tumor:**<br>- *Stage:*<br>88/129 68% operable early breast cancer<br>41/129 32% stage 4<br>- *Grade:* NR<br>- *Histological types:* NR<br>- *Molecular subtypes:*<br>ER+ NR<br>PR+ NR<br>HER2+ NR<br>Ki67+ NR<br><br>**Metastases:**<br>- *N:* 88/129, 68% patients had operable early breast cancers without metastases, 41/129, 32% had bone metastases<br>- *Method of diagnostic:* bone scan or plain x-rays	- *Sample type:* blood (plasma)<br>- *Tumor cells:* NR<br>- *Sampling method:* NR<br>- *Sample fixation:* NA<br>- *Samples storage:* NR<br>- *RNA extraction method:* NA<br>- *RNA quality assessment:* NA<br>- *cDNA synthesis method:* NA	- *Measurement method:* IRMA<br>- *Antibodies/probes:* targeting PTHrP(1-86)<br>- *Housekeeping gene(s):* NR<br>- *Quantification methods:* continuous, normal = limit of detection ≤0.23pmol/l<br>- *Positive controls:* NR<br>- *Negative controls:* NR<br>- *Reproducibility assessments:* NR<br>- *Statistical analysis:* continuous<br>Statistical significance of differences between groups were assessed as appropriate by Student’s paired or unpaired t test or by the Mann-Whitney or Wilcoxon tests.	- Routine prognostic markers	**All patients:**<br>*No association:*<br>- Routine prognostic markers<br><br>Plasma PTHrP was detectable, but only at very low levels in 8/88 (9%) women with early breast cancer (median 0.5pmol/L; range 0.37-0.78).
Wulf *et al.*, 1997, Germany, cohort study	34	- *Period of recruitment:* NR<br>- *Age:* median = 57 years, range 34 to 87<br>- *Ethnicity:* NR<br>- *Menopausal status:* NR<br>- *Parity status:* NR<br>- *Follow-up:* median = 11 months<br>- *Treatment regimen:* adjuvant therapy regimens<br><br>**Calcemia:**<br>- *Status:* NR<br>- *Method of diagnostic:* NR	**Breast tumor:**<br>- *Stage:*<br>10/34 29% 1<br>15/34 44% 2<br>5/34 15% 3<br>2/34 6% 4<br>2/34 6% missing<br>- *Grade:* NR<br>- *Histological types:* NR<br>- *Molecular subtypes:*<br>ER+ NR<br>PR+ NR<br>HER2+ NR<br>Ki67+ NR<br><br>**Metastases:**<br>- *N:* 2/34, 6% stage 4 patients (2/34, 6% missing)<br>- *Method of diagnostic:* NR<br>- *Receptor status:* NR	- *Sample type:* blood (plasma, *n*=30), bone marrow (*n*=34) and tumor (*n*=15)<br>- *Tumor cells :* NR<br>- *Sampling method:* all samples were collected at time of first surgery. The bone marrow was obtained by a one-sided iliac crest aspiration. The mononuclear cell fraction of 10-20ml heparinized full blood/bone marrow was separated by density centrifugation according to the method of Boyum. Mononuclear cells were washed, counted and 1 x 10^7 cells were used for RNA extraction<br>- *Sample fixation:*<br>**Blood:** NA<br>**Bone marrow:** NA<br>**Tumor:** snap frozen in liquid nitrogen, minced to a powder at -70°C	- *Measurement method:* RT-PCR<br>- *Antibodies/probes:* PTHrP upstream 1: 5’-GACTGGTTCAGCAGTGGAGC-3’<br>PTHrP downstream 1: 5’-ATCGAGCTCCAGCGACGTTGT-3’<br><br>PTHrP upstream 2: 5’-TGTTCCTGCTGAGCTACGC-3’<br>PTHrP downstream 2: 5’-TACCCCACTCCCAGTCACT-3’<br><br>PTHrP internal primer: 5’-CTCAAAAGAGCTGTGTCTGAACAT-3’<br>- *Housekeeping gene(s):* β-actin<br>- *Quantification methods:* qualitative (detection *versus* no detection)<br>- *Positive controls:* NR<br>- *Negative controls:* 30 blood samples, 25 bone marrow samples and 6 benign breast lesion samples from healthy patients<br>- *Reproducibility assessments:* NR<br>- *Statistical analysis:* qualitative (detectable, not detectable)	- Tumor size (T1/T2, T3/T4)<br>- Nodal status (positive, negative)	**All patients:**<br>Blood (*n*=30)<br>Bone marrow (*n*=34)<br>Tumor (*n*=15)<br>*No association:*<br>- Tumor size<br>- Nodal status

				- *Samples storage:*<br>**Blood:** NR<br>**Bone marrow:** NR<br>**Tumor:** stored at -70°C until RNA extraction<br>- *RNA extraction method:* total mRNA was isolated by chloroform/phenol extraction with RNAzol (Biotecx, Houston, USA) according to the manufacturer's instructions<br>- *RNA quality assessment:* NR<br>- *cDNA synthesis method:* murine leukemia virus reverse transcriptase (500U/reaction; Gibco, Eggenstein-Leopoldshafen, Germany)			
Bucht *et al.*, 1998, Sweden, cross-sectional study	48	- *Period of recruitment:* NR<br>- *Age:* 58 ± 8 years, range 29-79 years<br>- *Ethnicity:* NR<br>- *Menopausal status:* NR<br>- *Parity status:* NR<br>- *Follow-up:* NR<br>- *Treatment regimen:* NR<br><br>**Calcemia:**<br>- *Status:* 48/48, 100% normocalcemic at time of surgery<br>- *Method of diagnostic:* NR	**Breast tumor:**<br>- *Stage:*<br>Axillary node status<br>16/48 33% positive<br>25/48 52% negative<br>7/48 15% missing<br>- *Grade[K]:*<br>5/48 10% grade 1<br>11/48 23% grade 2<br>19/48 40% grade 3<br>13/48 27% missing<br>- *Histological types[T]:*<br>4/48 8% lobular<br>1/48 2% lobular/tubular<br>1/48 2% lobular/ductal<br>29/48 60% ductal<br>6/48 13% others<br>7/48 15% missing<br>- *Molecular subtypes:*<br>ER[U]+ 28/48 58% (10/48, 21% missing)<br>PR[U]+ 23/48 48% (10/48, 21% missing)<br>HER2+ NR<br>Ki67 NR<br><br>**Metastases:**<br>- *N:* NR<br>- *Method of diagnostic:* NR	- *Sample type:* blood (plasma) and tumor<br>- *Tumor cells:* NR<br>- *Sampling method:*<br>**Blood:** collected preoperatively in tubes containing protease inhibitors (PTHrP Cocktail Tubes; Nichols Institute) and centrifugation<br>**Tumor:** surgery<br>- *Sample fixation:*<br>**Blood:** NA<br>**Tumor:** fixed in 10% formalin and embedded in paraffin<br>- *Samples storage:*<br>**Blood:** aliquoted and kept frozen at -80°C<br>**Tumor:** NR<br>- *RNA extraction method:* NA<br>- *RNA quality assessment:* NA<br>- *cDNA synthesis method:* NA	- *Measurement method:*<br>**Blood:** RIA, IFMA and IRMA<br>**Tumor:** IHC<br>- *Antibodies/probes:*<br>**RIA =** mixture of two polyclonal rabbit antisera against PTHrP(63-77)<br>**IFMA =** polyclonal rabbit antiserum 1926 against PTHrP(38-67) and polyclonal sheep antiserum against PTHrP(1-34)<br>**IRMA =** two polyclonal antibodies against PTHrP(60-72) and PTHrP(1-40) (Nichols Institute)<br>**IHC =** mouse monoclonal antibody 8B12 against PTHrP(1-34) (10µg/ml)<br>- *Housekeeping gene(s):* NA<br>- *Quantification methods:*<br>**RIA =** continuous, synthetic midmolecular fragment of PTHrP(63-77) reference standard (pmol/l)<br>**IFMA =** continuous, recombinant PTHrP(1-84) reference standard<br>**IRMA =** continuous<br>**IHC =** semiquantitative, no staining in tumor cells (0), weak staining in tumor cells (+), moderate staining in tumor cells (++) and strong staining of tumor cells (+++)<br>- *Positive controls:*<br>**RIA =** synthetic midmolecular fragment of PTHrP(63-77)	**Tumor:**<br>- Tumor size (mm)<br>- Histologic type[T] (lobular, lobular/tubular, lobular/ductal, ductal, others)<br>- Grade[K] (I, II, III)<br>- Lymph node status (positive, negative)<br>- ER[U] status (positive, negative)<br>- PR[U] status (positive, negative)<br><br>**Blood:**<br>- Grade[K] (I, II, III)<br>- Histologic type[T] (lobular, lobular/tubular, lobular/ductal, ductal, others)<br>- Lymph node status (positive, negative)<br>- ER[U] status (positive, negative)<br>- PR[U] status (positive, negative)	**Tumor (*n*=42):**<br>**All patients:**<br>*No association:*<br>- Tumor size<br>- Histologic type[T]<br>- Grade[K]<br>- Lymph node status<br>- ER[U] status<br>- PR[U] status<br><br>**Blood (*n*=33-47):**<br>**All patients:**<br>*No association:*<br>- Grade[K]<br>- Histologic type[T]<br>- Lymph node status<br>- ER[U] status<br>- PR[U] status

					**IFMA =** Recombinant PTHrP(1-84)<br>**IRMA =** NR<br>**IHC =** lung cancer cell line (BEN)<br>- *Negative/specificity controls:*<br>**RIA =** NR<br>**IFMA =** blank, there was no cross-reaction with 100pmol/l of the PTHrP(1-36), PTHrP(58-77) or PTH(1-84)<br>**IRMA =** no cross-reaction with PTH(1-34), PTH(53-84), PTH(44-68) of PTH(1-84)<br>**IHC =** parallel section incubated with equivalent concentrations of preimmune rabbit IgG, preadsorbtion of PTHrP antibody with 0.5mg/ml of PTHrP(1-36) (Peninsula Laboratory, Inc., Belmont, CA) and omission of the primary antibody (8B12)<br>- *Reproducibility assessments:*<br>**RIA =** intraassay variation <9%, interassay variation <15%<br>**IFMA =** intraassay variation <9.6%, interassay variation <14.3%<br>**IRMA =** NR<br>**IHC =** NR<br>- *Statistical analysis:*<br>**RIA =** continuous<br>**IFMA =** continuous<br>**IRMA =** continuous<br>**IHC =** categorical (0, +, ++, +++)		
Iezzoni *et al.*, 1998, USA, cross-sectional study	52	- *Period of recruitment:* NR<br>- *Age:* mean = 59 years, median = 58 years, range 28-96 years<br>- *Ethnicity:* NR<br>- *Menopausal status:* NR<br>- *Parity status:* NR<br>- *Follow-up:* NR<br>- *Treatment regimen:* surgery, none of the patients had received preoperative chemotherapy<br><br>**Calcemia:**<br>- *Status*: 52/52, 100% normocalcemic at time of surgery<br>- *Method of diagnostic:* NR	**Breast tumor:**<br>- *Stage:* NR<br>- *Grade*[D]*:*<br>6/52 11% grade 1<br>17/52 33% grade 2<br>29/52 56% grade 3<br>- *Histological types:*<br>2/52 4% lobular<br>50/52 96% ductal<br>- *Molecular subtypes:*<br>ER[V]+ 32/52 62% (3/52, 6% missing)<br>PR[V]+ 18/52 35% (9/52, 17% missing)<br>HER2+ NR<br>Ki67 NR<br><br>**Metastases:**<br>- *N:* NR<br>- *Method of diagnostic:* NR	- *Sample type:* tumor<br>- *Tumor cells :* NR<br>- *Sampling method:* surgery<br>- *Sample fixation:* zinc formalin-fixed and paraffin-embedded<br>- *Samples storage:* NR<br>- *RNA extraction method:* NA<br>- *RNA quality assessment:* NA<br>- *cDNA synthesis method:* NA	- *Measurement method:* IHC<br>- *Antibodies/probes:* murine monoclonal antibody (clone 9H7) against PTHrP(109-141)<br>- *Housekeeping gene(s):* NA<br>- *Quantification methods:* staining has been evaluated in the cytoplasm of tumor cells<br>• <u>Intensity of the staining:</u> no staining in comparison with the absorbed control (0), weak staining (1), moderate staining (2) and strong staining (3)<br>• <u>Extent of the staining:</u> no staining (0), 1-30% positive tumor cells (1), 31-60% positive tumor cells (2) and 61-100% positive tumor cells (3)<br>• <u>Staining index:</u> intensity of the staining times the extent of the staining = 1 and 2 (weak), 3 and 4 (moderate) and 6 and 9 (strong)	- Grade[D] (1, 2, 3)<br>- ER[V] status (positive, negative)<br>- PR[V] status (positive, negative)	<u>**All patients:**</u><br>*No association:*<br>- Grade[D]<br>- ER[V] status<br>- PR[V] status

					- *Positive controls:* adult kidney<br>- *Negative/specificity controls:* adsorption of the primary antibody with an excess of its specific immunogenic peptide; primary antibody, secondary antibody and avidin-horseradish peroxidase sequentially replaced by diluent; replacement of the anti-PTHrP antibody by the murine monoclonal antibody against tryptophan E<br>- *Reproducibility assessments:* NR<br>- *Statistical analysis:* semiquantitative (weak, moderate, strong)<br>The Kruskal-Wallis test was used for the 3-group comparison of tumor grade versus staining index and the Wilcoxon rank-sum test was used for the 2-group comparison of the hormone receptors versus tumor staining index		
Bouizar *et al.*, 1999, France, cohort study	74	- *Period of recruitment:* NR<br>- *Age:* mean = 58 years range 38 to 79 years<br>19/74 26% ≤50 years<br>55/74 74% >50 years<br>- *Ethnicity:* NR<br>- *Menopausal status:*<br>11/74 15% premenopausal<br>63/74 85% postmenopausal<br>- *Parity status:* NR<br>- *Follow-up:* from 1 to 14 years (median = 7 years). Patients were reviewed every 3 months during the first 2 years and yearly thereafter.<br>- *Treatment regimen:* NR<br><br>**Calcemia:**<br>- *Status:* 74/74, 100% normocalcemic at diagnosis.<br>- *Method of diagnostic:* NR	**Breast tumor:**<br>- *Stage:*<br>Tumor size<br>14/74 19% ≤2cm<br>60/74 81% >2cm<br>Nodal status<br>18/74 24% 0<br>22/74 30% 1-3<br>29/74 39% >3<br>5/74 7% missing<br>- *Grade*[W]*:*<br>2/74 3% grade I<br>39/74 52% grade II<br>31/74 42% grade III<br>2/74 3% missing<br>- *Histological types:*<br>66/74 89% infiltrating ductal carcinoma<br>7/74 10% infiltrating lobular carcinoma<br>1/74 1%MC<br>- *Molecular subtypes:*<br>ER[X]+ 48/74 65%<br>PR[X]+ 29/74 39%<br>HER2+ NR<br>Ki67+ NR<br><br>**Metastases:**<br>- *N:* 0/74 0% at time of diagnosis.<br>During follow up, 18/74 (24%) did not develop metastasis while 56/74 (76%) developed metastases.<br>22/56 (39%) developed soft	- *Sample type:* tumor<br>- *Tumor cells :* NR<br>- *Sampling method:* surgery<br>- *Sample fixation:* snap frozen in liquid nitrogen immediately after surgery and selection by the pathologist<br>- *Samples storage:* liquid nitrogen tumor bank<br>- *RNA extraction method:* guanidium thiocyanate-phenol/chloroform method using the ARNZol Kit (Bioprob Systems, Clays, Sous Bois, France)<br>- *RNA quality assessment:* NR<br>- *cDNA synthesis method:* NR	- *Measurement method:* semiquantitative PCR AmpliTaq DNA polymerase (Apligene, Illkirch, France) reactions<br>- *Antibodies/probes:*<br>P1 transcripts :<br>F=5’GCAGCTTGGAAGAGGTAC3’<br>R=5’GCGGAGGAATGTTCACAC3’<br>P2 transcripts :<br>F=5’GTGTGAACATTCCTCCG3’<br>R=5’GTAGCTCAGCAGGAACAC3’<br>P3 transcripts :<br>F=5’CTGCTGGCCAGATTAATTAG3’<br>R=5’GTAGCTCAGCAGGAACAC3’<br>PTHrP 139 :<br>F=5’GCGACGATTCTTCCTTCACC3’<br>R=5’AGAGTCTAACCAGGCAGAGC3’<br>PTHrP 173 :<br>F=5’GCGACGATTCTTCCTTCACC3’<br>R=5’TGATGTGTTCTTCTGTTGTT3’<br>PTHrP 173 :<br>F=5’GCGACGATTCTTCCTTCACC3’<br>R=5’GATAGGTCATTCACTGTGCTC3’<br>PTHrP 141 :	- Grade[W] (II *versus*, I and III)<br>- Menopausal status (premenopausal, postmenopausal)<br>- ER[X] status<br>- PR[X] status	*Association:*<br>- Grade[W]<br>PTHrP(139)/S14 was higher in grade II (0.30 ± 0.04) compared to grades I (0.04 ± 0.04) and III (0.19 ± 0.02)<br>p<0.05<br>- Menopausal status<br>PTHrP(141)/S14 was higher in premenopausal (0.29 ± 0.07) than in postmenopausal women (0.15 ± 0.03)<br>p<0.05<br><br>- ER[X] status<br>P2-initiated PTHrP Was lower in ER+ tumors (0.10 ± 0.02) compared to ER- tumors (0.23 ± 0.05)<br>p<0.009<br><br>*No association:*<br>PTHrP(139)/S14<br>- Menopausal status<br>- ER[X] status<br>- PR[X] status<br><br>PTHrP(141)/S14<br>- Grade[W]<br>- ER[X] status<br>- PR[X] status

			tissue metastases and 34/56 (61%) developed bone metastases<br>- *Method of diagnostic:* bone scintigraphy and conventional radiography		F=5'GCGACGATTCTTCCTTCACC3'<br>R=5'CCTTGGAAGGTCTCTGCTGA3'<br>- *Housekeeping gene(s):* human small ribosomal protein 14 (S14)<br>- *Quantification methods:* quantitative, relative to S14<br>- *Positive controls:* TT cells (thyroid carcinoma) and MCF7 cells (breast carcinoma)<br>- *Negative/specificity controls:* omission of the Moloney murine leukemia virus reverse transcriptase or digestion of the samples with RNAse A<br>- *Reproducibility assessments:* internal control<br>- *Statistical analysis:* qualitative (present, absent) semiquntitative (ratio between PTHrP and S14) Data were analyzed by Super-analysis of variance followed by Fisher's protected least significant difference test or Scheffe's *S*-test. The distribution frequency was compared using contingence analysis.		PTHrP(173)/S14<br>No expression detected<br><br>P2-initiated PTHrP<br>PR[X] status
Sugimoto *et al.*, 1999, Japan, cross-sectional study	35	- *Period of recruitment:* NR<br>- *Age:* range 32 to 70 years<br>- *Ethnicity:* NR<br>- *Menopausal status:*<br>17/35 49% premenopausal<br>18/35 51% postmenopausal<br>- *Parity status:* NR<br>- *Follow-up:* NR<br>- *Treatment regimen:* surgery, no endocrine therapy was performed before surgery<br><br>**Calcemia:**<br>- *Status:* NR<br>- *Method of diagnostic:* NR	**Breast tumor:**<br>- *Stage:*<br>*Tumor size*<br>3/35 9% <2 cm<br>24/35 69% 2-5 cm<br>5/35 14% >5 cm<br>3/35 9% missing<br>*Nodal status*<br>19/35 54% +<br>16/35 46% -<br>- *Grade:* NR<br>- *Histological types:*<br>14/35 40% papillotubular carcinoma<br>4/35 11% solid-tubular carcinoma<br>14/35 40% scirrhous carcinoma<br>2/35 6% medullary carcinoma<br>1/35 3% mucinous carcinoma<br>- *Molecular subtypes:*<br>ER[Y]+ 23/35 66%<br>PR+ NR<br>HER2+ NR<br>Ki67+ NR<br><br>**Metastases:**<br>- *N:* NR	- *Sample type:* tumor<br>- *Tumor cells :* NR<br>- *Sampling method:* resection surgery<br>- *Sample fixation:* NR<br>- *Samples storage:* NR<br>- *RNA extraction method:* 0.8ml Trizol reagent (Life Technologies, Gaithersburg, MD)<br>- *RNA quality assessment:* the concentration of recovered RNA was determined spectrophotometrically<br>- *cDNA synthesis method:* 800 units of M-MLV reverse transcriptase (Bethesda Res. Lab., Gaithersburg, MD)	- *Measurement method:* coamplification PCR of PTHrP with β-actin<br>- *Antibodies/probes:*<br>F = 5'ATGCAGCGGAGACTGGTTCAGCAGT3'<br>R = 5'GGGAGAGGGCTTGGAGTTAGGGG3'<br>- *Housekeeping gene(s):* β-actin<br>- *Quantification methods:* relative to β-actin, SYBR Green I (Takara Shuzo, Kyoto, Japan) fluorescence intensity was measured on gels<br>- *Positive controls:* NR<br>- *Negative controls:* NR<br>- *Reproducibility assessments:* NR<br>- *Statistical analysis:* continuous Statistical significance was calculated by Student's *t* test	- Menopausal status (premenopausal, postmenopausal)<br>- Tumor size (<2 cm, 2-5 cm, >5 cm)<br>- Nodal status (+, -)<br>- Histopathological subtypes (papillotubular carcinoma, solid-tubular carcinoma, scirrhous carcinoma, medullary carcinoma, mucinous carcinoma)<br>- ER[Y] status	**All patients:**<br>*Association:*<br>- premenopausal status<br>$p < 0.05$<br><br>*No association:*<br>- ER[Y] status<br>- Tumor size<br>- Nodal status<br>- Histopathological subtypes<br><br>**Subgroup:**<br>*Association:*<br>In ER+ tumors, PTHrP expression was higher in premenopausal women than in postmenopausal women<br>$p < 0.001$<br><br>*No association:*<br>In ER- tumors, PTHrP expression was similar between pre- and postmenopausal women

			- *Method of diagnostic:* NA				
Yoshida *et al.*, 2000, Japan, cohort study	177	- *Period of recruitment:* 1990 to 1996<br>- *Age:* mean = 53.4 (range 27 to 87 years)<br>80/177 45% ≤50 years<br>97/177 55% >50 years<br>- *Ethnicity:* NR<br>- *Menopausal status:* NR<br>- *Parity status:* NR<br>- *Follow-up:* mean = 6.1 years<br>- *Treatment regimen:* NR<br><br>**Calcemia:**<br>- *Status:* 0/177 (0%) of the cases exhibited humoral hypercalcemia.<br>- *Method of diagnostic:* NR	**Breast tumor:**<br>- *Stage:*<br>109/177 62% stage 1<br>29/177 16% stage 2<br>23/177 13% stage 3<br>6/177 3% stage 4<br>10/177 6% unknown<br>- *Grade[D]:*<br>60/177 34% grade I<br>65/177 37% grade II<br>52/177 29% grade III<br>- *Histological types:* carcinoma<br>- *Molecular subtypes:*<br>ER[V]+ 89/177, 50%<br>PR+ NR<br>HER2+ NR<br>Ki67+ NR<br><br>**Metastases:**<br>- *N:* 6/177 (3%) with bone metastases at time of diagnosis (10/177, 6% missing)<br>23/177 (13%) developed bone metastases during the follow-up period, and 22/177 (12%) developed metastases to other tissue than bone<br>126/177 (71%) patients did not develop metastasis during the follow-up period<br>- *Method of diagnostic:* scintigraphy and plain X-ray	- *Sample type:* tumor<br>- *Tumor cells :* NR<br>- *Sampling method:* surgical resection of the tumors<br>- *Sample fixation:* fixed in neutral formalin for 24h and paraffin-embedded<br>- *Samples storage:* NR<br>- *RNA extraction method:* NA<br>- *RNA quality assessment:* NA<br>- *cDNA synthesis method:* NA	- *Measurement method:* IHC<br>- *Antibodies/probes:* monoclonal antibody (Oncogene Science, Inc., Uniondale, NY, USA)<br>- *Housekeeping gene(s):* NA<br>- *Quantification methods:*<br>• <u>Intensity of cytoplasmic staining</u>: no positive staining of tumor cells (0), weak positive staining of tumor cells (1), strong positive staining of tumor cells (2)<br>• <u>Percentage of stained cells:</u> no positive tumor cells (0), 1-20% of the tumor cells were positive (1), >20% of the tumor cells were positive (2)<br>• <u>Degree of staining;</u> determined as the sum of the staining intensity and percentage of cells stained, 0-1 (negative, -), 2-3 (weakly positive, +), 4 (strongly positive, ++)<br>- *Positive controls:* NR<br>- *Negative/specificity controls:* primary antibody replaced by non-immunized mouse serum<br>- *Reproducibility assessments:* NR<br>- *Statistical analysis:* semiquantitative (-, +, ++) and qualitative (negative (-), positive (+ and ++))<br>The chi-squared test was performed to evaluate the relationship between PTHrP expression and clinicopathological features	- Age (≤ 50 or > 50)<br>- Tumor size (≤ 2.5 cm or > 2.5 cm)<br>- Nodal status (- or +)<br>- Distant metastases (presence, absence)<br>- Grade[D] (I, II or III)<br>- ER[V] status (- or +)	<u>**All patients:**</u><br>*Association (positive):*<br>- grade[D]<br>Chi-squared p = 0.002<br><br>*No association:*<br>- Age<br>- Tumor size<br>- Nodal status<br>- Distant metastases<br>- ER[V] status
Henderson *et al.*, 2006, Australia, cohort study	526	- *Period of recruitment:* between December 1, 1989 and December 31, 1996<br>- *Age:* median = 60 years (range 27 to 93)<br>- *Ethnicity:* NR<br>- *Menopausal status:* 429/526 82% postmenopausal<br>97/526 18% premenopausal<br>- *Parity status:* NR<br>- *Follow-up:* minimum of 8 years, median = 10 years, patients were reviewed at least twice yearly for the first 3 years and then,	- *Stage[A]:*<br>183/526 35% stage 1<br>161/526 31% stage 2a<br>71/526 13% stage 2b<br>77/526 15% stage 3a<br>34/526 6% stage 3b-c<br>- *Grade[Z]:*<br>66/526 13% grade I<br>270/526 51% grade II<br>190/526 36% grade III<br>- *Histological types:*<br>458/526 87% ductal<br>35/526 7% lobular<br>31/526 6% other<br>- *Molecular subtypes:*<br>ER[AA]+ 350/526 67% (11/526, 2% missing)<br>PR[AA]+ 312/526 59% (35/526, 7% missing)	- *Sample type:* tumor<br>- *Tumor cells :* NR<br>- *Sampling method:* surgery<br>- *Sample fixation:* NR<br>- *Samples storage :* NR<br>- *RNA extraction method:* NA<br>- *RNA quality assessment:* NA<br>- *cDNA synthesis method:* NA	- *Measurement method:* IHC<br>- *Antibody:* polyclonal rabbit anti-PTHrP(1-14)<br>- *Housekeeping gene(s):* NA<br>- *Quantification methods:* tumors were called positive when at least 1 tumor cell was stained for PTHrP<br>- *Positive controls:* normal skin<br>- *Negative/specificity controls:* nonimmune control, no cross-reactivity with PTH was observed<br>- *Reproducibility assessments:* 8% samples were re-stained and evaluated (100% concordance), each tumor section was stained in duplicate, with two dilutions of antiserum, and was assessed by a panel of individuals who were unaware of the clinical details	- Age (years)<br>- Tumor size (mm)<br>- Tumor size (T_{1a} + T_{1b}, T_{1c}, T_2, T_3/T_4)<br>- Lymph node status (N_0, N_1, N_2, N_3)<br>- Stage[A] (I, IIa, IIb, IIIa, IIIb + IIIc)<br>- Menopausal status (premenopausal, postmenopausal)<br>- ER[AA] status (positive, negative)<br>- PR[AA] status (positive, negative)<br>- Grade[Z] (I, II, III)<br>- Lymphatic,	<u>**All patients:**</u><br>*Association:*<br>- Tumor size (mm)<br>PTHrP-negative tumors (median = 31mm) were bigger than PTHrP-positive tumors (median = 27mm)<br>Mann-Whitney U test p = 0.007<br>- Tumor size (categorical)<br>PTHrP-negative tumors were bigger than PTHrP-positive tumors<br>Fisher's exact test p = 0.03<br>- Stage[A]<br>PTHrP-negative tumors

		annually.<br>- *Treatment regimen:*<br>28/526 5% preoperative chemotherapy<br>0/526 (0%) patient received preoperative systemic therapy<br><br>264/526 50% mastectomy<br>226/526 43% breast conservative surgery<br>36/526 7% underwent lesser procedures because of morbidities or patient preference<br><br>216/526 41% postoperative radiotherapy<br>184/526 35% postoperative chemotherapy<br>295/526 56% Tamoxifen for 5 years<br><br>**Calcemia:**<br>- *Status:* NR<br>- *Method of diagnostic:* NR	HER2+ NR<br>Ki67 NR<br><br>**Metastases:**<br>- *N:* 0/526, 0% at time of diagnosis<br>- *Method of diagnostic:* routine scans and blood tests		- *Statistical analysis:* qualitative (positive, negative)<br>Frequency data were assessed by standard contingency table analysis, including Fisher's exact test for binary or categorical variables and by the Mann-Whitney U test for continuous variables	vascular invasion (positive, negative)<br>- Tumor type (invasive ductal, lobular, special types)	tend to be more advanced than PTHrP-positive tumor<br>Fisher's exact test p = 0.01<br>- Menopausal status<br>Patients with PTHrP-positive tumors were more likely to be postmenopausal (349/415, 84%) compared to patients with PTHrP-negative tumors (80/111, 72%)<br>Fisher's exact test p = 0.006<br>- ER[AA] status<br>PTHrP-positive tumors were more likely to be ER positive<br>Fisher's exact test p = 0.01<br>- PR[AA] status<br>PTHrP-positive tumors were more likely to be PR positive<br>Fisher's exact test p = 0.03<br><br>*No association:*<br>- Age<br>- Lymph node status<br>- Grade[Z]<br>- Lymphatic, vascular invasion<br>- Tumor type
Zia *et al.*, 2007, UK, cohort study	124	- *Age:* NR<br>- *Ethnicity:* NR<br>- *Menopausal status:* NR<br>- *Parity status:* NR<br>- *Follow-up:* median = 10 years<br>- *Treatment regimen:* NR<br><br>**Calcemia:**<br>- *Status:* NR<br>- *Method of diagnostic:* NR	- *Stage:*<br>70/124 56% stage 1<br>40/124 32% stage 2<br>7/124 6% stage 3<br>4/124 3% stage 4<br>3/124 2% missing<br>- *Grade:*<br>24/124 19% grade I<br>42/124 34% grade II<br>58/124 47% grade III<br>- *Histological types:*<br>94/124 76% ductal<br>14/124 11% lobular<br>16/124 13% other<br>- *Molecular subtypes:*<br>ER+ NR<br>PR+ NR<br>HER2+ NR<br>Ki67 NR<br><br>**Metastases:**<br>- *N:* 4/124, 3% at time of	- *Sample type:* tumor<br>- *Tumor cells :* NR<br>- *Sampling method:* NR<br>- *Sample fixation:* fresh frozen in liquid nitrogen<br>- *Samples storage :* NR<br>- *RNA extraction method:* RNAzol procedure<br>- *RNA quality assessment:* spectrophotometry<br>- *cDNA synthesis method:* AB Gene Reverse Transcription System, Surrey, UK	- *Measurement method:* qPCR (AmplofluorTM UniprimerTM probe system, Intergen Company Oxford, UK)<br>- *Probe(s):* F: GGTGTTCCTGCTGAGCTAC<br>R: ACTGAACCTGACCGTACACGTAAATCTTGGATGGACTT<br>- *Housekeeping gene(s):* β-actin<br>- *Quantification methods:* purified plasmid internal standards<br>- *Positive controls:* NR<br>- *Negative controls:* NR<br>- *Reproducibility assessments:* qPCRs have been performed on duplicates<br>- *Statistical analysis:* continuous (mean No. of copies per 50 ng RNA)	- Nodal involvement (+ or -)<br>- Grade (I, II, III)<br>- Nottingham prognostic index (1, 2, 3)	**<u>All patients:</u>**<br>*No association:*<br>- Nodal involvement<br>- Grade<br>- Nottingham prognostic index

			diagnosis, 3/124 (2%) missing<br>7/124 (6%) developed distant metastases during the follow-up<br>- *Method of diagnostic:* NR				
Fleming *et al.*, 2009, Australia, cross-sectional study (the original cohort from the Cooperative Breast Cancer Tissue Resource was however, a cohort study)	237	- *Period of recruitment:* 212/237, 89% samples from the Cooperative Breast Cancer Tissue Resource were isolated from a cohort of 9308 cases diagnosed between 1974 and 1997<br>- *Age:* NR<br>- *Ethnicity:* NR<br>- *Menopausal status:* NR<br>- *Parity status:* NR<br>- *Follow-up:* NR<br>- *Treatment regimen:* NR<br><br>**Calcemia:**<br>- *Status:* NR<br>- *Method of diagnostic:* NR	**Breast tumor:**<br>- *Stage:* NR<br>- *Grade:* NR<br>- *Histological types:* 192/237 81% invasive breast cancers<br>45/237 19% ductal carcinoma *in situ*<br>- *Molecular subtypes:*<br>ER+ NR<br>PR+ NR<br>HER2+<br>*Ductal carcinoma in situ:* 20/45, 44%<br>*Invasive breast cancers:* 28/192, 15%<br>Ki67+ NR<br><br>**Metastases:**<br>- *N:* NR<br>- *Method of diagnostic:* NR	- *Sample type:* tumor<br>- *Tumor cells :* 212/237 89% samples from the Cooperative Breast Cancer Tissue Resource were reviewed by pathologists<br>- *Sampling method:* NR<br>- *Sample fixation:* NR<br>- *Samples storage:* NR<br>- *RNA extraction method:* NA<br>- *RNA quality assessment:* NA<br>- *cDNA synthesis method:* NA	- *Measurement method:* IHC<br>- *Antibodies/probes:* goat polyclonal antibody anti-PTHrP(1-14)<br>- *Housekeeping gene(s):* NA<br>- *Quantification methods:* semiquantitative, based on staining intensity (score of 0, 1, 2, 3)<br>- *Positive controls:* NR<br>- *Negative controls:* NR<br>- *Reproducibility assessments:* NR<br>- *Statistical analysis:* semiquantitative (0, 1, 2, 3) and qualitative<br>The chi-squared test has been used to compare frequencies	- HER2 status (+,-)<br>- Monocyte infiltration (CD38 staining, 0, 1, 2, 3)	<u>**Subgroup analysis**</u><br>In ductal carcinomas *in situ*<br>*Association:*<br>- HER2 status<br>The intensity of PTHrP staining correlated with HER2 expression<br>Chi-square p = 0.013<br>- Monocyte infiltration<br>The intensity of PTHrP staining correlated with the extent of monocyte infiltration<br>Chi-square p = 0.032<br><br>In invasive breast cancers<br>*No association:*<br>- HER2 status<br>- Monocyte infiltration
Takagaki *et al.*, 2012, Japan, cohort study	125	- *Period of recruitment:* 1996 to 1999<br>- *Age:* mean = 57.4, range 31 to 88 years<br>- *Ethnicity:* NR<br>- *Menopausal status:*<br>35/125 28% premenopausal<br>90/125 72% postmenopausal<br>- *Parity status:* NR<br>- *Follow-up:* median = 97 months, range = 5 to 243 months<br>- *Treatment regimen:* patients who received either preoperative chemotherapy or endocrine therapy were excluded, then, patients have been treated with surgery and postoperative adjuvant therapy including chemotherapy, hormonal therapy and/or radiation therapy according to the status of the disease and the condition of the patient. No patient had received	**Breast tumor:**<br>- *Stage:*<br>Tumor size<br>34/125 27% T1<br>63/125 50% T2<br>14/125 11% T3<br>11% T4<br>Nodal status<br>55/125 44% -<br>70/125 56% +<br>Metastasis<br>116/125 93% M0<br>9/125 7% M1<br>- *Grade:* NR<br>- *Histological types:* NR<br>- *Molecular subtypes:*<br>ER+ 61/125 49%<br>PR+ NR<br>HER2+ NR<br>Ki67 NR<br><br>**Metastases:**<br>- *N:* 9/125 % had distant metastases at time of surgery<br>20/125 (16%) developed bone metastases, 9/125 (7%) developed local recurrence and 25/125 (20%) succumbed during follow-up<br>- *Method of diagnostic:* ultrasonography for local	- *Sample type:* tumor<br>- *Tumor cells :* NR<br>- *Sampling method:* surgery<br>- *Sample fixation:* formalin-fixed, paraffin-embedded<br>- *Samples storage:* NR<br>- *RNA extraction method:* NA<br>- *RNA quality assessment:* NA<br>- *cDNA synthesis method:* NA	- *Measurement method:* IHC<br>- *Antibodies/probes:* mouse monoclonal anti-PTHrP (100μg/ml; Oncogene Science Inc., Uniondale, NY, USA)<br>- *Housekeeping gene(s):* NA<br>- *Quantification methods:* positive when >10% of the tumor cells were positive<br>- *Positive controls:* NR<br>- *Negative controls:* normal mouse IgG was substituted for the primary antibody<br>- *Reproducibility assessments:* staining was assessed by two investigators without knowledge of the clinical outcome of the patient independently (inter-reader variability NR)<br>- *Statistical analysis:* qualitative (positive, negative)<br>Mann-Whitney's *U* test was used to define statistical significance	- Menopausal status (premenopausal, postmenopausal)<br>- Tumor size (T1, T2, T3, T4)<br>- Nodal status (-, +)<br>- Distant metastasis (M1, M0)<br>- ER status (-, +)<br>- Lymphatic infiltration (-, +)<br>- Vascular infiltration (-, +)	<u>**All patients:**</u><br>*Association:*<br>- Menopausal status<br>PTHrP was more frequently expressed in the tumors of premenopausal women (28/35, 80%) than postmenopausal women (51/90, 57%)<br>Mann-Whitney's U test p = 0.015<br>- Distant metastasis<br>Primary tumors from patients with distant metastasis were more frequently PTHrP-positive (9/9, 100%) than primary tumors from patients without distant metastases (70/116, 60%)<br>Mann-Whitney's U test p = 0.044<br><br>There were 3 cases that have bone metastases at time of surgery, 100% of primary lesions were PTHrP-positive

		elective administration of bisphosphonates<br><br>**Calcemia:**<br>- *Status:* NR<br>- *Method of diagnostic:* NR	recurrence, compute tomography for lung and liver metastases, annual scintigraphy for bone metastases and physical examination every 6 months				*No association:*<br>- Tumor size<br>- Nodal status<br>- ER status<br>- Lymphatic infiltration<br>- Vascular infiltration
Skondra *et al.* 2014, Greece, cross-sectional study	54	- *Period of recruitment:* NR<br>- *Age:* mean = 53 years (range 30-76)<br>22/54 41% <57 years<br>30/54 56% ≥57 years<br>2/54 4% missing<br>- *Ethnicity:* NR<br>- *Menopausal status:* NR<br>- *Parity status:* NR<br>- *Follow-up:* NR<br>- *Treatment regimen:* NR<br><br>**Calcemia:**<br>- *Status:* NR<br>- *Method of diagnostic:* NR	- *Stage:*<br>3/54 6% stage 1<br>28/54 52% stage 2<br>6/54 11% stage 3<br>16/54 30% stage 4<br>1/54 2% missing<br>- *Grade:*<br>12/54 22% grade I-II<br>35/54 65% grade III<br>7/54 13% missing<br>- *Histological types:*<br>40/54 74% ductal<br>8/54 15% lobular<br>4/54 7% others<br>2/54 4% missing<br>- *Molecular subtypes:*<br>ER+ : 32/54 59% (2/54, 4% missing)<br>PR+ : 19/54 35% (2/54, 4% missing)<br>HER2+ : 22/54 41% (2/54, 4% missing)<br>Ki67: 19/54 35% low (21/54, 39% missing)<br><br>**Metastases:**<br>- *N:* 16/54 30% had distant metastases at time of diagnosis<br>- *Method of diagnostic:* NR	- *Sample type:* blood<br>- *Tumor cells:* NA<br>- *Sampling method:* 6ml of blood were collected using a venous catheter into 3ml EDTA-containing vacutainers after discarding the first 2ml of blood to avoid possible contamination with epidermal cells<br>- *Sample storage:* samples were processed within 6h of collection<br>- *RNA extraction method:* TriReagent RT-118<br>- *RNA quality assessment:* ultra-violet spectrophotometry<br>- *cDNA synthesis method:* Moloney Murine Leukemia Virus (M-MLV) Reverse Transcriptase (Invitrogen)	- *Measurement method:* multiplex RT-PCR (Qiagen Multiplex PCR Kit, Qiagen, Hilden, Germany)<br>- *Probe(s):* F: CTGGTTCAGCAGTGGAGC<br>R: TTCTGCGATCAGATGGTG<br>- *Housekeeping gene(s): GAPDH*<br>- *Quantification methods:* positive or negative PTHrP detection<br>- *Positive controls:* spiking experiments of the MCF-7 in peripheral blood of healthy male blood samples<br>- *Negative controls:* peripheral blood of healthy blood samples<br>- *Reproducibility assessments:* NR<br>- *Statistical analysis:* qualitative (positive or negative)<br>Comparison between subgroups based on demographic, clinical and pathological characteristics for the expression of PTHrP was performed with the chi-squared test and Fisher's exact test. For comparisons of more than 2 groups, the Kruskal-Wallis test was performed	- Age (≤57 and >58)<br>- Histological type (ductal, lobular)<br>- Maximum tumor size (≤3cm, >3cm)<br>- Grade (low, high)<br>- Lympho-vascular invasion (presence of absence)<br>- ER status (+, -)<br>- PR status (+, -)<br>- HER2 status (+, -)<br>- Ki67 status (low, high)<br>- Tumor size (T1, T2, T3)<br>- Regional lymph nodes (N0, N1, N2, N3)<br>- Distant metastasis (M0. M1)<br>- Stage (1, 2, 3, 4)	<u>**All patients:**</u><br>*Association:*<br>*Positive PTHrP:*<br>- Stage<br>Stage 3 and 4 OR = 4.941, CI = 1.204-20.283<br>p = 0.02<br>Advanced stage disease was correlated with positive detection of PTHrP.<br><br>*Positive PTHrP + KRT19:*<br>- Distant metastasis<br>M1 OR = 4.444, CI = 1.078-18.321 p = 0.031<br>Breast cancer patients with metastatic disease have 4.4 times increased probability to have positive detection of PTHrP + KRT19 in blood.<br>- Stage<br>Stage 3 and 4 OR = 4.431, CI = 1.293-15.186<br>p = 0.0150<br>Advanced stage disease was correlated with positive detection of PTHrP + KRT19<br><br>*No association:*<br>- Age<br>- Histological type<br>- Maximum tumor size<br>- Grade<br>- Lympho-vascular invasion<br>- ER status<br>- PR status<br>- HER2 status<br>- Ki67 status<br>- Tumor size<br>- Regional lymph nodes
Xu *et al.*, 2015, China, cohort study	497	- *Period of recruitment:* January 2006 to December 2009	**Breast tumor:**<br>- *Stage*[A]:<br>195/497 39% 1	- *Sample type:* tumor<br>- *Tumor cells :* NR<br>- *Sampling method:*	- *Measurement method:* IHC<br>- *Antibodies/probes:* monoclonal antibody against PTHrP	- Age (≤55 years, >55 years)<br>- Tumor size	<u>**All patients:**</u><br>*Association:*<br>- Tumor size >2cm

		- *Age:* median = 56.7 years range 26 to 95 years<br>267/497 54% ≤55 years<br>230/497 46% >55 years<br>- *Ethnicity:* NR<br>- *Menopausal status:* NR<br>- *Parity status:* NR<br>- *Follow-up:* median = 48 months range 2 to 85 months, 108/497 22% patients lost during the process<br>- *Treatment regimen:* surgery and standardized adjuvant therapy.<br>Patients on neoadjuvant chemotherapy, as well as those with positive margins on histopathology were excluded.<br><br>**Calcemia:**<br>- *Status:* NR<br>- *Method of diagnostic:* NR	210/497 42% 2<br>92/497 19% 3<br>- *Grade:*<br>352/497 71% ≤II<br>133/497 27% >II<br>12/497 2% missing<br>- *Histological types:*<br>423/497 85% infiltrating ductal carcinoma<br>74/497 15% other<br>- *Molecular subtypes:*<br>ER+ 341/497 69% (22/497 4% missing)<br>PR+ NR<br>HER2+ 265/497 53% (55/497 11% missing)<br>Ki67+ NR<br><br>**Metastases:**<br>- *N:* 0/497, 0% at time of surgery.<br>After surgery, 116/497 (23%) patients suffered from local recurrence or distant metastasis. By the end of follow-up, 26/497 (5%) patients had died, 21/497 (4%) of breast cancer and 273/497 (55%) patients had developed no recurrence.<br>- *Method of diagnostic:* local or regional recurrence was confirmed by histology and the distant metastasis was detected by biopsy or imaging techniques.	surgery<br>- *Sample fixation:* immediately fixed in formalin following surgery, dehydrated and embedded in paraffin<br>- *Samples storage:* NR<br>- *RNA extraction method:* NA<br>- *RNA quality assessment:* NA<br>- *cDNA synthesis method:* NA	(ABGENAT, 1:2000 dilution)<br>- *Housekeeping gene(s):* NA<br>- *Quantification methods:* cytoplasmic staining was evaluated in a qualitative way (positive, negative)<br>- *Positive controls:* human placenta sections<br>- *Negative controls:* omission of the primary antibody<br>- *Reproducibility assessments:* staining has been assessed by 2 independent pathologists in a blinded manner, discrepancies were solved by re-examination and consensus<br>- *Statistical analysis:* qualitative (positive, negative)<br>The correlation between PTHrP expression and the relevant clinicopathologic features were analyzed by Pearson's chi-squared correlation test	(≤2cm, >2cm)<br>- Skin involvement (no, yes)<br>- Lymph node metastasis (no, yes)<br>- Grade (≤II, >II)<br>- Stage[A] (I, II, III)<br>- ER status (-, +)<br>- HER2 status (-, +)<br>- Tumor type (infiltrating ductal carcinoma, non-infiltrating ductal carcinoma)	PTHrP-positive tumors were more frequently >2cm compared to PTHrP-negative<br>Chi-square = 11.372<br>p = 0.001<br>- Presence of lymph node metastases<br>Chi-square = 5.014<br>p = 0.025<br>PTHrP-positive tumors were more frequently lymph nodes positive compared to PTHrP-negative<br>- Later clinical stage[A]<br>Chi-square = 6.937<br>p = 0.031<br>PTHrP-positive tumors had a more advanced stage compared to PTHrP-negative<br><br>*No association:*<br>- Age<br>- Skin involvement<br>- Grade<br>- ER status<br>- HER2 status<br>- Tumor type
Tran *et al.*, 2018, USA, cohort study<br><br>Cohort 1 (training set)	410 [AB]	- *Period of recruitment:* between 1962 and 1982<br>- *Age:* mean = 58.1 (range 30 to 88)<br>- *Ethnicity:*<br>396/410 97% white<br>12/410 3% black<br>1/410 0% Asian<br>1/410 0% Other<br>- *Menopausal status:*<br>302/410 74% post-menopausal<br>108/410 26% pre-menopausal<br>- *Parity status:* NR<br>- *Follow-up:* median disease-free follow-up 8.8 years<br>- *Treatment regimen:* NR<br><br>**Calcemia:**<br>- *Status:* NR	- *Stage:*<br>Tumor size (cm)<br>16/410 4% ≤0.5<br>59/410 14% >0.5-1<br>108/410 26% >1-2<br>159/410 39% >2-5<br>39/410 10% >5<br>29/410 7% missing<br>Nodal status<br>222/410 54% positive<br>188/410 46% negative<br>- *Grade:*<br>69/410 17% grade I<br>212/410 52% grade II<br>108/410 26% grade III<br>21/410 5% missing<br>- *Histological types:* NR<br>- *Molecular subtypes:*<br>ER+ 222/410 54% (9/410, 2% missing)<br>PR+ 211/410 51% (23/410, 6% missing)	- *Sample type:* tumor<br>- *Tumor cells :* NR<br>- *Sampling method:* NR<br>- *Sample fixation:* formalin-fixed, paraffin-embedded<br>- *Samples storage:* Yale University pathology archives<br>- *RNA extraction method:* NA<br>- *RNA quality assessment:* NA<br>- *cDNA synthesis method:* NA	- *Measurement method:* immunofluorescence-based IHC<br>- *Antibodies:* Santa Cruz, H137, PTHrP(41-177), 1:200 dilution<br>- *Quantification methods:* nuclear PTHrP was considered, AQUA scores (mean signal intensity)<br>- *Positive controls:* NR<br>- *Negative controls:* NR<br>- *Reproducibility assessments:* NR<br>- *Statistical analysis:* continuous	- Grade (I, II, III)<br>- ER status (+, -)	**All patients:**<br>*Association:*<br>- Grade<br>Nuclear PTHrP expression was higher in well-differentiated low-grade (grade 1) tumors compared to grade 3<br>p<0.01<br><br>- ER status<br>Nuclear PTHrP expression was higher in ER-positive breast cancer compared to ER-negative breast cancer<br>p<0.02

		- *Method of diagnostic:* NR	HER2+ 43/410 10% (17/410, 4% missing)<br>Ki67+ NR<br><br>**Metastases:**<br>- *N:* NR<br>- *Method of diagnostic:* NR				
Cohort 2 (validation set)	387 [AC]	- *Period of recruitment:* between 1988 and 2000<br>- *Age:* mean = 59.3 (range 29 to 90)<br>- *Ethnicity:*<br>330/387 85% White<br>46/387 12% Black<br>6/387 2% Asian<br>1/387 0% Other<br>4/387 1% missing<br>- *Menopausal status:*<br>286/387 74% post-menopausal<br>101/387 26% pre-menopausal<br>- *Parity status:* NR<br>- *Follow-up:* median recurrence-free follow-up 7.5 years<br>- *Treatment regimen:* NR<br><br>**Calcemia:**<br>- *Status:* NR<br>- *Method of diagnostic:* NR	- *Stage:*<br>Tumor size (cm)<br>12/387 3% ≤0.5<br>43/387 11% >0.5-1<br>99/387 26% >1-2<br>75/387 19% >2-5<br>19/387 5% >5<br>139/387 36% missing<br>Nodal status<br>115/387 30% positive<br>153/387 40% negative<br>119/387 31% missing<br>- *Grade:*<br>71/387 18% grade I<br>245/387 63% grade II<br>63/387 16% grade III<br>8/387 2% missing<br>- *Histological types:* NR<br>- *Molecular subtypes:*<br>ER+ 222/387 57% (99/387, 26% missing)<br>PR+ 171/387 44% (101/387, 26% missing)<br>HER2+ 25/387 7% (140/387, 36% missing)<br>Ki67+ NR<br><br>**Metastases:**<br>- *N:* NR<br>- *Method of diagnostic:* NR	- *Sample type:* tumor<br>- *Tumor cells :* NR<br>- *Sampling method:* NR<br>- *Sample fixation:* formalin-fixed, paraffin-embedded<br>- *Samples storage :* NR<br>- *RNA extraction method:* NA<br>- *RNA quality assessment:* NA<br>- *cDNA synthesis method:* NA	- *Measurement method:* immunofluorescence-based IHC<br>- *Antibodies:* Santa Cruz, H137, PTHrP(41-177), 1:200<br>- *Quantification methods:* AQUA scores (mean signal intensity)<br>- *Positive controls:* NR<br>- *Negative controls:* NR<br>- *Reproducibility assessments:* NR<br>- *Statistical analysis:* continuous	- Grade (I, II, III)<br>- ER status (+, -)	**<u>All patients:</u>**<br>*Association:*<br>- Grade<br>Nuclear PTHrP expression was higher in well-differentiated low-grade (grade 1) tumors compared to grade 3 p<0.01<br><br>- ER status<br>Nuclear PTHrP expression was higher in ER-positive breast cancer compared to ER-negative breast cancer p<0.02
Cohort 3	737 [AD]	- *Period of recruitment:* NR<br>- *Age (n=1881):* median = 55 ± 13 years<br>- *Ethnicity:* NR<br>- *Menopausal status:* NR<br>- *Parity status:* NR<br>- *Follow-up:* censored at 10 years<br>- *Treatment regimen:*<br>927/1881 49% Systemically untreated<br>326/1881 17% Tamoxifen alone<br><br>**Calcemia:**<br>- *Status:* NR<br>- *Method of diagnostic:* NR	- *Stage:*<br>Tumor size (n=1881)<br>Mean = 20 ± 12mm<br>Lymph node status<br>365/1881 19% positive<br>1383/1881 74% negative<br>133/1881 7% missing<br>- *Grade:*<br>239/1881 13% grade I<br>677/1881 36% grade II<br>495/1881 26% grade III<br>470/1881 25% unknown<br>- *Histological types:* NR<br>- *Molecular subtypes:*<br>ER+ 1225/1881 65% (261/1881, 14% unknown)<br>PR+ NR<br>HER2+ NR	- *Sample type:* tumor<br>- *Tumor cells :* NR<br>- *Sampling method:* NR<br>- *Sample fixation:* NR<br>- *Samples storage:* NR<br>- *RNA extraction method:* NA<br>- *RNA quality assessment:* NA<br>- *cDNA synthesis method:* NA	- *Measurement method:* Affymetrix U133A microarrays<br>- *Probes:* Affymetrix probes<br>- *Quantification methods:* continuous log2 expression<br>- *Positive controls:* NR<br>- *Negative controls:* NR<br>- *Reproducibility assessments:* NR<br>- *Statistical analysis:* continuous	- Grade (I, II, III)<br>- ER status (+, -)	**<u>All patients:</u>**<br>*Association:*<br>- Grade<br>PTHrP expression was higher in well-differentiated low-grade (grade 1) tumors compared to grade 3<br><br>- ER status<br>PTHrP expression was higher in ER-positive breast cancer compared to ER-negative breast cancer

			PAM50 subtypes :<br>304/1881 16% basal<br>240/1881 13% HER2-enriched<br>465/1881 25% luminal A<br>471/1881 25% luminal B<br>304/1881 16% normal-like<br><br>**Metastases:**<br>- *N:* NR<br>- *Method of diagnostic:* NR				
Cohort 4	3,951	- *Period of recruitment:* NR<br>- *Age:* NR<br>- *Ethnicity:* NR<br>- *Menopausal status:* NR<br>- *Parity status:* NR<br>- *Follow-up:* censored at 10 years<br>- *Treatment regimen:* NR<br><br>**Calcemia:**<br>- *Status:* NR<br>- *Method of diagnostic:* NR	- *Stage:* NR<br>- *Grade:* NR<br>- *Histological types:* NR<br>- *Molecular subtypes:*<br>ER+ NR<br>PR+ NR<br>HER2+ NR<br><br>**Metastases:**<br>- *N:* NR<br>- *Method of diagnostic:* NR	- *Sample type:* tumor<br>- *Tumor cells : NR*<br>- *Sampling method: NR*<br>- *Sample fixation: NR*<br>- *Samples storage : NR*<br>- *RNA extraction method:* NA<br>- *RNA quality assessment:* NA<br>- *cDNA synthesis method:* NA	- *Measurement method:* Affymetrix HG-U133A and HG-U133 Plus 2.0 microarrays<br>- *Probes:* Affy ID: 206300_s_at<br>- *Quantification methods:* microarrays<br>- *Positive controls:* NR<br>- *Negative controls:* NR<br>- *Reproducibility assessments:* NR<br>- *Statistical analysis:* auto-selected cutoff value of 52 (range 1-13641, high *versus* low)	- Grade (I, II, III)<br>- ER status (+, -)	**<u>All patients:</u>**<br>*Association:*<br>- Grade<br>PTHrP expression was higher in well-differentiated low-grade (grade 1) tumors compared to grade 3<br><br>- ER status<br>PTHrP expression was higher in ER-positive breast cancer compared to ER-negative breast cancer
Assaker *et al.*, 2020, Canada, cohort study	314	- *Period of recruitment:* between January 1998 and December 2008<br>- *Age:* median = 52 years range 27 to 90 years<br>151/314 48% ≤50 years<br>163/314 52% >50 years<br>- *Ethnicity:* NR<br>- *Menopausal status:* NR<br>- *Parity status:* NR<br>- *Follow-up:* median for overall survival = 3.6 years range 0.1 to 9.8 years<br>- *Treatment regimen:*<br>178/314 57% MRM<br>136/314 43% breast conservative surgery (lumpectomy)<br><br>220/314 70% adjuvant chemotherapy (2/314, 1% missing)<br><br>192/314 61%	**Breast tumor:**<br>- *Stage:*<br>*Tumor size (cm)*<br>174/314 55% T1 (<2)<br>126/314 40% T2 (2-5)<br>14/314 4% T3 (>5)<br>*Lymph node status*<br>216/314 69% N0<br>39/314 12% N1<br>59/314 19% N2<br>- *Grade:*<br>6/314 2% I<br>41/314 13% II<br>265/314 84% III<br>2/314 1% missing<br>- *Histological types:* NR<br>- *Molecular subtypes:* 100% TNBC<br>ER+ 0/314 0%<br>PR+ 0/314 0%<br>HER2+ 0/314 0%<br>Ki67+ NR<br><br>**Metastases:**<br>- *N:* 0/314, 0% Patients with metastatic BC at presentation were excluded<br>- *Method of diagnostic:* NR	- *Sample type:* tumor<br>- *Tumor cells :* NR<br>- *Sampling method:* surgery<br>- *Sample fixation:* formalin-fixed paraffin-embedded<br>- *Samples storage:* NR<br>- *RNA extraction method:* NA<br>- *RNA quality assessment:* NA<br>- *cDNA synthesis method:* NA	- *Measurement method:* automated IHC<br>- *Antibodies/probes:* rabbit polyclonal anti-PTHrP antibody (Santa Cruz, sc20728, 1:10 dilution), PTHrP(41-177)<br>- *Housekeeping gene(s):* NA<br>- *Quantification methods:* pathologist blinded to clinical outcomes, PTHrP expression was estimated relative to its expression in normal breast tissue, the strongest PTHrP staining among cores from the same patients was used for the final scoring, localized to the cytoplasm<br>○ PTHrP-high = PTHrP expression higher than non-tumoral tissue<br>○ PTHrP-low = PTHrP expression lower than non-tumoral tissue<br>- *Positive controls:* normal kidney, normal Kupffer cells, normal tonsil inflammatory lymphocytes, normal pancreas, normal placenta (syncytiotrophoblasts/cytotrophoblasts)<br>- *Negative controls:* omission of the primary antibody, normal hepatocytes, normal tonsil	- Age (≤50 years, >50 years)<br>- Tumor size (T1, T2, T3)<br>- Grade (1, 2, 3)<br>- Lymph node status (N0, N1, N2)<br>- Lympho-vascular invasion (negative, positive)	**<u>All patients:</u>**<br>*No association:*<br>- Age<br>- Tumor size<br>- Grade<br>- Lymph node status<br>- Lympho-vascular invasion

		adjuvant radiotherapy, 121/192 63% breast/chest wall alone, 71/192 37% locoregional<br><br>**Calcemia:**<br>- *Status:* NR<br>- *Method of diagnostic:* NR			squamous epithelium, normal pancreas stroma<br>- *Reproducibility assessments:* various normal tissues have been assessed for PTHrP expression and compared to the Human Protein Atlas, each sample have been included in TMA in triplicate<br>- *Statistical analysis:* qualitative (PTHrP-high, PTHrP-low relative to normal tissue)<br>Chi-squared test and Fisher exact test have been used to evaluate the association of PTHrP expression with clinicopathologic characteristics		
Grinman *et al.*, 2022, USA, cross-sectional study	25	- *Period of recruitment:* NR<br>- *Age:*<br>TNBC (*n*=4) mean = 68.5 ± 10.5 years<br>HER2+ (*n*=6) mean = 59.3 ± 17.4 years<br>ER+ (*n*=18) mean = 61.8 ± 12.2 years<br>- *Ethnicity: NR*<br>- *Menopausal status:* NR<br>- *Parity status:* NR<br>- *Follow-up:* NR<br>- *Treatment regimen:* NR<br><br>**Calcemia:**<br>- *Status:* NR<br>- *Method of diagnostic:* NR	**Breast tumor:**<br>- *Stage:* NR<br>- *Grade:*<br>TNBC<br>4/4 100% grade III<br>HER2+<br>6/6 100% grade III<br>ER+<br>3/18 17% grade I<br>7/18 39% grade II<br>8/18 44% grade III<br>- *Histological types:* NR<br>- *Molecular subtypes:*<br>ER+ 15/25 60%<br>PR+ NR<br>HER2+ 6/25 24%<br>Ki67+ NR<br>TNBC = 4/25 16%<br><br>**Metastases:**<br>- *N:* NR<br>- *Method of diagnostic:* NR	- *Sample type:* tumor<br>- *Tumor cells :* NR<br>- *Sampling method:* patient-direct tumors were minced and then digested with collagenase and hyluronidase in DMEM/F12 containing insulin, hydrocorticone and EGF, supplemented with 5% bovin calf serum in the presence of deoxyribonuclease at 37°C with agitation. The resulting suspension was digested with 0.25% trypsin/1 mM EGTA for 1–3 min at 37°C. A single-cell suspension was obtained by filtration (40 μm). If necessary, red blood cells were removed by lysis.<br>- *Sample fixation:* NA<br>- *Samples storage:* NA<br>- *RNA extraction method:* NR<br>- *RNA quality assessment:* NR<br>- *cDNA synthesis method:* cDNA preparations according to the Single Cell 3' Protocol recommended by the manufacturer	- *Measurement method:* single cell RNA sequencing (Illumina output from 10X Genomics Chromium sequencing)<br>- *Antibodies/probes:* NR<br>- *Housekeeping gene(s):* NR<br>- *Quantification methods:* cells were divided into two groups depending on their normalized counts of *PTHLH*:<br>○ >0 count = high<br>○ 0 count = low<br>- *Positive controls:* NR<br>- *Negative controls:* NR<br>- *Reproducibility assessments:* NR<br>- *Statistical analysis:* categorical (PTHrP-high, PTHrP-low)	- Molecular subtype (ER+, HER2+, TNBC)	**<u>All patients:</u>**<br>*Association:*<br>The fractions of *PTHLH*-high cells were :<br>ER+ = 1.50%<br>HER2+ = 1.55%<br>TNBC = 8.92%
Shalaby *et al.*, 2025, Egypt, cohort study	123	- *Period of recruitment:* January 2018 to January 2022<br>- *Age:* mean = 55.05 ± 11.86, median = 55.0 (47.0-65.0)	**Breast tumor:**<br>- *Stage*[A]*:*<br>56/123 46% early<br>67/123 54% advanced<br>- *Grade*[Z]*:*<br>2/123 2% grade I	- *Sample type:* tumor<br>- *Tumor cells :* NR<br>- *Sampling method:* surgery<br>- *Sample fixation:* paraffin-embedded	- *Measurement method:* IHC<br>- *Antibodies/probes:* mouse monoclonal antibody (Chongqing Biospes, Catalog #YMA1281, 1:100 dilution)<br>- *Housekeeping gene(s):* NA	- Metastasis status (yes, no)<br>- Lymphovascular invasion (yes, no)<br>- Menopausal status	**<u>All patients:</u>**<br>• **High percent PTHrP expression**<br>*Association:*<br>- Presence of metastases (p-

	Range 30.3 to 82.0<br><br>39/123 32% < 50 years<br>84/123 68% ≥ 50 years<br><br>- *Ethnicity:* NR<br>- *Menopausal status:*<br>55/123 45% premenopausal<br>68/123 55% postmenopausal<br><br>- *Parity status:* NR<br>- *Follow-up:* January 2018 to December 2022, with survival time ranging from 9 to 52 months, mean = 30.15 ± 11.25 months, median = 30 months<br>- *Treatment regimen:*<br>103/123 84% modified radical mastectomy<br>20/123 16% breast conservative surgery<br>None of the patients received prior neoadjuvant therapy<br><br>**Calcemia:**<br>- *Status:* NR<br>- *Method of diagnostic:* NR	103/123 84% grade II<br>18/123 15% grade III<br>- *Histological types:* 123/123 (100%) invasive breast cancer of no special type<br>- *Molecular subtypes:*<br>ER[AE]+ 97/123 79%<br>PR[AE]+ 86/123 70%<br>HER2[AE]+ 48/123 39%<br>Ki67[AE]+ 53/123 43%<br><br>Luminal A 50/123 41%<br>Luminal B 47/123 38%<br>TNBC 10/123 8%<br>HER2 enriched 16/123 13%<br><br>**Metastases:**<br>- *N:* 13/123 (11%) diagnosed with metastatic disease<br>7/13 (54%) with bone metastases<br>- *Method of diagnostic:* NR	- *Samples storage:* NR<br>- *RNA extraction method:* NA<br>- *RNA quality assessment:* NA<br>- *cDNA synthesis method:* NA	- *Quantification methods:* quantitative (percent expression) and semi-quantitative (H-Score) cytoplasmic pattern of expression<br>- *Positive controls:* kidney<br>- *Negative controls:* NR<br>- *Reproducibility assessments:* each TMA block contained duplicate cores from each tissue sample<br>- *Statistical analysis:* quantitative (percent expression) and semi-quantitative (H-score)<br>Chi-squared test, Monte Carlo test, Student's t-test, Kruskal-Wallis test	(premenopausal, postmenopausal)<br>- Molecular subtype (luminal A, luminal B, TNBC, HER2-enriched)<br>- ER status (positive, negative)<br>- PR status (positive, negative)	value = 0.009)<br>- Lymphovascular invasion (p-value = 0.037)<br><br>• **High PTHrP H-score**<br>*Association:*<br>- Postmenopausal status (p-value = 0.044)<br>- Presence of metastasis (p-value = 0.007)<br>- Luminal B subtype (p-value = 0.022)<br>- ER positivity (p-value = 0.032)<br>- PR positivity (p-value = 0.005)

NOTE: sums of percentages could differ from 100 due to rounding of numbers.
ABBREVIATIONS: NR = not reported; NA = not applicable; ER = estrogen receptor; PR = progesterone receptor; HER2 = human epidermal growth factor receptor 2; IHC = immunohistochemistry; IRMA = immunoradiometric assay; RIA = radioimmunoassay; IFMA = immunofluorometric assay; OR = odds ratio; *GAPDH = Glyceraldehyde-3-phosphate dehydrogenase*
[A] According to the American Joint Committee on Cancer staging system;
[B] According to the method of Bloom and Richardson;
[C] According to Mercer *et al.,* tumors with hormone receptor levels ≥10fmol/mg protein were considered to be ER and/or PR positive while tumors with hormone receptor levels <5fmol/mg protein were considered to be negative. Tumors with hormone receptor levels between 5 and 10fmol/mg protein were considered equivocal,
[D] According to a modified version of Bloom and Richardson method;
[E] Assessed by dextran-coated charcoal method and Scatchard analysis, tumors containing hormone receptor levels ≥5fmol/mg protein were considered positive;
[F] According to the Union for International Cancer Control classification system;
[G] According to the methods of Bloom and Richardson and Scarff and Torlini;
[H] Assessed by dextran-coated charcoal method, tumors containing hormone receptor levels ≥10fmol/mg protein were considered positive. However, in Table 1 of the article, authors have presented the results as "<10fmol/mg of protein or ≤ 10fmol/mg of protein" instead of "<10fmol/mg or ≥10fmol/mg" or "≤10fmol/mg of protein or >10fmol/mg";
[I] Assessed by dextran-coated charcoal method, 10fmol/mg protein was used as threshold;
[J] Score calculated using the formula : pathological nodal status (N0 = 0, N1-3 = 13, N≥3 = 31) + ER status (ER-negative = 0, ER-positive = 15) + PR status (PR-negative = 0, PR-positive = 12.5) + Age (score 1 for each year > 65), patients in group 1 (favorable outcome) had a prognostic index < 25, patients in group 2 (unfavorable outcome) developed distant metastases within 3 years of presentation (regardless of their initial prognostic index), patients in group 3 (unfavorable presentation) had metastases at the time of presentation;
[K] According to Page & Anderson;
[L] Assessed according to the standard medical practice;
[M] 28 cases are mentioned in the materials and methods section of the article. 34 cases are mentioned in the title of Table 6. 35 cases are counted in Table 6;

[N] Assessed by dextran-coated charcoal method, tumors containing hormone receptor levels ≥10fmol/mg protein were considered positive;
[O] 132 patients with breast cancer have been mentioned in the "patients and methods" section of the article. 147 are mentioned in the "Relationship between tumor PTHrP and prognostic factors" section, *i.e.* 41 premenopausal and 106 postmenopausal.
[P] Assessed by dextran-coated charcoal method and Scatchard analysis, tumors containing hormone receptor levels >10fmol/mg protein were considered positive;
[Q] Using a standard cut-off level of 5fmol/mg tissue protein;
[R] According to the system adopted by the National Health Service Breast Screening Programme;
[S] Assessed by immunocytochemical method using commercial kits (Abbott Laboratories, Maidenhead, U.K.);
[T] According to WHO, Histological Typing of Breast Tumors, Ed. 2, Geneva, in *Neoplasma*, *30*(1): 113-120, 1982;
[U] Receptor levels >0.1fmol/μg DNA were considered positive;
[V] Assessed by standard dextran-coated charcoal assay;
[W] According to the methods of Scarff and Bloom and Richardson;
[X] Assessed by dextran-coated charcoal method, tumors containing hormone receptor levels >10fmol/mg protein were considered positive;
[Y] Assessed by enzymeimmunoassay (Sumitomo Kinzoku Bioscience, Tokyo, Japan). The cut-off point was 4.9fmol/mg protein;
[Z] According to the Elston-Ellis modification of the Scarff-Bloom-Richardson classification;
[AA] Assessed by immunohistochemistry (monoclonal antibodies, NCL-ER-6F11 and NCL-PR, Novocastra Laboratories Ltd., Newcastle upon Tyne, UK). Tumors were called positive if there was unequivocal nuclear staining in ≥10% of tumor cells;
[AB] Original cohort composed by 619 patients, PTHrP expression was evaluable in 410 patients only;
[AC] Original cohort composed by 540 patients, PTHrP expression was evaluable in 387 patients only;
[AD] Original cohort composed by 1,881 patients, PTHrP expression was evaluable in 737 patients only;
[AE] Assessed by immunostaining.
