# Supplementary material for: Clinical and prognostic significance of parathyroid hormone-related protein in breast cancer: a systematic review and meta-analyses of observational studies in women
Source: Endocr Relat Cancer. 2026 Mar 5;33(3):e250324. doi: 10.1530/ERC-25-0324 (PMC12978662; doi:10.1530/ERC-25-0324)
Supplement: Supplementary file 14 [file supplementary_table_6.pdf]

**Supplementary Table 6. Extraction sheet: Association between PTHrP/*PTHLH* expression and breast cancer prognostic**

| Study | Population | | | Tissue samples | | Statistical modeling | | Study results |
|---|---|---|---|---|---|---|---|---|
| **First author, year, Country, study design** | **N** | **Participant characteristics** | **Tumor characteristics** | **Tissue sample(s) type and preprocessing** | **PTHrP/*PTHLH* measurement(s)** | **Statistical model(s)** | **Adjustment** | **Outcomes** |
| Kao *et al.*, 1990, USA, cross-sectional study | 4 | - *Age:* NR<br>- *Ethnicity:* NR<br>- *Menopausal status:* NR<br>- *Parity status:* NR<br>- *Follow-up:* NR<br>- *Treatment regimen:* NR<br><br>**Calcemia:**<br>- *Status:* 4/4, 100% hypercalcemic<br>- *Method of diagnostic:* serum calcium level higher than normal range (8.9 to 10.1 mg/dL) | **Breast tumor:**<br>- *Stage:* NR<br>- *Grade:* NR<br>- *Histological types:* NR<br>- *Molecular subtypes:* NR<br><br>**Bone metastases:**<br>- *N:* 3/4 (75%) hypercalcemic patients with solid breast tumors but without bone metastases, 1/4 (25%) hypercalcemic patient with solid breast tumor and bone metastases<br>- *Method of diagnostic :* NR | - *Sample type:* blood (plasma)<br>- *Tumor cells :* NA<br>- *Sampling method:* blood collected in EDTA and centrifuged at 3,000*g* for 10 minutes<br>- *Sample fixation:* NA<br>- *Samples storage:* NR<br>- *RNA extraction method:* NA<br>- *RNA quality assessment:* NA<br>- *cDNA synthesis method:* NA | - *Measurement method:* RIA<br>- *Antibodies:* rabbit anti Human PTHrP(1-34), Peninsula Laboratories, inc. (Belmont, California)<br>- *Quantification methods:* standard curve<br>- *Positive controls:* synthetic human PTHrP(1-34)<br>- *Negative/specificity controls:* no cross-reactivity with the synthetic 1034 fragment of human PTH<br>- *Reproducibility assessments:* all standards and samples were measured in duplicate, within-assay and between-assay variabilities were less than 7% and 10% respectively<br>- *Statistical analysis:* continuous | NR | NR | <u>**All patients**</u><br>***Distant bone metastases***<br>No association<br>HR = NR<br><br>3/3 (100%) of patients with breast cancers without bone metastases and 1/1 (100%) of patients with breast cancers with bone metastases had a higher PTHrP level than healthy controls |
| Southby *et al.*, 1990, Australia, cohort study | 102 | - *Period of recruitment:* August 1987 to March 1989<br>- *Age:*<br>30/102 29% <50 years<br>61/102 60% >51 years<br>11/102 11% missing<br>- *Ethnicity:* NR<br>- *Menopausal status:* NR<br>- *Parity status:* NR<br>- *Follow-up:* NR<br>- *Treatment regimen:* Of the 91 patients who did not present with a local recurrence, 78% had a mastectomy, 20% had breast conservation and 2% had biopsy alone. In addition, 85% had an axillary clearance. Patients considered to have a high risk of recurrence were given adjuvant chemotherapy (25%), tamoxifen (29%) | **Breast tumor:**<br>- *Stage*[A]*:*<br>19/102 19% stage 1<br>46/102 45% stage 2<br>19/102 19% stage 3<br>7/102 7% stage 4<br>11/102 11% missing<br>- *Grade*[B]*:*<br>8/102 8% grade I<br>32/102 31% grade II<br>45/102 44% grade III<br>17/102 17% missing<br>- *Histological types:*<br>52/102 51% infiltrating ductal<br>22/102 22% infiltrating ductal + ductal carcinoma *in situ*<br>5/102 5% infiltrating lobular<br>3/102 3% mucinous<br>5/102 5% anaplastic<br>4/102 4% tubular/papillary/cribriform<br>11/102 11% missing<br>- *Molecular subtypes:* | - *Sample type:* tumor<br>- *Tumor cells :* NR<br>- *Sampling method:* surgery or biopsy<br>- *Sample fixation:* fixed in 10% buffered formalin for 12-24 hours and paraffin-embedded<br>- *Samples storage:* NR<br>- *RNA extraction method:* NA<br>- *RNA quality assessment:* NA<br>- *cDNA synthesis method:* NA | - *Measurement method:* IHC<br>- *Antibodies/probes:* New Zealand white rabbits' polyclonal antibody against PTHrP(1-34) (1:25 and 1:50 dilutions)<br>- *Housekeeping gene(s):* NA<br>- *Quantification methods:*<br>• <u>Positive/negative =</u> tumors were called positive if at least 1 tumoral cell was stained<br>• <u>Intensity of staining =</u> no staining of tumor cells (0), weak positive staining (1), moderately strong positive staining (2), strong positive staining (3)<br>• <u>Type of staining =</u> cytoplasmic pattern, vesicular pattern, mixed pattern<br>• <u>Area of staining =</u> estimate percentage of | Frequency analyses with Chi-squared and Fisher exact test when n<20 | *Method of selection:* NR<br>*Selected variables:* NR | <u>**Subgroup analysis**</u><br>***Local and distant recurrence***<br>- Stage 1-3 primary breast cancer *n* = 84<br>No association<br>HR = NR<br><br>49/84 (58%) PTHrP+<br>35/84 (42%) PTHrP-<br><br>17/84 (20%) have developed recurrence (local, distant, local+distant)<br><br>11/84 (13%) have developed distant metastases and 5/11 (45%) were PTHrP+ |

| | | | | | | | | |
|---|---|---|---|---|---|---|---|---|
| | | and radiotherapy (25%).<br><br>**Calcemia:**<br>- *Status:* 102/102 100% normocalcemic<br>- *Method of diagnostic:* NR | ER[c]+ 54/102 53% (15/102 15% missing)<br>PR[c]+ 49/102 48% (26/102 25% missing)<br>HER2+ NR<br>Ki67 NR<br><br>**Metastases:**<br>- *N:* 7/102 (7%) stage 4 at time of diagnosis, 17/102 (17%) missing<br><br>17/84 (20%) have developed recurrent disease (6/17 local + distant, 6/17 local only and 5/17 distant alone)<br><br>- *Method of diagnostic:* NR | | cells in the section which stained positively, 0-5% of positive tumor cells (1), 5-25% of positive tumor cells (2), 26-50% of positive tumor cells (3) and >50% positive cells (4)<br>• <u>Staining index =</u> area times intensity of staining, weak (1 and 2), moderate (3 and 4) and strong (>6)<br>- *Positive controls:* squamous cell carcinoma of the skin<br>- *Negative/specificity controls:* Alternative deletion of the antibody layers, pre-absorption of the primary antibody with 0.5mg/ml PTHrP(1-34), application of 0.5mg/ml PTHrP(1-34) to the section 5 minutes before the addition of the primary antibody, replacement of the primary antibody with non-immune rabbit serum<br>- *Reproducibility assessments:* Each tumor was assessed independently by two observers with ~95% agreement, when the two were not in concordance, the tumor was re-stained and reassessed by a panel of five observers, including the two initial observers and three observers blinded from clinicopathological data as well as previous results.<br>- *Statistical analysis:* qualitative<br>• Positive/negative<br>semiquantitative<br>• Intensity of staining (1, 2, 3)<br>• Area of staining (<5%, 6-25%, 26-50%, >51%)<br>• Staining index (weak, moderate, strong)<br>• Type of staining (cytoplasmic, vesicular, mixed) | | | |
| Bundred *et* | 98 | - *Period of recruitment:* | **Breast tumor:** | - *Sample type:* | - *Measurement method:* two | Mann- | *Method of* | <u>**Subgroup analysis:**</u> |

| | | | | | | | | |
|---|---|---|---|---|---|---|---|---|
| *al.*, 1991, UK, cross-sectional study | | NR<br>- *Age:* NR<br>- *Ethnicity:* NR<br>- *Menopausal status:* NR<br>- *Parity status:* NR<br>- *Follow-up:* NR<br>- *Treatment regimen:* NR<br><br>**Calcemia:**<br>- *Status :* 13/98 (13%) hypercalcemic (100% had bone metastases)<br>- *Method of diagnostic :* NR<br>- *Subtypes:* NR<br>- *PTH levels:* reference range (9-40ng/L)<br>9/10 patients with bone metastases and detectable PTHrP in whom PTH has been measured, the mean concentration was 23 (range 8-32) ng/L. In 9 hypercalcemic patients, plasma PTH concentration was <5ng/L in 4/9 and 5/9 had a concentration of 11 (range 7-17) ng/L. | - *Stage:*<br>57/98 (58%) early operable breast cancer without apparent bone metastases<br>41/98 (42%) stage 4<br>- *Grade:* NR<br>- *Histological types:* NR<br>- *Molecular subtypes:*<br>ER+ NR<br>PR+ NR<br>HER2+ NR<br>Ki67+ NR<br><br>**Metastases:**<br>- *N:* 41/98 (42%) bone metastases<br>- *Method of diagnostic :* NR<br>- *Bone markers:* NR<br>- *Subtypes:* NR | blood (plasma)<br>- *Tumor cells :* NA<br>- *Sampling method:* NR<br>- *Samples fixation:* NA<br>- *Samples storage:* NR<br>- *RNA extraction method:* NA<br>- *RNA quality assessment:* NA<br>- *cDNA synthesis method:* NA | site IRMA (INCSTAR, Wokingham, Berkshire)<br>- *Antibodies:* anti-PTHrP(1-86)<br>- *Housekeeping gene(s):* NA<br>- *Quantification methods:* continuous, concentrations are undetectable ≤0.23pmol/L<br>- *Positive controls:* patients with cancer-related (non-breast cancer) hypercalcemia<br>- *Negative controls:* normocalcemic controls<br>- *Reproducibility assessments:* NR<br>- *Statistical analysis:* qualitative (positive *versus* negative) and quantitative | Whitney U test | *selection:* NR<br>*Selected variables:* NR | **Development of bone metastases**<br>*In patient without hypercalcemia (n=85)*<br>- 5/57 (9%) of early breast cancers without bone metastases (mean = 0.33pmol/L; range 0.25-0.46pmol/L)<br>*vs* 10/28 (36%) of breast cancers with bone metastases (mean = 0.59pmol/L; range 0.23-1.50pmol/L) |
| | 178 | - *Period of recruitment:* NR<br>- *Age:* NR<br>- *Ethnicity:* NR<br>- *Menopausal status:* NR<br>- *Parity status:* NR<br>- *Follow-up:* NR<br>- *Treatment regimen:* NR<br><br>**Calcemia:**<br>- *Status :* 178/178 (100%) normocalcemic at time of diagnostic, 25/178 (14%) subsequently developed hypercalcemia (100% had bone metastases)<br>- *Method of diagnostic:* corrected calcium range 2.7-3.9 mmol/l)<br>- *Subtypes:* NR<br>- *PTH levels:* NR | **Breast tumor:**<br>- *Stage:* NR<br>- *Grade:* NR<br>- *Histological types:* NR<br>- *Molecular subtypes:*<br>ER+ NR<br>PR+ NR<br>HER2+ NR<br>Ki67+ NR<br><br>**Metastases:**<br>- *N:* 72/178 (40%) with bone metastases as initial site of metastases<br>- *Method of diagnostic :* NR<br>- *Bone markers:* NR<br>- *Subtypes:* NR | - *Sample type:* tumor<br>- *Tumor cells :* NR<br>- *Sampling method:* NR<br>- *Samples fixation:* 4% formaldehyde in saline fixation and paraffin embedding<br>- *Samples storage:* NR<br>- *RNA extraction method:* NA<br>- *RNA quality assessment:* NA<br>- *cDNA synthesis method:* NA | - *Measurement method:* IHC<br>- *Antibodies:* rabbit antibody against mid-region sequence of PTHrP(37-67)<br>- *Housekeeping gene(s):* NA<br>- *Quantification methods:* only cytoplasmic reactivity was considered positive<br>- *Positive controls:* squamous carcinoma of lung<br>- *Negative controls:* omission of the primary antibody, preincubation of the primary antibody with PTHrP(37-67) for 16 hours at 4°C and non-immune serum as replacement of the primary antibody<br>- *Reproducibility assessments:* staining of tumors was assessed by two independent observers (blinded)<br>- *Statistical analysis:* qualitative (positive *versus* negative) | Fisher's exact test | *Method of selection:* NR<br>*Selected variables:* NR | **<u>All patients:</u>**<br>**Development of bone metastases**<br>- 55/106 (52%) of early breast cancers without bone metastases *vs* 47/72 (65%) of the primary tumors which developed bone metastases |
| Bundred *et* | 155 | - *Period of recruitment:* | **Breast tumor:** | - *Sample type:* | - *Measurement method:* IHC | Kaplan- | *Method of* | **<u>All patients</u>** |

| | | | | | | | | |
|---|---|---|---|---|---|---|---|---|
| *al.*, 1992, UK, cohort study | | August 1984 to December 1985<br>- *Age:*<br>65/155 42% ≤ 50 years<br>90/155 58% ≥ 51 years<br>- *Ethnicity:* NR<br>- *Menopausal status:*<br>43/155 28% premenopausal<br>112/155 72% postmenopausal<br>- *Parity status:* NR<br>- *Follow-up:* ≥ 5 years or until death, all patients were seen every 4 months in the first year after surgery and thereafter at 6-month intervals<br>- *Treatment regimen:* mastectomy with axillary clearance or node sampling (97/155, 63%), or breast conserving operations and radiotherapy (58/155, 37%)<br><br>**Calcemia:**<br>- *Status:* 155/155 100% normocalcemic<br>- *Method of diagnostic:* the upper limit of the reference range for corrected calcium was 2.65mmol/l | - *Stage:* NR<br>- *Grade*[D]*:*<br>30/155 19% grade I<br>88/155 57% grade II<br>37/155 24% grade III<br>- *Histological types:*<br>126/155 81% infiltrating ductal<br>29/155 19% other types<br>- *Molecular subtypes:*<br>ER[E]+ 64/155 41% (55/155 35% missing)<br>PR[E]+ 55/155 35% (69/155 45% missing)<br>HER2+ NR<br>Ki67+ NR<br><br>**Metastases:**<br>- *N:* none of the patients had clinical evidence of bone metastases at presentation<br>- *Method of diagnostic:* bone scan/scintigraphy, conventional radiography, ultrasound and/or surgical biopsy | tumor<br>- *Tumor cells :* NR<br>- *Sampling method:* surgery<br>- *Sample fixation:* 4% formaldehyde in saline and paraffin embedding<br>- *Samples storage:* NR<br>- *RNA extraction method:* NA<br>- *RNA quality assessment:* NA<br>- *cDNA synthesis method:* NA | - *Antibodies/probes:* rabbit anti PTHrP(37-67)<br>- *Housekeeping gene(s):* NA<br>- *Quantification methods:* only cytoplasmic staining was considered<br>- *Positive controls:* squamous carcinoma of the lung included in each batch of slides<br>- *Negative/specificity controls:* preincubation of the primary antibody with 0.5mg/ml PTHrP(37-67), omission of the primary antibody, primary antibody replaced by non-immune serum<br>- *Reproducibility assessments:* staining was assessed by two independent observers<br>- *Statistical analysis:* qualitative (positive *versus* negative) | Meier, univariate chi-squared for the log-rank test | *selection:* correlation between prognostic factors and PTHrP expression<br>*Selected variables:* None | ***Overall survival***<br>No association<br>24/87 (28%) of PTHrP-positive tumors have died compared to 19/68 (28%) PTHrP-negative tumors.<br>Univariate Chi-Squared for the log-rank test p = 0.74<br><br>***Recurrence free survival***<br>No association<br>31/87 (35%) of PTHrP-positive tumors have relapsed compared to 16/68 (24%) PTHrP-negative tumors.<br>Univariate Chi-Squared for the log-rank test p = 0.091<br><br>***Bone metastases free survival***<br>PTHrP expression was associated with a higher risk of bone metastases development<br>Univariate Chi-Squared for the log-rank test p = 0.029 |
| Bouizar *et al.*, 1993, France, cohort study | 38 | - *Period of recruitment:* between 1981 and 1989<br>- *Age:* mean = 58 years, range 38-79 years<br>13/38 34% ≤ 50 years<br>25/38 66% > 51 years<br>- *Ethnicity:* NR<br>- *Menopausal status:*<br>14/38 37% premenopausal<br>24/38 63% postmenopausal<br>- *Parity status:* NR<br>- *Follow-up:* at least 5 years<br>- *Treatment regimen:* NR<br><br>**Calcemia:** | **Breast tumor:**<br>- *Stage*[F]*:*<br>4/38 11% stage 1<br>21/38 55% stage 2<br>13/38 34% stage 3<br>- *Grade*[G]*:*<br>0/38 0% grade I<br>19/38 50% grade II<br>11/38 29% grade III<br>8/38 21% missing<br>- *Histological types:*<br>34/38 89% infiltrating ductal carcinoma<br>2/38 5% infiltrating lobular carcinoma<br>1/38 3% adenocarcinoma<br>1/38 3% medullary carcinoma<br>- *Molecular subtypes:* | - *Sample type:* tumor<br>- *Tumor cells :* selected by a pathologist<br>- *Sampling method:* surgery<br>- *Sample fixation:* fresh frozen in liquid nitrogen<br>- *Samples storage:* liquid nitrogen tumor bank<br>- *RNA extraction method:* guanidinium thiocyanate-phenol- | - *Measurement method:* RT-PCR<br>- *Antibodies/probes: F 5′-GCGACGATTCTTCCTTCACC-3′ and R 5′-AGAGTCTAACCAGGCAGAGC-3′*<br>- *Housekeeping gene(s):* NA<br>- *Quantification methods:* densitometric scanning<br>- *Positive controls:* NR<br>- *Negative/specificity controls:* absence of Moloney murine leukemia virus reverse transcriptase during cDNA preparation, reverse transcription done after digestion of RNA by RNAse A (*n* = 4)<br>- *Reproducibility* | Analysis of variance and regression analysis, Kruskal Wallis test, Mann-Whitney and Spearman rank correlation | *Method of selection:* NR<br>*Selected variables:* NR | <u>**Subgroup analyses**</u><br>**Development of bone metastases (*n*=28)**<br>PTHrP expression was higher in patients who developed bone metastases compared to patients without metastases<br>p < 0.001 |

| | | | | | | | | |
|---|---|---|---|---|---|---|---|---|
| | | - *Status:* 38/38, 100% normocalcemic<br>- *Method of diagnostic:* NR | ER[H]+ NR<br>PR[H]+ NR<br>HER2+ NR<br>Ki67 NR<br><br>**Metastases:**<br>- *N:*<br>11/38, 29% without recurrence nor metastases<br>10/38, 26% who developed metastases in soft tissues<br>17/38, 45% who developed bone metastases<br>- *Method of diagnostic:* NR | chloroform method<br>- *RNA quality assessment:* NR<br>- *cDNA synthesis method:* Moloney murine leukemia virus reverse transcriptase (BRL; Gibco France, Cergy, Paris, France) | *assessments:* PCR and densitometric scanning were performed twice for 12 tumors r = 0.92, p < 0.005, densitometric values were the mean of 3 scans<br>- *Statistical analysis:* continuous (absorbance) | | | |
| Kissin *et al.*, 1993, Australia, cohort study | 82 | - *Period of recruitment:* between 1984 and 1987<br>- *Age:* mean =<br>14/82 17% <50 years<br>68/82 83% >50 years<br>- *Ethnicity:* NR<br>- *Menopausal status:* NR<br>- *Parity status:* NR<br>- *Follow-up:* at least 3 years<br>- *Treatment regimen:*<br>9/82, 11% needle biopsy<br>15/82, 18% wide local excision<br>58/82, 71% total mastectomy<br><br>63/82, 77% axillary dissection<br><br>22/82, 27% adjuvant tamoxifen<br>16/82, 20% chemotherapy<br>27/82, 33% radiotherapy<br><br>**Calcemia:**<br>- *Status:* 13/82, 16% patients developed symptomatic hypercalcemia and/or prolonged hypercalcemia during the period of follow up.<br>- *Method of diagnostic:* NR | **Breast tumor:**<br>- *Stage*[F]:<br>8/82 10% 1<br>40/82 49% 2<br>8/82 10% 3<br>26/82 32% 4<br>- *Grade:* NR<br>- *Histological types:* NR<br>- *Molecular subtypes:*<br>ER[G]+ 53/82 65%<br>PR[G]+ 47/82 57%<br>HER2+ NR<br>Ki67+ NR<br><br>**Metastases:**<br>- *N:* 26/82, 32% patients had metastases at time of initial diagnosis<br>- *Method of diagnostic:* chest X-ray and bone scan | - *Sample type:* tumor<br>- *Tumor cells :* NR<br>- *Sampling method:* surgery<br>- *Sample fixation:* 10% buffered formalin for 12 to 24 hours and embedded in paraffin<br>- *Samples storage:* NR<br>- *RNA extraction method:* NA<br>- *RNA quality assessment:* NA<br>- *cDNA synthesis method:* NA | - *Measurement method:* IHC<br>- *Antibodies/probes:* rabbit polyclonal antibody against PTHrP(1-34)<br>- *Housekeeping gene(s):* NA<br>- *Quantification methods:* A tumor was called positive for PTHrP if any of the tumor cells were specifically stained brown (cytoplasmic and/or vesicular).<br>• Localization = cytoplasmic, vesicular, mixed<br>• Intensity (subjective) = weak (1), moderate (2), strong (3)<br>• Area of staining (percent positive tumor cells) = <5% (1), 6-25% (2), 26-50% (3), >50% (4)<br>• Staining index (intensity x area of staining) = 1 and 2 (weak), 3 and 4 (moderate), >6 (strong)<br>- *Positive controls:* squamous cell carcinoma of the skin<br>- *Negative/specificity controls:* alternate deletions of the primary antiserum, secondary antibody and peroxidase anti-peroxidase complex, pre-absorption of the anti-PTHrP(1-34) antiserum with 0.5 mg/ml PTHrP(1-34) overnight at 4C, sections of breast | Chi-squared test, Fisher exact test, Kaplan Meier estimates and Log Rank Test | *Method of selection:* NR<br>*Selected variables:* NR | **All patients**<br>***Overall survival***<br>No association<br>HR = NR<br><br>***Disease free interval***<br>No association<br>HR = NR<br><br>***Local recurrence***<br>No association<br>HR = NR<br><br>- Stage 1-3 primary breast cancer *n* = 56<br><br>37/56 (66%) PTHrP+<br>19/56 (34%) PTHrP-<br><br>26/56 (46%) patients have developed distant recurrence and 17/26 (65%) had a PTHrP+ primary tumor |

| | | | | | | | | |
|---|---|---|---|---|---|---|---|---|
| | | | | | tumors and squamous cell carcinoma of the skin where the specific antiserum was replaced with non-immune rabbit serum<br>- *Reproducibility assessments:* each tumor was stained twice, and in duplicate, evaluation of staining was done by 1 the principal investigator and subsequently by a panel of 4 observers with no knowledge of the patient data. If there was a disagreement between observers, the tumor was restained and reassessed.<br>- *Statistical analysis:* semiquantitative<br>• Intensity of staining (1, 2, 3)<br>• Area of staining (<5%, 6-25%, 26-50%, >51%)<br>• Staining index (weak, moderate, strong) | | | |
| Kohno *et al.*, 1994, Japan, cohort study | 28 | - *Period of recruitment:* between 1980 and 1985<br>- *Age:*<br>Group A, *n*=12 (skeletal metastases) 51±9 years<br>Group B, *n*=8 (lung metastases) 52±13 years<br>Group C, *n*=8 (no distant metastasis) 51±12 years<br>- *Ethnicity:* NR<br>- *Menopausal status:* NR<br>- *Parity status:* NR<br>- *Follow-up:* 8 years<br>- *Treatment regimen:* 28/28, 100% mastectomy<br><br>**Calcemia:**<br>- *Status:* NR<br>- *Method of diagnostic:* NR | **Breast tumor:**<br>- *Stage:* NR<br>- *Grade*[B]*:*<br>7/28 25% grade I<br>13/28 46% grade II<br>8/28 29% grade III<br>- *Histological types:* 28/28, 100% infiltrating ductal carcinoma<br>- *Molecular subtypes:*<br>ER[G]+ 10/28 36% (8/28, 29% missing)<br>PR[G]+ 10/28 36% (8/28, 29% missing)<br>HER2+ NR<br>Ki67 NR<br><br>**Metastases:**<br>- *N:* 12/28, 43% bone metastases, 8/28, 29% lung metastases<br>- *Method of diagnostic:* bone scanning, plain X-rays films | - *Sample type:* tumor<br>- *Tumor cells:* NR<br>- *Sampling method:* mastectomy<br>- *Sample fixation:* formalin-fixed and paraffin embedding<br>- *Samples storage :* NR<br>- *RNA extraction method:* NA<br>- *RNA quality assessment:* NA<br>- *cDNA synthesis method:* NA | - *Measurement method:* IHC<br>- *Antibody:* monoclonal antibody against PTHrP(1-34) designated 4B3<br>- *Housekeeping gene(s):* NA<br>- *Quantification methods:*<br>• Grade of the intensity of staining: no positive staining (0), weak positive staining (1), moderate staining (2), strongly positive staining (3)<br>• Grade of the area of staining: the percentage of stained cells in each section, no positive tumor cells (0), 1%-5% positive tumor cells (1), 6%-25% positive tumor cells (2), 26%-50% positive tumor cells (3), >50% positive tumor cells (5)<br>• Degree of staining: as sum of staining grades of the intensity and area, 1-2 (-) , 3 (±), 4-5 (+) and 6 (++)<br>- *Positive controls:* NR<br>- *Negative/specificity* | The statistical analysis of frequency data was done by Yates' corrected chi-squared test or Fisher's exact test | *Method of selection:* NR<br>*Selected variables:* NR | **All patients (*n*=28)**<br>16/28 (57%) PTHrP+<br>12/28 (43%) PTHrP-<br><br>**Subgroup analyses**<br>In all patients with metastases (*n*=20):<br>13/20 (65%) PTHrP+<br>7/20 (35%) PTHrP-<br><br>**Development of bone metastases (*n*=20)**<br>PTHrP was found (+, ++) in 10/12 (83%) patients with bone metastases (group A) compared to 3/8 (38%) patients without recurrence (group C) |

| | | | | | | | | |
|---|---|---|---|---|---|---|---|---|
| | | | | | *controls:* non-immunized mouse serum<br>- *Reproducibility assessments:* NR<br>- *Statistical analysis:* semiquantitative, frequences in degree of staining (-, ±, +, ++) and qualitative (-/± *versus* +/++) | | | |
| Bundred *et al.*, 1996, UK, cohort study | 227 | - *Period of recruitment:* NR<br>- *Age:* NR<br>- *Ethnicity:* NR<br>- *Menopausal status:* NR<br>- *Parity status:* NR<br>- *Follow-up:* NR<br>- *Treatment regimen:* NR<br><br>**Calcemia:**<br>- *Status:* 25/72 (35%) patients with primary breast cancer and bone metastases subsequently developed hypercalcemia, 5/155 (3%) patients with early breast cancer without bone metastasis subsequently developed hypercalcemia.<br>- *Method of diagnostic:* NR | **Breast tumor:**<br>- *Stage:* NR<br>- *Grade:* NR<br>- *Histological types:* NR<br>- *Molecular subtypes:*<br>ER+ NR<br>PR+ NR<br>HER2+ NR<br>Ki67+ NR<br><br>**Metastases:**<br>- N: 155/227 (68%) early breast cancer without metastasis at time of diagnostic, 72/227 (32%) primary breast cancers which had developed bone metastases<br>28/155 (18%) early breast cancer without metastasis at time of diagnostic have subsequently developed bone metastases<br>- *Method of diagnostic:* NA<br>- *Receptor status:* NA | - *Sample type:* tumor<br>- *Tumor cells :* NR<br>- *Sampling method:* NR<br>- *Sample fixation:* paraffin-fixed sections<br>- *Samples storage:* NR<br>- *RNA extraction method:* NA<br>- *RNA quality assessment:* NA<br>- *cDNA synthesis method:* NA | - *Measurement method:* IHC<br>- *Antibodies/probes:* polyclonal antibody against PTHrP(34-67)<br>- *Housekeeping gene(s):* NA<br>- *Quantification methods:* NR<br>- *Positive controls:* NR<br>- *Negative controls:* NR<br>- *Reproducibility assessments:* NR<br>- *Statistical analysis:* NR | NR | *Method of selection:* NR<br>*Selected variables:* NR | **<u>All patients</u>**<br>***Bone metastases development***<br>87/155 (56%) of primary breast tumors expressed PTHrP compared to 47/72 (65%) of primary breast tumors which had developed bone metastases<br><br>47/134 (35%) PTHrP-positive tumors had developed bone metastases compared to 25/93 (27%) PTHrP-negative<br><br>**<u>Subgroup analyses</u>**<br>***Bone metastases free survival***<br>- In patients with early breast cancer without bone metastasis (*n*=155), PTHrP staining was able to predict the development of bone metastases HR = NR<br>p = 0.029<br><br>***Overall survival***<br>- In patients with early breast cancer without bone metastasis (*n*=155)<br>No association |
| | 132 | - *Period of recruitment:* NR<br>- *Age:* NR<br>- *Ethnicity:* NR<br>- *Menopausal status:* NR<br>- *Parity status:* NR<br>- *Follow-up:* NR<br>- *Treatment regimen:* NR<br><br>**Calcemia:**<br>- *Status:* NR<br>- *Method of diagnostic:* NR | **Breast tumor:**<br>- *Stage:* NR<br>- *Grade:* NR<br>- *Histological types:* NR<br>- *Molecular subtypes:*<br>ER+ NR<br>PR+ NR<br>HER2+ NR<br>Ki67+ NR<br><br>**Metastases:**<br>- *Method of diagnostic:* NA<br>- *Receptor status:* NA | - *Sample type:* tumor<br>- *Tumor cells :* NR<br>- *Sampling method:* NR<br>- *Sample fixation:* snap frozen in liquid nitrogen<br>- *Samples storage:* NR<br>- *RNA extraction method:* NA<br>- *RNA quality assessment:* NA<br>- *cDNA synthesis* | - *Measurement method:* IRMA<br>- *Antibodies/probes:* PTHrP(1-86)<br>- *Housekeeping gene(s):* NR<br>- *Quantification methods:* only cytosol PTHrP was considered<br>- *Positive controls:* NR<br>- *Negative controls:* NR<br>- *Reproducibility assessments:* NR<br>- *Statistical analysis:* NR | NR | *Method of selection:* NR<br>*Selected variables:* NR | **<u>All patients</u>**<br>***Bone metastases development***<br>9/90 (10%) of PTHrP-positive tumors have developed bone metastases compared to 2/42 (5%) of PTHrP-negative |

| | | | | | | | | |
|---|---|---|---|---|---|---|---|---|
| | | | | *method:* NA | | | | |
| | 267 | - *Period of recruitment:* NR<br>- *Age:* NR<br>- *Ethnicity:* NR<br>- *Menopausal status:* NR<br>- *Parity status:* NR<br>- *Follow-up:* NR<br>- *Treatment regimen:* NR<br><br>**Calcemia:**<br>- *Status:* NR<br>- *Method of diagnostic:* NR | **Breast tumor:**<br>- *Stage:* NR<br>- *Grade:* NR<br>- *Histological types:* NR<br>- *Molecular subtypes:*<br>ER+ NR<br>PR+ NR<br>HER2+ NR<br>Ki67+ NR<br><br>**Metastases:**<br>- *N:* 195/267 (73%) early breast cancers without metastases, 72/267 (27%) primary breast cancer with bone metastases<br>- *Method of diagnostic:* NA<br>- *Receptor status:* NA | - *Sample type:* blood (plasma)<br>- *Tumor cells:* NR<br>- *Sampling method:* NR<br>- *Sample fixation:* NA<br>- *Samples storage:* NR<br>- *RNA extraction method:* NA<br>- *RNA quality assessment:* NA<br>- *cDNA synthesis method:* NA | - *Measurement method:* NR<br>- *Antibodies/probes:* PTHrP(1-86)<br>- *Housekeeping gene(s):* NR<br>- *Quantification methods:* continuous<br>- *Positive controls:* NR<br>- *Negative controls:* NR<br>- *Reproducibility assessments:* NR<br>- *Statistical analysis:* continuous | NR | *Method of selection:* NR<br>*Selected variables:* NR | **<u>All patients</u>**<br>***Bone metastases development***<br>19/195 (12%) early breast cancer patients had detectable PTHrP compared to 25/72 (32%) bone metastases patients $p \leq 0.001$ |
| Mawer *et al.*, 1997, UK, cross-sectional study | 129 | - *Period of recruitment:* NR<br>- *Age:* NR<br>- *Ethnicity:* NR<br>- *Menopausal status:* NR<br>- *Parity status:* NR<br>- *Follow-up:* NR<br>- *Treatment regimen:* No treatment or adjuvant tamoxifen. Patients with tamoxifen adjuvant therapy were changed to megesterol if disease progressed<br><br>**Calcemia:**<br>- *Status:* 12/129, 9% had bone metastases with hypercalcemia<br>- *Method of diagnostic:* hypercalcemia was defined as a serum calcium >2.6mmol/L when adjusted for serum albumin | **Breast tumor:**<br>- *Stage:*<br>88/129 68% operable early breast cancer<br>41/129 32% stage 4<br>- *Grade:* NR<br>- *Histological types:* NR<br>- *Molecular subtypes:*<br>ER+ NR<br>PR+ NR<br>HER2+ NR<br>Ki67+ NR<br><br>**Metastases:**<br>- *N:* 88/129, 68% patients had operable early breast cancers without metastases, 41/129, 32% had bone metastases<br>- *Method of diagnostic:* bone scan or plain x-rays | - *Sample type:* blood (plasma)<br>- *Tumor cells:* NR<br>- *Sampling method:* NR<br>- *Sample fixation:* NA<br>- *Samples storage:* NR<br>- *RNA extraction method:* NA<br>- *RNA quality assessment:* NA<br>- *cDNA synthesis method:* NA | - *Measurement method:* IRMA<br>- *Antibodies/probes:* targeting PTHrP(1-86)<br>- *Housekeeping gene(s):* NR<br>- *Quantification methods:* continuous, normal = limit of detection ≤0.23pmol/l<br>- *Positive controls:* NR<br>- *Negative controls:* NR<br>- *Reproducibility assessments:* NR<br>- *Statistical analysis:* continuous | Student's paired or unpaired t test or Mann-Whitney or Wilcoxon tests | *Method of selection:* NR<br>*Selected variables:* NR | **<u>All patients</u>**<br>***Bone metastases development***<br>No association<br>PTHrP was detectable in 8/88 (9%) women with early breast cancer, 1/29 (3%) women with bone metastases and eucalcemia |
| Bouizar *et al.*, 1999, France, cohort study | 74 | - *Period of recruitment:* NR<br>- *Age:* mean = 58 years range 38 to 79 years<br>19/74 26% ≤50 years<br>55/74 74% >50 years<br>- *Ethnicity:* NR<br>- *Menopausal status:*<br>11/74 15% premenopausal | **Breast tumor:**<br>- *Stage:*<br>Tumor size<br>14/74 19% ≤2cm<br>60/74 81% >2cm<br>Nodal status<br>18/74 24% 0<br>22/74 30% 1-3<br>29/74 39% >3<br>5/74 7% missing<br>- *Grade*[I]*:*<br>2/74 3% grade I | - *Sample type:* tumor<br>- *Tumor cells :* NR<br>- *Sampling method:* surgery<br>- *Sample fixation:* snap frozen in liquid nitrogen immediately after surgery and | - *Measurement method:* semiquantitative PCR AmpliTaq DNA polymerase (Apligene, Illkirch, France) reactions<br>- *Antibodies/probes:*<br>P1 transcripts :<br>F=5'GCAGCTTGGAAGAG GTAC3'<br>R=5'GCGGAGGAATGTTCA CAC3'<br>P2 transcripts : | Super-analysis of variance followed by Fisher's protected least significant difference test or Scheffe's S-test, | *Method of selection:* NR<br>*Selected variables:* NR | **<u>All patients</u>**<br>***Metastases development***<br>• PTHrP(139)/S14 was higher in tumors that metastasized (0.29 ± 0.03) than in patients who did not develop metastases (0.13 ± 0.03), $p < 0.01$<br>• PTHrP(141)/S14 No association<br>• PTHrP(173)/S14 No expression detected |

| | | | | | | | | |
|---|---|---|---|---|---|---|---|---|
| | | 63/74 85% postmenopausal<br>- *Parity status:* NR<br>- *Follow-up:* from 1 to 14 years (median = 7 years). Patients were reviewed every 3 months during the first 2 years and yearly thereafter.<br>- *Treatment regimen:* NR<br><br>**Calcemia:**<br>- *Status:* 74/74, 100% normocalcemic at diagnosis.<br>- *Method of diagnostic:* NR | 39/74 52% grade II<br>31/74 42% grade III<br>2/74 3% missing<br>- *Histological types:*<br>66/74 89% infiltrating ductal carcinoma<br>7/74 10% infiltrating lobular carcinoma<br>1/74 1%MC<br>- *Molecular subtypes:*<br>ER[J]+ 48/74 65%<br>PR[J]+ 29/74 39%<br>HER2+ NR<br>Ki67+ NR<br><br>**Metastases:**<br>- *N:* 0/74 0% at time of diagnosis.<br>During follow up, 18/74 (24%) did not develop metastasis while 56/74 (76%) developed metastases.<br>22/56 (39%) developed soft tissue metastases and 34/56 (61%) developed bone metastases<br>- *Method of diagnostic:* bone scintigraphy and conventional radiography | selection by the pathologist<br>- *Samples storage:* liquid nitrogen tumor bank<br>- *RNA extraction method:* guanidium thiocyanate-phenol/chloroform method using the ARNZol Kit (Bioprob Systems, Clays, Sous Bois, France)<br>- *RNA quality assessment:* NR<br>- *cDNA synthesis method:* NR | F=5'GTGTGAACATTCCTCCG3'<br>R=5'GTAGCTCAGCAGGAACAC3'<br>P3 transcripts :<br>F=5'CTGCTGGCCAGATTAATTAG3'<br>R=5'GTAGCTCAGCAGGAACAC3'<br>PTHrP 139 :<br>F=5'GCGACGATTCTTCCTTCACC3'<br>R=5'AGAGTCTAACCAGGCAGAGC3'<br>PTHrP 173 :<br>F=5'GCGACGATTCTTCCTTCACC3'<br>R=5'TGATGTGTTCTTCTGTTGTT3'<br>PTHrP 173 :<br>F=5'GCGACGATTCTTCCTTCACC3'<br>R=5'GATAGGTCATTCACTGTGCTC3'<br>PTHrP 141 :<br>F=5'GCGACGATTCTTCCTTCACC3'<br>R=5'CCTTGGAAGGTCTCTGCTGA3'<br>- *Housekeeping gene(s):* human small ribosomal protein 14 (S14)<br>- *Quantification methods:* quantitative, relative to S14<br>- *Positive controls:* TT cells (thyroid carcinoma) and MCF7 cells (breast carcinoma)<br>- *Negative/specificity controls:* omission of the Moloney murine leukemia virus reverse transcriptase or digestion of the samples with RNAse A<br>- *Reproducibility assessments:* internal control<br>- *Statistical analysis:* qualitative (present, absent) semiquantitative (ratio between PTHrP and S14) | distribution of frequency was compared using contingence analyses | | • P2-initiated PTHrP No association P2-initiated mRNA was present in 31/50 (62%) patients with metastases and 5/13 (38%) patients without metastases<br>• P3-initiated PTHrP was more frequent in patients who developed metastases (37/48, 78%) than in nonmetastatic tumors (11/16, 64%), $p < 0.01$<br>• P3-initiated PTHrP was higher in tumors that metastasized ($0.31 \pm 0.04$) than in patients who did not develop metastases ($0.13 \pm 0.04$), $p < 0.01$<br><br>***Bone metastases development***<br>• PTHrP(139)/S14 was more frequent in patients who developed bone metastases (33/34, 97%) than in nonmetastatic tumors (20/34, 57%), $p < 0.05$<br>• PTHrP(139)/S14 was higher in tumors from patients who developed bone metastases ($0.39 \pm 0.04$) than in tumors from patients who did not develop metastases ($0.13 \pm 0.03$), $p<0.0001$<br>• PTHrP(141)/S14 No association<br>• PTHrP(173)/S14 No expression detected<br>• P3-initiated PTHrP was higher in tumors from patients who developed bone metastases ($0.32 \pm 0.05$) than in tumors from patients who did not develop metastases ($0.13 \pm 0.04$), $p < 0.01$ |
| Yoshida *et al.*, 2000, Japan, cohort study | 177 | - *Period of recruitment:* 1990 to 1996<br>- *Age:* mean = 53.4 (range 27 to 87 years)<br>80/177 45% ≤50 years<br>97/177 55% >50 | **Breast tumor:**<br>- *Stage:*<br>109/177 62% stage 1<br>29/177 16% stage 2<br>23/177 13% stage 3<br>6/177 3% stage 4<br>10/177 6% unknown | - *Sample type:* tumor<br>- *Tumor cells :* NR<br>- *Sampling method:* surgical resection of the | - *Measurement method:* IHC<br>- *Antibodies/probes:* monoclonal antibody (Oncogene Science, Inc., Uniondale, NY, USA)<br>- *Housekeeping gene(s):* NA<br>- *Quantification methods:* | Kaplan-Meier estimator of the survival curves and log-rank test, Cox regression | *Method of selection:* NR<br>*Selected variables:*<br>- Age (≤ 50 or > 50)<br>- Tumor size (≤ | ***<u>Univariate</u>***<br>***<u>All patients</u>***<br>***Disease free survival***<br>No association when PTHrP is divided into –, + and ++, HR = NR, p=0.09 |

| | | | | | | | | |
|---|---|---|---|---|---|---|---|---|
| | | years<br>- *Ethnicity:* NR<br>- *Menopausal status:* NR<br>- *Parity status:* NR<br>- *Follow-up:* mean = 6.1 years<br>- *Treatment regimen:* NR<br><br>**Calcemia:**<br>- *Status:* 0/177 (0%) of the cases exhibited humoral hypercalcemia.<br>- *Method of diagnostic:* NR | - *Grade*[D]*:*<br>60/177 34% grade I<br>65/177 37% grade II<br>52/177 29% grade III<br>- *Histological types:* carcinoma<br>- *Molecular subtypes:*<br>ER[K]+ 89/177, 50%<br>PR+ NR<br>HER2+ NR<br>Ki67+ NR<br><br>**Metastases:**<br>- *N:* 6/177 (3%) with bone metastases at time of diagnosis (10/177, 6% missing)<br>23/177 (13%) developed bone metastases during the follow-up period, and 22/177 (12%) developed metastases to other tissue than bone<br>126/177 (71%) patients did not develop metastasis during the follow-up period<br>- *Method of diagnostic:* scintigraphy and plain X-ray | tumors<br>- *Sample fixation:* fixed in neutral formalin for 24h and paraffin-embedded<br>- *Samples storage:* NR<br>- *RNA extraction method:* NA<br>- *RNA quality assessment:* NA<br>- *cDNA synthesis method:* NA | • Intensity of cytoplasmic staining: no positive staining of tumor cells (0), weak positive staining of tumor cells (1), strong positive staining of tumor cells (2)<br>• Percentage of stained cells*:* no positive tumor cells (0), 1-20% of the tumor cells were positive (1), >20% of the tumor cells were positive (2)<br>• Degree of staining*;* determined as the sum of the staining intensity and percentage of cells stained, 0-1 (negative, -), 2-3 (weakly positive, +), 4 (strongly positive, ++)<br>- *Positive controls:* NR<br>- *Negative/specificity controls:* primary antibody replaced by non-immunized mouse serum<br>- *Reproducibility assessments:* NR<br>- *Statistical analysis:* semiquantitative (-, +, ++) and qualitative (negative (-), positive (+ and ++) | analysis and logistic regression analysis | 2.5 cm or > 2.5 cm)<br>- Nodal status (- or +)<br>- Grade (I, II or III)<br>- ER status (- or +) | ***Distant metastases to bones***<br>When PTHrP is divided into – and +, PTHrP expression is higher in group A (bone metastases with or without other metastases) compared to group C (without metastases), p<0.05<br><br>In patient with bone metastases, 24/29 (83%) were PTHrP+ (+, ++) while in patients with non-bone metastases 15/22 (68%) were PTHrP+ (+, ++)<br><br>**Subgroup analyses**<br>***Overall survival***<br>In patients without distant metastases at time of diagnostic and when PTHrP is divided into –, + and ++, PTHrP is associated with a worst patients' outcome p<0.05<br><br>***Distant metastases other than bone***<br>No association<br><br>***Multivariate***<br>**All patients**<br>***Distant metastases to bones***<br>Logistic regression analysis (multivariate)<br>• PTHrP (-, +)<br>OR = 3.131, p = 0.039<br>• PTHrP (-, +, ++)<br>No association<br><br>**Subgroup analyses**<br>***Overall survival***<br>In patients without distant metastases at time of diagnostic and when PTHrP is divided into –, + and ++, PTHrP is associated with a worst patients' outcome.<br>RR = 1.979 [1.090-3.590], p = 0.025 |
| Linforth *et al.*, 2002, UK, cohort study | 177 | - *Period of recruitment:* NR<br>- *Age:*<br>28/177 16% <50 | **Breast tumor:**<br>- *Stage:*<br>Tumor size<br>76/177 43% <20 mm | - *Sample type:* tumor<br>- *Tumor cells :* NR | - *Measurement method:* IHC<br>- *Antibodies:* polyclonal rabbit antibody against human PTHrP(1-34) | Pearson's chi-square test, Kaplan-Meier | *Method of selection:* variables known to predict patient | ***Univariate analyses***<br>***All patients:***<br>***Disease-free survival***<br>PTHrP is associated with a |

| | | | | | | | | |
|---|---|---|---|---|---|---|---|---|
| | | years<br>125/177 71% 50-70 years<br>24/177 14% >70 years<br>- *Ethnicity:* NR<br>- *Menopausal status:* NR<br>- *Parity status:* NR<br>- *Follow-up:* 5 years<br>- *Treatment regimen:* conservative wide local excision with axillary clearance and radiotherapy or mastectomy with axillary clearance and routine adjuvant therapy and follow-up in accordance to national guidelines<br><br>**Calcemia:**<br>- *Status:* NR<br>- *Method of diagnostic:* NR | 97/177 55% >20 mm<br>4/177 2% missing<br>Nodal status<br>104/177 59% -<br>66/177 37% +<br>7/177 4% missing<br>- *Grade:*<br>21/177 12% grade I<br>73/177 41% grade II<br>71/177 40% grade III<br>12/177 7% missing<br>- *Histological types:* NR<br>- *Molecular subtypes:*<br>ER+ 72/177 41% (55/177 31% missing)<br>PR+ 60/177 34% (55/177 31% missing)<br>HER2+ NR<br>Ki67 NR<br><br>**Metastases:**<br>- *N:* 0/177, 0% at time of diagnostic. All patients were early breast cancer patients.<br>- *Method of diagnostic:* NR | - *Sampling method:* surgery<br>- *Sample fixation:* fixed in 10% neutral buffered formalin and embedded in paraffin wax<br>- *Samples storage:* NR<br>- *RNA extraction method:* NA<br>- *RNA quality assessment:* NA<br>- *cDNA synthesis method:* NA | - *Housekeeping gene(s):* NA<br>- *Quantification methods:* From Iddon J. *et al.*, *The Journal of Pathology*, **2000**<br>• <u>Positive/negative:</u> specimens were scored positive if they exhibited brown cytoplasmic staining and negative if not<br>• <u>Percent positive cells:</u> 25% positive cells = 1 ; 25-75% positive cells = 2 ; more than 75% = 3<br>• <u>Intensity of the staining :</u> +, ++ and +++ for the most intense<br>• <u>Score:</u> percent positive cells x intensity of the staining<br>- *Positive controls:* known positive control (no detail)<br>- *Negative controls:* omission of the primary antiserum and known negative control (no detail)<br>- *Reproducibility assessments:* NR<br>- *Statistical analysis:* qualitative (positive *versus* negative) | estimates of the survival curves and log rank test | survival<br>*Selected variables:*<br>- Age (<50, 50-70, >70)<br>- Tumor size (<20mm, >20mm)<br>- Tumor grade (I, II, III)<br>- Nodal Status (+, -)<br>- ER status (+, -)<br>- PR status (+, -)<br>- PTHrP receptor expression (+, -) | reduction in disease-free survival<br>Pearson's chi-square test p = 0.03<br><br>**<u>Overall survival (5-year follow-up)</u>**<br>No association<br><br>***<u>Multivariate analyses</u>***<br>**<u>All patients:</u>**<br>**<u>Overall survival (5-year follow-up)</u>**<br>No association |
| Surowiak *et al.*, 2003, Poland, cohort study | 47 | - *Period of recruitment:* patients who underwent surgery in 1993-1994<br>- *Age:* mean = 60.39 ± 10.18 years<br>- *Ethnicity:* NR<br>- *Menopausal status:* NR<br>- *Parity status:* NR<br>- *Follow-up:* 7 years<br>- *Treatment regimen:* 47/47, 100% mastectomy (Patey's procedure) and subsequent tamoxifen therapy, 13/47 28% radiotherapy<br><br>**Calcemia:**<br>- *Status:* NR<br>- *Method of diagnostic:* NR | - *Stage:*<br>47/47 100% stage 2<br>- *Grade:*<br>47/47 100% grade II<br>- *Histological types:*<br>47/47 100% ductal carcinoma<br>- *Molecular subtypes:*<br>ER+ NR<br>PR+ NR<br>HER2+ NR<br>Ki67 NR<br><br>**Metastases:**<br>- *N:* 0/47, 0%<br>- *Method of diagnostic:* NR | - *Sample type:* tumor<br>- *Tumor cells :* NR<br>- *Sampling method:* mastectomy (Patey's procedure)<br>- *Sample fixation:* 10% buffered formalin followed by paraffin embedding<br>- *Samples storage:* NR<br>- *RNA extraction method:* NA<br>- *RNA quality assessment:* NA<br>- *cDNA synthesis method:* NA | - *Measurement method:* IHC<br>- *Antibodies:* mouse monoclonal (clone 212-10.7) anti-PTHrP(38-64) (Calbiochem, CA)<br>- *Housekeeping gene(s):* NA<br>- *Quantification methods:* immunoreactive score (IRS) range 0 to 12 (Remmele *et al.*)<br>- *Positive controls:* NR<br>- *Negative controls:* in each case, the negative control was included with Primary Negative Control (Dako, Denmark)<br>- *Reproducibility assessments:* NR<br>- *Statistical analysis:*<br>Semiquantitative IRS (range 0-12)<br>Qualitative IRS (IRS≤6 and IRS > 6) | Cox's model of proportional hazard and Kaplan-Meier curves<br><br>Relationship between intensity of PTHrP expression and the number of metastases: Kruskal-Wallis test | *Method of selection:* NR<br>*Selected variables:* NR | **<u>All patients</u>**<br>***Overall survival***<br>No association<br>Chi-squared = 0.72, p = 0.39<br>Mean survival time was higher in patients with IRS > 6 compared to patients with IRS ≤ 6, p = 0.03<br>***Relapse-free survival time***<br>No association<br>Chi-squared = 0.11, p = 0.74<br>***Distant metastases to bones***<br>No association<br>Chi-squared = 0.87, p = 0.35<br>***Distant metastases (excluding bone)***<br>No association<br>Number of metastases, range 0 to 3) H=3.34, p=0.34<br><br>**<u>Subgroup analyses</u>** |

| | | | | | | | | |
|---|---|---|---|---|---|---|---|---|
| | | | | | | | | ***Overall survival***<br>- IRS≤6 (*n*=22): low expression of PTHrP was related to patients' survival Chi-squared = 4.01, p = 0.045<br>- IRS>6 (*n*=25)<br>No association<br>- IRS=1 78% of patients survived 30 quarters, IRS=3.16 60% of patients survived 30 quarters, IRS=6 25% of patients survived 30 quarters |
| Henderson *et al.*, 2006, Australia, cohort study | 526 | - *Period of recruitment:* between December 1, 1989 and December 31, 1996<br>- *Age:* median = 60 years (range 27 to 93)<br>- *Ethnicity:* NR<br>- *Menopausal status:* 429/526 82% postmenopausal<br>97/526 18% premenopausal<br>- *Parity status:* NR<br>- *Follow-up:* minimum of 8 years, median = 10 years, patients were reviewed at least twice yearly for the first 3 years and then, annually.<br>- *Treatment regimen:* 28/526 5% preoperative chemotherapy<br>0/526 0% preoperative systemic therapy<br>264/526 50% mastectomy<br>226/526 43% breast conservative surgery<br>36/526 7% underwent lesser procedures because of morbidities or patient preference<br>216/526 41% postoperative radiotherapy<br>184/526 35% postoperative chemotherapy<br>295/526 56% Tamoxifen for 5 years<br><br>**Calcemia:**<br>- *Status:* NR<br>- *Method of diagnostic:* NR | - *Stage*[A]*:*<br>183/526 35% stage 1<br>161/526 31% stage 2a<br>71/526 13% stage 2b<br>77/526 15% stage 3a<br>34/526 6% stage 3b-c<br>- *Grade*[L]*:*<br>66/526 13% grade I<br>270/526 51% grade II<br>190/526 36% grade III<br>- *Histological types:*<br>458/526 87% ductal<br>35/526 7% lobular<br>31/526 6% other<br>- *Molecular subtypes:*<br>ER[M]+ 350/526 68% (11/526, 2% missing)<br>PR[M]+ 312/526 64% (35/526, 7% missing)<br>HER2+ NR<br>Ki67 NR<br><br>**Metastases:**<br>- *N:* 0/526, 0% at time of diagnosis<br>- *Method of diagnostic:* routine scans and blood tests | - *Sample type:* tumor<br>- *Tumor cells :* NR<br>- *Sampling method:* NR<br>- *Sample fixation:* NR<br>- *Samples storage :* NR<br>- *RNA extraction method:* NA<br>- *RNA quality assessment:* NA<br>- *cDNA synthesis method:* NA | - *Measurement method:* IHC<br>- *Antibody:* polyclonal rabbit anti-PTHrP(1-14)<br>- *Housekeeping gene(s):* NA<br>- *Quantification methods:* tumors were called positive when at least 1 tumor cell was stained for PTHrP<br>- *Positive controls:* normal skin<br>- *Negative/specificity controls:* nonimmune control, no cross-reactivity with PTH was observed<br>- *Reproducibility assessments:* 8% samples were re-stained and evaluated (100% concordance), each tumor section was stained in duplicate, with two dilutions of antiserum, and was assessed by a panel of individuals who were unaware of the clinical details<br>- *Statistical analysis:* qualitative (positive, negative) | Kaplan-Meier survival curves and Cox proportional hazards model. Nested models were compared using the likelihood ratio test | *Method of selection:* variables were fitted separately to datasets and those having no significant impact on the model were removed (age, ER status and vascular-lymphatic invasion)<br>*Selected variables:*<br>- Lymph node status (0, 1-3, 4-9 and >9)<br>- PR status[f]<br>- log tumor size | ***<u>Univariate analyses:</u>***<br>**<u>All patients:</u>**<br>***Overall survival***<br>PTHrP-positive staining was associated with a better survival outcome.<br>HR = 0.43 [0.30-0.62], p<0.001<br><br>***Bone metastases***<br>After stratification by stage, the HR was lower in the PTHrP-positive group.<br>HR = 0.63 [0.41-0.98], p = 0.04<br><br>***<u>Multivariate analyses:</u>***<br>**<u>All patients:</u>**<br>***Overall survival***<br>PTHrP-positive staining was associated with a better survival outcome.<br>HR = 0.47 [0.32-0.69], p<0.001 |

| | | | | | | | | |
|---|---|---|---|---|---|---|---|---|
| Zia *et al.*, 2007, UK, cohort study | 124 | - *Period of recruitment:* NR<br>- *Age:* NR<br>- *Ethnicity:* NR<br>- *Menopausal status:* NR<br>- *Parity status:* NR<br>- *Follow-up:* median = 10 years<br>- *Treatment regimen:* NR<br><br>**Calcemia:**<br>- *Status:* NR<br>- *Method of diagnostic:* NR | - *Stage:*<br>70/124 56% stage 1<br>40/124 32% stage 2<br>7/124 6% stage 3<br>4/124 3% stage 4<br>3/124 2% missing<br>- *Grade:*<br>24/124 19% grade I<br>42/124 34% grade II<br>58/124 47% grade III<br>- *Histological types:*<br>94/124 76% ductal<br>14/124 11% lobular<br>16/124 13% other<br>- *Molecular subtypes:*<br>ER+ NR<br>PR+ NR<br>HER2+ NR<br>Ki67 NR<br><br>**Metastases:**<br>- *N:* 4/124, 3% at time of diagnosis, 3/124 (2%) missing<br>7/124 (6%) developed distant metastases during the follow-up<br>- *Method of diagnostic:* NR | - *Sample type:* tumor<br>- *Tumor cells :* NR<br>- *Sampling method:* NR<br>- *Sample fixation:* fresh frozen in liquid nitrogen<br>- *Samples storage :* NR<br>- *RNA extraction method:* RNAzol procedure<br>- *RNA quality assessment:* spectrophotometry<br>- *cDNA synthesis method:* AB Gene Reverse Transcription System, Surrey, UK | - *Measurement method:* qPCR (AmplofluorTM UniprimerTM probe system, Intergen Company Oxford, UK)<br>- *Probe(s):* F: GGTGTTCCTGCTGAGCTAC<br>R: ACTGAACCTGACCGTACACGTAAATCTTGGATGGACTT<br>- *Housekeeping gene(s):* β-actin<br>- *Quantification methods:* purified plasmid internal standards<br>- *Positive controls:* purified plasmid internal standards<br>- *Negative controls:* NR<br>- *Reproducibility assessments:* qPCRs have been performed on duplicates and migration on agarose gel for products verification<br>- *Statistical analysis:* continuous (mean No. of copies per 50 ng RNA) | NR | NR | <u>**All patients:**</u><br>***Clinical outcomes (disease free, metastatic disease, local recurrence, breast cancer specific death)***<br>No association<br><br>***Survival status (disease free versus local recurrence + distant metastases + breast cancer specific death)***<br>Patients with poor prognosis (local recurrence, metastases or breast cancer specific death, *n*=30) had higher PTHrP levels (123±2.4) compared to good prognosis group (disease free, *n*=89, 4.8±2.3, p<0.05) |
| Bohn *et al.*, 2009, USA, cross-sectional study | 80 | - *Period of recruitment:* NR<br>- *Age:* NR<br>- *Ethnicity:* NR<br>- *Menopausal status:* NR<br>- *Parity status:* NR<br>- *Follow-up:* NR<br>- *Treatment regimen:* most patients received standard chemotherapy and hormonal treatment<br><br>**Calcemia:**<br>- *Status:* NR<br>- *Method of diagnostic:* NR | - *Stage:*<br>*Tumor size*<br>41/80 51% <2cm<br>27/80 34% >2cm<br>10/80 13% >5cm<br>2/80 3% unknown<br>*Node status*<br>34/80 43% positive<br>42/80 53% negative<br>4/80 5% unknown<br>- *Grade:* NR<br>- *Histological types:*<br>66/80 83% ductal<br>10/80 13% lobular<br>3/80 4% mixed<br>1/80 1% metaplastic<br>- *Molecular subtypes:*<br>ER+ 70/80 88%<br>PR+ 63/80 79% (1/80 1% unknown)<br>HER2+ 9/80 11% (2/80 3% unknown)<br>Ki67 NR<br><br>**Metastases:**<br>- *N:* 16/80 (20%) metastatic breast cancer to bone<br>- *Method of diagnostic:* NR | - *Sample type:* tumor<br>- *Tumor cells:* NR<br>- *Sampling method:* NR<br>- *Sample fixation:* formalin-fixed and paraffin embedded<br>- *Samples storage:* NR<br>- *RNA extraction method:* NA<br>- *RNA quality assessment:* NA<br>- *cDNA synthesis method:* NA | - *Measurement method:* IHC<br>- *Antibody:* rabbit polyclonal anti-PTHrP (Cosmo Bio Co., LTD/YII-Y201-EX, 1:100)<br>- *Housekeeping gene(s):* NA<br>- *Quantification methods:* qualitative, stains were considered positive if >1% cells showed cytoplasmic reactivity<br>- *Positive controls:* NR<br>- *Negative controls:* NR<br>- *Reproducibility assessments:* the tissue microarray with 3-fold redundancy was created<br>- *Statistical analysis:* qualitative (positive *versus* negative) | Fisher's exact test | *Method of selection:* NR<br>*Selected variables:* NR | <u>**All patients:**</u><br>***Distant metastases to bones***<br>No association |

| | | | | | | | | |
|---|---|---|---|---|---|---|---|---|
| Takagaki *et al.*, 2012, Japan, cohort study | 125 | - *Period of recruitment:* 1996 to 1999<br>- *Age:* mean = 57.4, range 31 to 88 years<br>- *Ethnicity:* NR<br>- *Menopausal status:*<br>35/125 28% premenopausal<br>90/125 72% postmenopausal<br>- *Parity status:* NR<br>- *Follow-up:* median = 97 months, range = 5 to 243 months<br>- *Treatment regimen:* patients who received either preoperative chemotherapy or endocrine therapy were excluded, then, patients have been treated with surgery and postoperative adjuvant therapy including chemotherapy, hormonal therapy and/or radiation therapy according to the status of the disease and the condition of the patient. No patient had received elective administration of bisphosphonates<br><br>**Calcemia:**<br>- *Status:* NR<br>- *Method of diagnostic:* NR | **Breast tumor:**<br>- *Stage:*<br>Tumor size<br>34/125 27% T1<br>63/125 50% T2<br>14/125 11% T3<br>11% T4<br>Nodal status<br>55/125 44% -<br>70/125 56% +<br>Metastasis<br>116/125 93% M0<br>9/125 7% M1<br>- *Grade:* NR<br>- *Histological types:* NR<br>- *Molecular subtypes:*<br>ER+ 61/125 49%<br>PR+ NR<br>HER2+ NR<br>Ki67 NR<br><br>**Metastases:**<br>- *N:* 9/125 % had distant metastases at time of surgery<br>20/125 (16%) developed bone metastases, 9/125 (7%) developed local recurrence and 25/125 (20%) succumbed during follow-up<br>- *Method of diagnostic:* ultrasonography for local recurrence, compute tomography for lung and liver metastases, annual scintigraphy for bone metastases and physical examination every 6 months | - *Sample type:* tumor<br>- *Tumor cells :* NR<br>- *Sampling method:* surgery<br>- *Sample fixation:* formalin-fixed, paraffin-embedded<br>- *Samples storage:* NR<br>- *RNA extraction method:* NA<br>- *RNA quality assessment:* NA<br>- *cDNA synthesis method:* NA | - *Measurement method:* IHC<br>- *Antibodies/probes:* mouse monoclonal anti-PTHrP (100$\mu$g/ml; Oncogene Science Inc., Uniondale, NY, USA)<br>- *Housekeeping gene(s):* NA<br>- *Quantification methods:* positive when >10% of the tumor cells were positive<br>- *Positive controls:* NR<br>- *Negative controls:* normal mouse IgG was substituted for the primary antibody<br>- *Reproducibility assessments:* staining was assessed by two investigators without knowledge of the clinical outcome of the patient independently (inter-reader variability NR)<br>- *Statistical analysis:* qualitative (positive, negative) | Kaplan-Meier estimator of the survival curves, log-rank test, logistic regression analysis and multivariate Cox regression analysis | *Method of selection:* NR<br>*Selected variables:*<br>- Menopausal status (pre- or postmenopausal )<br>- Tumor size (T1- T3 *versus* T4)<br>- Nodal status (-, +)<br>- ER status (-, +)<br>- Lymphatic infiltration (-, +)<br>- Vascular invasion (-, +) | ***Univariate analyses***<br>**All patients:**<br>***Distant bone metastases***<br>Patients with PTHrP+ tumor develop bone metastases more often (20/79, 25%) than those with a PTHrP- tumor (3/46, 7%) p = 0.018<br><br>**Subgroup analysis:**<br>***Overall survival (censored at 5 years)***<br>- In patients without initial metastatic disease (*n*=116), the 5-year survival rate was 94% in PTHrP-negative patients while it was 77% in PTHrP-positive patients p = 0.030<br><br>***Multivariate analyses***<br>**All patients:**<br>***Distant bone metastases***<br>PTHrP expression was an independent risk factor for bone metastasis.<br>HR = 7.104 [1.7815-48.1101], p = 0.0037<br><br>**Subgroup analysis:**<br>***Overall survival***<br>- In patients without initial metastatic disease (*n*=116), PTHrP expression was independently correlated with shorter overall survival RR = 3.644 [1.1821-15.8803], p = 0.0226 |
| Xu *et al.*, 2015, China, cohort study | 497 | - *Period of recruitment:* January 2006 to December 2009<br>- *Age:* median = 56.7 years range 26 to 95 years<br>267/497 54% ≤55 years<br>230/497 46% >55 years<br>- *Ethnicity:* NR<br>- *Menopausal status:* NR<br>- *Parity status:* NR<br>- *Follow-up:* median = 48 months range 2 to 85 months, 108/497 22% patients lost during the process<br>- *Treatment regimen:* surgery and standardized | **Breast tumor:**<br>- *Stage*[A]*:*<br>195/497 39% 1<br>210/497 42% 2<br>92/497 19% 3<br>- *Grade:*<br>352/497 71% ≤II<br>133/497 27% >II<br>12/497 2% missing<br>- *Histological types:*<br>423/497 85% infiltrating ductal carcinoma<br>74/497 15% other<br>- *Molecular subtypes:*<br>ER+ 341/497 69% (22/497 4% missing)<br>PR+ NR | - *Sample type:* tumor<br>- *Tumor cells :* NR<br>- *Sampling method:* surgery<br>- *Sample fixation:* immediately fixed in formalin following surgery, dehydrated and embedded in paraffin<br>- *Samples storage:* NR<br>- *RNA extraction* | - *Measurement method:* IHC<br>- *Antibodies/probes:* monoclonal antibody against PTHrP (ABGENAT, 1:2000 dilution)<br>- *Housekeeping gene(s):* NA<br>- *Quantification methods:* cytoplasmic staining was evaluated in a qualitative way (positive, negative)<br>- *Positive controls:* human placenta sections<br>- *Negative controls:* omission of the primary antibody<br>- *Reproducibility assessments:* staining has been assessed by 2 | Pearson's chi-squared correlation test, survival time was calculated by the Kaplan-Meier method and analyzed by the log-rank test and Cox proportional Hazard Model | *Method of selection:* NR<br>*Selected variables:*<br>- Tumor size (≤2 cm *versus* >2 cm)<br>- Skin involvement (no *versus* yes)<br>- Lymph node metastasis (no *versus* yes)<br>- Histologic grade (≤II *versus* >II)<br>- ER status (negative *versus* | ***Univariate analysis***<br>**All patients:**<br>***Bone metastases***<br>Higher bone metastases rate in patients with higher PTHrP expression (68%) compared to lower PTHrP expression (46%)<br>Chi-squared = 5.52<br>Chi-squared p=0.019<br><br>46/68 (68%) primary tumors from patients with bone metastases were PTHrP+ compared to 144/273 (53%) primary tumors from patients without recurrence (local + distal). |

| | | | | | | | | |
|---|---|---|---|---|---|---|---|---|
| | | adjuvant therapy.<br>Patients on neoadjuvant chemotherapy, as well as those with positive margins on histopathology were excluded<br><br>**Calcemia:**<br>- *Status:* NR<br>- *Method of diagnostic:* NR | HER2+ 265/497 53%<br>(55/497 11% missing)<br>Ki67+ NR<br><br>**Metastases:**<br>- *N:* 0/497, 0% at time of surgery.<br>After surgery, 116/497 (23%) patients suffered from local recurrence or distant metastasis. By the end of follow-up, 26/497 (5%) patients had died, 21/497 (4%) of breast cancer and 273/497 (55%) patients had developed no recurrence.<br>- *Method of diagnostic:* local or regional recurrence was confirmed by histology and the distant metastasis was detected by biopsy or imaging techniques. | *method:* NA<br>- *RNA quality assessment:* NA<br>- *cDNA synthesis method:* NA | independent pathologists in a blinded manner, discrepancies were solved by re-examination and consensus<br>- *Statistical analysis:* qualitative (positive, negative) | | positive)<br>- TGF-β (positive *versus* negative) | In PTHrP+ tumors, 46/190 (24%) developed bone metastases while 144/190 (76%) did not develop any recurrence. On the other hand, in PTHrP- tumors, 22/151 (15%) developed bone metastases while 129/151 (85%) did not develop any recurrence.<br><br>***Disease free survival***<br>No association<br>68/212 (32%) PTHrP-positive tumors have relapsed compared to 48/177 (27%) PTHrP-negative tumors. Chi-squared= 1.133, p=0.287<br><br>**<u>Subgroup analyses:</u>**<br>***Disease free survival***<br>- In patients with ER-negative tumors, higher PTHrP was associated with a worst prognosis<br>p = 0.027<br><br>- In patients with ER-positive tumors, PTHrP was not associated with prognosis<br><br>***<u>Multivariate analyses</u>***<br>**<u>All patients:</u>**<br>***Disease free survival***<br>No association<br>PTHrP was not an independent prognostic factor<br>HR = 1.022 [0.684 – 1.527]; p = 0.916 |
| Tran *et al.*, 2018, USA, cohort study | 180 | - *Period of recruitment:* NR<br>- *Age:* NR<br>- *Ethnicity:* NR<br>- *Menopausal status:* NR<br>- *Parity status:* NR<br>- *Follow-up:* NR<br>- *Treatment regimen:* NR<br><br>**Calcemia:**<br>- *Status:* NR<br>- *Method of diagnostic:* NR | **Breast tumor:**<br>- *Stage:* NR<br>- *Grade (invasive tumors only, n=100)*:<br>20/100 20% grade I<br>40/100 40% grade II<br>40/100 40% grade III<br>- *Histological types:*<br>40/180 22% normal breast<br>20/180 11% ductal carcinoma *in situ*<br>100/180 56% invasive breast carcinomas<br>20/180 11% lymph | - *Sample type:* tumor<br>- *Tumor cells :* NR<br>- *Sampling method:* surgery<br>- *Sample fixation: NR*<br>- *Samples storage:* NR | - *Measurement method:* immunofluorescence-based IHC<br>- *Antibodies:* Santa Cruz, H137, PTHrP(41-177), 1:200 dilution<br>- *Housekeeping gene(s):* NA<br>- *Quantification methods:* AQUA scores<br>- *Positive controls:* NR<br>- *Negative controls:* NR<br>- *Reproducibility assessments:* NR<br>- *Statistical analysis:* continuous | Robust one-way analysis of variance model | *Method of selection:* NR<br>*Selected variables:* NR | **<u>All patients:</u>**<br>***Breast cancer progression***<br><u>Nuclear PTHrP</u><br>Gradual decrease in PTHrP expression according to breast cancer progression<br>p<0.001<br><br><u>Cytoplasmic PTHrP</u><br>Gradual decrease in PTHrP expression according to breast cancer progression<br>All p < 0.02 |

| | | | | | | | | |
|---|---|---|---|---|---|---|---|---|
| | | | node metastases<br>- *Molecular subtypes (invasive tumors only, n=100):*<br>ER+ 59/100 59%<br>PR+ 42/100 42%<br>HER2+ 20/100 20%<br>Ki67+ NR<br><br>**Metastases:**<br>- *N:* NR<br>- *Method of diagnostic:* NR | | | | | |
| Cohort 1 (training set) | 410<br>N | - *Period of recruitment:* between 1962 and 1982<br>- *Age:* mean = 58.1 (range 30 to 88)<br>- *Ethnicity:*<br>396/410 97% white<br>12/410 3% black<br>1/410 0% Asian<br>1/410 0% Other<br>- *Menopausal status:*<br>302/410 74% post-menopausal<br>108/410 26% pre-menopausal<br>- *Parity status:* NR<br>- *Follow-up:* median disease-free follow-up 8.8 years<br>- *Treatment regimen:* NR<br><br>**Calcemia:**<br>- *Status:* NR<br>- *Method of diagnostic:* NR | **Breast tumor:**<br>- *Stage:*<br>Tumor size (cm)<br>16/410 4% ≤0.5<br>59/410 14% >0.5-1<br>108/410 26% >1-2<br>159/410 39% >2-5<br>39/410 10% >5<br>29/410 7% missing<br>Nodal status<br>222/410 54% positive<br>188/410 46% negative<br>- *Grade:*<br>69/410 17% grade I<br>212/410 52% grade II<br>108/410 26% grade III<br>21/410 5% missing<br>- *Histological types:* NR<br>- *Molecular subtypes:*<br>ER+ 222/410 54% (9/410, 2% missing)<br>PR+ 211/410 51% (23/410, 6% missing)<br>HER2+ 43/410 10% (17/410, 4% missing)<br>Ki67+ NR<br><br>**Metastases:**<br>- *N:* NR<br>- *Method of diagnostic:* NR | - *Sample type:* tumor<br>- *Tumor cells :* NR<br>- *Sampling method:* NR<br>- *Sample fixation:* formalin-fixed, paraffin-embedded<br>- *Samples storage:* Yale University pathology archives<br>- *RNA extraction method:* NA<br>- *RNA quality assessment:* NA<br>- *cDNA synthesis method:* NA | - *Measurement method:* immunofluorescence-based IHC<br>- *Antibodies:* Santa Cruz, H137, PTHrP(41-177) 1:200 dilution<br>- *Housekeeping gene(s):* NA<br>- *Quantification methods:* nuclear PTHrP was considered, AQUA scores (mean signal intensity)<br>- *Positive controls:* NR<br>- *Negative controls:* NR<br>- *Reproducibility assessments:* NR<br>- *Statistical analysis:* continuous | Kaplan-Meier estimator of the survival curves and log-rank test, the threshold used in order to segregate PTHrP-high *versus* PTHrP-low was determined using recursive partitioning with 10 cross-validations to establish optimal cutpoint for dichotomization | *Method of selection:* NR<br>*Selected variables:* NR | **<u>All patients:</u>**<br>***Disease free survival***<br><u>Nuclear PTHrP</u> expression was associated with a better outcome<br>HR = 1.37 [1.04-1.80], p=0.028<br><br><u>Cytoplasmic PTHrP</u><br>No statistically significant association (data not shown) |
| Cohort 2 (validation set) | 387<br>O | - *Period of recruitment:* between 1988 and 2000<br>- *Age:* mean = 59.3 (range 29 to 90)<br>- *Ethnicity:*<br>330/387 85% White<br>46/387 12% Black<br>6/387 2% Asian<br>1/387 0% Other<br>4/387 1% missing<br>- *Menopausal status:*<br>286/387 74% post-menopausal<br>101/387 26% pre-menopausal | **Breast tumor:**<br>- *Stage:*<br>Tumor size (cm)<br>12/387 3% ≤0.5<br>43/387 11% >0.5-1<br>99/387 26% >1-2<br>75/387 19% >2-5<br>19/387 5% >5<br>139/387 36% missing<br>Nodal status<br>115/387 30% positive<br>153/387 40% negative<br>119/387 31% missing<br>- *Grade:* | - *Sample type:* tumor<br>- *Tumor cells :* NR<br>- *Sampling method:* NR<br>- *Sample fixation:* formalin-fixed, paraffin-embedded<br>- *Samples storage :* archives<br>- *RNA extraction* | - *Measurement method:* immunofluorescence-based IHC<br>- *Antibodies:* Santa Cruz, H137, PTHrP(41-177), 1:200<br>- *Housekeeping gene(s):* NA<br>- *Quantification methods:* AQUA scores (mean signal intensity)<br>- *Positive controls:* NR<br>- *Negative controls:* NR<br>- *Reproducibility assessments:* NR<br>- *Statistical analysis:* | Kaplan-Meier estimator of the survival curves and log-rank test the threshold used to segregate PTHrP-high *versus* PTHrP-low was the one determined on cohort 1 (*n*=410) | *Method of selection:* NR<br>*Selected variables:* NR | **<u>All patients:</u>**<br>***Recurrence free survival***<br><u>Nuclear PTHrP</u> expression was associated with a better outcome<br>HR = 1.86 [1.16-3.00], p=0.011<br><br><u>Cytoplasmic PTHrP</u><br>No statistically significant association |

| | | | | | | | | |
|---|---|---|---|---|---|---|---|---|
| | | - *Parity status:* NR<br>- *Follow-up:* median recurrence-free follow-up 7.5 years<br>- *Treatment regimen:* NR<br><br>**Calcemia:**<br>- *Status:* NR<br>- *Method of diagnostic:* NR | 71/387 18% grade I<br>245/387 63% grade II<br>63/387 16% grade III<br>8/387 2% missing<br>- *Histological types:* NR<br>- *Molecular subtypes:*<br>ER+ 222/387 57% (99/387, 26% missing)<br>PR+ 171/387 44% (101/387, 26% missing)<br>HER2+ 25/387 7% (140/387, 36% missing)<br>Ki67+ NR<br><br>**Metastases:**<br>- *N:* NR<br>- *Method of diagnostic:* NR | *method:* NA<br>- *RNA quality assessment:* NA<br>- *cDNA synthesis method:* NA | continuous | | | |
| Cohort 3 | 737 P | - *Period of recruitment:* NR<br>- *Age (n=1,881):* median = 55 ± 13 years<br>- *Ethnicity:* NR<br>- *Menopausal status:* NR<br>- *Parity status:* NR<br>- *Follow-up:* censored at 10 years<br>- *Treatment regimen:*<br>927/1,881 49% Systemically untreated<br>326/1,881 17% Tamoxifen alone<br><br>**Calcemia:**<br>- *Status:* NR<br>- *Method of diagnostic:* NR | **Breast tumor:**<br>- *Stage:*<br>Tumor size (n=1,881)<br>Mean = 20 ± 12mm<br>Lymph node status<br>365/1,881 19% positive<br>1,383/1,881 74% negative<br>133/1,881 7% missing<br>- *Grade:*<br>239/1,881 13% grade I<br>677/1,881 36% grade II<br>495/1,881 26% grade III<br>470/1,881 25% unknown<br>- *Histological types:* NR<br>- *Molecular subtypes:*<br>ER+ 1,225/1,881 65% (261/1,881, 14% unknown)<br>PR+ NR<br>HER2+ NR<br>Ki67+ NR<br><br>PAM50 subtypes :<br>304/1,881 16% basal<br>240/1,881 13% HER2-enriched<br>465/1,881 25% luminal A<br>471/1,881 25% luminal B<br>304/1,881 16% normal-like | - *Sample type:* tumor<br>- *Tumor cells :* NR<br>- *Sampling method:* NR<br>- *Sample fixation:* NR<br>- *Samples storage:* NR<br>- *RNA extraction method:* NR<br>- *RNA quality assessment:* NR<br>- *cDNA synthesis method:* NR | - *Measurement method:* Affymetrix U133A microarrays<br>- *Probes:* Affymetrix probes<br>- *Housekeeping gene(s):* NA<br>- *Quantification methods:* continuous log2 expression<br>- *Positive controls:* NR<br>- *Negative controls:* NR<br>- *Reproducibility assessments:* NR<br>- *Statistical analysis:* continuous | Kaplan-Meier estimator of the survival curves, log-rank test and multivariate Cox regression analysis, three quantiles, based on *PTHLH* expression were used to stratify the cohort in high *PTHLH* (0.247 to 6.075), medium *PTHLH* (-0.558 to 0.247) and low *PTHLH* (-4.97 to -0.558) | *Method of selection:* NR<br>*Selected variables:*<br>- tumor size (>2.0cm)<br>- age (>50)<br>- grade (III)<br>- node status (negative)<br>- ER status (positive) | ***Univariate analyses:***<br>**All patients:**<br>***Overall survival (10-year censoring)***<br>PTHrP expression (high, medium, low) was associated with a better outcome<br>p = 0.007<br><br>**Subgroups analyses:**<br>- In lymph node negative: low *PTHLH* was associated with a worst outcome<br>HR = NR, p = 0.031<br>- In ER-positive:<br>low *PTHLH* was associated with a worst outcome<br>HR = NR, p = 0.027<br>- In other subgroups (lymph node positive and ER-negative): no association<br><br>***Multivariate analyses:***<br>**All patients:**<br>***Overall survival (10-year censoring)***<br>Low tumor *PTHLH* was associated with an unfavorable outcome<br>HR = between 1.25 and 1.5, p = 0.03 |

| | | | | | | | | |
|---|---|---|---|---|---|---|---|---|
| | | | **Metastases:**<br>- *N:* NR<br>- *Method of diagnostic:* NR | | | | | |
| Cohort 4 | 3,951 | - *Period of recruitment:* NR<br>- *Age:* NR<br>- *Ethnicity:* NR<br>- *Menopausal status:* NR<br>- *Parity status:* NR<br>- *Follow-up:* censored at 10 years<br>- *Treatment regimen:* NR<br><br>**Calcemia:**<br>- *Status:* NR<br>- *Method of diagnostic:* NR | **Breast tumor:**<br>- *Stage:* NR<br>- *Grade:* NR<br>- *Histological types:* NR<br>- *Molecular subtypes:*<br>ER+ NR<br>PR+ NR<br>HER2+ NR<br>Ki67+ NR<br><br>**Metastases:**<br>- *N:* NR<br>- *Method of diagnostic:* NR | - *Sample type:* tumor<br>- *Tumor cells : NR*<br>- *Sampling method: NR*<br>- *Sample fixation: NR*<br>- *Samples storage : NR*<br>- *RNA extraction method:* NA<br>- *RNA quality assessment:* NA<br>- *cDNA synthesis method:* NA | - *Measurement method:* Affymetrix HG-U133A and HG-U133 Plus 2.0 microarrays<br>- *Probes:* Affy ID: 206300_s_at<br>- *Housekeeping gene(s):* NA<br>- *Quantification methods:* microarrays<br>- *Positive controls:* NR<br>- *Negative controls:* NR<br>- *Reproducibility assessments:* NR<br>- *Statistical analysis:* continuous | Kaplan-Meier estimator of the survival curves and log-rank test, the best cutoff to segregate PTHrP-high *versus* PTHrP-low was automatically selected | *Method of selection:* NR<br>*Selected variables:* NR | <u>**All patients:**</u><br>***Overall survival***<br>Low tumor *PTHLH* is associated with an unfavorable outcome<br>HR = 1.5 [1.3-1.7]<br>p < 0.001 |
| Assaker *et al.*, 2020, Canada, cohort study | 314 | - *Period of recruitment:* between January 1998 and December 2008<br>- *Age:* median = 52 years range 27 to 90 years<br>151/314 48% ≤50 years<br>163/314 52% >50 years<br>- *Ethnicity:* NR<br>- *Menopausal status:* NR<br>- *Parity status:* NR<br>- *Follow-up:* median for overall survival = 3.6 years range 0.1 to 9.8 years<br>- *Treatment regimen:*<br>178/314 57% MRM<br>136/314 43% breast conservative surgery (lumpectomy)<br><br>220/314 70% adjuvant chemotherapy (2/314, 1% missing)<br><br>192/314 61% adjuvant radiotherapy, 121/192 63% breast/chest wall alone, 71/192 37% locoregional<br><br>**Calcemia:**<br>- *Status:* NR | **Breast tumor:**<br>- *Stage:*<br>*Tumor size (cm)*<br>174/314 55% T1 (<2)<br>126/314 40% T2 (2-5)<br>14/314 4% T3 (>5)<br>*Lymph node status*<br>216/314 69% N0<br>39/314 12% N1<br>59/314 19% N2<br>- *Grade:*<br>6/314 2% grade I<br>41/314 13% grade II<br>265/314 84% grade III<br>2/314 1% missing<br>- *Histological types:* NR<br>- *Molecular subtypes:*<br>100% TNBC<br>ER+ 0/314 0%<br>PR+ 0/314 0%<br>HER2+ 0/314 0%<br>Ki67+ NR<br><br>**Metastases:**<br>- *N:* 0/314, 0% Patients with metastatic BC at presentation were excluded<br>- *Method of diagnostic:* NR | - *Sample type:* tumor<br>- *Tumor cells :* NR<br>- *Sampling method:* surgery<br>- *Sample fixation:* formalin-fixed paraffin-embedded<br>- *Samples storage:* NR<br>- *RNA extraction method:* NA<br>- *RNA quality assessment:* NA<br>- *cDNA synthesis method:* NA | - *Measurement method:* automated IHC<br>- *Antibodies/probes:* rabbit polyclonal anti-PTHrP antibody (Santa Cruz, sc20728, 1:10 dilution) PTHrP(41-177)<br>- *Housekeeping gene(s):* NA<br>- *Quantification methods:* pathologist blinded to clinical outcomes, PTHrP expression was estimated relative to its expression in normal breast tissue, the strongest PTHrP staining among cores from the same patients was used for the final scoring, localized to the cytoplasm<br>○ PTHrP-high = PTHrP expression higher than non-tumoral tissue<br>○ PTHrP-low = PTHrP expression lower than non-tumoral tissue<br>- *Positive controls:* normal kidney, normal Kupffer cells, normal tonsil inflammatory lymphocytes, normal pancreas, normal placenta (syncytiotrophoblasts/cytotrophoblasts)<br>- *Negative controls:* omission of the primary antibody, normal hepatocytes, normal tonsil squamous epithelium, | Chi-squared test, Fisher's exact test, Kaplan Meier estimates, Log Rank Test and proportional hazards regression models | *Method of selection:* variables with a statistically significant p value (p <0.05) in univariate analysis were included in the multivariable model<br>*Selected variables:*<br><u>**All patients:**</u><br>***Overall survival***<br>- Age at diagnosis (≤50 years *versus* >50 years)<br>- Tumor size (T2, T3 *versus* T1)<br>- Lymph node status (N1, N2 *versus* N0)<br>- Type of surgery (modified radical mastectomy *versus* breast conserving surgery)<br>- Adjuvant chemotherapy (yes *versus* no)<br><br>***Central nervous system progression-*** | <u>***Univariate analyses***</u><br><u>**All patients:**</u><br>***Overall survival***<br>PTHrP expression is associated with a worst prognostic<br>HR = 2.033 [1.221- 3.386]<br>p = 0.0064<br>log rank p = 0.0055<br><br>***Progression-free survival***<br>No association<br>HR = 1.487 [0.890- 2.485]<br>p = 0.1295<br>log rank p = 0.1270<br><br>PTHrP expression was not statistically significantly correlated with the rate and type of progression of TNBC patients.<br>25/130 19.2% of low-PTHrP tumors had a progression compared to 36/160 22.5% of high-PTHrP tumors p = 0.5631<br>Low-PTHrP :<br>8/130 6.1% locoregional<br>13/130 10% Distant<br>4/130 3.1% both<br>High-PTHrP :<br>11/160 6.9% locoregional<br>19/160 11.9% Distant<br>6/160 3.7% both<br>p = 0.9240<br><br>***Central nervous system*** |

| | | | |
|---|---|---|---|
| - *Method of diagnostic:*<br>NR | normal pancreas stroma<br>- *Reproducibility assessments:* various normal tissues have been assessed for PTHrP expression and compared to the Human Protein Atlas, each sample have been included in TMA in triplicate<br>- *Statistical analysis:* qualitative (PTHrP-high, PTHrP-low relative to normal tissue) | ***free survival***<br>- Lymph node status (N1, N2 *versus* N0)<br><br>**<u>Subgroups analyses:</u>**<br>***Overall survival***<br>*In lymph node-negative TNBC patients*<br>- Age at diagnosis (≤50 years *versus* >50 years)<br>- Grade (2 and 3 *versus* 1)<br>- Adjuvant chemotherapy (yes *versus* no) | ***progression free survival***<br>PTHrP expression was associated with a worst prognostic<br>HR = 5.519 [1.570- 19.398], p = 0.0077<br>Log rank p = 0.0029<br><br>***Brain metastases free survival***<br>PTHrP expression was associated with a worst outcome<br>Log rank p = 0.0019<br><br>**<u>Subgroup analyses:</u>**<br>***Overall survival***<br>- In lymph node-negative TNBC patients, PTHrP was associated with a worst outcome<br>HR = 2.238 [1.076-4.656], p = 0.0312<br>Log rank p = 0.0272<br>- In lymph node-positive TNBC patients<br>No association<br>- In lymph node-negative TNBC patients without adjuvant chemotherapy, PTHrP was associated with a worst outcome<br>HR = 2.576 [1.019-6.513], p = 0.0456<br>Log rank p = 0.0387<br>- In lymph node-negative TNBC patients with adjuvant chemotherapy, PTHrP<br>Log rank p = 0.1488<br><br>***<u>Multivariate analyses</u>***<br>**<u>All patients:</u>**<br>***Overall survival***<br>No association<br>HR = 1.590 [0.925- 2.736] p = 0.0936<br><br>***Central nervous system progression-free survival***<br>PTHrP expression was associated with a shorter central nervous system progression-free survival<br>HR = 5.014 [1.421- 17.692], p = 0.0122<br><br>**<u>Subgroup analyses:</u>** |

| | | | | | | | | |
|---|---|---|---|---|---|---|---|---|
| | | | | | | | | ***Overall survival***<br>- In lymph node-negative TNBC patients, PTHrP was associated with a shorter overall survival<br>HR = 2.423 [1.129-5.197]<br>p = 0.0231 |
| | 55[Q] | - *Period of recruitment:* NR<br>- *Age:* NR<br>- *Ethnicity:* NR<br>- *Menopausal status:* NR<br>- *Parity status:* NR<br>- *Follow-up:* NR<br>- *Treatment regimen:* NR<br><br>**Calcemia:**<br>- *Status:* NR<br>- *Method of diagnostic:* NR | **Breast tumor:**<br>- *Stage:* NR<br>- *Grade:* NR<br>- *Histological types:* 100% breast invasive carcinoma<br>- *Molecular subtypes:*<br>100% TNBC (determined by IHC)<br>ER+ 0%<br>PR+ 0%<br>HER2+ 0%<br>Ki67+ NR<br><br>**Metastases:**<br>- *Method of diagnostic:* NR<br>- *Receptor status:* NR | - *Sample type:* tumor<br>- *Tumor cells :* NR<br>- *Sampling method:* NR<br>- *Sample fixation:* NR<br>- *Samples storage:* NR<br>- *RNA extraction method:* NR<br>- *RNA quality assessment:* NR<br>- *cDNA synthesis method:* NR | - *Measurement method:* Agilent G4502A_07_3 plateform<br>- *Antibodies/probes:* NR<br>- *Housekeeping gene(s):* NA<br>- *Quantification methods:* *PTHLH* expression was filtered from 17,815 genes, and a cutoff of ±1.5-fold change as cutoff to determine high *versus* low *PTHLH* levels<br>- *Positive controls:* NR<br>- *Negative controls:* NR<br>- *Reproducibility assessments:* NR<br>- *Statistical analysis:* qualitative (high *versus* low) | Kaplan Meier estimates, Log Rank Test and proportional hazards regression models | *Method of selection:* NR<br>*Selected variables:* NR | **<u>All patients:</u>**<br>***Overall survival***<br>Patients with higher PTHrP (*n*=17) had a worst prognosis than patients with lower PTHrP expression (*n*=37) p=0.0018 |
| Shalaby *et al.*, 2025, Egypt, cohort study | 123 | - *Period of recruitment:* January 2018 to January 2022<br>- *Age:* mean = 55.05 ± 11.86, median = 55.0 (47.0-65.0)<br>Range 30.3 to 82.0<br><br>39/123 32% < 50 years<br>84/123 68% ≥ 50 years<br><br>- *Ethnicity:* NR<br>- *Menopausal status:*<br>55/123 45% premenopausal<br>68/123 55% postmenopausal<br><br>- *Parity status:* NR<br>- *Follow-up:* January 2018 to December 2022, with survival time ranging from 9 to 52 months, mean = 30.15 ± 11.25 months, median = 30 months<br>- *Treatment regimen:*<br>103/123 84% modified radical mastectomy<br>20/123 16% breast | **Breast tumor:**<br>- *Stage*[A]*:*<br>56/123 46% early<br>67/123 54% advanced<br>- *Grade*[L]*:*<br>2/123 2% grade I<br>103/123 84% grade II<br>18/123 15% grade III<br>- *Histological types:* 123/123 (100%) invasive breast cancer of no special type<br>- *Molecular subtypes:*<br>ER[R]+ 97/123 79%<br>PR[R]+ 86/123 70%<br>HER2[R]+ 48/123 39%<br>Ki67[R]+ 53/123 43%<br><br>Luminal A 50/123 41%<br>Luminal B 47/123 38%<br>TNBC 10/123 8%<br>HER2 enriched 16/123 13%<br><br>**Metastases:**<br>- *N:* 13/123 (11%) diagnosed with metastatic disease<br>7/13 (54%) with bone metastases | - *Sample type:* tumor<br>- *Tumor cells :* NR<br>- *Sampling method:* surgery<br>- *Sample fixation:* paraffin-embedded<br>- *Samples storage:* NR<br>- *RNA extraction method:* NA<br>- *RNA quality assessment:* NA<br>- *cDNA synthesis method:* NA | - *Measurement method:* IHC<br>- *Antibodies/probes:* mouse monoclonal antibody (Chongqing Biospes, Catalog #YMA1281, 1:100 dilution)<br>- *Housekeeping gene(s):* NA<br>- *Quantification methods:* quantitative (percent expression) and semi-quantitative (H-Score) cytoplasmic pattern of expression<br>- *Positive controls:* kidney<br>- *Negative controls:* NR<br>- *Reproducibility assessments:* each TMA block contained duplicate cores from each tissue sample<br>- *Statistical analysis:* quantitative (percent expression) and semi-quantitative (H-score) | Chi-squared test, Monte Carlo tests and Kruskal Wallis test | *Method of selection:* NR<br>*Selected variables:* NR | ***<u>Univariate analyses</u>***<br>**<u>All patients:</u>**<br>***Presence of bone metastasis***<br>High PTHrP percent expression was associated with the presence of bone metastasis (p-value = 0.0120)<br><br>***Breast cancer progression***<br>Significant progressive increase in the percent of PTHrP expression from normal (mean = 64.86 ± 11.58, median = 65.0) to adjacent DCIS lesions (mean = 68.06 ± 7.92, median = 65.0) peaking in invasive breast cancer (mean = 80.53 ± 8.91, median = 80.0; p-value < 0.001)<br><br>Significant progressive increase in the PTHrP H-score from normal (mean = 97.97 ± 37.18, median = 80.0) to adjacent DCIS lesions (mean = 186.5 ± 36.77, median = 195.0) |

| | | | | |
|---|---|---|---|---|
| | conservative surgery None of the patients received prior neoadjuvant therapy<br><br>**Calcemia:**<br>- *Status:* NR<br>- *Method of diagnostic:* NR | - *Method of diagnostic:* NR | | peaking in invasive breast cancer (mean = 209.51 ± 49.86, median = 210.0; p-value < 0.001) |

NOTE: sums of percentages could differ from 100 due to rounding of numbers.

ABBREVIATIONS: NR = not reported; NA = not applicable; ER = estrogen receptor; PR = progesterone receptor; HER2 = human epidermal growth factor receptor 2; IHC = immunohistochemistry; IRMA = immunoradiometric assay; RIA = radioimmunoassay; IFMA = immunofluorometric assay; RR = relative risk, HR = hazard ratio, OR = odds ratio; *GAPDH = Glyceraldehyde-3-phosphate dehydrogenase*

[A] According to the American Joint Committee on Cancer staging system;

[B] According to the method of Bloom and Richardson;

[C] According to Mercer *et al.,* tumors with hormone receptor levels ≥10fmol/mg protein were considered to be ER and/or PR positive while tumors with hormone receptor levels <5fmol/mg protein were considered to be negative. Tumors with hormone receptor levels between 5 and 10fmol/mg protein were considered equivocal,

[D] According to a modified version of Bloom and Richardson method;

[E] Assessed by dextran-coated charcoal method and Scatchard analysis, tumors containing hormone receptor levels ≥5fmol/mg protein were considered positive;

[F] According to the Union for International Cancer Control classification system;

[G] According to the methods of Bloom and Richardson and Scarff and Torlini;

[H] Assessed by dextran-coated charcoal method, tumors containing hormone receptor levels ≥10fmol/mg protein were considered positive. However, in Table 1 of the article, authors have presented the results as "<10fmol/mg of protein or ≤ 10fmol/mg of protein" instead of "<10fmol/mg or ≥10fmol/mg" or "≤10fmol/mg of protein or >10fmol/mg";

[I] According to the methods of Scarff and Bloom and Richardson;

[J] Assessed by dextran-coated charcoal method, tumors containing hormone receptor levels >10fmol/mg protein were considered positive;

[K] Assessed by standard dextran-coated charcoal assay;

[L] According to the Elston-Ellis modification of the Scarff-Bloom-Richardson classification;

[M] Assessed by immunohistochemistry (monoclonal antibodies, NCL-ER-6F11 and NCL-PR, Novocastra Laboratories Ltd., Newcastle upon Tyne, UK). Tumors were called positive if there was unequivocal nuclear staining in ≥10% of tumor cells;

[N] Original cohort composed by 619 patients, PTHrP expression was evaluable in 410 patients only;

[O] Original cohort composed by 540 patients, PTHrP expression was evaluable in 387 patients only;

[P] Original cohort composed by 1,881 patients, PTHrP expression was evaluable in 737 patients only as described in the material and methods section. It was also indicated in the main text of the results section, that the cohort was composed on 937 patients while figure 1E of the publication showed 737 patients only. In the multivariate analysis presented in figure 1F of the publication, *n*=698;

[Q] Subset of 55 TNBC patients from The Cancer Genome Atlas dataset;

[R] Assessed by immunostaining.
