# Supplementary material for: Clinical and prognostic significance of parathyroid hormone-related protein in breast cancer: a systematic review and meta-analyses of observational studies in women
Source: Endocr Relat Cancer. 2026 Mar 5;33(3):e250324. doi: 10.1530/ERC-25-0324 (PMC12978662; doi:10.1530/ERC-25-0324)
Supplement: Supplementary file 16 [file supplementary_table_8.pdf]

Supplementary Table 8. Extraction sheet: Association between PTHrP/*PTHLH* expression and other genes

Study	Population		Tissue samples			Study results	
First author/year/ Country/ Study design	N	Participant characteristics	Tumor characteristics	Tissue sample(s) type and preprocessing	PTHrP/*PTHLH* measurement(s)	Statistical model(s)	Correlation
Downey *et al.*, 1997, UK, cross-sectional study	107	- *Period of recruitment:* NR<br>- *Age:* mean = 58.6 years range 30 to 79 years<br>- *Ethnicity:* NR<br>- *Menopausal status:*<br>9/107 8% premenopausal<br>98/107 92% postmenopausal<br>- *Parity status:* NR<br>- *Follow-up:* NR<br>- *Treatment regimen:* local excision followed by radiotherapy or mastectomy. Axillary node resection was carried out in 98/107 (92%) cases<br><br>**Calcemia:**<br>- *Status:* 107/107, 100% normocalcemic at time of surgery<br>- *Method of diagnostic:* NR	**Breast tumor:**<br>- *Stage:*<br>*Lymph node status*<br>28/107 26% positive<br>79/107 74% negative<br>- *Grade[A]:*<br>13/107 12% 1<br>63/107 59% 2<br>23/107 21% 3<br>8/107 7% missing<br>- *Histological types:*<br>91/107 85% ductal carcinoma<br>12/107 11% lobular carcinoma<br>3/107 3% mucoid carcinoma<br>1/107 1% tubular carcinoma<br>- *Molecular subtypes:*<br>ER[B]+ 75/107 70%<br>PR[B]+ 63/107 59%<br>HER2+ NR<br>Ki67[C] +<br>56/107 52% 1<br>35/107 33% 2<br>16/107 15% 3<br><br>**Metastases:**<br>- *N:* 0/107, 0% known to have bone metastases<br>- *Method of diagnostic:* NR	- *Sample type:* tumor<br>- *Tumor cells :* NR<br>- *Sampling method:* surgery<br>- *Sample fixation:* fixed in 10% v/v neutral buffered formalin and embedded in paraffin wax<br>- *Samples storage:* NR<br>- *RNA extraction method:* NA<br>- *RNA quality assessment:* NA<br>- *cDNA synthesis method:* NA	- *Measurement method:* IHC<br>- *Antibodies/probes:* antiserum raised in rabbit against PTHrP(1-34), dilution 1:800<br>- *Housekeeping gene(s):* NA<br>- *Quantification methods:* semiquantitative<br>• Number of positive tumor cells: 0% (0), <20% (1), 20-80% (2), >80% (3)<br>• Density of stain: none (0), weak (1), moderate (2), strong (3)<br>• Total score (number of positive tumor cells x density of stain): between 0 and 9<br>- *Positive controls:* breast cancer and skin<br>- *Negative/specificity controls:* omission of the primary antibody, non-immune rabbit serum, pre-absorption of PTHrP antibody with PTHrP at 4°C overnight<br>- *Reproducibility assessments:* staining was assessed by two independent observers, inter-observer agreement = 94%, slides were re-stained and reassessed if there was failure to achieve consensus<br>- *Statistical analysis:* qualitative (positive *versus* negative) relative to hyperplastic breast tissue	Mann-Whitney U-test to compare scores and chi-squared test to compare frequences	**All patients:**<br>No association between PTHrP and PTHrP receptor mRNA expression<br><br>36/72 (50%) PTHrP-positive tumors overexpressed *PTH1R*, while 13/35 (37%) PTHrP-negative tumors overexpressed *PTH1R*.
Iezzoni *et al.*, 1998, USA, cross-sectional study	52	- *Period of recruitment:* NR<br>- *Age:* mean = 59 years, median = 58 years, range 28-96 years<br>- *Ethnicity:* NR<br>- *Menopausal status:* NR<br>- *Parity status:* NR<br>- *Follow-up:* NR<br>- *Treatment regimen:* surgery, none of the patients had received preoperative	**Breast tumor:**<br>- *Stage:* NR<br>- *Grade[D]:*<br>6/52 11% grade 1<br>17/52 33% grade 2<br>29/52 56% grade 3<br>- *Histological types:*<br>2/52 4% lobular<br>50/52 96% ductal<br>- *Molecular subtypes:*<br>ER[E]+ 32/52 62% (3/52, 6% missing)<br>PR[E]+ 18/52 35% (9/52, 17%	- *Sample type:* tumor<br>- *Tumor cells :* NR<br>- *Sampling method:* surgery<br>- *Sample fixation:* zinc formalin-fixed and paraffin-embedded<br>- *Samples storage:* NR<br>- *RNA extraction method:* NA<br>- *RNA quality assessment:* NA<br>- *cDNA synthesis method:* NA	- *Measurement method:* IHC<br>- *Antibodies/probes:* murine monoclonal antibody (clone 9H7) against PTHrP(109-141)<br>- *Housekeeping gene(s):* NA<br>- *Quantification methods:* staining has been evaluated in the cytoplasm of tumor cells<br>• Intensity of the staining: no staining in comparison with the absorbed control (0), weak staining (1), moderate staining (2) and strong staining (3)<br>• Extent of the staining: no staining (0), 1-30% positive tumor cells (1), 31-60%	The Kruskal-Wallis test was used for the 3-group comparison of tumor grade versus staining index and the Wilcoxon rank-sum test was used for the 2-group comparison of the hormone receptors versus tumor staining index	**All patients:**<br>52/52 (100%) of the tumors were PTHrP-positive and 50/52 (96%) were also PTH1R-positive

		chemotherapy<br><br>**Calcemia:**<br>- *Status*: 52/52, 100% normocalcemic at time of surgery<br>- *Method of diagnostic:* NR	missing)<br>HER2+ NR<br>Ki67 NR<br><br>**Metastases:**<br>- *N:* NR<br>- *Method of diagnostic:* NR		positive tumor cells (2) and 61-100% positive tumor cells (3)<br>• Staining index: intensity of the staining times the extent of the staining = 1 and 2 (weak), 3 and 4 (moderate) and 6 and 9 (strong)<br>- *Positive controls:* adult kidney<br>- *Negative/specificity controls:* adsorption of the primary antibody with an excess of its specific immunogenic peptide; primary antibody, secondary antibody and avidin-horseradish peroxidase sequentially replaced by diluent; replacement of the anti-PTHrP antibody by the murine monoclonal antibody against tryptophan E<br>- *Reproducibility assessments:* NR<br>- *Statistical analysis:* semiquantitative (weak, moderate, strong)		
Dittmer *et al.*, 2006, The Netherlands, cross-sectional study	50	- *Period of recruitment:* between 1987 and 1997<br>- *Age:* NR<br>- *Ethnicity:* NR<br>- *Menopausal status:* NR<br>- *Parity status:* NR<br>- *Follow-up:* NR<br>- *Treatment regimen:* resection of primary tumor<br><br>**Calcemia:**<br>- *Status:* NR<br>- *Method of diagnostic:* NR	**Breast tumor:**<br>- *Stage:* 50/50 (100%) unilateral operable breast cancer)<br>- *Grade:* NR<br>- *Histological types:* NR<br>- *Molecular subtypes:*<br>ER+ NR<br>PR+ NR<br>HER2+ NR<br>Ki67+<br><br>**Metastases:**<br>- *Method of diagnostic:* NA<br>- *Receptor status:* NA	- *Sample type:* tumor<br>- *Tumor cells :* NR<br>- *Sampling method:* surgery<br>- *Samples fixation:* NR<br>- *Samples storage:* frozen<br>- *RNA extraction method:* RNeasy (Qiagen) or Nucleospin RNA II (Macherey & Nagel)<br>- *RNA quality assessment:* NR<br>- *cDNA synthesis method:* 1μg of total RNA with 1μl of Superscript II (200units/μl; Invitrogen)	- *Measurement method:* RT-PCR (Absolute QPCR SYBR Green Fluorescein mix, ABgene)<br>- *Antibodies/probes:*<br>PTHrP (exon 1C) F = 5'ACTAACGACCCGCCCTCG3'<br>R = 5' GAACAAGTTTCAAGTGCGTGT GTC3'<br>PTHrP (exon 2) F = 5'AGGAGGCGGTTAGCCCTGT3'<br>R = 5' TCCCATAGCAATGTCTAATTAA TCTGG3'<br>PTHrP (exon 4) F = 5' ACCTCGGAGGTGTCCCCTAAC 3'<br>R = 5' TCAGACCCAAATCGGACGG3'<br>- *Housekeeping gene(s): GAPDH*<br>- *Quantification methods:* comparative Ct ($2^{-\Delta\Delta Ct}$) method<br>- *Positive controls:* NR<br>- *Negative controls:* NR<br>- *Reproducibility assessments:* each sample was analyzed in duplicate<br>- *Statistical analysis:* quantitative	Spearman rank correlations	**All patients:**<br>*Association:*<br>Expression of *ITGA6* positively correlates with PTHrP (3 samples with the highest PTHrP expression, and low *ITGA6* have been excluded).<br><br>Expression of CDC2 and CDC25B were constant over a large range of PTHrP. However, higher levels of *CDC2* and *CDC25B* were only observed when PTHrP exon 4 levels were very low.<br><br>*No association:*<br>- *PAI-I*<br>- *uPA*
Mi *et al.*, 2010, China, cross-sectional study	35	- *Age:* mean = 53.6 ± 13.5 years<br>- *Ethnicity:* NR<br>- *Menopausal*	- *Stage:* NR<br>- *Grade:* NR<br>- *Histological types:* NR<br>- *Molecular subtypes:*	- *Sample type:* tumor<br>- *Tumor cells :* NR<br>- *Sampling method:*	- *Measurement method:* RT-PCR<br>- *Probes:* NR<br>- *Housekeeping gene(s): GAPDH*<br>- *Quantification methods:*	Chi-squared test and Fisher's exact test	**All patients:**<br>PTHrP and BMP-6 are inversely correlated<br>P = 0.000069

		status: NR<br>- *Parity status:* NR<br>- *Follow-up:* NR<br>- *Treatment regimen:* excision surgery<br><br>**Calcemia:**<br>- *Status:* NR<br>- *Method of diagnostic:* NR	ER+ NR<br>PR+ NR<br>HER2+ NR<br>Ki67 NR<br><br>**Metastases:**<br>- *Method of diagnostic:* NR<br>- *Receptor status:* NR	excision surgery<br>- *Sample fixation:* NR<br>- *Samples storage :* NR<br>- *RNA extraction:* total RNA was isolated using TRIzol Reagent (Life Technologies Inc., Grand Island, NY, USA)<br>- *RNA quality assessment:* NR<br>- *cDNA synthesis method:* 2μg of total RNA were used	continuous reported as the fold of relative light units for experimental groups when compared with those for control group after normalization with *GAPDH* expression<br>- *Positive controls:* NR<br>- *Negative controls:* NR<br>- *Reproducibility assessments:* three repeat tubes per cDNA specimen, three cDNA specimens independently for each data point<br>- *Statistical analysis:* categorical (tumor expression > non-tumoral margin expression, tumor expression < non-tumoral margin expression)		PTHrP expression was higher than in non-tumoral tissue in 17/35 (49%) patients. 12/17 (71%) had a lower BMP-6 expression in the tumor compared to non-tumoral tissue.<br><br>On the other hand, 18/35 (51%) patients had higher PTHrP-level in the non-tumoral tissue compared to tumoral tissue. 17/18 (94%) had a lower BMP-6 level in the non-tumoral tissue compared to the tumoral tissue.
Sato *et al.*, 2013, USA, cross-sectional study	100[F]	- *Period of recruitment:* NR<br>- *Age:* NR<br>- *Ethnicity:* NR<br>- *Menopausal status:* NR<br>- *Parity status:* NR<br>- *Follow-up:* NR<br>- *Treatment regimen:* NR<br><br>**Calcemia:**<br>- *Status:* NR<br>- *Method of diagnostic:* NR	**Breast tumor:**<br>- *Stage:* NR<br>- *Grade:*<br>20/100 20% I<br>40/100 40% II<br>40/100 40% III<br>- *Histological types:*<br>100/100 100% primary invasive ductal carcinoma<br>- *Molecular subtypes:*<br>ER[G]+ 59/100 59%<br>PR[G]+ 42/100 42%<br>HER2[G]+ 20/100 20%<br>Ki67+ NR<br><br>**Metastases:**<br>- *Method of diagnostic:* NR<br>- *Receptor status:* NR	- *Sample type:* tumor<br>- *Tumor cells :* NR<br>- *Sampling method:* surgery<br>- *Samples fixation:* NR<br>- *Samples storage:* NR<br>- *RNA extraction method:* NA<br>- *RNA quality assessment:* NA<br>- *cDNA synthesis method:* NA	- *Measurement method:* fluorescence-based quantitative IHC<br>- *Antibodies/probes:* PTHrP (Santa Cruz, 1 :200)<br>- *Housekeeping gene(s):* NA<br>- *Quantification methods:* AQUA score = average signal intensity within the cancer cell compartment normalized per cytokeratin signal<br>- *Positive controls:* NR<br>- *Negative controls:* NR<br>- *Reproducibility assessments:* NR<br>- *Statistical analysis:* quantitative	Pearson's correlation	**All patients (*n*=92):**<br>Positive correlation between PTHrP and Nuc-pYStat5 in breast tumors<br>Pearson's correlation R = 0.51<br>P < 0.001
Skondra *et al.* 2014, Greece, cross-sectional study	54	- *Period of recruitment:* NR<br>- *Age:* mean = 53 years (range 30-76)<br>22/54 41% <57 years<br>30/54 56% ≥57 years<br>2/54 4% missing<br>- *Ethnicity:* NR<br>- *Menopausal status:* NR<br>- *Parity status:* NR<br>- *Follow-up:* NR<br>- *Treatment regimen:* NR<br><br>**Calcemia:**	- *Stage:*<br>3/54 6% stage 1<br>28/54 52% stage 2<br>6/54 11% stage 3<br>16/54 30% stage 4<br>1/54 2% missing<br>- *Grade:*<br>12/54 22% grade I-II<br>35/54 65% grade III<br>7/54 13% missing<br>- *Histological types:*<br>40/54 74% ductal<br>8/54 15% lobular<br>4/54 7% others<br>2/54 4% missing	- *Sample type:* blood<br>- *Tumor cells:* NA<br>- *Sampling method:* 6ml of blood were collected using a venous catheter into 3ml EDTA-containing vacutainers after discarding the first 2ml of blood to avoid possible contamination with epidermal cells<br>- *Samples fixation:* NA<br>- *Sample storage:* samples were	- *Measurement method:* multiplex RT-PCR (Qiagen Multiplex PCR Kit, Qiagen, Hilden, Germany)<br>- *Probe(s):* F: CTGGTTCAGCAGTGGAGC<br>R: TTCTGCGATCAGATGGTG<br>- *Housekeeping gene(s): GAPDH*<br>- *Quantification methods:* positive or negative PTHrP detection<br>- *Positive controls:* spiking experiments of the MCF-7 in peripheral blood of healthy male blood samples<br>- *Negative controls:* peripheral blood of healthy blood samples<br>- *Reproducibility assessments:* NR<br>- *Statistical analysis:* qualitative (positive or negative)	Comparison between subgroups based on demographic, clinical and pathological characteristics for the expression of PTHrP was performed with the chi-squared test and Fisher's exact test. For comparisons of more than 2 groups, the	**All patients (*n*=54):**<br>37/54 (69%) patients had a *PTHLH*-positive tumor<br><br>34/54 (63%) patients had a *KRT19*-positive tumor<br><br>12/54 (22%) patients had a *MGB*-positive tumor<br><br>30/54 patients had a tumor *PTHLH* and *KRT19*-positive tumor<br><br>10/54 patients had a tumor *PTHLH* and *MGB*-positive tumor

		- *Status:* NR<br>- *Method of diagnostic:* NR	- *Molecular subtypes:*<br>ER+ : 32/54 59% (2/54, 4% missing)<br>PR+ : 19/54 35% (2/54, 4% missing)<br>HER2+ : 22/54 41% (2/54, 4% missing)<br>Ki67: 19/54 35% low (21/54, 39% missing)<br><br>**Metastases:**<br>- *N:* 16/54 30% had distant metastases at time of diagnosis<br>- *Method of diagnostic:* NR	processed within 6h of collection<br>- *RNA extraction method:* TriReagent RT-118<br>- *RNA quality assessment:* ultra-violet spectrophotometry<br>- *cDNA synthesis method:* Moloney Murine Leukemia Virus (M-MLV) Reverse Transcriptase (Invitrogen)		Kruskal-Wallis test was performed	
Tran *et al.*, 2018, USA, cohort study<br><br>Cohort 1 (training set)	410[l]	- *Period of recruitment:* between 1962 and 1982<br>- *Age:* mean = 58.1 (range 30 to 88)<br>- *Ethnicity:*<br>396/410 97% white<br>12/410 3% black<br>1/410 0% Asian<br>1/410 0% Other<br>- *Menopausal status:*<br>302/410 74% post-menopausal<br>108/410 26% pre-menopausal<br>- *Parity status:* NR<br>- *Follow-up:* median disease-free follow-up 8.8 years<br>- *Treatment regimen:* NR<br><br>**Calcemia:**<br>- *Status:* NR<br>- *Method of diagnostic:* NR	- *Stage:*<br>Tumor size (cm)<br>16/410 4% ≤0.5<br>59/410 14% >0.5-1<br>108/410 26% >1-2<br>159/410 39% >2-5<br>39/410 10% >5<br>29/410 7% missing<br>Nodal status<br>222/410 54% positive<br>188/410 46% negative<br>- *Grade:*<br>69/410 17% grade I<br>212/410 52% grade II<br>108/410 26% grade III<br>21/410 5% missing<br>- *Histological types:* NR<br>- *Molecular subtypes:*<br>ER+ 222/410 54% (9/410, 2% missing)<br>PR+ 211/410 51% (23/410, 6% missing)<br>HER2+ 43/410 10% (17/410, 4% missing)<br>Ki67+ NR<br><br>**Metastases:**	- *Sample type:* tumor<br>- *Tumor cells :* NR<br>- *Sampling method:* NR<br>- *Sample fixation:* formalin-fixed, paraffin-embedded<br>- *Samples storage:* Yale University pathology archives<br>- *RNA extraction method:* NA<br>- *RNA quality assessment:* NA<br>- *cDNA synthesis method:* NA	- *Measurement method:* immunofluorescence-based IHC<br>- *Antibodies:* Santa Cruz, H137, PTHrP(41-177), 1:200 dilution<br>- *Housekeeping gene(s):* NA<br>- *Quantification methods:* nuclear PTHrP was considered, AQUA scores (mean signal intensity)<br>- *Positive controls:* NR<br>- *Negative controls:* NR<br>- *Reproducibility assessments:* NR<br>- *Statistical analysis:* quantitative	Robust minimum covariance determinant estimator	**All patients :**<br>Nuclear PTHrP and nuclear pY-STAT5A/B (*n*=359):<br>R = 0.66 [0.56-0.73] p<0.001<br><br>Nuclear PTHrP and nuclear STAT5A (*n*=359):<br>R = 0.61 [0.53-0.70] p<0.001<br><br>Nuclear PTHrP and nuclear STAT5B (*n*=359):<br>R = 0.48 [0.22-0.64] p<0.001

			- *N:* NR<br>- *Method of diagnostic:* NR				
Cohort 2 (validation set)	387[J]	- *Period of recruitment:* between 1988 and 2000<br>- *Age:* mean = 59.3 (range 29 to 90)<br>- *Ethnicity:*<br>330/387 85% White<br>46/387 12% Black<br>6/387 2% Asian<br>1/387 0% Other<br>4/387 1% missing<br>- *Menopausal status:*<br>286/387 74% post-menopausal<br>101/387 26% pre-menopausal<br>- *Parity status:* NR<br>- *Follow-up:* median recurrence-free follow-up 7.5 years<br>- *Treatment regimen:* NR<br><br>**Calcemia:**<br>- *Status:* NR<br>- *Method of diagnostic:* NR	- *Stage:*<br>Tumor size (cm)<br>12/387 3% ≤0.5<br>43/387 11% >0.5-1<br>99/387 26% >1-2<br>75/387 19% >2-5<br>19/387 5% >5<br>139/387 36% missing<br>Nodal status<br>115/387 30% positive<br>153/387 40% negative<br>119/387 31% missing<br>- *Grade:*<br>71/387 18% grade I<br>245/387 63% grade II<br>63/387 16% grade III<br>8/387 2% missing<br>- *Histological types:* NR<br>- *Molecular subtypes:*<br>ER+ 222/387 57% (99/387, 26% missing)<br>PR+ 171/387 44% (101/387, 26% missing)<br>HER2+ 25/387 7% (140/387, 36% missing)<br>Ki67+ NR<br><br>**Metastases:**<br>- *N:* NR<br>- *Method of diagnostic:* NR	- *Sample type:* tumor<br>- *Tumor cells :* NR<br>- *Sampling method:* NR<br>- *Sample fixation:* formalin-fixed, paraffin-embedded<br>- *Samples storage :* Jefferson University Hospital pathology archives<br>- *RNA extraction method:* NA<br>- *RNA quality assessment:* NA<br>- *cDNA synthesis method:* NA	- *Measurement method:* immunofluorescence-based IHC<br>- *Antibodies:* Santa Cruz, H137, PTHrP(41-177), 1:200<br>- *Housekeeping gene(s):* NA<br>- *Quantification methods:* AQUA scores (mean signal intensity)<br>- *Positive controls:* NR<br>- *Negative controls:* NR<br>- *Reproducibility assessments:* NR<br>- *Statistical analysis:* quantitative	Robust minimum covariance determinant estimator	**All patients :**<br>Nuclear PTHrP and nuclear pY-Stat5a1b (*n*=250):<br>R = 0.56 [0.36-0.66] p<0.001<br><br>Nuclear PTHrP and nuclear STAT5A (*n*=250):<br>R = 0.80 [0.67-0.86] p<0.001<br><br>Nuclear PTHrP and nuclear STAT5B (*n*=250):<br>R = 0.67 [0.50-0.76] p<0.001<br><br>**Subgroups :**<br>Nuclear PTHrP and nuclear pY-STAT5A/B:<br>- Luminal A R = 0.46<br>- Luminal B R = 0.72<br>-HER2-positive R = 0.69<br>- Triple negative breast cancer R = 0.63
Assaker *et al.*, 2020, Canada, cohort study	22-5210	- *Period of recruitment:* NR<br>- *Age:* NR<br>- *Ethnicity:* NR<br>- *Menopausal status:* NR<br>- *Parity status:* NR<br>- *Follow-up:* NR<br>- *Treatment regimen:* NR<br><br>**Calcemia:**	**Breast tumor:**<br>- *Stage:* NR<br>*Lymph node status:* NR<br>- *Grade:* NR<br>- *Histological types:* NR<br>- *Molecular subtypes:* NR<br><br>**Metastases:**<br>- *N:* NR<br>- *Method of diagnostic:* NR	- *Sample type:* tumor<br>- *Tumor cells :* NR<br>- *Sampling method:* NR<br>- *Sample fixation:* NR<br>- *Samples storage:* NR<br>- *RNA extraction method:* NA<br>- *RNA quality*	- *Measurement method:* NR<br>- *Housekeeping gene(s):* NA<br>- *Quantification methods:* NR<br>- *Positive controls:* NR (<br>- *Negative controls:* NR<br>- *Reproducibility assessments:* NR<br>- *Statistical analysis:* quantitative	NR	**All patients (*n*=1036):**<br>*Association*<br>Correlation between *PTHLH* and *COX2* R = -0.07, p = 0.0251<br><br>*No association*<br>*HBEGF, ANGPTL4, ST6GALNAC5*<br><br>**TNBC (IHC):**<br>*Association*<br>Correlation between *PTHLH* and *HBEGF* (*n*=374) R = 0.1, p = 0.0444

	- *Status:* NR<br>- *Method of diagnostic:* NR	*assessment:* NA<br>- *cDNA synthesis method:* NA		Correlation between *PTHLH* and *ANGPTL4* (*n*=362) R = 0.1, p = 0.0252<br><br>Correlation between *PTHLH* and *TGFB1* (*n*=373) R = 0.11, p = 0.0344<br><br>*No association*<br>*ST6GALNAC5, COX2*<br><br>**Basal-like (Robust Molecular Subtype Predictors Classification using the six molecular subtype predictors) :**<br>*Association*<br>Correlation between *PTHLH* and *HBEGF* (*n*=580) R = 0.1, p = 0.0213<br><br>Correlation between *PTHLH* and *ANGPTL4* (*n*=516) R = 0.1, p = 0.0227<br><br>*No association*<br>*ST6GALNAC5, COX2, TGFB1*<br><br>**ER-negative (IHC) :**<br>*Association*<br>Correlation between *PTHLH* and *HBEGF* (*n*=1412) R = 0.06, p = 0.0387<br><br>*No association*<br>*ANGPTL4, ST6GALNAC5, COX2, TGFB1*<br><br>**ER-positive (IHC) :**<br>*Association*<br>Correlation between *PTHLH* and *COX2* (*n*=690) R = -0.09, p = 0.0163<br><br>*No association*<br>*HBEGF, ANGPTL4, ST6GALNAC5, TGFB1*<br><br>**HER2 (Robust Molecular Subtype Predictors Classification using the six molecular subtype predictors) :**<br>*No association*<br>*HBEGF, ANGPTL4, ST6GALNAC5, COX2, TGFB1*<br><br>**Luminal A (Robust Molecular Subtype Predictors Classification using the six molecular subtype predictors) :**<br>*No association*<br>*HBEGF, ANGPTL4, ST6GALNAC5, COX2, TGFB1*<br><br>**Luminal B (Robust Molecular Subtype Predictors Classification using the six molecular subtype predictors) :**<br>*Association*

							Correlation between *PTHLH* and *COX2* (*n=22)* R = -0.44, p = 0.0.0428<br><br>*No association*<br>*ANGPTL4, ST6GALNAC5, COX2, TGFB1*
Shalaby *et al.*, 2025, Egypt, cohort study	123	- *Period of recruitment:* January 2018 to January 2022<br>- *Age:* mean = 55.05 ± 11.86, median = 55.0 (47.0-65.0)<br>Range 30.3 to 82.0<br><br>39/123 32% < 50 years<br>84/123 68% ≥ 50 years<br><br>- *Ethnicity:* NR<br>- *Menopausal status:*<br>55/123 45% premenopausal<br>68/123 55% postmenopausal<br><br>- *Parity status:* NR<br>- *Follow-up:* January 2018 to December 2022, with survival time ranging from 9 to 52 months, mean = 30.15 ± 11.25 months, median = 30 months<br>- *Treatment regimen:*<br>103/123 84% modified radical mastectomy<br>20/123 16% breast conservative surgery<br>None of the patients received prior neoadjuvant therapy<br><br>**Calcemia:**<br>- *Status:* NR	**Breast tumor:**<br>- *Stage^{H}:*<br>56/123 46% early<br>67/123 54% advanced<br>- *GradK^{L}:*<br>2/123 2% grade I<br>103/123 84% grade II<br>18/123 15% grade III<br>- *Histological types:* 123/123 (100%) invasive breast cancer of no special type<br>- *Molecular subtypes:*<br>ER^{L}+ 97/123 79%<br>PR^{L}+ 86/123 70%<br>HER2^{L}+ 48/123 39%<br>Ki67^{L}+ 53/123 43%<br><br>Luminal A 50/123 41%<br>Luminal B 47/123 38%<br>TNBC 10/123 8%<br>HER2 enriched 16/123 13%<br><br>**Metastases:**<br>- *N:* 13/123 (11%) diagnosed with metastatic disease<br>7/13 (54%) with bone metastases<br>- *Method of diagnostic:* NR	- *Sample type:* tumor<br>- *Tumor cells :* NR<br>- *Sampling method:* surgery<br>- *Sample fixation:* paraffin-embedded<br>- *Samples storage:* NR<br>- *RNA extraction method:* NA<br>- *RNA quality assessment:* NA<br>- *cDNA synthesis method:* NA	- *Measurement method:* IHC<br>- *Antibodies/probes:* mouse monoclonal antibody (Chongqing Biospes, Catalog #YMA1281, 1:100 dilution)<br>- *Housekeeping gene(s):* NA<br>- *Quantification methods:* quantitative (percent expression) and semi-quantitative (H-Score) cytoplasmic pattern of expression<br>- *Positive controls:* kidney<br>- *Negative controls:* NR<br>- *Reproducibility assessments:* each TMA block contained duplicate cores from each tissue sample<br>- *Statistical analysis:* quantitative (percent expression) and semi-quantitative (H-score)	Chi-squared test, Monte Carlo tests and Kruskal Wallis test	**All patients:**<br>*Association*<br>Correlation between PTHrP and ezrin expression regarding percentage (r_s = 0.238, p-value = 0.011) and H-score (r_s = 0.341, p-value < 0.001)

	- *Method of diagnostic:* NR	

NOTE: sums of percentages could differ from 100 due to rounding of numbers.

ABBREVIATIONS: NR = not reported; NA = not applicable; ER = estrogen receptor; PR = progesterone receptor; HER2 = human epidermal growth factor receptor 2; IHC = immunohistochemistry; IRMA = immunoradiometric assay; RIA = radioimmunoassay; IFMA = immunofluorometric assay; OR = odds ratio; *GAPDH = Glyceraldehyde-3-phosphate dehydrogenase*

[A] According to the system adopted by the National Health Service Breast Screening Programme;
[B] Assessed by immunocytochemical method using commercial kits (Abbott Laboratories, Maidenhead, U.K.);
[C] According to WHO, Histological Typing of Breast Tumors, Ed. 2, Geneva, in *Neoplasma*, *30*(1): 113-120, 1982;
[D] According to a modified version of Bloom and Richardson method;
[E] Assessed by standard dextran-coated charcoal assay;
[F] PTHrP and Nuc-pStat5 were evaluable for 92/100 (92%) patients;
[G] Determined using standard IHC;
[H] According to the American Joint Committee on Cancer staging system;
[I] Original cohort composed by 619 patients, PTHrP expression was evaluable in 410 patients only;
[J] Original cohort composed by 540 patients, PTHrP expression was evaluable in 387 patients only;
[K] According to the Elston-Ellis method;
[L] Assessed by immunostaining.
