# Supplementary material for: Clinical and prognostic significance of parathyroid hormone-related protein in breast cancer: a systematic review and meta-analyses of observational studies in women
Source: Endocr Relat Cancer. 2026 Mar 5;33(3):e250324. doi: 10.1530/ERC-25-0324 (PMC12978662; doi:10.1530/ERC-25-0324)
Supplement: Supplementary file 18 [file supplementary_table_10.pdf]

**Supplementary Table 10. Extraction sheet. Association between PTHrP/*PTHLH* expression and breast cancer-associated hypercalcemia.**

| Study | Population | | Tissue samples | | | Statistical modeling | | Study results |
|---|---|---|---|---|---|---|---|---|
| **First author/year/ Country/ Study design** | **N** | **Participant characteristics** | **Diseases characteristics** | **Tissue sample(s) type and preprocessing** | **PTHrP/*PTHLH* measurement(s)** | **Statistical model(s)** | **Adjustment** | **Correlation** |
| Henderson *et al.*, 1990, Canada, cross-sectional | 31 | - *Period of recruitment:* between March and June 1988<br>- *Age:* NR<br>- *Ethnicity:* NR<br>- *Menopausal status:* NR<br>- *Parity status:* NR<br>- *Follow-up:* monthly intervals for 6 months<br>- *Treatment regimen:* NR | **Breast tumor:**<br>- *Stage:* NR<br>- *Grade:* NR<br>- *Histological types:* NR<br>- *Molecular subtypes:*<br>ER+ NR<br>PR+ NR<br>HER2+ NR<br>Ki67+ NR<br><br>**Metastases:**<br>- *N:* 13/31 (42%) bone metastases (11/31, 35% missing)<br>- *Method of diagnostic* means of $[^{99m}$TC]diphosphonate bone scans<br>- *Receptor status:* NR<br><br>**Hypercalcemia:**<br>- *Status:* 10/31 (32%) at time of diagnosis<br>- *Method of diagnostic:* NR<br>- *Subtypes:* NR<br>- *PTH levels:* below the mean normal level in patients with hypercalcemia and malignancy | - *Sample type:* blood (plasma)<br>- *Tumor cells:* NR<br>- *Sampling method:* blood collected in heparinized (143 units per 10ml tubes) vacutainer tubes (Becton Dickinson) and kept at 4°C for up to 2 hours prior to centrifugation. Plasma was then immediately frozen.<br>- *Samples fixation:* NA<br>- *Samples storage:* Samples were stored at -20°C for up to 2 weeks before assay.<br>- *RNA extraction method:* NA<br>- *RNA quality assessment:* NA<br>- *cDNA synthesis method:* NA | - *Measurement method:* RIA<br>- *Antibodies/probes:* antiserum 5087/3 (dilution 1:25,000) raised in rabbits to human PTHrP(1-34)<br>- *Housekeeping gene(s):* NR<br>- *Quantification methods:* continuous, standard using synthetic human PTHrP(1-34)<br>- *Positive controls:* standard using synthetic human PTHrP(1-34)<br>- *Negative controls:* outdated blood bank plasma<br>- *Reproducibility assessments:* samples were assayed in triplicates, interassay coefficient of variation was 19% (*n*=5) and intraassay coefficient of variation was 6% (*n*=10)<br>- *Statistical analysis:* quantitative and qualitative (elevated *versus* not elevated) | NR | *Method of selection:* NR<br>*Selected variables:* NR | 12/31 (39%) patients had elevated PTHrP(1-34)<br><br>Plasma PTHrP(1-34) in hypercalcemic patients (*n*=10) = 0.61 ± 0.17ng equivalents of standard per ml compared to 0.39 ± 0.08ng equivalents of standard per ml<br>In normocalcemic patients<br><br>6/10 (60%) hypercalcemic patients had elevated PTHrP(1-34) compared to 6/21 (29%) normocalcemic patients<br><br>In 2 patients with breast adenocarcinoma metastatic to the liver, treatment with chemotherapy was associated with a decrease in tumor mass, circulating calcium levels and PTHrP(1-34) |
| Southby *et al.*, 1990, Australia, cohort study | 102 | - *Period of recruitment:* August 1987 to March 1989<br>- *Age:*<br>30/102 29% <50 years<br>61/102 60% >51 years<br>11/102 11% missing<br>- *Ethnicity:* NR<br>- *Menopausal status:* NR<br>- *Parity status:* NR<br>- *Follow-up:* NR<br>- *Treatment regimen:* Of the 91 patients who did not present | **Breast tumor:**<br>- *Stage*[A]*:*<br>19/102 19% stage 1<br>46/102 45% stage 2<br>19/102 19% stage 3<br>7/102 7% stage 4<br>11/102 11% missing<br>- *Grade*[B]*:*<br>8/102 8% grade I<br>32/102 31% grade II<br>45/102 44% grade III<br>17/102 17% missing<br>- *Histological types:*<br>52/102 51% infiltrating ductal<br>22/102 22% infiltrating ductal + ductal carcinoma *in situ*<br>5/102 5% infiltrating lobular<br>3/102 3% mucinous | - *Sample type:* tumor<br>- *Tumor cells :* NR<br>- *Sampling method:* surgery or biopsy<br>- *Samples fixation:* fixed in 10% buffered formalin for 12-24 hours and paraffin-embedded<br>- *Samples storage:* NR<br>- *RNA extraction method:* NA<br>- *RNA quality assessment:* NA<br>- *cDNA synthesis method:* NA | - *Measurement method:* IHC<br>- *Antibodies/probes:* New Zealand white rabbits' polyclonal antibody against PTHrP(1-34) (1:25 and 1:50 dilutions)<br>- *Housekeeping gene(s):* NA<br>- *Quantification methods:*<br>• <u>Positive/negative =</u> tumors were called positive if at least 1 tumoral cell was stained<br>• <u>Intensity of staining =</u> no staining of tumor cells (0), weak positive staining (1), moderately strong positive staining (2), strong positive staining (3)<br>• <u>Type of staining =</u> | Frequency data were analyzed by the chi-squared test or Fisher's exact test. Means were compared with Student's *t* test. | *Method of selection:* NR<br>*Selected variables:* NR | In stage 1-3 primary breast cancer 49/84 were PTHrP-positive<br><br>In stage 1-3 primary cancer who developed hypercalcemia (3/84), all (3/3, 100%) had a primary PTHrP positive tumor<br><br>In stage 1-3 primary cancer who did not develop hypercalcemia (81/84), 46/81 (57%) had a PTHrP positive tumor |

| | | | | | |
|---|---|---|---|---|---|
| | with a local recurrence, 78% had a mastectomy, 20% had breast conservation and 2% had biopsy alone. In addition, 85% had an axillary clearance. Patients considered to have a high risk of recurrence were given adjuvant chemotherapy (25%), tamoxifen (29%) and radiotherapy (25%). | 5/102 5% anaplastic<br>4/102 4% tubular/papillary/cribriform<br>11/102 11% missing<br>- *Molecular subtypes:*<br>ER[C]+ 54/102 53% (15/102 15% missing)<br>PR[C]+ 49/102 48% (26/102 25% missing)<br>HER2+ NR<br>Ki67 NR<br><br>**Metastases:**<br>- *N:* 7/102 (7%) stage 4 at time of diagnosis, 17/102 (17%) missing<br>- *Method of diagnostic:* NR<br><br>**Hypercalcemia:**<br>- *Status:* 102/102 100% normocalcemic at time of diagnostic, 3/102 (3%) subsequently developed hypercalcemia<br>- *Method of diagnostic:* NR<br>- *Subtypes:* NR<br>- *PTH levels:* NR | | cytoplasmic pattern, vesicular pattern, mixed pattern<br>• Area of staining = estimate percentage of cells in the section which stained positively, 0-5% of positive tumor cells (1), 5-25% of positive tumor cells (2), 26-50% of positive tumor cells (3) and >50% positive cells (4)<br>• Staining index = area times intensity of staining, weak (1 and 2), moderate (3 and 4) and strong (>6)<br>- *Positive controls:* squamous cell carcinoma of the skin<br>- *Negative/specificity controls:* Alternative deletion of the antibody layers, pre-absorption of the primary antibody with 0.5mg/ml PTHrP(1-34), application of 0.5mg/ml PTHrP(1-34) to the section 5 minutes before the addition of the primary antibody, replacement of the primary antibody with non-immune rabbit serum<br>- *Reproducibility assessments:* Each tumor was assessed independently by two observers with ~95% agreement, when the two were not in concordance, the tumor was re-stained and reassessed by a panel of five observers, including the two initial observers and three observers blinded from clinicopathological data as well as previous results.<br>- *Statistical analysis:* qualitative<br>○ Positive/negative<br>semiquantitative<br>○ Intensity of staining (1, 2, 3)<br>○ Area of staining (<5%, 6-25%, 26-50%, >51%)<br>○ Staining index (weak, moderate, strong)<br>○ Type of staining (cytoplasmic, vesicular, mixed) | |

| | | | | | | | | |
|---|---|---|---|---|---|---|---|---|
| Bundred *et al.*, 1991, UK, cross-sectional study | 98 | - *Period of recruitment:* NR<br>- *Age:* NR<br>- *Ethnicity:* NR<br>- *Menopausal status:* NR<br>- *Parity status:* NR<br>- *Follow-up:* NR<br>- *Treatment regimen:* NR | **Breast tumor:**<br>- *Stage:*<br>57/98 (58%) early operable breast cancer without apparent bone metastases<br>41/98 (42%) stage 4<br>- *Grade:* NR<br>- *Histological types:* NR<br>- *Molecular subtypes:*<br>ER+ NR<br>PR+ NR<br>HER2+ NR<br>Ki67+ NR<br><br>**Metastases:**<br>- *N:* 41/98 (42%) bone metastases<br>- *Method of diagnostic :* NR<br>- *Bone markers:* NR<br>- *Subtypes:* NR<br><br>**Hypercalcemia:**<br>- *Status :* 13/98 (13%) hypercalcemic (100% had bone metastases)<br>- *Method of diagnostic :* NR<br>- *Subtypes:* NR<br>- *PTH levels:* reference range (9-40ng/L)<br>9/10 patients with bone metastases and detectable PTHrP in whom PTH has been measured, the mean concentration was 23 (range 8-32) ng/L. In 9 hypercalcemic patients, plasma PTH concentration was <5ng/L in 4/9 and 5/9 had a concentration of 11 (range 7-17) ng/L. | - *Sample type:* blood (plasma)<br>- *Tumor cells :* NA<br>- *Sampling method:* NR<br>- *Samples fixation:* NA<br>- *Samples storage:* NR<br>- *RNA extraction method:* NA<br>- *RNA quality assessment:* NA<br>- *cDNA synthesis method:* NA | - *Measurement method:* two site IRMA (INCSTAR, Wokingham, Berkshire)<br>- *Antibodies:* anti-PTHrP(1-86)<br>- *Housekeeping gene(s):* NA<br>- *Quantification methods:* continuous, concentrations are undetectable ≤0.23pmol/L<br>- *Positive controls:* patients with cancer-related (non-breast cancer) hypercalcemia<br>- *Negative controls:* normocalcemic controls<br>- *Reproducibility assessments:* NR<br>- *Statistical analysis:* qualitative (positive *versus* negative) and quantitative | Mann-Whitney U test | *Method of selection:* NR<br>*Selected variables:* NR | 41/98 (42%) patients had bone metastases<br><br>28/41 (68%) had bone metastases without hypercalcemia and 13/41 (32%) had bone metastases + hypercalcemia<br><br>- 12/13 (92%) of breast cancer patients with hypercalcemia and bone metastases were PTHrP-positive (mean = 3.7pmol/L; range 0.54-8.1pmol/L) *vs* 10/28 (36%) of normocalcemic breast cancer patients with bone metastases (mean = 0.59pmol/L; range 0.23-1.50pmol/L, p<0.001)<br><br>PTHrP was also found in higher concentration in breast cancer patients with hypercalcemia and bone metastases (mean = 3.7pmol/L; range 0.54-8.1pmol/L) than in patients with early breast cancer (mean = 0.33pmol/L; range 0.25-0.46pmol/L, p<0.01) |
| | 178 | - *Period of recruitment:* NR<br>- *Age:* NR<br>- *Ethnicity:* NR<br>- *Menopausal status:* NR<br>- *Parity status:* NR<br>- *Follow-up:* NR<br>- *Treatment regimen:* NR | **Breast tumor:**<br>- *Stage:* NR<br>- *Grade:* NR<br>- *Histological types:* NR<br>- *Molecular subtypes:*<br>ER+ NR<br>PR+ NR<br>HER2+ NR<br>Ki67+ NR<br><br>**Metastases:**<br>- *N:* 72/178 (40%) with bone metastases as initial site of metastases<br>- *Method of diagnostic :* NR<br>- *Bone markers:* NR<br>- *Subtypes:* NR<br><br>**Hypercalcemia:** | - *Sample type:* tumor<br>- *Tumor cells :* NR<br>- *Sampling method:* NR<br>- *Samples fixation:* 4% formaldehyde in saline fixation and paraffin embedding<br>- *Samples storage:* NR<br>- *RNA extraction method:* NA<br>- *RNA quality assessment:* NA<br>- *cDNA synthesis method:* NA | - *Measurement method:* IHC<br>- *Antibodies:* rabbit antibody against mid-region sequence of PTHrP(37-67)<br>- *Housekeeping gene(s):* NA<br>- *Quantification methods:* only cytoplasmic reactivity was considered positive<br>- *Positive controls:* squamous carcinoma of lung<br>- *Negative controls:* omission of the primary antibody, preincubation of the primary antibody with PTHrP(37-67) for 16 hours at 4°C and non-immune serum as replacement of the primary antibody<br>- *Reproducibility assessments:* staining of tumors was | Fisher's exact test | *Method of selection:* NR<br>*Selected variables:* NR | - 22/25 (88%) of breast cancers with bone metastases and hypercalcemia were PTHrP-positive *vs* 25/47 (53%) of breast cancers with bone metastases without hypercalcemia p=0.004 |

| | | | | | | | | |
|---|---|---|---|---|---|---|---|---|
| | | | - *Status :* 178/178 (100%) normocalcemic at time of diagnostic, 25/178 (14%) subsequently developed hypercalcemia (100% had bone metastases)<br>- *Method of diagnostic:* corrected calcium range 2.7-3.9 mmol/l)<br>- *Subtypes:* NR<br>- *PTH levels:* NR | | assessed by two independent observers (blinded)<br>- *Statistical analysis:* qualitative (positive *versus* negative) | | | |
| Blind *et al.*, 1992, Germany, cohort study | 14 | - *Period of recruitment:* September 1989 to March 1991<br>- *Age:* NR<br>- *Ethnicity:* NR<br>- *Menopausal status:* NR<br>- *Parity status:* NR<br>- *Follow-up:* NR<br>- *Treatment regimen:* several patients received therapy to reduce serum calcium, but all patients were still hypercalcemic when blood was collected for PTH and PTHrP measurements | **Breast tumor:**<br>- *Stage:* NR<br>- *Grade:* NR<br>- *Histological types:* NR<br>- *Molecular subtypes:*<br>ER+ NR<br>PR+ NR<br>HER2+ NR<br>Ki67+ NR<br><br>**Metastases:**<br>- *N:* 5/5 patients with breast cancer had bone metastases<br>- *Method of diagnostic:* X-ray and/or bone scan<br>- *Bone markers:* NR<br>- *Subtypes:* NR<br><br>**Hypercalcemia:**<br>- *Status:* 100%<br>- *Method of diagnostic:* serum total calcium concentration > 2.70 mmol/l on 2 occasions<br>- *PTH levels:* the population was controlled for hyperparathyroidism. Patients with abnormal circulating intact PTH were excluded. | - *Sample type:* blood<br>- *Tumor cells :* NA<br>- *Sampling method:* blood samples were collected at or 1 day following admission at the hospital<br>- *Sample fixation:* NA<br>- *Samples storage:* 4°C<br>- *RNA extraction method:* NA<br>- *RNA quality assessment:* NA<br>- *cDNA synthesis method:* NA | - *Measurement method:* RIA<br>- *Antibodies/probes:* antiserum against PTHrP(53-84) and PTHrP(1-86) as label and standard<br>- *Housekeeping gene(s):* NA<br>- *Quantification methods:* quantitative (pmol/l), then divided in 2 groups:<br>High PTHrP: PTHrP higher than the upper limit of the healthy group (21pmol/l, *n*=87)<br>Low PTHrP: : PTHrP levels lower than the upper limit of the healthy group (21pmol/l, *n*=87)<br>- *Positive controls:* standard curve, supernatants from cell cultures of the hypercalcemia-inducing Walker carcinosarcoma 256, 4 samples of human breast milk from lactating women<br>- *Negative/specificity controls:* NR<br>- *Reproducibility assessments:* repeated measurements of pooled serum samples from several healthy subjects and patients. Interassay variation = 20.3% at 9.7 pmol/l, 11.4% at 36 pmol/l and 4.2% at 76 pmol/l (*n* = 6)<br>- *Statistical analysis:* qualitative | No specific analysis for the subgroup included in this systematic review. | *Method of selection:* NR<br>*Selected variables:* NR | 5/5 (100%) hypercalcemic patients with breast carcinoma and bone metastases had elevated PTHrP<br><br>0/9 (0%) normocalcemic breast cancer patients had elevated PTHrP |
| Bundred *et al.*, 1992, UK, cohort study | 155 | - *Period of recruitment:* August 1984 to December 1985<br>- *Age:*<br>65/155 42% ≤ 50 years<br>90/155 58% ≥ 51 years<br>- *Ethnicity:* NR<br>- *Menopausal status:* | **Breast tumor:**<br>- *Stage:* NR<br>- *Grade*[D]*:*<br>30/155 19% grade I<br>88/155 57% grade II<br>37/155 24% grade III<br>- *Histological types:*<br>126/155 81% infiltrating ductal<br>29/155 19% other types<br>- *Molecular subtypes:*<br>ER[E]+ 64/155 41% (55/155 | - *Sample type:* tumor<br>- *Tumor cells :* NR<br>- *Sampling method:* surgery<br>- *Sample fixation:* 4% formaldehyde in saline and paraffin embedding<br>- *Samples storage:* NR<br>- *RNA extraction* | - *Measurement method:* IHC<br>- *Antibodies/probes:* rabbit anti PTHrP(37-67)<br>- *Housekeeping gene(s):* NA<br>- *Quantification methods:* only cytoplasmic staining was considered<br>- *Positive controls:* squamous carcinoma of the lung included in each batch of slides<br>- *Negative/specificity controls:* preincubation of the primary | NR | *Method of selection:* NR<br>*Selected variables:* NR | 87/155 (56%) breast cancer patients had a PTHrP-positive primary tumor and 21/28 (75%) breast cancer patients who subsequently developed bone metastases<br><br>6/28 (21%) patients who subsequently developed bone metastases also |

| | | | | | | | | |
|---|---|---|---|---|---|---|---|---|
| | | 43/155 28% premenopausal<br>112/155 72% postmenopausal<br>- *Parity status:* NR<br>- *Follow-up:* ≥ 5 years or until death, all patients were seen every 4 months in the first year after surgery and thereafter at 6-month intervals<br>- *Treatment regimen:* mastectomy with axillary clearance or node sampling (97/155, 63%), or breast conserving operations and radiotherapy (58/155, 37%) | 35% missing)<br>PR[E]+ 55/155 35% (69/155 45% missing)<br>HER2+ NR<br>Ki67+ NR<br><br>**Metastases:**<br>- *N:* none of the patients had clinical evidence of bone metastases at presentation<br>- *Method of diagnostic:* bone scan/scintigraphy, conventional radiography, ultrasound and/or surgical biopsy<br>- *Bone markers:* NR<br>- *Subtypes:* NR<br><br>**Hypercalcemia:**<br>- *Status:* 155/155 100% normocalcemic at time of diagnostic<br>- *Method of diagnostic:* the upper limit of the reference range for corrected calcium was 2.65mmol/l<br>- *PTH levels:* NR | *method:* NA<br>- *RNA quality assessment:* NA<br>- *cDNA synthesis method:* NA | antibody with 0.5mg/ml PTHrP(37-67), omission of the primary antibody, primary antibody replaced by non-immune serum<br>- *Reproducibility assessments:* staining was assessed by two independent observers<br>- *Statistical analysis:* qualitative (positive *versus* negative) | | | developed hypercalcemia, 6/6 (100%) had PTHrP-positive primary tumor |
| Francini *et al.*, 1993, Italy, cross-sectional study | 24 | - *Period of recruitment:* since 1987<br>- *Age:* NR<br>- *Ethnicity:* NR<br>- *Menopausal status:* NR<br>- *Parity status:* NR<br>- *Follow-up:* NR<br>- *Treatment regimen:* NR | **Breast tumor:**<br>- *Stage:* NR<br>- *Grade:* NR<br>- *Histological types:* ductal or lobular carcinoma<br>- *Molecular subtypes:*<br>ER+ NR<br>PR+ NR<br>HER2+ NR<br>Ki67+ NR<br><br>**Metastases:**<br>- *N:* 22/24 (92%) bone metastases<br>- *Method of diagnostic :* bone scan, skeletal survey, and bone biopsy<br>- *Bone markers:* serum alkaline phosphatase and osteocalcin, urinary excretion of calcium, phosphate and hydroxyproline<br>- *Subtypes:* NR<br><br>**Hypercalcemia:**<br>- *Status:* 24/24 (100%) hypercalcemia<br>- *Method of diagnostic :* measurement of calcium content in urine and blood, normal calcium values were 2.10-2.50mmol/L<br>- *Subtypes:* | - *Sample type:* blood (plasma) and urine<br>- *Tumor cells :* NA<br>- *Sampling method:* samples were collected at 8AM after an overnight fast, all samples were collected before hydration and before specific treatment<br>- *Samples fixation:* NA<br>- *Samples storage :* -20°C until the day of assay<br>- *RNA extraction method:* NA<br>- *RNA quality assessment:* NA<br>- *cDNA synthesis method:* NA | - *Measurement method:* RIA (Immuno Technology Service Productions)<br>- *Antibodies:* NR<br>- *Quantification methods:* continuous, pg/ml<br>- *Housekeeping gene(s):* NA<br>- *Positive controls:* serum from 20 normal subjects and 6 hypercalcemic patients with hyperparathyroidism<br>- *Negative/specificity controls:* human PTH(1-34) and bovine PTH(1-84) (Sigma), no cross-reactivity<br>- *Reproducibility assessments:* intra- and inter-assay coefficients of variation (5% and 8% respectively)<br>- *Statistical analysis:* quantitative | Independent two-sided t-tests | *Method of selection:* NR<br>*Selected variables:* NR | PTHrP was higher in patients with humoral hypercalcemia and bone metastases (218.00 ± 148.30 pg/ml) compared to patients with cellular mediated hypercalcemia and bone metastases (39.16 ± 21.75 pg/ml; $p<0.001$) or patients with hypercalcemia but without bone metastases (43.00 pg/ml) |

| | | | | | | | | |
|---|---|---|---|---|---|---|---|---|
| | | | 10/24 (42%) humoral hypercalcemia + bone metastases<br>12/24 (50%) cellular mediated hypercalcemia and bone metastases<br>2/24 (8%) hypercalcemia without bone metastases<br>- *PTH levels:* intact PTH, C-terminal PTH and mid region PTH were in normal ranges | | | | | |
| Kissin *et al.*, 1993, Australia, cohort study | 82 | - *Period of recruitment:* between 1984 and 1987<br>- *Age:* mean =<br>14/82 17% <50 years<br>68/82 83% >50 years<br>- *Ethnicity:* NR<br>- *Menopausal status:* NR<br>- *Parity status:* NR<br>- *Follow-up:* at least 3 years<br>- *Treatment regimen:*<br>9/82, 11% needle biopsy<br>15/82, 18% wide local excision<br>58/82, 71% total mastectomy<br><br>63/82, 77% axillary dissection<br><br>22/82, 27% adjuvant tamoxifen<br>16/82, 20% chemotherapy 27/82, 33% radiotherapy | **Breast tumor:**<br>- *Stage*[F]:<br>8/82 10% stage 1<br>40/82 49% stage 2<br>8/82 10% stage 3<br>26/82 32% stage 4<br><br>- *Grade:* NR<br>- *Histological types:* NR<br>- *Molecular subtypes:*<br>ER[G]+ 53/82 65%<br>PR[G]+ 47/82 57%<br>HER2+ NR<br>Ki67+ NR<br><br>**Metastases:**<br>- *N:* 26/82, 32% patients had metastases at time of initial diagnosis<br>- *Method of diagnostic:* chest X-ray and bone scan<br><br>**Hypercalcemia:**<br>- *Status:* 13/82, 16% patients developed symptomatic hypercalcemia and/or prolonged hypercalcemia during the period of follow up.<br>- *Method of diagnostic:* NR<br>- *PTH levels:* NR | - *Sample type:* tumor<br>- *Tumor cells :* NR<br>- *Sampling method:* surgery<br>- *Samples fixation:* 10% buffered formalin for 12 to 24 hours and embedded in paraffin<br>- *Samples storage:* NR<br>- *RNA extraction method:* NA<br>- *RNA quality assessment:* NA<br>- *cDNA synthesis method:* NA | - *Measurement method:* IHC<br>- *Antibodies/probes:* rabbit polyclonal antibody against PTHrP(1-34)<br>- *Housekeeping gene(s):* NA<br>- *Quantification methods:* A tumor was called positive for PTHrP if any of the tumor cells were specifically stained brown (cytoplasmic and/or vesicular).<br>• Localization = cytoplasmic, vesicular, mixed<br>• Intensity (subjective) = weak (1), moderate (2), strong (3)<br>• Area of staining (percent positive tumor cells) = <5% (1), 6-25% (2), 26-50% (3), >50% (4)<br>• Staining index (intensity x area of staining) = 1 and 2 (weak), 3 and 4 (moderate), >6 (strong)<br>- *Positive controls:* squamous cell carcinoma of the skin<br>- *Negative/specificity controls:* alternate deletions of the primary antiserum, secondary antibody and peroxidase anti-peroxidase complex, pre-absorption of the anti-PTHrP(1-34) antiserum with 0.5 mg/ml PTHrP(1-34) overnight at 4C, sections of breast tumors and squamous cell carcinoma of the skin where the specific antiserum was replaced with non-immune rabbit serum<br>- *Reproducibility assessments:* each tumor was stained twice, and in duplicate, evaluation of staining was done by 1 the principal investigator and subsequently by a panel of 4 observers with no knowledge | Frequency data were analyzed with the chi-squared test and the Fisher's exact test when numbers were less than 20. Means were compared with Student's *t* test. | *Method of selection:* NR<br>*Selected variables:* NR | 53/82 (63%) patients had a PTHrP-positive tumor<br>35/82 patients had or developed bone metastases<br><br>35/52 patients with metastases had bone metastases<br><br>13/82 (16%) patients developed hypercalcemia during follow-up<br>10/13 (77%) had bone metastases, 3/13 (23%) had soft tissue sites metastases<br><br>9/10 (90%) patients with hypercalcemia and bone metastases had positive PTHrP tumors<br>1/3 (33%) patients with hypercalcemia and soft tissue sites metastases had PTHrP positive tumors<br>P = 0.11 |

| | | | | | | | | |
|---|---|---|---|---|---|---|---|---|
| | | | | | of the patient data. If there was a disagreement between observers, the tumor was restained and reassessed.<br>- *Statistical analysis:* semiquantitative<br>○ Intensity of staining (1, 2, 3)<br>○ Area of staining (<5%, 6-25%, 26-50%, >51%)<br>○ Staining index (weak, moderate, strong) | | | |
| Bundred *et al.*, 1996, UK, cohort study | 155 +72 (227) | - *Period of recruitment:* NR<br>- *Age:* NR<br>- *Ethnicity:* NR<br>- *Menopausal status:* NR<br>- *Parity status:* NR<br>- *Follow-up:* NR<br>- *Treatment regimen:* NR | **Breast tumor:**<br>- *Stage:* NR<br>- *Grade:* NR<br>- *Histological types:* NR<br>- *Molecular subtypes:*<br>ER+ NR<br>PR+ NR<br>HER2+ NR<br>Ki67+ NR<br><br>**Metastases:**<br>- N: 155/227 (68%) early breast cancer without metastasis at time of diagnostic, 72/227 (32%) primary breast cancers which had developed bone metastases<br>28/155 (18%) early breast cancer without metastasis at time of diagnostic have subsequently developed bone metastases<br>- *Method of diagnostic:* NA<br>- *Receptor status:* NA<br><br>**Hypercalcemia:**<br>- *Status:* 25/72 (35%) patients with primary breast cancer and bone metastases subsequently developed hypercalcemia, 5/155 (3%) patients with early breast cancer without bone metastasis subsequently developed hypercalcemia.<br>- *Method of diagnostic:* NR<br>- *PTH levels:* NR | - *Sample type:* tumor<br>- *Tumor cells :* NR<br>- *Sampling method:* NR<br>- *Sample fixation:* paraffin-fixed sections<br>- *Samples storage:* NR<br>- *RNA extraction method:* NA<br>- *RNA quality assessment:* NA<br>- *cDNA synthesis method:* NA | - *Measurement method:* IHC<br>- *Antibodies/probes:* polyclonal antibody against PTHrP(34-67)<br>- *Housekeeping gene(s):* NA<br>- *Quantification methods:* NR<br>- *Positive controls:* NR<br>- *Negative controls:* NR<br>- *Reproducibility assessments:* NR<br>- *Statistical analysis:* NR | NR | *Method of selection:* NR<br>*Selected variables:* NR | In patients with bone metastases 25/72 (35%) developed hypercalcemia, and 22/25 (88%) had PTHrP staining in their primary tumor ($P \leq 0.002$)<br><br>Out of the 155 early breast cancer patients who have been followed up for a minimum of 5 years, 6 developed hypercalcemia, and 6/6 (100%) had PTHrP-positive tumors |

| | | | | | | | |
|---|---|---|---|---|---|---|---|
| 195 +72 + 31 (298) | - *Period of recruitment:* NR<br>- *Age:* NR<br>- *Ethnicity:* NR<br>- *Menopausal status:* NR<br>- *Parity status:* NR<br>- *Follow-up:* NR<br>- *Treatment regimen:* NR | **Breast tumor:**<br>- *Stage:* NR<br>- *Grade:* NR<br>- *Histological types:* NR<br>- *Molecular subtypes:*<br>ER+ NR<br>PR+ NR<br>HER2+ NR<br>Ki67+ NR<br><br>**Metastases:**<br>- *N:* 195/298 (65%) early breast cancer, 72/298 (24%) bone metastases patients, 31/298 (10%) patients with hypercalcemia of malignancy owing to breast cancer<br>- *Method of diagnostic:* NA<br>- *Bone markers:* NR<br>- *Subtypes:* NR<br><br>**Hypercalcemia:**<br>- *Status:* 31/298 (10%) patients with hypercalcemia of malignancy owing to breast cancer<br>- *Method of diagnostic :* NR<br>- *PTH levels:* NR | - *Sample type:* blood (plasma)<br>- *Tumor cells:* NR<br>- *Sampling method:* NR<br>- *Sample fixation:* NA<br>- *Samples storage:* NR<br>- *RNA extraction method:* NA<br>- *RNA quality assessment:* NA<br>- *cDNA synthesis method:* NA | - *Measurement method:* NR<br>- *Antibodies/probes:* NR<br>- *Housekeeping gene(s):* NR<br>- *Quantification methods:* NR<br>- *Positive controls:* NR<br>- *Negative controls:* NR<br>- *Reproducibility assessments:* NR<br>- *Statistical analysis:* NR | NR | *Method of selection:* NR<br>*Selected variables:* NR | 19/195 (10%) early breast cancer patients had detectable PTHrP and 25/72 (35%) patients with bone metastases for a total of 44/267 (16%) normocalcemic patients with breast cancer.<br><br>On the other hand, 26/31 (84%) women with hypercalcemia of malignancy owing to breast cancer were PTHrP-positive ($P \leq 0.001$) |
| 185 + 72 + 31 (288) | - *Period of recruitment:* NR<br>- *Age:* NR<br>- *Ethnicity:* NR<br>- *Menopausal status:* NR<br>- *Parity status:* NR<br>- *Follow-up:* NR<br>- *Treatment regimen:* NR | **Breast tumor:**<br>- *Stage:* NR<br>- *Grade:* NR<br>- *Histological types:* NR<br>- *Molecular subtypes:*<br>ER+ NR<br>PR+ NR<br>HER2+ NR<br>Ki67+ NR<br><br>**Metastases:**<br>- *N:* 185/288 (64%) early breast cancer, 72/288 (25%) bone metastases patients, 31/288 (11%) patients with hypercalcemia of malignancy owing to breast cancer<br>- *Method of diagnostic:* NA<br>- *Bone markers:* NR<br>- *Subtypes:* NR<br><br>**Hypercalcemia:**<br>- *Status:* 31/288 (11%) patients with hypercalcemia of malignancy owing to breast cancer<br>- *Method of diagnostic :* NR<br>- *PTH levels:* NR | - *Sample type:* blood (plasma)<br>- *Tumor cells:* NR<br>- *Sampling method:* NR<br>- *Sample fixation:* NA<br>- *Samples storage:* NR<br>- *RNA extraction method:* NA<br>- *RNA quality assessment:* NA<br>- *cDNA synthesis method:* NA | - *Measurement method:* NR<br>- *Antibodies/probes:* targeting PTHrP(1-86)<br>- *Housekeeping gene(s):* NR<br>- *Quantification methods:* NR<br>- *Positive controls:* NR<br>- *Negative controls:* NR<br>- *Reproducibility assessments:* NR<br>- *Statistical analysis:* NR | NR | *Method of selection:* NR<br>*Selected variables:* NR | The median level of PTHrP detectable was significantly higher in the hypercalcemic patients (mean 5.6 pmol/l, range 0.23 to 66pmol/l) compared with normocalcemic patients (with or without bone metastases, mean = 0.46, range 0.23 to 1.7) |

| | | | | | | | | |
|---|---|---|---|---|---|---|---|---|
| LaMaute *et al.*, 1996, USA, cross-sectional study | 9 | - *Period of recruitment:* calendar years 1992 and 1993<br>- *Age:* range 36 to 84 years<br>- *Ethnicity:* NR<br>- *Menopausal status:* NR<br>- *Parity status:* NR<br>- *Follow-up:* NR<br>- *Treatment regimen:* NR | **Breast tumor:**<br>- *Stage:*<br>1/9 (11%) I<br>5/9 (56%) IIA<br>3/9 (33%) IIIB<br>- *Grade:* NR<br>- *Histological types:*<br>8/9 (89%) carcinoma<br>1/9 (11%) malignant cystosarcoma phylloides<br>- *Molecular subtypes:*<br>ER+ NR<br>PR+ NR<br>HER2+ NR<br>Ki67+ NR<br><br>**Metastases:**<br>- *N:* 0/9, 0%<br>- *Method of diagnostic:* NR<br>- *Receptor status:* NR<br>- *Bone markers:* NR<br>- *Subtypes:* NR<br><br>**Hypercalcemia:**<br>- *Status:* 1/9 (11%) hypercalcemic<br>- *Method of diagnostic:* serum calcium determination was performed by the standard technique<br>- *PTH levels:* NR | - *Sample type:* blood (plasma)<br>- *Tumor cells:* NR<br>- *Sampling method:* specimens for PTHrP assay were obtained pre-operatively, placed in a special tube, gently inverted twice and centrifuged immediately. Plasma was transferred to a special plastic tube from the Nichols Institute and frozen<br>- *Sample fixation:* NA<br>- *Samples storage:* NR<br>- *RNA extraction method:* NA<br>- *RNA quality assessment:* NA<br>- *cDNA synthesis method:* NA | - *Measurement method:* 2-site IRMA, modified from Pandian Morgan *et al.*, 1992<br>- *Antibodies/probes:* anti-PTHrP(37-74) as the capture antibody and a radiolabeled anti-PTHrP(1-36) as signal antibody<br>- *Housekeeping gene(s):* NR<br>- *Quantification methods:* continuous, in pmol/L<br>- *Positive controls:* NR<br>- *Negative controls:* NR<br>- *Reproducibility assessments:* NR<br>- *Statistical analysis:* qualitative (detectable *versus* undetectable) and quantitative | NR | *Method of selection:* NR<br>*Selected variables:* NR | 1/9 (11%) patient was hypercalcemic without bone metastases (serum calcium: 12.3) and she also has a raised plasma PTHrP (4.7pmol/L).<br><br>All other subjects had undetectable levels of PTHrP (below 0.2pmol/l) |
| Nagasaki *et al.*, 1996, Japan, cross-sectional study | 6 | - *Period of recruitment:*<br>- *Age:* mean = 43.7 ± 9.54<br>- *Ethnicity:* NR<br>- *Menopausal status:* NR<br>- *Parity status:* NR<br>- *Follow-up:* NR<br>- *Treatment regimen:* NR | **Breast tumor:**<br>- *Stage:* NR<br>- *Grade:* NR<br>- *Histological types:* NR<br>- *Molecular subtypes:* NR<br><br>**Metastases:**<br>- *N:* 3/6 (50%) bone metastases<br>- *Method of diagnostic :* bone scintigraphy and/or bone roentgenography<br>- *Bone markers:* NR<br>- *Subtypes:* NR<br><br>**Hypercalcemia:**<br>- *Status:* 6/6, 100% hypercalcemic<br>- *Method of diagnostic :* corrected serum calcium > 10.5 mg/dl<br>- *Subtypes:* NR<br>- *PTH levels:* NR | - *Sample type:* blood (serum)<br>- *Tumor cells :* NA<br>- *Sampling method:* serum samples were collected without protease inhibitors<br>- *Sample fixation:* NA<br>- *Samples storage:* collected serum samples were stored at -20°C<br>- *RNA extraction method:* NA<br>- *RNA quality assessment:* NA<br>- *cDNA synthesis method:* NA | - *Measurement method:* RIA<br>- *Antibodies:* antiserum against human PTHrP(109-141) (Daiichi Radioisotope Laboratories, Ltd., Tokyo) and $^{108}$Tyr-PTHrP(108-141) as the tracer<br>- *Quantification methods:* continuous (pmol/L)<br>- *Positive controls:* NR<br>- *Negative controls:* NR<br>- *Reproducibility assessments:* NR<br>- *Statistical analysis:* quantitative | NR | *Method of selection:* NR<br>*Selected variables:* NR | Mean serum PTHrP (pmol/l) in all patients with breast cancer and hypercalcemia (*n*=6) = 321.2 ± 557.2<br><br>Mean serum PTHrP (pmol/l) in patients with HHM (no bone metastases, *n*=3) = 587.8 ± 750.2<br><br>Mean serum PTHrP (pmol/l) in patients with bone metastases (*n*=3) = 54.53 ± 15.76 |

| | | | | | | | | |
|---|---|---|---|---|---|---|---|---|
| Mawer *et al.*, 1997, UK, cross-sectional study | 129 | - *Period of recruitment:* NR<br>- *Age:* NR<br>- *Ethnicity:* NR<br>- *Menopausal status:* NR<br>- *Parity status:* NR<br>- *Follow-up:* NR<br>- *Treatment regimen:* No treatment or adjuvant tamoxifen. Patients with tamoxifen adjuvant therapy were changed to megesterol if disease progressed | **Breast tumor:**<br>- *Stage:*<br>88/129 68% operable early breast cancer<br>41/129 32% stage 4<br>- *Grade:* NR<br>- *Histological types:* NR<br>- *Molecular subtypes:*<br>ER+ NR<br>PR+ NR<br>HER2+ NR<br>Ki67+ NR<br><br>**Metastases:**<br>- *N:* 88/129, 68% patients had operable early breast cancers without metastases, 41/129, 32% had bone metastases<br>- *Method of diagnostic:* bone scan or plain x-rays<br><br>**Hypercalcemia:**<br>- *Status:* 12/129, 9% had bone metastases with hypercalcemia<br>- *Method of diagnostic :* hypercalcemia was defined as a serum calcium >2.6mmol/L when adjusted for serum albumin<br>- *Subtypes:* NR<br>- *PTH levels:* PTH concentration did not differ between early breast cancer patients with raised 1,25-$(OH)_2$D (25.2 range 17-47ng/L) and those with normal 1,25-$(OH)_2$D (28.6 range 6-56ng/L) | - *Sample type:* blood (plasma)<br>- *Tumor cells:* NR<br>- *Sampling method:* NR<br>- *Sample fixation:* NA<br>- *Samples storage:* NR<br>- *RNA extraction method:* NA<br>- *RNA quality assessment:* NA<br>- *cDNA synthesis method:* NA | - *Measurement method:* IRMA<br>- *Antibodies/probes:* targeting PTHrP(1-86)<br>- *Housekeeping gene(s):* NR<br>- *Quantification methods:* continuous, normal = limit of detection ≤0.23pmol/l<br>- *Positive controls:* NR<br>- *Negative controls:* NR<br>- *Reproducibility assessments:* NR<br>- *Statistical analysis:* quantitative | Statistical significance of differences between groups were assessed as appropriate by Student's paired or unpaired t test or by the Mann-Whitney or Wilcoxon tests. | *Method of selection:* NR<br>*Selected variables:* NR | PTHrP was detectable in 1/29 (3%, 1.20pmol/L) women with bone metastases and eucalcemia and in 11/12 (92%, median = 2.14pmol/L, range 0.46-20.74)) women with bone metastases and hypercalcemia. |
| Pyke *et al.*, 1997, Australia, cross-sectional study | 33 | - *Period of recruitment:* patients already receiving treatment or palliation for advanced breast cancer between December 1992 and May 1993<br>- *Age:* median = 60, range 34-96<br>- *Ethnicity:* NR<br>- *Menopausal status:* NR<br>- *Parity status:* NR<br>- *Follow-up:* NR<br>- *Treatment regimen:* NR | **Breast tumor:**<br>- *Stage:* 33/33 100% stage 4<br>- *Grade:* NR<br>- *Histological types:*<br>24/33 (73%) invasive ductal carcinoma<br>9/33 (27%) missing<br>- *Molecular subtypes:*<br>ER+ NR<br>PR+ NR<br>HER2+ NR<br>Ki67+ NR<br><br>**Metastases:**<br>- *N:*<br>17/33 (52%) bone metastases only<br>8/33 (24%) bone and visceral metastases<br>4/33 (12%) liver | - *Sample type:* blood (serum)<br>- *Tumor cells:* NR<br>- *Sampling method:* NR<br>- *Sample fixation:* NA<br>- *Samples storage:* NR<br>- *RNA extraction method:* NA<br>- *RNA quality assessment:* NA<br>- *cDNA synthesis method:* NA | - *Measurement method:* two-sites IRMA (Allegro PTHrP Immunoassay kit: Nichols Institute Diagnostics, CA, USA) in tubes containing EDTA and the antiproteases aprotinin, lupeptin and pepstatin<br>- *Antibodies/probes:* polyclonal antiserum against PTHrP(1-40) and PTHrP(60-72)<br>- *Housekeeping gene(s):* NA<br>- *Quantification methods:* categorical, samples with >2.6pmol/L were considered "abnormal"<br>- *Positive controls:* NR<br>- *Negative controls:* NR<br>- *Reproducibility assessments:* NR | Fisher's exact test for discrete variables after dividing the groups into "normal" versus "abnormal" results using the reference range (2.6pmol/L) | *Method of selection:* NR<br>*Selected variables:* NR | 8/33 (24%) had hypercalcemia 6/33 (18%) patients with elevated PTHrP, 4/6 (67%) had hypercalcemia (all had normal PTH levels). 4/6 (67%) patients had bone metastases, 1/6 (17%) had peritoneal metastases and 1/6 (17%) had liver metastases.<br><br>4/8 (50%) patients with hypercalcemia had elevated PTHrP (all had normal PTH levels) |

| | | | | | | | | |
|---|---|---|---|---|---|---|---|---|
| | | | metastases<br>8/33 (24%) lung metastases<br>6/33 (18%) others<br>- *Method of diagnostic:* clinical grounds, along with chest X-ray, liver ultrasound and bone scan<br>- *Receptor status:* NR<br><br>**Hypercalcemia:**<br>- *Status:* 8/33 (24%) hypercalcemic<br>- *Method of diagnostic :* defined as corrected calcium serum levels >2.6mmol/L<br>- *Subtypes:* NR<br>- *PTH levels:* considered elevated if PTH >7.0pmol/L<br><br>6/6 (100%) patients with elevated PTHrP had normal PTH<br><br>7/33 (21%) had elevated PTH level, 6/7 (86%) were normocalcemic, none of them had elevated PTHrP. | | - *Statistical analysis:* qualitative (normal *versus* abnormal) | | | |
| De Miguel *et al.*, 1998, Spain, cross-sectional study | 4 | - *Period of recruitment:* NR<br>- *Age:* NR<br>- *Ethnicity:* NR<br>- *Menopausal status:* NR<br>- *Parity status:* NR<br>- *Follow-up:* NR<br>- *Treatment regimen:* no patients had received therapy for their hypercalcemia at the initiation of the study | **Breast tumor:**<br>- *Stage:* NR<br>- *Grade:* NR<br>- *Histological types:* NR<br>- *Molecular subtypes:*<br>ER+ NR<br>PR+ NR<br>HER2+ NR<br>Ki67+ NR<br><br>**Metastases:**<br>- *N:* NR<br>- *Method of diagnostic:* bone scan<br>- *Receptor status:* NR<br><br>**Hypercalcemia:**<br>- *Status:* 2/4 (50%) hypercalcemic<br>- *Method of diagnostic :* defined as corrected serum calcium ≥2.7mmol/L<br>- *Subtypes:* NR<br>- *PTH levels:* normal range of 0.8-6.9 pmol/L<br><br>Intact PTH levels in normocalcemic patients (*n*=2) were 6.6 and 4.1 pmol/L while in hypercalcemic patients (*n*=2) they were 8.4 and 12.7 pmol/L | - *Sample type:* blood (plasma)<br>- *Tumor cells:* NR<br>- *Sampling method:* samples were collected in ice-cold tubes containing, per mL: 1μmol EDTA, 500 kallikrein inhibition units (aprotinin), 5μg leupeptin, 5μg pepstatin and immediately centrifuged<br>- *Sample fixation:* NA<br>- *Samples storage:* plasma was stored at -70°C<br>- *RNA extraction method:* NA<br>- *RNA quality assessment:* NA<br>- *cDNA synthesis method:* NA | - *Measurement method:* two-sites IRMA<br>○ PTHrP-N (Nichols, San Juan Capistrano, CA, USA)<br>○ PTHrP-I (INCSTAR, Stillwater, MN, USA)<br>- *Housekeeping gene(s):* NR<br>- *Quantification methods:*<br>○ PTHrP-N : affinity-purified antibodies directed to PTHrP(1-40) and PTHrP(60-72) and PTHrP(1-86) as standard (detection limit = 0.3pmol/L)<br>○ PTHrP-I : two affinity- purified antibodies directed to PTHrP(1-40) and PTHrP(57-80) and PTHrP(1-84) as the standard (detection limit = 0.2pmol/L)<br>- *Positive controls:* NR<br>- *Negative controls:* healthy normocalcemic subjects<br>- *Reproducibility assessments:* Intra- and Inter-assay variation | Continuous variables were analyzed by either the Wilcoxon test or the Mann-Whitney U test | *Method of selection:* NR<br>*Selected variables:* NR | Breast cancer eucalcemic patients (*n*=2) had PTHrP levels below the limit of detection<br><br>Breast cancer patients with hypercalcemia (*n*=2) had PTHrP-N = 3.9 and 2.5pmol/L and PTHrP-I = 3.9 and 2.1 |

| |
|---|
| coefficients from replicate determinations on plasma samples containing PTHrP in the range 1-100pM were <10% for both assays<br>- *Statistical analysis:* quantitative |

NOTE: sums of percentages could differ from 100 due to rounding of numbers.

ABBREVIATIONS: NR = not reported; NA = not applicable; ER = estrogen receptor; PR = progesterone receptor; HER2 = human epidermal growth factor receptor 2; IHC = immunohistochemistry; IRMA = immunoradiometric assay; RIA = radioimmunoassay; IFMA = immunofluorometric assay; OR = odds ratio; *GAPDH = Glyceraldehyde-3-phosphate dehydrogenase*

[A] According to the American Joint Committee on Cancer staging system;

[B] According to the method of Bloom and Richardson;

[C] According to Mercer *et al.,* tumors with hormone receptor levels ≥10fmol/mg protein were considered to be ER and/or PR positive while tumors with hormone receptor levels <5fmol/mg protein were considered to be negative. Tumors with hormone receptor levels between 5 and 10fmol/mg protein were considered equivocal,

[D] According to a modified version of Bloom and Richardson method;

[E] Assessed by dextran-coated charcoal method and Scatchard analysis, tumors containing hormone receptor levels ≥5fmol/mg protein were considered positive;

[F] According to the Union for International Cancer Control classification system;

[G] Assessed by dextran-coated charcoal method, 10fmol/mg protein was used as threshold;
